# Methylene-Bridged Bis(imidazoline)-Derived 2-Oxopyrimidinium Salts as Catalysts for Asymmetric Michael Reactions**

**DOI:** 10.1002/anie.201300614

**Published:** 2013-05-27

**Authors:** Andrey E Sheshenev, Ekaterina V Boltukhina, Andrew J P White, King Kuok (Mimi) Hii

**Affiliations:** Department of Chemistry, Imperial College LondonExhibition Road, South Kensington, London SW7 2AZ (UK) E-mail: mimi.hii@imperial.ac.uk Homepage: http://www.ch.ic.ac.uk/mimi

**Keywords:** asymmetric synthesis, diastereoselectivity, enantioselectivity, Michael addition, phase-transfer catalysis

Conjugate addition of glycine-derived imine esters (**1**) to Michael acceptors can generate highly functionalized molecules with up to three contiguous stereogenic centers (Scheme 1), which is an attractive strategy for assembling molecular complexity from achiral precursors in a single step without byproducts.^1^

**Scheme 1:**
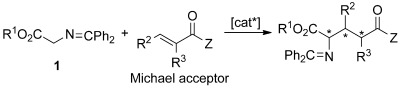
Conjugate addition of glycine imine esters (**1**) to α,β-unsaturated carbonyl compounds.

Presently, nonmetal-based phase-transfer catalysts (PTCs) and organocatalysts^2^ have been deployed to great effect for these reactions.^3^ Corey et al. first reported the conjugate addition of **1** to acrylates and enones with notable enantioselectivity (>90 % *ee*) in the presence of an N-alkylated cinchonidine salt.^4^ Subsequently, the scope of the reaction was expanded with other modified cinchona alkaloids^5^ as well as new catalysts, comprising largely of quaternary bis(ammonium) and N-spiro ammonium moieties derived from tartrates,^6^ axially-chiral 1,1′-biaryl units,5f, 7 inositol-derived crown ethers,^8^ and a calix[4]arene amino acid.^9^ The use of these pH-neutral catalysts requires strong bases to generate the nucleophile, thus very low temperatures (typically −40 to −78 °C) were necessary to suppress competitive reactions.

In contrast, deployment of catalysts containing planar nitrogen entities received far less attention. In 2001, Ishikawa et al. showed that the modified guanidine derivative **2** (Figure 1) can be employed as a chiral Brønsted superbase for Michael reactions.^10^ The basicity of the catalyst allowed reactions to proceed under ambient conditions in good enantioselectivities, but reactions were sluggish. They required days to complete even without using any solvents, which may account for the lack of development of this type of catalyst in the ensuing decade. However, two recent breakthroughs have rekindled interest in this area, with independent reports of the pentanidium derivative **3**^11^ and cyclopropenimine **4**^12^ (Figure 1), which can deliver very favorable catalytic turnovers and enantioselectivities between room temperature and −20 °C.

**Figure 1:**
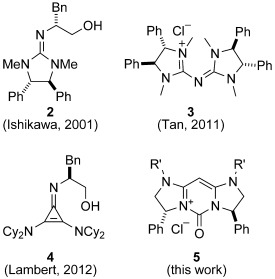
Effective catalysts containing planar nitrogen atoms for asymmetric Michael reactions.

Herein, we describe the preparation of a family of structurally novel 2-oxopyrimidinium salts (**5**), and their performance as asymmetric PTCs in the conjugate addition of the glycine imine ester **1 a** (R^1^=*t*Bu) to vinyl ketone and chalcone derivatives.

The structure of **5** is derived from chiral methylene-bridged bis(imidazolines) (MBI), previously reported by Pfaltz and co-workers as a variant of bisoxazoline ligands for asymmetric catalysis.^13^ The C_2_-symmetrical architecture was assembled in five steps from the N-Boc-protected amino acids **6 a**–**c** (Scheme 2): the MBIs **10 a**–**j** were prepared by a modified literature procedure, and subsequently treated with triphosgene to afford the 2-oxo-pyrimidinium salts **5**. Single-crystal X-ray diffraction analysis of the *n*-butyl-substituted derivative **5 b** (Figure 2) revealed planar fused rings, corroborating a highly mesomeric tricyclic system.

**Figure 2:**
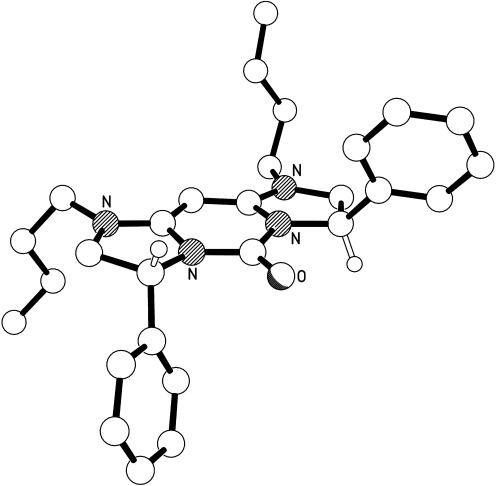
Structure of (*R*,*R*)-**5 b** as determined by single-crystal X-ray crystallography (nonstereogenic hydrogen atoms omitted).^17^

**Scheme 2:**
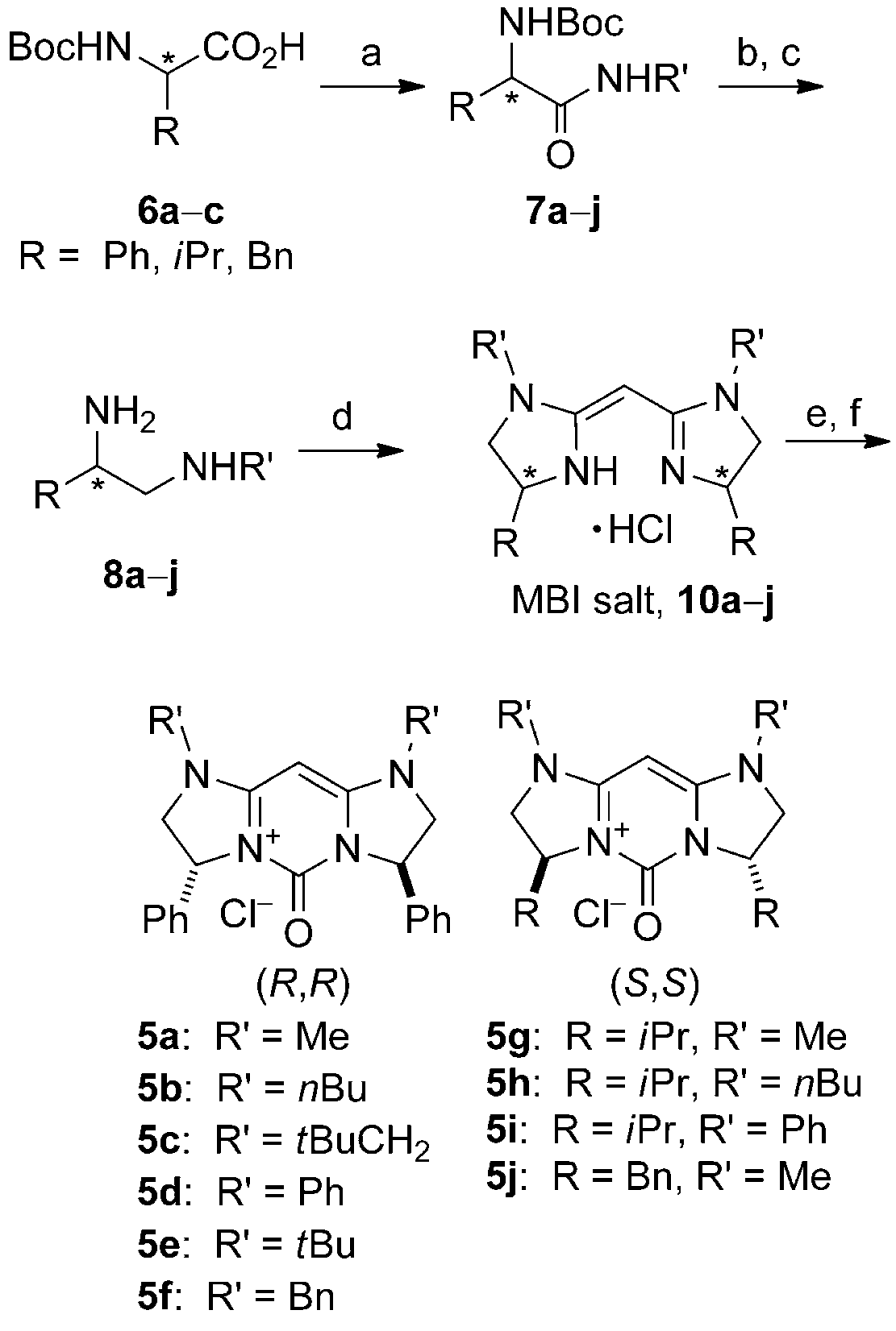
Synthesis of chiral 2-oxopyrimidinium salts (**5**) from the N-Boc amino acids **6 a**–**c**: a) *N*-methylmorpholine, ClCO_2_*i*Bu, R′NH_2_ (74–96 %). b) AcCl, MeOH, 0 °C→RT. c) LiAlH_4_, THF, reflux (73–98 % over 2 steps). d) CH_2_(C(=NH)OEt)_2_**⋅**2 HCl (**9**), CH_2_Cl_2_, RT→reflux, (65–100 %). e) 10 % aq. NaOH/CH_2_Cl_2_. f) triphosgene, CH_2_Cl_2_, NEt_3_, 0 °C→RT (84–93 % over 2 steps). Boc=*tert*-butoxycarbonyl.

The addition of the *tert*-butyl ester glycinate benzophenone Schiff base (**1 a**; Table 1) to MVK (**11 a**) in the presence of **5 a** was chosen for reaction optimization, including extensive screening of solvent, dilution, inorganic base, catalyst loading, and stoichiometry (see Tables S1–S6, in the Supporting Information). Under phase-transfer conditions, the solvent exerts an important effect. When using Cs_2_CO_3_ as a base at a 5 mol % catalyst loading, the reaction was complete within an hour at ambient temperature in toluene or xylene, furnishing the Michael adduct **12 a** with greater than 80 % *ee* (Table 1, entries 1 and 2). In comparison, the use of dichloromethane was detrimental for both productivity and enantiodiscrimination (entry 3).

**Table 1:** Conjugate addition of *tert*-butyl glycinate benzophenone Schiff base (1 a) to MVK (11 a)^[a]^

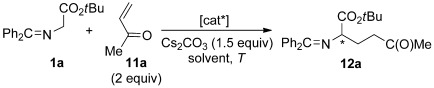						

Entry	Catalyst^[b]^ (mol %)	Solvent	*T* [°C]	*t* [min]	Yield [%]^[c]^	*ee* [%]^[d]^
1	**5 a** (5)	toluene	RT	65	82	81 (*S*)
2	**5 a** (5)	*o*-xylene	RT	45	78	82 (*S*)
3	**5 a** (5)	CH_2_Cl_2_	RT	120	85	2 (*S*)
4	**5 b** (5)	toluene	RT	65	88	84 (*S*)
5	**5 c** (5)	toluene	RT	65	85	88 (*S*)
6	**5 d** (5)	toluene	RT	65	84	24 (*R*)
7	**5 e** (5)	toluene	RT	65	87	6 (*S*)
8	**5 f** (5)	toluene	RT	35	82	79 (*S*)
9	**5 g** (5)	toluene	RT	45	87	35 (*R*)
10	**5 h** (5)	toluene	RT	45	86	32 (*R*)
11	**5 i** (5)	toluene	RT	65	80	16 (*S*)
12	**5 j** (5)	toluene	RT	45	90	48 (*R*)
13	**5 c** (2)	toluene	0	300	85	93 (*S*)
14	**5 c** (2)	toluene	−20	1440	76^[e]^	93 (*S*)
15	**5 c** (2)	o-xylene	0	300	79	93 (*S*)

[a] Reactions were performed using **1 a** (0.05 mmol), **11 a** (0.1 mmol), and Cs_2_CO_3_ (0.075 mmol) in 0.5 mL of solvent. [b] Catalyst loading is indicated within parentheses. [c] Yield of the isolated product after purification by column chromatography. Reactions were complete (TLC), unless otherwise indicated. [d] Determined by HPLC using a chiral stationary phase. Absolute configuration assigned by comparison with literature data. [e] 97 % conversion (^1^H NMR spectroscopy).

As might be expected, variations in the structure of the catalyst have a profound effect on the reaction outcome. Extending the N-alkyl chain (from methyl to *n*-butyl and neopentyl) led to an increase in the product *ee* value to 88 % (Table 1, entries 4 and 5), whereas the substitution with phenyl and bulky *tert*-butyl groups has the opposite effect (entries 6 and 7). The level of enantioselectivity was restored with the N-benzyl derivative **5 f**, which also afforded a faster reaction (entry 8). In contrast, attempts to replace the phenyl substituents on the stereogenic centers of the catalyst with isopropyl (entries 9–11) or benzyl (entry 12) groups did not lead to any improvement. Concurrently, the study also revealed a highly synergistic relationship between the N and C substituents in determining the stereochemical outcome. For catalysts containing phenyl substituents at the stereogenic centers, the selectivity for the *S* isomer can be overturned by changing the N-alkyl substituent to a phenyl group (Table 1, entry 6 versus entries 1, 4, 5, 7, and 8). The same effect was also observed for the isopropyl-substituted series (entry 11 versus entries 9 and 10).

Eventually, the best yield and *ee* value were attained with 2 mol % of **5 c** within 2 hours at 0 °C in toluene or *o*-xylene (Table 1, entries 13 and 15). Additional lowering of temperature led only to a slower reaction with no detectable improvement in the product *ee* value (entry 14). With these optimized reaction conditions in hand, five additional vinyl ketone substrates (**11 b**–**f**) were evaluated (Table 2). In all cases, the product can be obtained with good to excellent yields and enantioselectivities, which compare favorably with previously reported systems.

**Table 2:** Addition of 1 a to vinyl ketones catalyzed by 5 c^[a]^

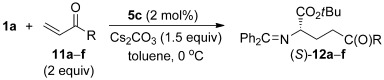					

Entry	R	Product	*t* [h]	Yield [%]^[b]^	*ee* [%]^[c]^
1	Me	**12 a**	5	85	93
2	Et	**12 b**	10	92	90
3	*n*Pr	**12 c**	3	95	92
4	CH_2_CH_2_Ph	**12 d**	2	94	85
5	Ph	**12 e**	12	82	80
6	2-naphthyl	**12 f**	24	76	83

[a] Reactions were performed using **1 a** (0.05 mmol), **11** (0.1 mmol), **5 c** (1 μmol), and Cs_2_CO_3_ (0.075 mmol) in toluene (0.5 mL) at 0 °C. [b] Yield of the isolated product after purification by column chromatography. [c] Determined by HPLC using a chiral stationary phase. Absolute stereochemistry established by comparison with literature data.

Chalcone derivatives are a particularly challenging class of Michael acceptors. To date, only two catalysts have been reported to have broad generality for these substrates: a dimeric binol-derived (binol=2,2′-dihydroxy-1,1′-binaphthyl) N-spiroammonium salt (≤96 % *de*, 93 % *ee*),7e and the pentanidium derivative **3** (100 % *de*, ≤94 % *ee*).11a Hence, we were delighted to find that **5 c** is not only catalytically active, but furnishes the Michael adducts as single diastereoisomers with excellent yields and enantioselectivities (Table 3) under adjusted reaction conditions (see Table S7 in the Supporting Information). In terms of reaction scope, a variety of aryls and heteroaryls can be accommodated within the Michael acceptor. Reactions catalyzed by the 2-oxopyrimidinium salt **5 c** appeared to be faster than those mediated by the pentanidium catalyst **3**. Under practically identical reaction conditions, reactions were complete within 6 hours, compared to the 10 or more hours provided in the earlier report.

**Table 3:** Conjugate addition of 1 a to chalcone derivatives 13^[a]^

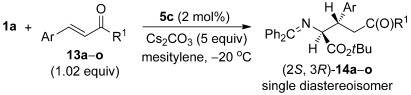						

Entry	Ar	R^1^	Product	*t* [h]	Yield [%]^[b]^	*ee* [%]^[c]^
1	Ph	Ph	**14 a**	3	98	93
2	4-NO_2_C_6_H_4_	Ph	**14 b**	2	96	90
3	4-ClC_6_H_4_	Ph	**14 c**	4	94	91
4	2-F-5-BrC_6_H_3_	Ph	**14 d**	5	94	93
5	4-CF_3_C_6_H_4_	Ph	**14 e**	2	92	91
6	2-naphthyl	Ph	**14 f**	4	96	85
7	2-pyridyl	Ph	**14 g**	3	96	93
8	3-pyridyl	Ph	**14 h**	3	98	93
9	Ph	4-BrC_6_H_4_	**14 i**	6	88	85
10	Ph	2-naphthyl	**14 j**	6	92	86
11	Ph	4-ClC_6_H_4_	**14 k**	2	90	87
12	Ph	2-furyl	**14 l**	3	87	90
13	Ph	2-thienyl	**14 m**	2	93	85
14	Ph	4-CF_3_C_6_H_4_	**14 n**	2	90	83
15	Ph	4-pyridyl	**14 o**	3	96	88

[a] Reactions were performed using **1 a** (0.05 mmol), **13** (0.051 mmol), **5 c** (1 μmol) and Cs_2_CO_3_ (0.25 mmol) in mesitylene (0.5 mL) at −20 °C for the indicated time. [b] Yield of the isolated product after purification by column chromatography. [c] HPLC using a chiral stationary phase. Absolute stereochemistry established by comparison with literature data.

As the Michael adduct of the methoxy-substituted chalcone **13 p** was prone to retro-Michael reaction at ambient temperature, it was subjected to deprotection/cyclization to afford the dihydropyrrole derivative **15 a** prior to analysis [Scheme 3, Eq. (1)]. This strategy was employed to achieve an expedient synthesis of a dihydropyrrole derivative on a semipreparative scale [Eq. (2)]. Using a catalytic loading of 0.5 mol %, a telescoped Michael addition/deprotection/cyclization sequence furnished the product **15 b** within a reasonable timescale in high yields and enantiopurity, without the need for column chromatography.

**Scheme 3:**
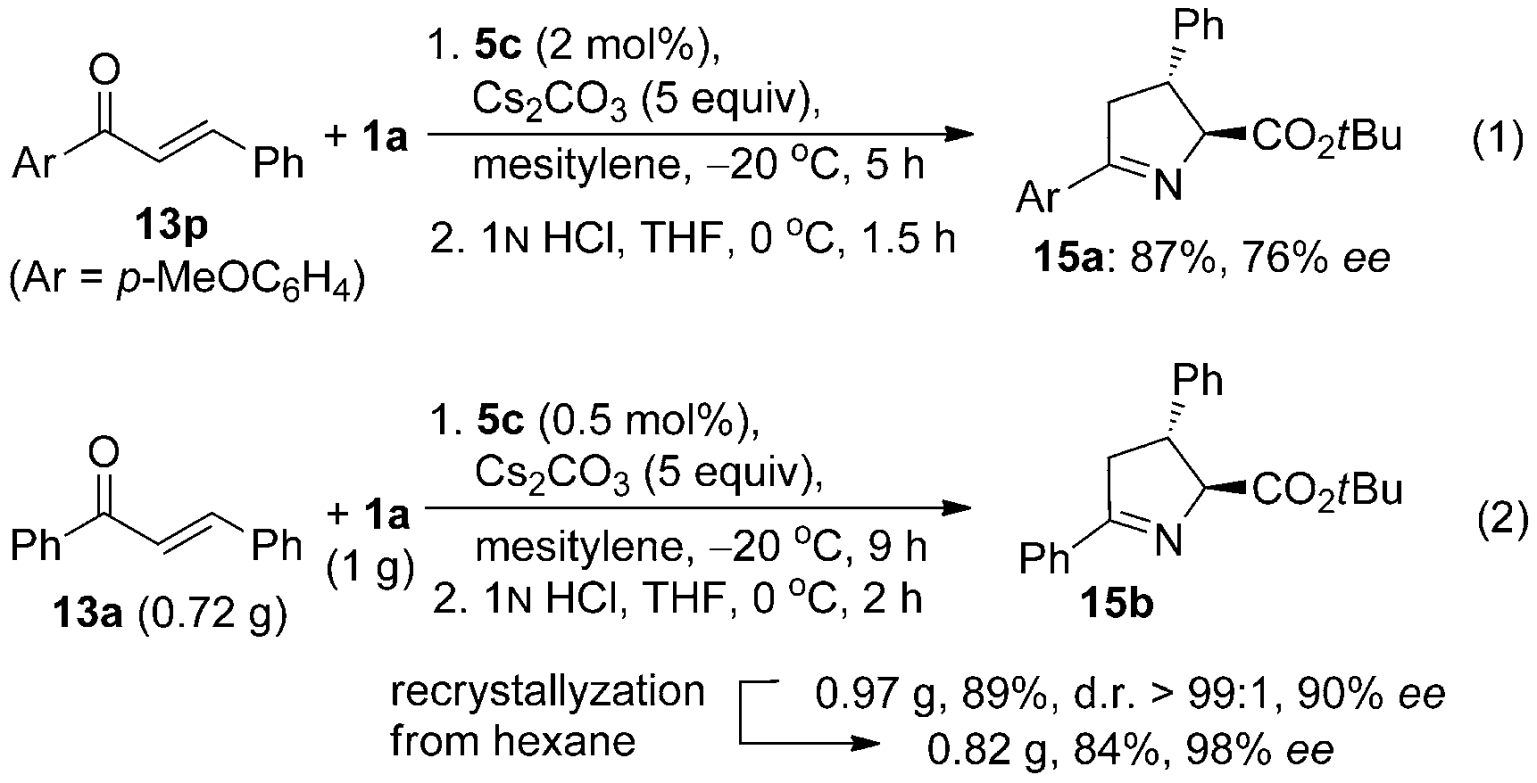
Synthesis of chiral dihydropyrrole derivatives **15**.

The synthetic utility of the methodology was further demonstrated by the preparation of the novel proline/nicotine hybrid molecule (2*S*,3*R*,5*S*)-**16**,^14^ containing three well-defined stereogenic centers, in just three steps (Scheme 4). Following the previous procedure, the dihydropyrrole intermediate **15 c** was obtained in good yield and selectivity. Reduction of the imine moiety with sodium borohydride furnished (2*S*,3*R*,5*S*)-**16** as a single diastereoisomer with excellent optical purity (94 % *ee*).^15^

**Scheme 4:**
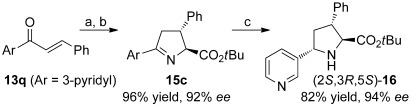
Synthesis of the novel nicotine/proline hybrid (2*S*,3*R*,5*S*)-**16**: a) **1 a**, **5 c** (2 mol %), mesitylene, −20 °C, 3 h. b) 1 N HCl, THF, 0 °C, 1.5 h. c) NaBH_4_, MeOH, 0 °C→RT, 24 h. THF=tetrahydrofuran.

In conclusion, a new family of 2-oxopyrimidinium salts has been shown to be highly effective catalysts for the asymmetric Michael addition of a glycine imine ester to vinyl ketones and chalcones under synthetically practical conditions. Although these catalysts contain only planar nitrogen moieties (Figure 2), they are entirely devoid of Brønsted basicity.^16^ Thus, it is tantalizing to suggest that these first-in-class compounds may offer a greater reaction scope, particularly towards substrates with base-labile moieties. Future work will include delineating the mechanism of these reactions, and applications in other asymmetric processes.

## Supplementary material

As a service to our authors and readers, this journal provides supporting information supplied by the authors. Such materials are peer reviewed and may be re-organized for online delivery, but are not copy-edited or typeset. Technical support issues arising from supporting information (other than missing files) should be addressed to the authors.
