# Supplementary material for: Methylene-Bridged Bis(imidazoline)-Derived 2-Oxopyrimidinium Salts as Catalysts for Asymmetric Michael Reactions
Source: Angew Chem Int Ed Engl. 2013 May 27;52(27):6988–91. doi: 10.1002/anie.201300614 (PMC3749446; doi:10.1002/anie.201300614)
Supplement: Supplementary file 1 [file anie0052-6988-SD1.pdf]

Angewandte Chemie
Eine Zeitschrift der Gesellschaft Deutscher Chemiker

Supporting Information

© Wiley-VCH 2013

69451 Weinheim, Germany

Methylene-Bridged Bis(imidazoline)-Derived 2-Oxopyrimidinium Salts as Catalysts for Asymmetric Michael Reactions**

*Andrey E. Sheshenev, Ekaterina V. Boltukhina, Andrew J. P. White, and King Kuok (Mimi) Hii**

anie_201300614_sm_miscellaneous_information.pdf

Supporting Information

Table of Contents

**S2**	General Information
**S3–S18**	Synthetic protocols for compounds **7**, **8**, **10**, **5**
**S18–S25**	Synthetic protocols for compounds **11** and **13**
**S25–S37**	Catalytic experiments, synthesis of compounds **12**, **14**, **15** and **16**
**S38**	Determination of the absolute configuration of adducts **12** and **14**
**S39–S40**	NOESY experiments for compounds **15c** and **16**
**S41–S43**	Crystallographic data for (*R,R*)-**5b**
**S44–S47**	Optimization tables
**S48**	References
**S49–S74**	Chiral HPLC chromatograms
**S75-S140**	NMR spectra of novel compounds

General Information

Solvents were dried by passing through the columns of molecular sieves in a solvent purification system (Innovative Technology Inc.). Unless otherwise stated, materials obtained from commercial suppliers were used without further purification. Preparative separations were performed by silica gel gravity column chromatography (Silica gel 60, Fluka or Kiesgel 60). TLC were visualised with molybdate dip or UV light. ^{1}H NMR (400 MHz, 500 MHz), ^{13}C NMR (100 MHz, 125 MHz) and ^{19}F {^{1}H} NMR (377 MHz) spectra were recorded at 25 °C on Bruker Avance I™ 400 MHz or 500 MHz spectrometers. Chemical shifts (δ) were reported in ppm relative to $CDCl_3$ (δ = 7.26) or CD_3CN (δ = 1.94) for ^{1}H NMR and to $CDCl_3$ (δ = 77.00) or CD_3CN (δ = 1.32 and 118.26) for ^{13}C NMR. IR spectra were recorded with neat solid/liquid samples using a Perkin Elmer Spectrum 100 spectrometer fitted with an ATR accessory. High-resolution mass spectra were recorded at the Imperial College London Mass Spectrometry Service Unit using EI, CI or ESI ionization methods. Optical rotation was measured on Autopol IV polarimeter. Melting points were determined using an Electrothermal Gallenhamp apparatus fitted with a calibrated thermometer with an error of ±2 °C and are uncorrected. HPLC analyses were performed on Hewlett Packard HP1050 instruments. Elemental analysis was performed at London Metropolitan University.

Diethyl malonimidate dihydrochloride **9**, diphenylchalcone **13a**, methyl-, ethyl- and propyl-vinyl ketones were purchased from Sigma-Aldrich.

N-Boc-protected amino acids **6a-c** were prepared as described earlier.[1]

Preparation of *N*-Boc-protected amino amides 7

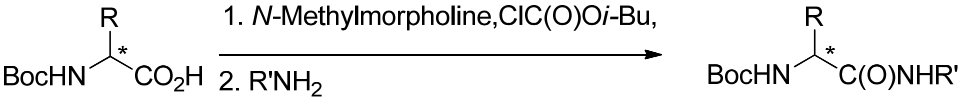

6a-c **7a-j**

General procedure: N-Methylmorpholine (1 mol equiv.) and *iso*-butylchloroformate (1 mol equiv.) were successively added to a solution of the corresponding acid **6** (1 mol equiv.) in dry THF (100 mL per 50 mmol of **6**) at –20 °C. After 5 min at –20 °C the corresponding amine (1.2 mol equiv.) was added and the reaction mixture was stirred at –20 °C for 2 h prior to the addition of 5% aq. $NaHCO_3$ (100 mL per 50 mmol of **6**). After 30 min at r.t. the solution was extracted with CH_2Cl_2 (3×100 mL), and the combined organic phases were washed with 5% aq. $NaHCO_3$ (100 mL), 5% aq. HCl (100 mL) and water (100 mL), dried over $MgSO_4$ and concentrated. The crude product was purified by recrystallization from CH_2Cl_2/pentane mixture (1:1 v/v) to provide amides **7a-j** as white solids.

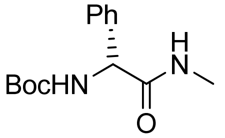

(*R*)-2-*N*-Boc-*N*-methyl-2-phenyl-acetamide, 7a. White powder; yield 96%; mp 133.5–136 °C [lit.[2] 136 °C]; $[\alpha]_D^{20}$ = –135.9 (c 0.78, $CHCl_3$) [lit.[2] –108.4 (c 1.0, MeOH)]; ^1H NMR (400 MHz, $CDCl_3$) δ 1.41 (s, 9H, *t*-Bu), 2.78 (d, J = 4.3 Hz, 3H, NCH_3), 5.17 (br s, 1H, NH), 5.89 (br s, 1H, CH), 6.02 (br s, 1H, NH), 7.30–7.36 (m, 5H, Ph); ^{13}C NMR (100 MHz, $CDCl_3$) δ 26.5 ($NHCH_3$), 28.3 ($OC(CH_3)_3$), 58.4 (CHNH), 80.02 ($OC(CH_3)_3$), 127.2, 128.2, 129.0, 138.6 (Cq), 155.2 (CO_2), 170.7 (C(O)NH); IR/cm^{-1}: 3339 m, 3301 m, 2983 w, 2937 w, 1655 s, 1526 s, 1364 m, 1251 m, 1244 m, 1162 s, 1060 m, 948 w, 873 w, 693 s; HRMS (ESI) *m/z*: found: 287.1362, calcd for $C_{14}H_{20}O_3N_2Na$ $[M+Na]^+$: 287.1372.

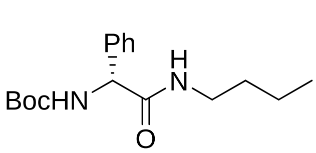

(*R*)-2-*N*-Boc-*N*-(*n*-butyl)-2-phenyl-acetamide, 7b. White powder; yield 93%; mp 89–92 °C; $[\alpha]_D^{20}$ = –100.0 (c 1.03, $CHCl_3$); ^1H NMR (400 MHz, $CDCl_3$) δ 0.85 (t, J = 7.3 Hz, 3H, CH_3), 1.23 (sextet, J = 7.3 Hz, 2H, CH_2CH_3), 1.35–1.46 (m, 11H, *t*-Bu & CH_2), 3.19–3.24 (m, 2H, NCH_2), 5.13 (br s, 1H, NH), 5.87 (br s, 2H, CH & NH), 7.28–7.36 (m, 5H, Ph); ^{13}C NMR (100 MHz, $CDCl_3$) δ 13.6 (CH_3), 19.8 (CH_2), 28.3 ($OC(CH_3)_3$), 31.4 (CH_2), 39.5 (CH_2N), 58.5 (CH), 79.9 ($OC(CH_3)_3$), 127.1, 128.2, 128.9, 138.6 (Cq), 155.2 (CO_2), 170.0 (C(O)NH); IR/cm^{-1}: 3314 m, 2973 m, 2934 m, 1696 w, 1654 s, 1516 s, 1362 m, 1247 m, 1165 s, 1024 m, 877 w, 729 m, 696 s; HRMS (ESI) *m/z*: found: 307.2016, calcd for $C_{17}H_{27}O_3N_2$ $[M+H]^+$: 307.2022.

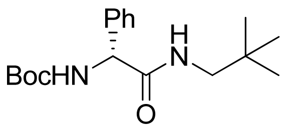

(*R*)-2-*N*-Boc-*N*-neopentyl-2-phenyl-acetamide, 7c. White solid; yield 94%; mp 132–133 °C; $[\alpha]_D^{20}$ = –95.6 (c 0.92, $CHCl_3$); ^1H NMR (400 MHz, $CDCl_3$) δ 0.77 (s, 9H, $CH_2C(CH_3)_3$), 1.41 (s, 9H, $OC(CH_3)_3$), 2.91 (dd, J = 13.4, 5.6 Hz, 1H, CH_2), 3.14 (dd, J = 13.4, 7.2 Hz, 1H, CH_2), 5.13 (br s, 1H, NH), 5.81 (br s, 1H, CH), 5.90 (br s, 1H, NH), 7.30–7.39 (m, 5H, Ph); ^{13}C NMR (100 MHz, $CDCl_3$) δ 26.9, 28.3, 32.0 ($CH_2C(CH_3)_3$), 50.7 (CH_2), 58.8 (CH), 79.9 ($OC(CH_3)_3$), 127.1, 128.3, 129.0, 138.8 (Cq), 155.2 (CO_2), 170.1 (C(O)NH); IR ($CHCl_3$) cm^{-1}: 3436 w, 3291 m, 2962 w, 1711 m, 1697 m, 1649 s, 1500 s, 1365 m, 1240 m, 1161 s, 897 w, 876 w, 698 s; HRMS (ESI) *m/z*: found: 321.2178, calcd for $C_{18}H_{29}O_3N_2$ $[M+H]^+$: 321.2178.

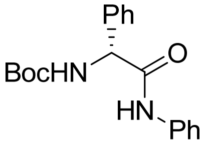

(*R*)-2-*N*-Boc-*N*-phenyl-2-phenyl-acetamide, 7d. White powder; yield 78%; mp 120–121 °C; $[\alpha]_D^{14}$ = –107.3 (c 1.02, $CHCl_3$); ^1H NMR (400 MHz, $CDCl_3$) δ 1.42 (s, 9H, *t*-Bu), 5.43 (br s, 1H, NH), 5.88 (br s, 1H, CH), 7.04 (t, J = 7.4 Hz, 1H), 7.22 (t, J = 7.8 Hz, 2H), 7.27–7.38 (m, 3H), 7.41 (d, J = 8.0 Hz, 2H), 7.46 (d, J = 6.4 Hz, 1H), 8.13 (br s, 1H, NH); ^{13}C NMR (100 MHz, $CDCl_3$) δ 28.3 ($OC(CH_3)_3$), 59.3 (CHNH), 80.6 ($OC(CH_3)_3$), 119.9, 124.5, 127.4, 128.6, 128.9, 129.1, 137.4 (Cq), 137.5 (Cq), 155.6 (CO_2), 168.6 (C(O)NH); IR/cm^{-1}: 3318 m, 3065 w, 2978 m, 2938 w, 1684 m, 1661 s, 1601m, 1517 s, 1496 m, 1442 m, 1365 m, 1249 m, 1166 s, 1078 m, 1052 m, 1027 m, 984 m, 887 m, 869 m, 753 s, 694 s; HRMS (ESI) *m/z*: found: 327.1716, calcd for $C_{19}H_{23}O_3N_2$ $[M+H]^+$: 327.1709.

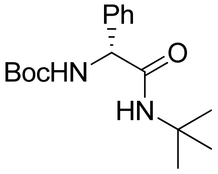

(*R*)-2-*N*-Boc-*N*-(*tert*-butyl)-2-phenyl-acetamide, 7e. White powder; yield 74%; mp 137–138 °C; $[\alpha]_D^{14}$ = –86.8 (c 1.08, $CHCl_3$); ^1H NMR (400 MHz, $CDCl_3$) δ 1.29 (s, 9H, N*t*-Bu), 1.40 (s, 9H, O*t*-Bu), 5.01 (br s, 1H, NH), 5.45 (br s, 1H, CH), 5.83 (br s, 1H, NH), 7.28–7.35 (m, 5H, Ph); ^{13}C NMR (100 MHz, $CDCl_3$) δ 28.3 ($C(CH_3)_3$), 28.6 ($C(CH_3)_3$), 51.7 ($NHC(CH_3)_3$), 58.8 (CHNH), 79.9 ($OC(CH_3)_3$), 127.2, 128.2, 129.0, 139.0 (Cq), 155.2 (CO_2), 169.1 (C(O)NH); IR/cm^{-1}: 3357 m, 3279 m, 3068 w, 2976 m, 2934 w, 1709 m, 1689 s, 1665 m, 1644 s, 1560 m, 1529 s, 1498 m, 1478 m, 1453 m, 1391 m, 1361 s, 1295 m, 1250 m, 1163 s, 1078 m, 1050 m, 1026 m, 915 m, 890 m, 853 m, 811 m, 779 m, 703 s, 693 s; HRMS (ESI) *m/z*: found: 307.2021, calcd for $C_{17}H_{27}O_3N_2$ $[M+H]^+$: 307.2022.

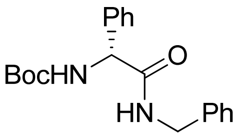

(*R*)-2-*N*-Boc-*N*-benzyl-2-phenyl-acetamide, 7f. White powder; yield 79%; mp 118–119 °C; $[\alpha]_D^{14}$ = –79.2 (c 1.06, $CHCl_3$); ^1H NMR (400 MHz, $CDCl_3$) δ 1.39 (s, 9H, *t*-Bu), 4.39 (d, *J* = 5.8 Hz, 2H, CH_2), 5.21 (br s, 1H, NH), 5.86 (br s, 1H, CH), 6.31 (br s, 1H, NH), 7.10–7.12 (m, 1H, Ph), 7.20–7.37 (m, 4H, Ph); ^{13}C NMR (100 MHz, $CDCl_3$) δ 28.3 (OC($C$$H_3$)$_3$), 43.6 ($CH_2$), 58.6 (CHNH), 80.1 (O*C*(CH_3)$_3$), 127.2, 127.5, 128.4, 128.6, 129.0, 137.7 (Cq), 138.4 (Cq), 155.2 (*C*O_2), 170.2 (C(O)NH); IR/cm^{-1}: 3302 m, 2979 m, 2931 w, 1698 m, 1654 s, 1519 m, 1497 m, 1455 m, 1392 m, 1367 m, 1353 m, 1249 m, 1164 m, 1080 m, 1060 m, 1023 m, 937 m, 896 m, 735 m, 676 s; HRMS (ESI) *m/z*: found: 341.1863, calcd for $C_{20}H_{25}O_3N_2$ $[M+H]^+$: 341.1865.

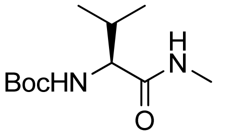

(*S*)-2-*N*-Boc-*N*,3-dimethylbutanamide, 7g. White solid; yield 83%; mp 128–130 °C [lit.[3] 132–133 °C (Et_2O/ petroleum ether)]; $[\alpha]_D^{20}$ = –11.9 (c 0.50, $CHCl_3$) [lit.[3] –11.7 (c 1.0, MeOH)]; ^1H NMR (400 MHz, $CDCl_3$) δ 0.91 (d, *J* = 6.8 Hz, 3H, CHC*H*$_3$), 0.95 (d, *J* = 6.8 Hz, 3H, CHC*H*$_3$), 1.44 (s, 9H, *t*-Bu), 2.03–2.27 (m, 1H, C*H*(CH_3)$_2$), 2.81 (d, *J* = 4.9 Hz, 3H, NCH_3), 3.88 (dd, *J* = 9.0, 6.4 Hz, 1H, NHC*H*), 5.10 (br s, 1H, NH), 6.18 (br s, 1H, NH); ^{13}C NMR (100 MHz, $CDCl_3$) δ 17.9 (CH_3), 19.3 (CH_3), 26.1 ($NHCH_3$), 28.3 (OC(*C*H_3)$_3$), 30.9 (*C*H(CH_3)$_2$), 60.1 (NHCH), 79.8 (O*C*(CH_3)$_3$), 155.9 (*C*O_2), 172.2 (C(O)NH); IR ($CHCl_3$) cm^{-1}: 3437 w, 3337 w, 2973 m, 1699 s, 1659 s, 1503 s, 1368 m, 1295 m, 1241 s, 1165 s, 1094 w, 1047 w, 1013 m, 919 w, 870 w, 687 m; HRMS (ESI) *m/z*: found: 253.1523, calcd for $C_{11}H_{22}O_3N_2Na$ $[M+Na]^+$: 253.1528.

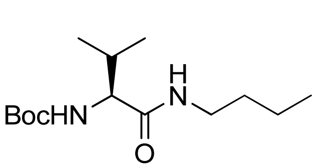

(*S*)-2-*N*-Boc-*N*-(*n*-butyl)-3-methylbutanamide, 7h. White solid; yield 94%; mp 117–119 °C [lit.[4] 113–116 °C]; $[\alpha]_D^{20}$ = –19.1 (c 0.51, $CHCl_3$) [lit.[4] $[\alpha]_D^{25}$ = –22.7 (2.2, MeOH)]; ^1H NMR (400 MHz, $CDCl_3$) δ 0.90–0.96 (m, 9H, CH_3), 1.28–1.39 (m, 2H, CH_2), 1.44 (s, 9H, *t*-Bu), 1.42–1.52 (m, 2H, CH_2), 2.02–2.26 (m, 1H, C*H*(CH_3)$_2$), 3.19–3.34 (m, 2H, C*H*$_2$NH), 3.84 (dd, *J* = 8.8, 6.5 Hz, 1H, C*H*NH), 5.11 (br s, 1H, NH), 6.04 (br s, 1H, NH); ^{13}C NMR (100 MHz, $CDCl_3$) δ 13.7 (CH_3), 17.9 (CH_3), 19.3 (CH_3), 20.0 (CH_2), 28.3 (OC(*C*H_3)$_3$), 30.7 (*C*H(CH_3)$_2$), 31.6 (CH_2), 39.1 (CH_2N), 60.3 (NHCH), 79.8 (O*C*(CH_3)$_3$), 155.9 (*C*O_2), 171.4 (C(O)NH); IR ($CHCl_3$) cm^{-1}: 3316 w, 3097 w, 2966 m, 2933 m, 1694 s, 1651 s, 1503 s, 1463 m, 1367 m, 1296 m, 1241 s, 1166 s, 1044 m, 1015 m, 924 w; HRMS (ESI) *m/z*: found: 295.1992, calcd for $C_{14}H_{28}O_3N_2Na$ $[M+Na]^+$: 295.1998.

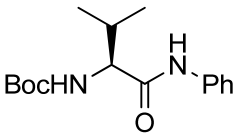

(*S*)-2-*N*-Boc-*N*-phenyl-3-methylbutanamide, 7i. White powder; yield 89%; mp 139–143 °C [lit.[5] 122–124 °C]; $[\alpha]_D^{20}$ = –32.5 (c 0.80, $CHCl_3$) [lit.[5] –36.7 (c 1.0,

$CHCl_3$)]; ^{1}H NMR (400 MHz, $CDCl_3$) (two rotamers) δ 0.96–1.04 (m, 6H, CH_3), 1.44 (s, 9H, $C(CH_3)_3$), 1.93–2.23 (m, 1H, $CH(CH_3)_2$), 3.95–4.07 (m, 1H, *CH*NH), 5.31 (br s, 1H, NH), 7.06–7.09 (m, 1H, Ph), 7.27–7.50 (m, 4H, Ph), 8.32 (br s, 1H, NH); ^{13}C NMR (two rotamers) (100 MHz, $CDCl_3$) δ 18.2 (CH_3), 19.1 (CH_3), 19.4, 28.0, 28.3 ($OC(CH_3)_3$), 30.7 ($CH(CH_3)_2$), 61.0 (NHCH), 80.3 ($OC(CH_3)_3$), 112.7, 118.6, 120.0, 123.3, 124.3, 128.7, 128.9, 129.0, 137.6, 139.2, 156.3 (CO_2), 170.3 (C(O)NH); IR/cm^{-1}: 3661 w, 3303 m, 2973 m, 1662 s, 1602 m, 1500 s, 1444 m, 1366 m, 1291 m, 1244 m, 1164 s, 1045 m, 937 w, 753 s, 690 s; HRMS (ESI) *m/z*: found: 293.1875, calcd for $C_{16}H_{24}O_3N_2Na$ $[M+H]^+$: 293.1865.

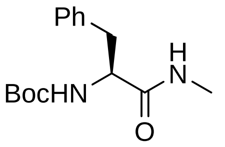

(*S*)-2-*N*-Boc-*N*-methyl-3-phenyl-propanamide, 7j. White solid; yield 95%; mp 137–139 °C [lit.[2] 142–143 °C]; $[\alpha]_D^{20}$ = +9.8 (c 0.82, $CHCl_3$) [lit.[2] +12.4 (c 1.0, MeOH)]; ^{1}H NMR (400 MHz, $CDCl_3$) δ 1.40 (s, 9H, *t*-Bu), 2.72 (d, J = 4.9 Hz, 3H, Me), 3.05 (d, J = 5.8 Hz, 2H, CH_2Ph), 4.30–4.31 (m, 1H, *CH*NH), 5.09 (br s, 1H, NH), 5.86 (br s, 1H, NH), 7.18–7.31 (m, 5H, Ph); ^{13}C NMR (100 MHz, $CDCl_3$) δ 26.1 (CH_3), 28.2 (CH_3), 38.8 (CH_2), 55.9 (CH), 80.1 ($OC(CH_3)_3$), 126.9, 128.6, 129.2, 136.8 (Cq), 155.4 (CO_2), 171.7 (C(O)NH); IR/cm^{-1}: 3339 m, 3030 w, 2947 w, 1678 s, 1655 s, 1516 s, 1492 m, 1320 m, 1292 s, 1242 s, 1164 s, 1014 m, 978 w, 868 m, 753 s, 699 s; HRMS (ESI) *m/z*: found: 301.1529, calcd for $C_{15}H_{22}O_3N_2Na$ $[M+Na]^+$: 301.1528.

Synthesis of diamines 8

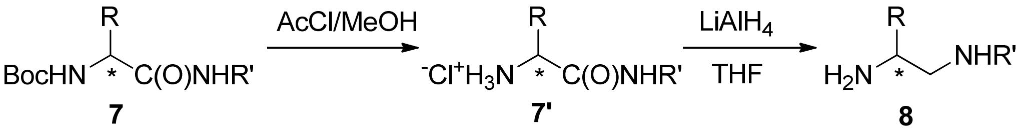

General procedure: 1. Deprotection. Freshly distilled acetyl chloride (5 mol equiv.) was added dropwise to dry MeOH (110 mL per 35 mmol of **7**) at 0–5 °C and the solution was stirred for 5 min at 5 °C. The corresponding amide **7** (1 mol equiv.) was then added in portions, the mixture was allowed to reach r.t., and stirred for 24 h. The solvent was removed under reduced pressure; the oily residue was triturated with dry ether and dried in high vacuum to afford the corresponding hydrochlorides **7'** in quantitative yields as colorless viscous glasses or white hydroscopic solids which were used for reduction without further purification.

2. Reduction. $LiAlH_4$ (2.5–4 mol equiv.) was added in portions (0.5 g each) to a solution of the corresponding hydrochloride **7'** (1 mol equiv.) in dry THF (100 mL per 50 mmol of **7'**) on cooling (0–5 °C). The reaction mixture was refluxed for 24 h, cooled to 0 °C and diluted with ether (150 mL)

followed by the careful addition of water (10 mL). The resulting suspension was subsequently quenched with 20% aq. NaOH (10 mL), water (10 mL) and stirred for 30 min at r.t. The white precipitate was filtered off and washed with ether (2×50 mL). The combined organic phases were dried over $MgSO_4$ and concentrated. The residue was distilled at reduced pressure or used without further purification.

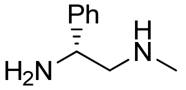

(*R*)-*N*[1]-methyl-1-phenylethane-1,2-diamine, 8a. Colorless oil; yield 77%; bp 85–86 °C (0.1 mmHg); $[\alpha]_D^{20}$ = –37.8 (c 0.90, $CHCl_3$); ^{1}H NMR (400 MHz, $CDCl_3$) δ 1.46 (br s, 3H, NH&NH_2), 2.44 (s, 3H, CH_3), 2.72 (dd, J = 11.8, 7.9 Hz, 1H, CH_2), 2.78 (dd, J = 11.8, 5.3 Hz, 1H, CH_2), 4.05 (dd, J = 7.9, 5.3 Hz, 1H, CH), 7.23–7.29 (m, 1H, Ph), 7.32–7.39 (m, 4H, Ph); ^{13}C NMR (100 MHz, $CDCl_3$) δ 36.4 (CH_3), 55.3 (CH_2), 60.0 (CH), 126.3, 127.1, 128.5, 144.7 (Cq); IR (film) cm^{-1}: 3281 w, 2889 w, 2844 w, 2789 w, 1602 s, 1492 w, 1451 m, 1349 w, 1111 m, 1067 m, 860 m, 757 s, 698 s, 615 m; HRMS (CI) *m/z*: found: 151.1238, calcd for $C_9H_{15}N_2$ $[M+H]^+$: 151.1235.

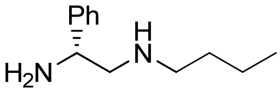

(*R*)-*N*[1]-butyl-1-phenylethane-1,2-diamine, 8b. Yellowish oil; yield 78%; bp 99–100 °C (0.08 mmHg); $[\alpha]_D^{20}$ = –26.8 (c 1.12, $CHCl_3$); ^{1}H NMR (400 MHz, $CDCl_3$) δ 0.90 (t, J = 7.3 Hz, 3H, CH_3), 1.28–1.50 (m, 7H, $CH_2CH_2CH_3$ & NH & NH_2), 2.61 (m, 2H, NH$CH_2$$CH_2$), 2.72 (dd, J = 11.8, 8.3 Hz, 1H, CHCH_2), 2.81 (dd, J = 11.8, 4.9 Hz, 1H, CHCH_2), 4.03 (dd, J = 8.3, 4.9 Hz, 1H, CH), 7.23–7.28 (m, 1H, Ph), 7.31–7.38 (m, 4H, Ph); ^{13}C NMR (100 MHz, $CDCl_3$) δ 14.0 (CH_3), 20.4 (CH_2), 32.2 (CH_2), 49.6 (CH_2), 55.6 (CH), 57.9 (CH_2), 126.3, 127.1, 128.4, 144.9 (Cq); IR (film) cm^{-1}: 3260 w, 3027 w, 2956 w, 2927 w, 2872 w, 1602 s, 1492 w, 1453 m, 1376 w, 1126 m, 1028 w, 855 m, 758 s, 698 s, 617 m; HRMS (ESI) *m/z*: found: 193.1703, calcd for $C_{12}H_{21}N_2$ $[M+H]^+$: 193.1705.

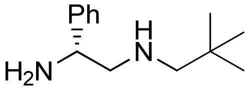

(*R*)-*N*[1]-neopentyl-1-phenylethane-1,2-diamine, 8c. Colorless oil; yield 76%; bp 96–98 °C (0.1 mmHg); $[\alpha]_D^{20}$ = –33.3 (c 1.17, $CHCl_3$); ^{1}H NMR (400 MHz, $CDCl_3$) δ 0.91 (s, 9H, *t*-Bu), 1.68 (s, 3H, NH&NH_2), 2.37 (d, J = 11.3 Hz, 1H, CH_2*t*-Bu), 2.38 (d, J = 11.3 Hz, 1H, CH_2*t*-Bu), 2.70 (dd, J = 12.0, 8.7 Hz, 1H, CHCH_2), 2.82 (dd, J = 12.0, 4.6 Hz, 1H, CHCH_2), 4.05 (dd, J = 8.7, 4.6 Hz, 1H, CH), 7.24–7.29 (m, 1H, Ph), 7.32–7.38 (m, 4H, Ph); ^{13}C NMR (100 MHz, $CDCl_3$) δ 27.7 ($C$$H_3$-*t*-Bu), 31.6 (*C*-*t*-Bu), 55.3 (CH), 58.7 (CH_2), 62.1 (CH_2), 126.4, 127.0, 128.4, 144.9 (Cq); IR (film) cm^{-1}: 3300 w, 2950 w, 2863 w, 2813 w, 16023 s, 1492 w, 1453 m, 1362 m, 1121 m, 1027 w, 860 m, 760 m, 698 s, 624 m; HRMS (ESI) *m/z*: found: 207.1858, calcd for $C_{13}H_{23}N_2$ $[M+H]^+$: 207.1861.

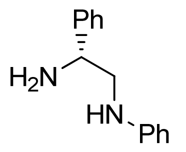

(*R*)-*N*[1],1-diphenyl-1,2-diamine, 8d. Viscous yellow oil; yield 84%; bp 145–147 °C (0.06 mmHg); $[\alpha]_D^{14}$ = +19.4 (c 1.44, $CHCl_3$); ^{1}H NMR (400 MHz, $CDCl_3$) δ 1.64 (br s, 2H, NH_2), 3.21 (dd, *J* = 12.7, 8.4 Hz, 1H, CH_2), 3.36 (dd, *J* = 12.7, 5.1 Hz, 1H, CH_2), 4.06 (br s, 1H, NH), 4.18 (dd, *J* = 8.4, 5.1 Hz, 1H, CH), 6.62–6.66 (m, 2H, Ph), 6.69–6.73 (m, 1H, Ph), 7.15–7.19 (m, 2H, Ph), 7.26–7.32 (m, 1H, Ph), 7.36 (d, *J* = 4.3 Hz, 4H, Ph); ^{13}C NMR (100 MHz, $CDCl_3$) δ 51.9 (CH_2), 55.0 (CH), 113.1, 117.6, 126.4, 127.5, 128.7, 129.3, 144.2 (Cq), 148.1 (Cq); IR/cm^{-1}: 3366 w, 3026 m, 2836 w, 1600 s, 1504 m, 1452 m, 1430 m, 1317 m, 1259 m, 1179 m, 1154 m, 1117 m, 1070 m, 1026 m, 990 m, 868 m, 746 s, 691 s; HRMS (CI) *m/z*: found: 213.1401, calcd for $C_{14}H_{17}N_2$ $[M+H]^+$: 213.1392.

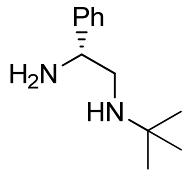

(*R*)-*N*[1]-(*tert*-butyl)-1-phenylethane-1,2-diamine, 8e. Yellow liquid; yield 84%; bp 83–84 °C (0.1 mmHg); $[\alpha]_D^{14}$ = –35.6 (c 0.78, $CHCl_3$); ^{1}H NMR (400 MHz, $CDCl_3$) δ 1.08 (s, 9H, *t*-Bu), 1.54 (br s, 3H, NH&NH_2), 2.64 (dd, *J* = 11.2, 8.6 Hz, 1H, CH_2), 2.80 (dd, *J* = 11.2, 4.6 Hz, 1H, CH_2), 3.94 (dd, *J* = 8.6, 4.6 Hz, 1H, CH), 7.24–7.29 (m, 1H, Ph), 7.32–7.37 (m, 4H, Ph); ^{13}C NMR (100 MHz, $CDCl_3$) δ 29.1 ($C(CH_3)_3$), 50.2 ($C(CH_3)_3$), 50.8 (CH_2), 56.6 (CH), 126.4, 127.1, 128.5, 145.1 (Cq); IR/cm^{-1}: 2962 m, 2865 w, 1603 m, 1493 m, 1479 m, 1453 m, 1361 m, 1231 m, 1212 m, 1097 m, 1027 m, 854 m, 761 m, 698 s; HRMS (CI) *m/z*: found:, calcd for $C_{12}H_{21}N_2$ $[M+H]^+$: 193.1705.

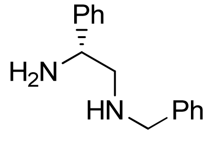

(*R*)-*N*[1]-benzyl-1-phenylethane-1,2-diamine, 8f. Yellow viscous oil; yield 98%; $[\alpha]_D^{14}$ = –29.8 (c 1.21, $CHCl_3$) [lit.[6] $[\alpha]_D^{20}$ = –6.7 (c 1.28, $CHCl_3$)]; ^{1}H NMR (400 MHz, $CDCl_3$) δ 1.65 (br s, 3H, NH&NH_2), 2.75 (dd, *J* = 11.8, 8.2 Hz, 1H, $CHCH_2$), 2.85 (dd, *J* = 11.8, 4.9 Hz, 1H, $CHCH_2$), 3.80 (d, *J* = 1.4 Hz, 2H, $NHCH_2$), 4.05 (dd, *J* = 8.2, 4.9 Hz, 1H, CH), 7.21–7.35 (m, 10H, Ph); ^{13}C NMR (100 MHz, $CDCl_3$) δ 53.9 (CH_2), 55.7 (CH), 57.2 (CH_2), 126.4, 126.9, 127.2, 128.1, 128.4, 128.5, 140.4 (Cq), 144.7 (Cq); IR/cm^{-1}: 3324 w, 3242 m, 3164 m, 3058 w, 3029 w, 2887 w, 2831 m, 1619 m, 1491 m, 1452 m, 1393 w, 1331 m, 1196 m, 1110 m, 1077 m, 1029 m, 983 m, 913 m, 844 m, 795 m, 762 m, 749 s, 697 s; HRMS (CI) *m/z*: found: 244.1806, calcd for $C_{15}H_{22}N_3$ $[M+NH_4]^+$: 244.1814.

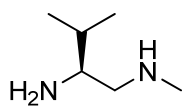

(*S*)-*N*[1],3-dimethylbutane-1,2-diamine, 8g. Clear liquid; yield 73%; bp 72–74 °C (30 mmHg); $[\alpha]_D^{20}$ = +32.8 (c 0.56, $CHCl_3$); ^{1}H NMR (400 MHz, $CDCl_3$) δ 0.91 (pseudo t, *J* = 7.5, 7.0 Hz, 6H, 2CH_3), 1.27 (br s, 3H, NH&NH_2), 1.54–1.63 (m, 1H, $CH(CH_3)_2$), 2.31–2.38 (m,

1H), 2.44 (s, 3H, NCH_3), 2.57–2.65 (m, 2H); ^{13}C NMR (100 MHz, $CDCl_3$) δ 17.7 (CH_3), 19.3 (CH_3), 32.3 ($\mathit{C}H(CH_3)_2$), 36.6 ($NHCH_3$), 56.3 ($CHNH_2$), 56.4 (NCH_2); IR (film) cm^{-1}: 3298 s, 2957 s, 2877 s, 2789 s, 1591 m, 1466 s, 1371 m, 1130 s, 1066 m, 847 s, 593 m; HRMS (ESI) *m/z*: found: 117.1386, calcd for $C_6H_{17}N_2$ $[M+H]^+$: 117.1392.

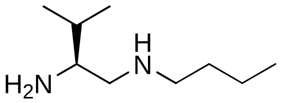

(*S*)-*N*^{1}-*n*-butyl-3-methylbutane-1,2-diamine, 8h. Clear liquid; yield 77%; bp 89–90 °C (5 mmHg); $[\alpha]_D^{20}$ = +34.4 (c 0.54, $CHCl_3$); ^{1}H NMR (400 MHz, $CDCl_3$) δ 0.89–0.94 (m, 9H, $3CH_3$), 1.14 (br s, 3H, NH&NH_2), 1.31–1.40 (m, 2H, CH_2), 1.44–1.51 (m, 2H, CH_2), 1.54–1.62 (m, 1H, $C\mathit{H}(CH_3)_2$), 2.35 (dd, *J* = 11.5, 9.4 Hz, 1H), 2.53–2.70 (m, 4H); ^{13}C NMR (100 MHz, $CDCl_3$) δ 14.0 (CH_3), 17.7 (CH_3), 19.3 (CH_3), 20.5 (CH_2), 32.40 (CH_2), 32.44 ($\mathit{C}H(CH_3)_2$), 49.9 (NCH_2), 54.3 (NCH_2), 56.6 ($CHNH_2$); IR (film) cm^{-1}: 3300 w, 2957 s, 2927 s, 2870 s, 2814 s, 1665 m, 1588 m, 1463 s, 1375 s, 1306 w, 1126 s, 1069 m, 844 s, 763 s, 592 m, 559 m; HRMS (ESI) *m/z*: found: 159.1856, calcd for $C_9H_{23}N_2$ $[M+H]^+$: 159.1861.

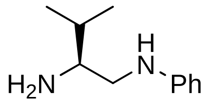

(*S*)-*N*^{1}-phenyl-3-methylbutane-1,2-diamine, 8i. Clear liquid; yield 87%; bp 114–115 °C (0.15 mmHg); $[\alpha]_D^{20}$ = +32.1 (c 1.06, $CHCl_3$) [lit.[5] $[\alpha]_D^{20}$ = +16.3 (c 1.0, $CHCl_3$)]; ^{1}H NMR (400 MHz, $CDCl_3$) δ 0.95 (d, *J* = 6.7 Hz, 3H, CH_3), 0.97 (d, *J* = 6.9 Hz, 3H, CH_3), 1.13 (br s, 2H, NH_2), 1.60–1.73 (m, 1H, $C\mathit{H}(CH_3)_2$), 2.71–2.76 (m, 1H), 2.80–2.86 (m, 1H), 3.23 (ddd, *J* = 11.7, 5.9, 3.4 Hz, 1H), 4.16 (br s, 1H, NH), 6.60–6.66 (m, 2H), 6.67–6.71 (m, 1H), 7.14–7.19 (m, 2H); ^{13}C NMR (100 MHz, $CDCl_3$) δ 17.8 (CH_3), 19.3 (CH_3), 32.5 ($\mathit{C}H(CH_3)_2$), 48.0 (NCH_2), 56.1 (NCH), 112.9, 117.2, 129.2, 148.6 (Cq); IR (film) cm^{-1}: 3377 w, 3052 w, 2957 w, 2871 w, 1602 s, 1504 s, 1466 m, 1320 m, 865 m, 746 s, 691 s; HRMS (ESI) *m/z*: found: 179.1546, calcd for $C_{11}H_{19}N_2$ $[M+H]^+$: 179.1548.

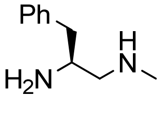

(*S*)-*N*^{1}-methyl-3-phenylpropane-1,2-diamine, 8j. Clear liquid; yield 98%; $[\alpha]_D^{20}$ = –6.2 (c 1.12, $CHCl_3$); ^{1}H NMR (400 MHz, $CDCl_3$) δ 1.46 (br s, 3H, NH&NH_2), 2.44 (s, 3H, Me), 2.43–2.53 (m, 2H), 2.66 (dd, *J* = 11.7, 3.9 Hz, 1H), 2.77–2.83 (m, 1H), 3.08–3.14 (m, 1H, $\mathit{CH}NH_2$), 7.19–7.24 (m, 3H, Ph), 7.29–7.32 (m, 2H, Ph); ^{13}C NMR (100 MHz, $CDCl_3$) δ 36.6 (CH_3), 42.9 (NCH_2), 52.3 (CH), 58.4 ($\mathit{C}H_2Ph$), 126.2, 128.4, 129.2, 139.2 (Cq); IR (film) cm^{-1}: 3287 w, 3026 w, 2929 w, 2844 w, 2787 w, 1601 w, 1494 m, 1453 m, 1360 w, 815 m, 742 s, 699 s; HRMS (ESI) *m/z*: found: 165.1392, calcd for $C_{10}H_{17}N_2$ $[M+H]^+$: 165.1392.

Preparation of chiral *bis*-imidazoline hydrochlorides 10

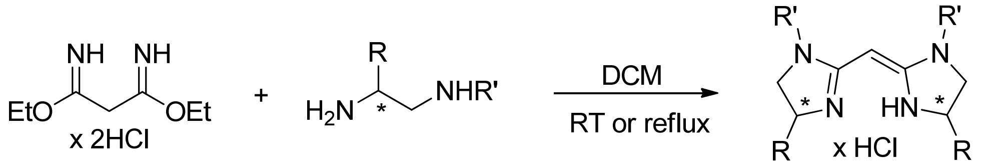

9 **8a-j** **10a-j**

General procedure: A mixture of diiminoester dihydrochloride **9** (1 mol equiv.) and the corresponding diamine **8** (2.1 mol equiv.) in dry CH_2Cl_2 (5 mL per 1 mmol of **9**) was stirred for 24–120 h at r.t. or refluxed (monitored by ^{1}H NMR) under an atmosphere of dry Ar or N_2. Once completed, the reaction mixture was diluted with CH_2Cl_2 (20 mL) and the organic extract washed with brine (20 mL). The organic phase was separated, and the aqueous phase was extracted with CH_2Cl_2 (20 mL). The combined organic phases were washed with brine (10 mL), dried over $MgSO_4$ and concentrated to dryness under reduced pressure to afford pure salts **10** as white or pale-yellow solids. In some cases further recrystallization of the prepared material from suitable solvents or solvents mixtures was required.

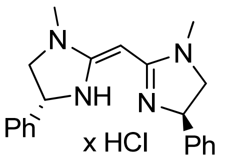

(4*R*)-1-Methyl-2-{(*E*)-[(4*R*)-1-methyl-4-phenylimidazolidin-2-ylidene]methyl}-4-phenyl-4,5-dihydro-1*H*-imidazole hydrochloride, 10a. Pale-yellow powder; yield 76%; mp 245–248 °C (MeCN); $[\alpha]_D^{20}$ = –532.7 (c 1.04, $CHCl_3$); ^{1}H NMR (400 MHz, $CDCl_3$) δ 2.90 (s, 6H, $2CH_3$), 3.43 (dd, J = 9.4, 6.2 Hz, 2H, CH_2N), 3.80 (s, 1H, =CH), 3.92 (t, J = 9.4 Hz, 2H, CH_2N), 5.13 (m, 2H, 2CHN), 7.24–7.24 (m, 2H, Ph), 7.31–7.39 (m, 8H, Ph), 9.67 (br s, 2H, NH & HCl); ^{13}C NMR (100 MHz, $CDCl_3$) δ 33.0 (CH_3), 55.2 (CH), 57.4 (=CH), 58.6 (CH_2), 126.2, 127.9, 128.7, 140.5 (Cq), 161.2 (C=N, *C*=CH); IR/cm^{-1}: 3125 m, 3026 m, 2863 m, 1571 s, 1521 s, 1492 s, 1463 m, 1322 m, 1278 s, 1245 s, 1092 m, 1072 m, 1026 m, 961 m, 756 m, 697 s, 656 m; HRMS (ESI) *m/z*: found: 333.2068, calcd for $C_{21}H_{25}N_4$ $[M–Cl]^+$: 333.2079.

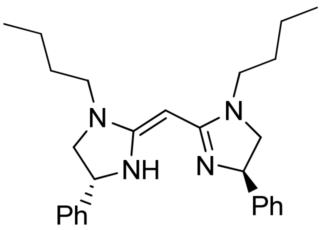

(4*R*)-1-Butyl-2-{(*E*)-[(4*R*)-1-butyl-4-phenylimidazolidin-2-ylidene]methyl}-4-phenyl-4,5-dihydro-1*H*-imidazole hydrochloride, 10b. Pale-yellow powder; yield 73%; mp 104–106 °C; $[\alpha]_D^{20}$ = –390.6 (c 1.07, $CHCl_3$); ^{1}H NMR (400 MHz, $CDCl_3$) δ 0.96 (t, J = 7.3 Hz, 6H, $2CH_3$), 1.31–1.41 (m, 4H, $2CH_2$), 1.55–1.62 (m, 4H, $2CH_2$), 3.14–3.27 (m, 4H, $2CH_2$), 3.43 (dd, J = 9.6, 6.4 Hz, 2H, CH_2N), 3.85 (s, 1H, =CH), 3.93 (t, J = 9.6 Hz, 2H, CH_2N), 5.13 (dd, J = 9.6, 6.4 Hz, 2H, 2CHN), 7.24–7.28 (m, 2H, Ph), 7.31–7.38 (m, 8H, Ph), 9.58 (br s, 2H,

NH&HCl); ^{13}C NMR (100 MHz, $CDCl_3$) δ 13.7 (CH_3), 20.0 (CH_2), 29.1 (CH_2), 45.8 (CH_2), 55.0 (CH), 56.5 (CH_2), 57.3 (=CH), 126.2, 127.8, 128.7, 140.7 (Cq), 160.5 (C=N, *C*=CH); IR ($CHCl_3$) cm^{-1}: 3119 m, 2957 m, 2925 m, 2871 m, 1561 s, 1511 s, 1492 s, 1448 s, 1322 m, 1283 m, 1238 s, 1094 m, 1075m, 1004 m, 941 m, 744 m, 694 s, 673 s; HRMS (ESI) *m/z*: found: 417.2999, calcd for $C_{27}H_{37}N_4$ $[M–Cl]^+$: 417.3018.

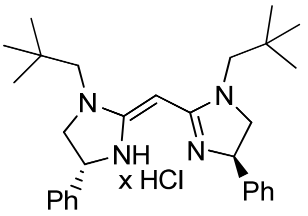

(4*R*)-1-Neopentyl-2-{(*E*)-[(4*R*)-1-neopentyl-4-phenylimidazolidin-2-ylidene]methyl}-4-phenyl-4,5-dihydro-1*H*-imidazole hydrochloride, 10c. Beige powder; yield 82%; mp 191–193 °C; $[\alpha]_D^{20}$ = –269.2 (c 0.91, $CHCl_3$); ^{1}H NMR (400 MHz, $CDCl_3$) δ 0.98 (s, 18H, 2*t*-Bu), 2.91 (d, *J* = 14.8 Hz, 2H, C*H*$_2$*t*-Bu), 3.06 (d, *J* = 14.8 Hz, 2H, C*H*$_2$*t*-Bu), 3.55 (dd, *J* = 9.7, 5.7 Hz, 2H, CH_2N), 3.96 (t, *J* = 9.7 Hz, 2H, CH_2N), 4.00 (s, 1H, =CH), 5.14 (dd, *J* = 9.7, 5.7 Hz, 2H, 2CHN) ,7.23–7.40 (m, 10H, Ph), 9.61 (br s, 2H, NH&HCl); ^{13}C NMR (100 MHz, $CDCl_3$) δ 28.4 (C(*C*H_3)$_3$), 34.2 (*C*(CH_3)$_3$), 56.7 (CH), 57.5 (=CH), 59.8 (CH_2), 60.1 (CH_2), 126.1, 128.0, 128.7, 140.6 (Cq), 162.7 (C=N, *C*=CH); IR ($CHCl_3$) cm^{-1}: 3392 w, 2956 w, 1588 s, 1528 m, 1503 m, 1456 w, 1360 w, 1277 m, 1052 m, 1033 s, 998 s, 765 m, 702 s, 697 s, 640 m; HRMS (ESI) *m/z*: found: 445.3325, calcd for $C_{29}H_{41}N_4$ $[M–Cl]^+$: 445.3331.

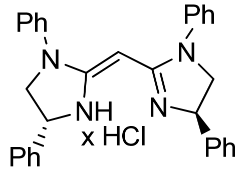

(4*R*)-2-{(*E*)-[(4*R*)-1,4-Diphenylimidazolidin-2-ylidene]methyl}-1,4-diphenyl-4,5-dihydro-1*H*-imidazole hydrochloride, 10d. Beige flaky solid; yield 65%; mp 129–131 °C; $[\alpha]_D^{14}$ = –183.9 (c 0.73, $CHCl_3$); ^{1}H NMR (400 MHz, $CDCl_3$) δ 3.80 (dd, *J* = 9.5, 6.1 Hz, 2H, CH_2N), 4.00 (s, 1H, =CH), 4.31 (t, *J* = 9.5 Hz, 2H, CH_2N), 5.31 (dd, *J* = 9.5, 6.0 Hz, 2H, 2CHN), 7.31–7.25 (m, 6H, Ph), 7.09 (d, *J* = 7.5 Hz, 4H, Ph), 7.18 (t, *J* = 7.4 Hz, 2H, Ph), 7.38 (t, *J* = 7.5 Hz, 4H, Ph), 7.46 (d, *J* = 7.4 Hz, 4H, Ph), 10.39 (br s, 2H, NH&HCl); ^{13}C NMR (100 MHz, $CDCl_3$) δ 57.9 (CH), 58.5 (=CH), 58.8 (CH_2), 125.4, 126.3, 127.2, 128.2, 128.9, 129.4, 138.9 (Cq), 140.2 (Cq), 159.6 (C=N, *C*=CH); IR/cm^{-1}: 3035 m, 1599 m, 1549 s, 1494 s, 1451 m, 1416 m, 1335 m, 1271 m, 1232 m, 1207 m, 1135 m, 1073 m, 1030 m, 1000 m, 966 m, 914 m, 839 m, 753 m, 694 s; HRMS (ESI) *m/z*: found: 457.2412, calcd for $C_{31}H_{29}N_4$ $[M–Cl]^+$: 457.2392.

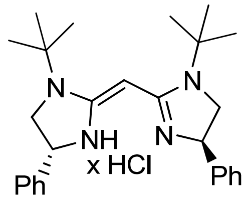

(4*R*)-1-(*tert*-Butyl)-2-{(*E*)-[(4*R*)-1-(*tert*-butyl)-4-phenylimidazolidin-2-ylidene]methyl}-4-phenyl-4,5-dihydro-1*H*-imidazole hydrochloride, 10e. Beige flaky solid; yield 72%; mp 216 °C; $[\alpha]_D^{14}$ = –407.9 (c 0.36, $CHCl_3$); ^{1}H NMR (400 MHz, $CDCl_3$) δ 1.49 (s, 18H, 2*t*-Bu), 3.51 (dd, *J* = 9.5, 7.1 Hz, 2H, CH_2N), 4.02 (t, *J* = 9.5

Hz, 2H, CH_2N), 4.54 (s, 1H, =CH), 5.03–5.08 (m, 2H, 2CHN), 7.25–7.43 (m, 10H, Ph), 8.86 (br s, 2H, NH&HCl); ^{13}C NMR (100 MHz, $CDCl_3$) δ 29.1 (C(CH$_3$)$_3$), 54.6 (C(CH$_3$)$_3$), 54.9 (CH_2), 56.6 (CH), 63.1 (=CH), 126.5, 128.0, 128.8 (CH), 140.6 (Cq), 160.9 (C=N, *C*=CH); IR/cm^{-1}: 3193 m, 3134 m, 2980 m, 1535 s, 1479 m, 1455 m, 1420 m, 1398 m, 1367m, 1303 m, 1265 m, 1209 s, 1135 m, 1096 m, 1078 m, 1004 m, 976 m, 843 w, 761 m, 727 m, 697 s; HRMS (ESI) *m/z*: found: 417.3002, calcd for $C_{27}H_{37}N_4$ $[M–Cl]^+$: 417.3018.

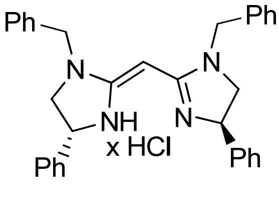

(4*R*)-1-Benzyl-2-{(*E*)-[(4*R*)-1-benzyl-4-phenylimidazolidin-2-ylidene]methyl}-4-phenyl-4,5-dihydro-1*H*-imidazole hydrochloride, 10f. Beige crystals; yield 76%; mp 177–178°C (acetone/ hexane); $[\alpha]_D^{14}$ = –281.7 (c 0.60, $CHCl_3$); ^1H NMR (400 MHz, $CDCl_3$) δ 3.44 (dd, *J* = 9.6, 6.5 Hz, 2H, CH_2N), 3.93 (t, *J* = 9.7 Hz, 2H, CH_2N), 4.02 (s, 1H, =CH), 4.27–4.35 (m, 4H, 2CH_2Ph), 5.17–5.21 (m, 2H, 2CHN), 7.09–7.10 (m, 4H, Ph), 7.25–7.39 (m, 16H, Ph), 9.90 (br s, 2H, NH&HCl); ^{13}C NMR (100 MHz, $CDCl_3$) δ 50.1 (CH_2), 55.9, 56.9 (CH_2), 57.6 (=CH), 126.3, 127.0, 128.0, 128.8, 129.0, 135.1 (Cq), 140.4 (Cq), 160.7 (C=N, *C*=CH); IR/cm^{-1}: 3088 m, 3000 m, 2850 m, 1589 m, 1567 s, 1513 s, 1494 s, 1452 m, 1424 m, 1352 m, 1339 m, 1299 w, 1266 m, 1239 s, 1172 m, 1125 m, 1093 m, 1071 m, 1028 m, 954 m, 938 m, 759 m, 732 s, 696 s; HRMS (ESI) *m/z*: found: 485.2693, calcd for $C_{33}H_{33}N_4$ $[M–Cl]^+$: 485.2705.

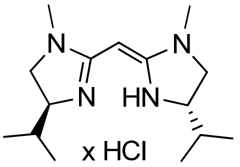

(*S*)-4-Isopropyl-2-{(*E*)-[(*S*)-4-isopropyl-1-methylimidazolidin-2-ylidene]methyl}-1-methyl-4,5-dihydro-1H-imidazole hydrochloride, 10g. White solid; yield 100%; mp 239–241 °C; $[\alpha]_D^{20}$ = +51.0 (c 0.52, $CHCl_3$); ^1H NMR (400 MHz, $CDCl_3$) δ 0.90 (d, *J* = 6.7 Hz, 6H, 2CH_3), 0.96 (d, *J* = 6.7 Hz, 6H, 2CH_3), 2.15–2.27 (m, 2H, 2CH(CH$_3$)$_2$), 2.84 (s, 6H, 2NCH_3), 3.25 (dd, *J* = 9.6, 6.8 Hz, 2H, CH_2), 3.48 (t, *J* = 9.6 Hz, 2H, CH_2), 3.54 (s, 1H, =CH), 3.92–3.98 (m, 2H, 2CHN), 8.93 (br s, 2H, NH&HCl); ^{13}C NMR (100 MHz, $CDCl_3$) δ 16.0 (CH_3), 18.6 (CH_3), 31.3 (CH(CH$_3$)$_2$), 32.8 (NCH_3), 52.0 (NCH_2), 54.3 (=CH), 59.7 (CHN), 160.9 (C=N, *C*=CH); IR ($CHCl_3$) cm^{-1}: 3219 w, 2961 m, 1576 s, 1525 m, 1465 w, 1325 m, 1284 m, 1248 m, 1068 m, 1027 m, 982 w, 926 w; HRMS (ESI) *m/z*: found: 265.2387, calcd for $C_{15}H_{29}N_4$ $[M–Cl]^+$: 265.2392.

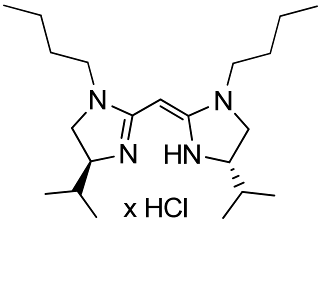

(*S*)-4-Isopropyl-2-{(*E*)-[(*S*)-4-isopropyl-1-butylimidazolidin-2-ylidene]methyl}-1-butyl-4,5-dihydro-1H-imidazole hydrochloride, 10h. White solid; yield 91%; mp 103–105 °C; $[\alpha]_D^{20}$ = +32.7 (c 0.51, $CHCl_3$); ^1H

NMR (400 MHz, $CDCl_3$) δ 0.89 (d, J = 6.8 Hz, 6H, 2CHCH_3), 0.95–0.98 (m, 12H, 4CH_3), 1.30–1.39 (m, 4H, 2CH_2), 1.52–1.60 (m, 4H, 2CH_2), 2.17–2.25 (m, 2H, 2CH(CH_3)$_2$), 3.10–3.15 (m, 4H, 2CH_2), 3.25 (dd, J = 9.6, 6.7 Hz, 2H, CH_2CH), 3.48 (t, J = 9.6 Hz, 2H, CH_2CH), 3.60 (s, 1H, =CH), 3.92–3.98 (m, 2H, 2CHN), 8.81 (br s, 2H, NH&HCl); ^{13}C NMR (100 MHz, $CDCl_3$) δ 13.7 (CH_3), 16.0 (CH_3), 18.6 (CH_3), 20.2 (CH_2), 29.2 (CH_2), 31.5 (CH(CH_3)$_2$), 45.9 (NCH_2), 49.9 (NCH_2), 54.2 (=CH), 59.6 (CHN), 160.3 (C=N, C=CH); IR ($CHCl_3$) cm^{-1}: 3219 w, 2962 m, 2873 m, 1571 s, 1518 m, 1463 w, 1332 w, 1281 m, 1242 m, 1083 w, 932 w, 631 m; HRMS (ESI) *m/z*: found: 349.3326, calcd for $C_{21}H_{41}N_4$ [M–Cl]$^+$: 349.3331.

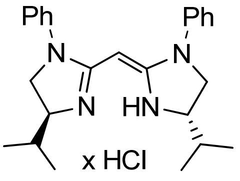

(*S*)-4-Isopropyl-2-{(*E*)-[(*S*)-4-isopropyl-1-phenylimidazolidin-2-ylidene]methyl}-1-phenyl-4,5-dihydro-1H-imidazole, 10i. Pale-yellow crystals; yield 75%; mp 76.5–79 °C; $[\alpha]_D^{20}$ = +55.9 (c 1.11, $CHCl_3$); ^{1}H NMR (400 MHz, $CDCl_3$) δ 1.00 (d, J = 6.8 Hz, 6H, 2CH_3), 1.01 (d, J = 6.8 Hz, 6H, 2CH_3), 2.29–2.37 (m, 2H, 2CH(CH_3)$_2$), 3.61 (dd, J = 9.6, 6.1 Hz, 2H, CH_2), 3.76 (s, 1H, =CH), 3.88 (t, J = 9.6 Hz, 2H, CH_2), 4.10–4.15 (m, 2H, 2CHN), 7.04–7.06 (m, 4H, Ph), 7.14–7.17 (m, 2H, Ph), 7.24–7.27 (m, 4H, Ph), 9.58 (br s, 2H, NH&HCl); ^{13}C NMR (100 MHz, $CDCl_3$) δ 16.0 (CH_3), 18.6 (CH_3), 31.5 (CH(CH_3)$_2$), 52.2 (NCH_2), 57.8 (=CH), 60.0 (CHN), 125.3, 126.8, 129.3, 139.1 (Cq), 159.2 (C=N, C=CH); IR ($CHCl_3$) cm^{-1}: 3400 w, 3200 w, 2958 m, 2871 m, 1599 s, 1547 s, 1496 s, 1460 m, 1276 m, 1231 m, 1131 m, 763 m, 696 s; HRMS (ESI) *m/z*: found: 389.2691, calcd for $C_{25}H_{33}N_4$ [M–Cl]$^+$: 389.2705.

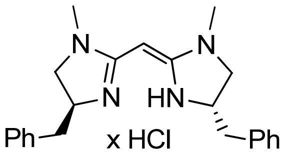

(4*S*)-4-Benzyl-2-{(*E*)-[(4*S*)-4-benzyl-1-methylimidazolidin-2-ylidene]methyl}-1-methyl-4,5-dihydro-1*H*-imidazole hydrochloride, 10j. Pale-yellow powder; yield 71%; mp 207–209 °C; $[\alpha]_D^{20}$ = +202.4 (c 0.83, $CHCl_3$); ^{1}H NMR (400 MHz, $CDCl_3$) δ 2.74 (s, 6H, 2CH_3), 2.85 (dd, J = 13.7, 9.5 Hz, 2H, CH_2Ph), 3.21 (dd, J = 9.5, 6.8 Hz, 2H, NCH_2), 3.39 (t, J = 9.5 Hz, 2H, NCH_2), 3.48 (dd, J = 13.7, 3.6 Hz, 2H, CH_2Ph), 3.51 (s, 1H, =CH), 4.31–4.38 (m, 2H, 2CHN), 7.20–7.31 (m, 10H, Ph), 9.32 (br s, 2H, NH&HCl); ^{13}C NMR (100 MHz, $CDCl_3$) δ 32.8 (CH_3), 40.2 (CH_2), 54.5 (CH_2), 54.8 (CH), 55.7 (=CH), 126.6, 128.4, 129.4, 136.8 (Cq), 160.5 (C=N, C=CH); IR ($CHCl_3$) cm^{-1}: 3422 w, 3225 w, 2918 w, 1572 s, 1523 s, 1498 s, 1455 m, 1320 m, 1201 m, 1023 m, 761 m, 734 m, 702 s, 642 m; HRMS (ESI) *m/z*: found: 361.2382, calcd for $C_{23}H_{29}N_4$ [M–Cl]$^+$: 361.2392.

Synthesis of catalysts 5

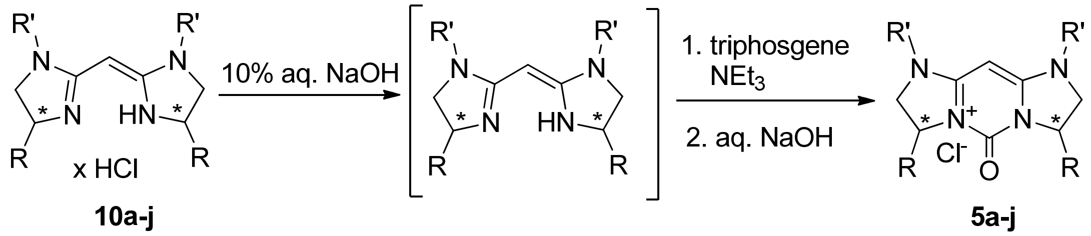

General procedure: A mixture of the requisite salt **10** (1 mol equiv.) and 10% aq. NaOH (15 mol equiv.) in CH_2Cl_2 (15 mL per 1 mmol of **10**) was vigorously shaken in a separatory funnel for 5 min. The organic phase was separated; the aqueous phase was extracted with CH_2Cl_2 (30 mL). The combined organic phases were washed with water (20 mL), dried over $MgSO_4$ and concentrated to dryness under reduced pressure to provide the corresponding free bases quantitatively as white solids. The corresponding free MBI base was then dissolved in dry CH_2Cl_2 (10 mL per 1 mmol) followed by the addition of NEt_3 (3.0 mol equiv.) in one portion. The resulting solution was cooled down to 0 °C under the atmosphere of dry N_2 followed by dropwise addition of a solution of triphosgene (0.66 mol equiv.) in dry CH_2Cl_2 (15 mL per 1 mmol of triphosgene). The reaction mixture was stirred for 0.5 h at 0 °C, further 2 h at r.t., and then quenched with 10% aq. NaOH (7 mol equiv.). After 10 min of vigorous stirring at r.t., the solvent was evaporated to dryness *in vacuo* (12 mbar), the solid residue was dissolved in CH_2Cl_2 (2×20 mL) and the resulting solution was washed with brine (10 mL), dried over $MgSO_4$ and concentrated *in vacuo* to afford crude tricycles **5** as pale yellow or orange solids which were further purified by recrystallization from suitable solvents or solvents mixtures.

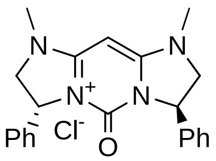

(4*R*,12*R*)-6,10-Dimethyl-2-oxo-4,12-diphenyl-1λ^5,3,6,10-tetraazatricyclo[7.3.0.0^{3,7}]dodeca-1(9),7-dien-1-ylium chloride, 5a. Yellow hygroscopic powder; yield 86%; mp 104–106 °C (dec.) (acetone/ hexane); $[\alpha]_D^{14}$ = –261.8 (c 0.45, $CHCl_3$); ^{1}H NMR (400 MHz, $CDCl_3$) δ 3.51 (s, 6H, 2Me), 3.71 (dd, J = 10.4, 4.1 Hz, 2H, CH_2N), 4.34 (t, J = 10.4 Hz, 2H, CH_2N), 5.37 (dd, J = 10.4, 4.1 Hz, 2H, 2CHN), 7.21–7.24 (m, 3H, Ph&=CH), 7.33–7.41 (m, 6H, Ph); ^{13}C NMR (100 MHz, $CDCl_3$) δ 34.2 (Me), 58.5 (CH), 59.0 (CH_2), 65.0 (=CH), 126.1, 129.4, 129.5, 137.6 (Cq), 143.8 (C=O), 158.0 (Cq); IR/cm^{-1}: 3368 m, 2934 w, 1717 m, 1624 s, 1518 m, 1494 m, 1452 m, 1425 m, 1323 w, 1288 m, 1233 m, 1074 m, 1031 m, 855 w, 747 m, 699 s; HRMS (ESI) *m/z*: found: 359.1862, calcd for $C_{22}H_{23}N_4O$ $[M-Cl]^+$: 359.1872.

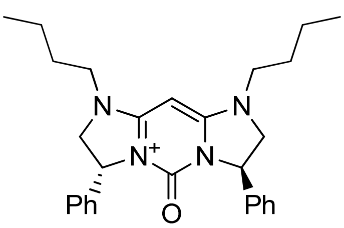

(4*R*,12*R*)-6,10-Dibutyl-2-oxo-4,12-diphenyl-1λ^5,3,6,10-tetraazatricyclo[7.3.0.0^{3,7}]dodeca-1(9),7-dien-1-ylium chloride, 5b.

White crystals; yield 86%; mp 291–293 °C (dec.) (chloroform); $[\alpha]_D^{14}$ = –281.2 (c 0.87, MeOH); ^{1}H NMR (400 MHz, CD_3CN) δ 0.96 (t, J = 7.3 Hz, 6H, $2CH_2CH_3$), 1.40 (sextet, J = 7.3 Hz, 4H, $2CH_2CH_3$), 1.64 (pentet, J = 7.3 Hz, 4H, $2CH_2CH_2CH_2$), 3.50 (td, J = 7.3, 3.7 Hz, 4H, $2NCH_2CH_2$), 3.65 (dd, J = 10.5, 4.2 Hz, 2H, CH_2N), 4.27 (t, J = 10.5 Hz, 2H, CH_2N), 5.39 (dd, J = 10.5, 4.2 Hz, 2H, 2CHN), 5.59 (s, 1H, =CH), 7.28–7.30 (m, 4H, Ph), 7.34–7.42 (m, 6H, Ph); ^{13}C NMR (100 MHz, CD_3CN) δ 13.0 (CH_3), 19.5 (CH_2), 28.4 (CH_2), 45.6 (CH_2), 56.8 (CH_2), 58.1 (CH), 62.0 (=CH), 126.4, 128.8, 129.0, 139.0 (Cq&C=O), 157.8 (Cq); IR/cm^{-1}: 3352 w, 3056 m, 2960 m, 2912 m, 2873 m, 1714 s, 1616 s, 1509 m, 1454 m, 1355 m, 1389 m, 1284 m, 1254 m, 1239 m, 1094 m, 933 m, 738 m, 697 m, 656 m; HRMS (ESI) *m/z*: found: 443.2811, calcd for $C_{28}H_{35}N_4O$ $[M-Cl]^+$: 443.2811. The crystal suitable for the X-Ray analysis was grown from CH_3CN at r.t.

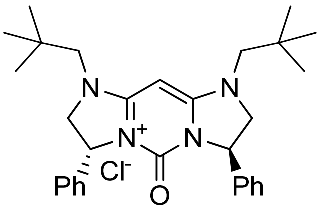

(4*R*,12*R*)-6,10-Dineopentyl-2-oxo-4,12-diphenyl-1λ^5,3,6,10-tetraazatricyclo[7.3.0.0^{3,7}]dodeca-1(9),7-dien-1-ylium chloride, 5c. Beige powder; yield 92%; mp 208–209 °C (dec.) (ethyl acetate); $[\alpha]_D^{14}$ = –186.7 (c 0.50, $CHCl_3$); ^{1}H NMR (400 MHz, $CDCl_3$) δ 1.04 (s, 18H, 2*t*-Bu), 3.65 (d, J = 14.7 Hz, 2H, C*H*$_2$*t*-Bu), 3.73 (d, J = 14.7 Hz, 2H, C*H*$_2$*t*-Bu), 3.74 (dd, J = 10.3, 4.3 Hz, 2H, CH_2N), 4.36 (t, J = 10.3 Hz, 2H, CH_2N), 5.31 (dd, J = 10.3, 4.3 Hz, 2H, 2CHN), 6.77 (s, 1H, =CH), 7.19–7.21 (m, 4H, Ph), 7.27–7.35 (m, 6H, Ph); ^{13}C NMR (100 MHz, $CDCl_3$) δ 28.1 ($C(CH_3)_3$), 34.7 ($C(CH_3)_3$), 58.6 (CH), 59.0 (CH_2), 60.3 (CH_2), 66.4 (=CH), 126.0, 129.4, 129.5, 137.6 (Cq), 143.9 (C=O), 159.1 (Cq); IR/cm^{-1}: 3353 w, 2954 m, 2870 m, 1721 m, 1611 s, 1510 m, 1479 m, 1454 m, 1398 m, 1365 m, 1277 m, 1253 m, 1225 m, 1101 m, 1132 m, 901 m, 857 m, 742 m, 698 m, 656 m; HRMS (ESI) *m/z*: found: 471.3118, calcd for $C_{30}H_{39}N_4O$ $[M-Cl]^+$: 471.3124.

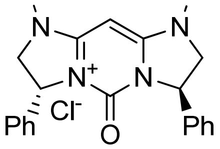

(4*R*,12*R*)-2-Oxo-4,6,10,12-tetraphenyl-1λ^5,3,6,10-tetraazatricyclo[7.3.0.0^{3,7}] dodeca-1(9),7-dien-1-ylium chloride, 5d. Yellow hygroscopic crystals; yield 84%; mp 211–213 °C (dec.) (CH_2Cl_2/ pentane); $[\alpha]_D^{14}$ = –109.8 (c 0.89, $CHCl_3$); ^{1}H NMR (400 MHz, $CDCl_3$) δ 3.94 (dd, J = 10.2, 4.1 Hz, 2H, CH_2N), 5.13 (t, J = 10.2 Hz, 2H, CH_2N), 5.18 (s, 1H, =CH), 5.68 (dd, J = 10.2, 4.1 Hz, 2H, 2CHN), 7.27–7.56 (m, 20H, Ph); ^{13}C NMR (100 MHz, $CDCl_3$) δ 58.7 (CH), 60.6 (CH_2), 64.9 (=CH), 124.7, 126.4, 128.9, 129.1, 129.4, 130.4, 136.1 (Cq), 137.5 (Cq), 143.9 (C=O), 157.4 (Cq); IR/cm^{-1}: 3338 m, 3046 m, 1726 m, 1614 s, 1571 s, 1514 m, 1495 m, 1454 m, 1285 m, 1230 m, 1208 m, 1132 m, 1083 m, 1028 m, 1002 m, 943 m, 750 m, 697 s; HRMS (ESI) *m/z*: found: 483.2182, calcd for $C_{32}H_{27}N_4O$ $[M-Cl]^+$: 483.2185.

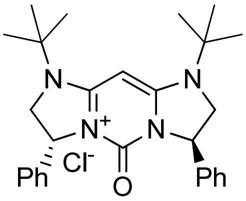

(4*R*,12*R*)-6,10-Di(*tert*-butyl)-2-oxo-4,12-diphenyl-1λ^5,3,6,10-tetraazatricyclo[7.3.0.0^{3,7}]dodeca-1(9),7-dien-1-ylium chloride, 5e. White crystals; yield 88%; mp 260–260.5 °C (dec.) (ethyl acetate); $[\alpha]_D^{14}$ = –264.4 (c 0.64, $CHCl_3$); ^{1}H NMR (400 MHz, $CDCl_3$) *δ* 1.62 (s, 18H, 2*t*-Bu), 3.81 (dd, *J* = 10.5, 4.0 Hz, 2H, CH_2N), 4.88 (t, *J* = 10.5 Hz, 2H, CH_2N), 5.41 (dd, *J* = 10.5, 4.0 Hz, 2H, 2CHN), 5.45 (s, 1H, =CH), 7.24–7.26 (m, 4H, Ph), 7.32–7.40 (m, 6H, Ph); ^{13}C NMR (100 MHz, $CDCl_3$) *δ* 28.4 (C(*C*$H_3)_3$), 56.2 (CH_2), 57.1 (*C*($CH_3)_3$), 57.2 (CH), 67.9 (=CH), 126.0, 129.2, 129.4, 137.9 (Cq&C=O), 155.4 (Cq); IR/cm^{-1}: 3346 w, 2981 m, 2902 m, 1718 m, 1594 s, 1504 m, 1455 m, 1404 m, 1374 m, 1278 m, 1197 m, 1080 m, 1051 m, 747 m, 699 m; HRMS (ESI) *m/z*: found: 443.2813, calcd for $C_{28}H_{35}N_4O$ $[M-Cl]^+$: 443.2811.

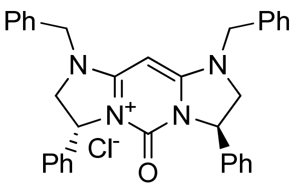

(4*R*,12*R*)-6,10-Dibenzyl-2-oxo-4,12-diphenyl-1λ^5,3,6,10-tetraazatricyclo[7.3.0.0^{3,7}]dodeca-1(9),7-dien-1-ylium chloride, 5f. White powder; yield 87%; mp 326–327.5 °C (dec.) (MeCN); $[\alpha]_D^{14}$ = –203.1 (c 0.36, MeOH); ^{1}H NMR (400 MHz, CD_3CN) *δ* 3.56 (dd, *J* = 10.6, 4.4 Hz, 2H, CH_2N), 4.19 (t, *J* = 10.6 Hz, 2H, CH_2N), 4.85 (s, 2H, C*H₂*Ph), 5.38 (dd, *J* = 10.6, 4.4 Hz, 2H, 2CHN), 6.57 (s, 1H, =CH), 7.24–7.46 (m, 20H, Ph); ^{13}C NMR (100 MHz, CD_3CN) *δ* 49.5 (CH_2), 56.6 (CH_2), 58.3(CH), 63.3 (=CH), 126.4, 128.5, 128.8, 128.9, 129.0, 134.2 (Cq), 138.8 (Cq&C=O), 158.2 (Cq); IR/cm^{-1}: 3044 m, 1713 s, 1621 s, 1511 m, 1496 m, 1455 m, 1389 m, 1350 m, 1284 m, 1243 m, 1206 m, 1112 m, 1080 m, 1030 m, 860 m, 822 m, 759 m, 748 m, 697 s; HRMS (ESI) *m/z*: found: 511.2488, calcd for $C_{34}H_{31}N_4O$ $[M-Cl]^+$: 511.2498.

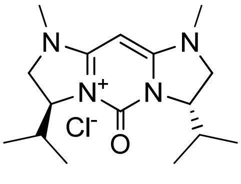

(4*S*,12*S*)-6,10-Dimethyl-2-oxo-4,12-bis(isopropyl)-1λ^5,3,6,10-tetraazatricyclo[7.3.0.0^{3,7}]dodeca-1(9),7-dien-1-ylium chloride, 5g. White powder; yield 93%; mp 296–297 °C (dec.) (MeCN); $[\alpha]_D^{20}$ = +76.1 (c 1.76, $CHCl_3$); ^{1}H NMR (400 MHz, $CDCl_3$) *δ* 0.79 (d, *J* = 7.0 Hz, 6H, 2CH*Me*), 0.96 (d, *J* = 7.0 Hz, 6H, 2CH*Me*), 2.63 (dqq, *J* = 10.4, 7.0, 3.5 Hz, 2H, 2C*H*Me_2), 3.41 (s, 6H, Me), 3.59 (dd, *J* = 10.4, 4.1 Hz, 2H, 2CHN), 3.91 (t, *J* = 10.4 Hz, 2H, CH_2N), 4.54 (dt, *J* = 10.4, 3.7 Hz, 2H, CH_2N), 6.91 (s, 1H, =CH); ^{13}C NMR (100 MHz, $CDCl_3$) *δ* 14.0 (CH*Me*), 17.9 (CH*Me*), 27.7 (*C*HMe_2), 33.9 (NMe), 50.6 (CH_2), 60.5 (CH), 63.8 (C=*C*H), 144.6 (C=O), 157.8 (Cq); IR/cm^{-1}: 2996 m, 2953 m, 2933 m, 2875 m, 1704 s, 1626 s, 1512 s, 1492 m, 1425 m, 1386 m, 1337 m, 1313 m, 1300 s, 1234 m, 1130 m, 1100 m, 1072 m, 1032 m, 986 m, 847 m, 812 m, 777 m, 754 s, 740 m, 691 m; HRMS (ESI) *m/z*: found: 291.2177, calcd for $C_{16}H_{27}N_4O$ $[M-Cl]^+$: 291.2185.

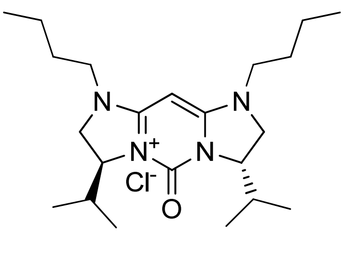

(4*S*,12*S*)-6,10-Dibutyl-2-oxo-4,12-bis(isopropyl)-1λ^5,3,6,10-tetraazatricyclo[7.3.0.0^{3,7}]dodeca-1(9),7-dien-1-ylium chloride, 5h. White hygroscopic powder; yield 87%; mp 189–190.5 °C (ethyl acetate); $[\alpha]_D^{18}$ = +99.0 (c 0.99, $CHCl_3$); ^{1}H NMR (400 MHz, $CDCl_3$) *δ* 0.80 (d, *J* = 7.0 Hz, 6H, 2C*H*$_3$CH), 0.96 (d, *J* = 7.0 Hz, 6H, 2C*H*$_3$CH), 0.96 (t, *J* = 7.3 Hz, 6H, 2C*H*$_3$CH$_2$), 1.45–1.54 (m, 4H), 1.62–1.69 (m, 4H), 2.60–2.71 (m, 2H, 2C*H*(CH_3)$_2$), 3.58 (dd, *J* = 10.8, 4.1 Hz, 2H), 3.75–3.90 (m, 6H), 4.54 (dt, *J* = 10.0 Hz, 3.8 Hz, 2NCH, 2H), 6.76 (s, 1H, =CH); ^{13}C NMR (100 MHz, $CDCl_3$) *δ* 14.0, 14.1, 18.0, 19.9 (CH_2), 27.8, 29.1 (CH_2), 46.6 (CH_2), 48.1 (CH_2), 60.3 (CH), 63.9 (C=*C*H), 144.8 (C=O), 157.4 (Cq); IR/cm^{-1}: 3374 w, 3040 w, 2958 m, 2932 m, 2872 m, 1704 s, 1617 s, 1514 s, 1460 m, 1393 m, 1370 m, 1342 m, 1292 m, 1244 m, 1214 m, 1191 m, 1094 m, 901 m, 797 m, 755 m; HRMS (ESI) *m/z*: found: 375.3120, calcd for $C_{22}H_{39}N_4O$ [M-Cl]$^+$: 375.3124; CHN: found: C 64.20, H 9.47, N 13.59%. $C_{22}H_{39}ClN_4O$ requires: C 64.29, H 9.56, N 13.63%.

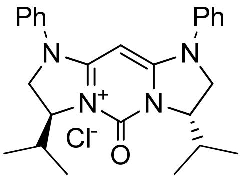

(4*S*,12*S*)-6,10-Diphenyl-2-oxo-4,12-bis(isopropyl)-1λ^5,3,6,10-tetraazatricyclo[7.3.0.0^{3,7}]dodeca-1(9),7-dien-1-ylium chloride, 5i. Yellowish powder; yield 91%; mp 233.5–235 °C (ethyl acetate); $[\alpha]_D^{20}$ = +161.5 (c 0.26, $CHCl_3$); ^{1}H NMR (400 MHz, $CDCl_3$) *δ* 1.01 (d, *J* = 6.8 Hz, 6H, 2Me), 1.02 (d, *J* = 6.8 Hz, 6H, 2Me), 2.74–2.77 (m, 2H, 2C*H*Me$_2$), 3.88–3.90 (m, 2H, 2CHN), 4.83–4.94 (m, 5H, 2CH$_2$N&=CH), 7.32–7.38 (m, 2H, Ph), 7.44–7.47 (m, 8H, Ph); ^{13}C NMR (100 MHz, $CDCl_3$) *δ* 14.6 (Me), 18.2 (Me), 28.0 (*C*HMe$_2$), 52.4 (CH_2), 60.8 (CH), 63.8 (C=*C*H), 124.4, 128.8, 130.4, 136.2 (Cq), 144.5 (C=O), 157.1 (Cq); IR/cm^{-1}: 3359 w, 2962 m, 2874 m, 1713 m, 1614 s, 1574 s, 1519 m, 1498 m, 1457 m, 1394 m, 1287 m, 1233 m, 1139 m, 1114 m, 1000 m, 943 m, 767 m, 752 m, 733 m, 691 m; HRMS (ESI) *m/z*: found: 415.2496, calcd for $C_{26}H_{31}N_4O$ [M-Cl]$^+$: 415.2498.

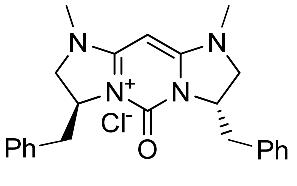

(4*S*,12*S*)-6,10-Dimethyl-2-oxo-4,12-dibenzyl-1λ^5,3,6,10-tetraazatricyclo[7.3.0.0^{3,7}]dodeca-1(9),7-dien-1-ylium chloride, 5j. Tan crystals; yield 85%; mp 65–66 °C (CH_2Cl_2/ diethyl ether); $[\alpha]_D^{20}$ = +119.0 (c 0.42, $CHCl_3$); ^{1}H NMR (400 MHz, $CDCl_3$) *δ* 2.93 (dd, *J* = 13.5, 9.4 Hz, 2H, C*H*$_2$Ph), 3.25 (s, 6H, 2Me), 3.49 (dd, *J* = 13.5, 3.0 Hz, 2H, C*H*$_2$Ph), 3.62 (dd, *J* = 10.4, 3.8 Hz, 2H, CH$_2$N), 3.87 (t, *J* = 10.4 Hz, 2H, CH$_2$N), 4.81–4.87 (m, 2H, 2CHN), 6.53 (s, 1H, =CH), 7.20–7.37 (m, 10H, Ph); ^{13}C NMR (100 MHz, $CDCl_3$) *δ* 33.8 (Me), 37.5 (*C*H$_2$Ph), 54.6 (CH_2), 56.6 (CH), 64.1 (=CH), 127.6, 129.1, 129.3, 134.5 (Cq), 144.9 (C=O), 157.4 (Cq); IR/cm^{-1}: 3364 m, 3059 m, 3028 m, 2939 m, 1707 m, 1623 s, 1520 m, 1493 m, 1454 m, 1424 m, 1334 m, 1293 m, 1235 m, 1081 m, 1033

m, 921 m, 848 m, 741 s, 703 s; HRMS (ESI) *m/z*: found: 387.2172, calcd for $C_{24}H_{27}N_4O$ $[M-Cl]^+$: 387.2185.

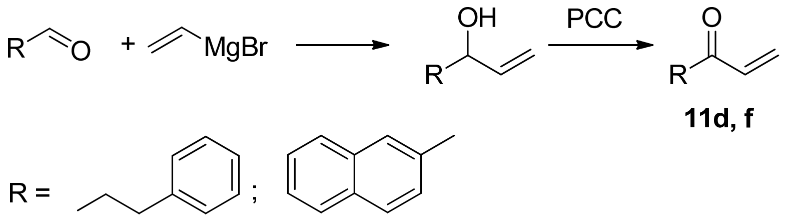

Vinyl ketones **11d, f** were prepared according to literature procedure:[7]

Grignard reaction: A solution of corresponding aldehyde (10.0 mmol, 1 equiv.) in anhydrous THF (40 mL) was stirred for 10 minutes under nitrogen atmosphere at 0 °C. A solution of vinylmagnesium bromide (20.0 mmol, 2 equiv., 1 M THF) was added dropwise, and the reaction mixture was stirred overnight. Then it was quenched with saturated aqueous NH_4Cl solution and extracted with diethyl ether. The combined organic layers were washed with brine, dried over anhydrous $MgSO_4$, filtered, and concentrated *in vacuo*. The residue was purified by silica gel column chromatography (hexane/ ethyl acetate) to afford the desired allylic alcohols.

Oxidation: PCC (12.0 mmol, 1.2 equiv.) was suspended in dry CH_2Cl_2 (50 mL), neutral aluminium oxide (5.0 g) was added followed by dropwise addition of the corresponding alcohol (10.0 mmol, 1.0 equiv.) dissolved in CH_2Cl_2 (10 mL). The reaction mixture was stirred at r.t. for 4 h (TLC control), filtered through a short pad of silica gel, concentrated and purified by column chromatography on silica gel (hexane/ ethyl acetate) to afford the desired vinyl ketones.

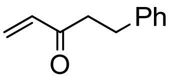

5-Phenylpent-1-ene-3-one, 11d. Clear liquid; R_f = 0.38 (hexane/ ethyl acetate = 10:1); ^1H NMR (400 MHz, $CDCl_3$) δ 2.90–2.98 (m, 4H), 5.84 (dd, J = 10.4, 1.0 Hz, 1H), 6.22 (dd, J = 17.6, 0.9 Hz, 1H), 6.36 (dd, J = 17.7, 10.6 Hz, 1H), 7.18–7.22 (m, 3H), 7.27–7.32 (m, 3H), ^{13}C NMR (100 MHz, $CDCl_3$) δ 29.8, 41.2, 126.2, 128.34, 128.38, 128.5, 136.5, 141.1, 199.8; IR/cm^{-1}: 1699 s, 1680 m, 1613 m, 1496 m, 1454 m, 1402 m, 1366 m, 1186 m, 1096 m, 1075 m, 1030 m, 986 m, 963 m, 748 m, 698 m; HRMS (CI) *m/z*: found: 178.1242, calcd for $C_{11}H_{16}NO$ $[M+NH_4]^+$: 178.1232.

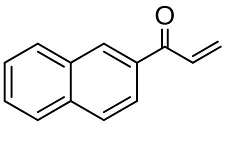

1-(Naphthalen-2-yl)-prop-2-ene-1-one, 11f. Colorless oil crystallizing on standing in a fridge; R_f = 0.46 (hexane/ ethyl acetate = 10:1); mp 38–40 °C {lit.[8] mp 39–40 °C}; ^1H NMR (400 MHz, $CDCl_3$) δ 5.99 (dd, J = 10.6, 1.8 Hz, 1H), 6.51 (dd, J = 17.1, 1.8 Hz, 1H), 7.33 (dd, J = 17.1, 10.6 Hz, 1H), 7.56–7.64 (m, 2H), 7.88–7.98 (m, 3H), 8.04 (dd, J = 8.6, 1.7 Hz, 1H), 8.47 (s, 1H); ^{13}C NMR (100 MHz, $CDCl_3$) δ 124.5, 126.9, 127.8, 128.55, 128.64, 129.6, 130.2, 130.4, 132.4, 132.5, 134.7, 135.6, 190.9; IR/cm^{-1}: 1661 s, 1625 m, 1606

m, 1597 m, 1575 m, 1508 m, 1465 m, 1437 m, 1366 m, 1348 m, 1276 m, 1253 m, 1220 m, 1191 m, 1181 m, 1122 m, 999 m, 974 m, 878 m, 864 m, 842 m, 821 m, 789 s, 768 m, 745 s, 721 m; HRMS (CI) *m/z*: found: 200.1084, calcd for $C_{13}H_{14}NO$ $[M+NH_4]^+$: 200.1075.

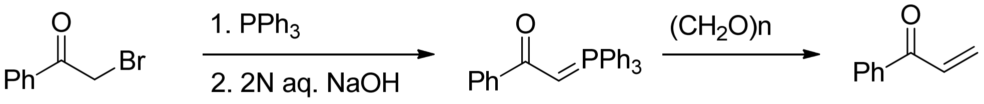

11e

1-Phenylprop-2-ene-1-one, 11e.[9] PPh_3 (1.0 equiv.) was added to a stirred solution of α-bromo acetophenone (1.0 equiv.) in CH_2Cl_2 (0.3 M). The reaction mixture was stirred at r.t. for 26 h (TLC control), concentrated under reduced pressure and the resulting precipitate was washed with Et_2O. Thus obtained crude PPh_3 salt was added to a mixture of H_2O and MeOH (v/v = 1:1; 0.25 M) and the reaction mixture was stirred at r.t. for 1 h, followed by the addition of 2N aq. NaOH to pH = 7–8 and vigorous stirring for another 3 h. After flash filtration of the suspension formed, the precipitate was washed with H_2O and dried. The dry phosphorane ylide was dissolved in freshly distilled CH_2Cl_2 (0.045 M) under N_2, followed by the addition of dry $(CH_2O)_n$ (3.0 equiv. by weight). The reaction mixture was heated at reflux under N_2 for 24 h (TLC control). On completion the reaction mixture was cooled down to r.t., flash filtered through a glass filter and concentrated under reduced pressure. The residue was purified by flash chromatography (hexane/ethyl acetate = 20:1) to afford the desired phenyl vinyl ketone **11e** as colorless viscous oil; R_f = 0.41 (hexane/ ethyl acetate = 10:1); ^{1}H NMR (400 MHz, $CDCl_3$) δ 5.94 (dd, J = 10.6, 1.7 Hz, 1H), 6.45 (dd, J = 17.2, 1.7 Hz, 1H), 7.17 (dd, J = 17.2, 10.6 Hz, 1H), 7.47–7.51 (m, 2H), 7.56–7.61 (m, 1H), 7.94–7.96 (m, 2H); ^{13}C NMR (100 MHz, $CDCl_3$) δ 128.6, 128.7, 130.3, 132.4, 133.0, 137.2, 191.1; IR/cm^{-1}: 1670 s, 1608 m, 1596 m, 1578 m, 1447 m, 1403 m, 1286 m, 1230 s, 1180 m, 1101 m, 1077 m, 1003 m, 993 m, 977 m, 964 m, 725 s, 686 s, 652 m; HRMS (CI) *m/z*: found: 150.0922, calcd for $C_9H_{12}NO$ $[M+NH_4]^+$: 150.0919.

Preparation of chalcones 13b-q by Claisen-Schmidt condensation

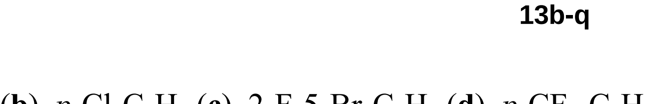

13b-q

R^1=Ph; Ar = *p*-NO_2-C_6H_4 (**b**), *p*-Cl-C_6H_4 (**c**), 2-F-5-Br-C_6H_3 (**d**), *p*-CF_3-C_6H_4 (**e**), 2-naphtyl (**f**), 2-pyridyl (**g**), 3-pyridyl (**h**).

Ar = Ph; R^1 = *p*-Br-C_6H_4 (**i**), 2-Naphtyl (**j**), *p*-Cl-C_6H_4 (**k**), 2-furyl (**l**), 2-thienyl (**m**), *p*-CF_3-C_6H_4 (**n**), 4-pyridyl (**o**), *p*-MeO-C_6H_4 (**p**), 3-pyridyl (**q**).

Compounds **13b-f, 13i-n, 13p** were prepared as described in a reported protocol.[10] *General procedure:* Corresponding benzaldehyde (4.2 mmol) was dissolved in methanol (10 mL) and the resulting solution was cooled down to 0 °C. Then aqueous NaOH solution (10% wt., 2.2 mL) was added dropwise followed by slow addition of the corresponding ketone (4.2 mmol). The reaction mixture was stirred allowed to slowly warm up to room temperature and stirred until the complete consumption of the starting materials (TLC). On completion the reaction mixture was diluted with water, the precipitate formed was collected by filtration, washed with water and pentane/ diethyl ether mixture to afford pure products. Most products obtained were known compounds.

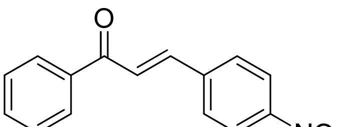

(*E*)-3-(4-nitrophenyl)-1-phenylprop-2-ene-1-one, 13b. Yellow powder; R_f = 0.38 (hexane/ ethyl acetate = 10:1); mp 158–159 °C {lit.[11]138–140 °C}; ^{1}H NMR (400 MHz, $CDCl_3$) δ 7.52–7.55 (m, 2H), 7.61–7.67 (m, 2H), 7.78–7.84 (m, 3H), 8.03–8.05 (m, 2H), 8.26–8.28 (m, 2H); ^{13}C NMR (100 MHz, $CDCl_3$) δ 124.2, 125.7, 128.6, 128.8, 129.0, 133.4, 137.5, 141.0, 141.5, 148.5, 189.6; IR/cm^{-1}: 1657 m, 1608 m, 1595 m, 1578 m, 1512 s, 1446 m, 1406 m, 1413 m, 1333 s, 1318 m, 1289 m, 1218 m, 1185 s, 1159 m, 1105 m, 1014 m, 982 s, 966 m, 844 m, 782 m, 743 s, 705 m, 685 s, 659 s; HRMS (ESI) *m/z*: found: 254.0831, calcd for $C_{15}H_{12}NO_3$ $[M+H]^+$: 254.0817.

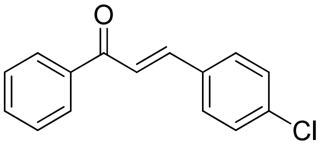

(*E*)-3-(4-chlrophenyl)-1-phenylprop-2-ene-1-one, 13c. White flaky solid; R_f = 0.41 (hexane/ ethyl acetate = 10:1); mp 109–110 °C {lit.[12] 114–115 °C}; ^{1}H NMR (400 MHz, $CDCl_3$) δ 7.38–7.41 (m, 2H), 7.49–7.53 (m, 3H), 7.56–7.62 (m, 3H), 7.76 (d, J = 15.7 Hz, 1H), 8.00–8.03 (m, 2H); ^{13}C NMR (100 MHz, $CDCl_3$) δ 122.4, 128.5, 128.7, 129.3, 129.6, 133.0, 133.4, 136.4, 138.0, 143.3, 190.2; IR/cm^{-1}: 1655 m, 1604 s, 1591 m, 1579 m, 1564 m, 1490 m, 1446 m, 1406 m, 1331 m, 1316 m, 1298 m, 1279 m, 1217 s, 1180 m, 1090 m, 1034 m, 1018 m, 1012 s, 983 s, 930 m, 844 m, 822 s, 797 m, 774 s, 718 m, 687 s; HRMS (ESI) *m/z*: found: 243.0586, calcd for $C_{15}H_{12}OCl$ $[M+H]^+$: 243.0577.

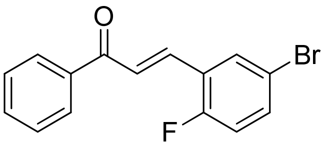

(*E*)-3-(5-bromo-2-fluorophenyl)-1-phenylprop-2-ene-1-one, 13d. Beige powder; mp 76–77 °C (pentane); ^{1}H NMR (400 MHz, $CDCl_3$) δ 7.01–7.06 (m, 1H), 7.46–7.54 (m, 3H), 7.59–7.64 (m, 2H), 7.78 (dd, 1H, J = 6.5, 2.5 Hz), 7.83 (d, 1H, J = 15.8 Hz, C=CH), 8.02–8.04 (m, 2H); ^{13}C NMR (100 MHz, $CDCl_3$) δ 117.1 (d, $^4J_{C,F}$ = 2.8 Hz, C–Br), 118.1 (d, $^2J_{C,F}$ = 23.6 Hz), 125.1 (d, $^2J_{C,F}$ = 12.4 Hz, Cq), 125.5 (d, ^3J = 6.0 Hz), 128.6, 128.8, 131.9 (d, $^3J_{C,F}$ = 2.6 Hz), 133.2, 134.4 (d, $^3J_{C,F}$ = 8.6 Hz), 135.7, 137.7 (Cq), 160.6 (d, $^1J_{C,F}$ = 253.7 Hz, C–F), 189.9(C=O); ^{19}F NMR {^{1}H} (377 MHz, $CDCl_3$) δ –115.8; IR/cm^{-1}: 3064 w, 1658 s, 1604 s, 1593 s, 1577 m, 1476 s, 1448 m, 1407 m, 1339 m, 1316 s, 1277 m, 1266 m, 1235 s,

1217 s, 1181 s, 1172 m, 1104 m, 1015 s, 1035 m, 972 s, 919 m, 860 m, 810 s, 783 s, 773 m, 717 s, 692 s, 679 m, 655 s; HRMS (EI) *m/z*: found: 303.9875, calcd for $C_{15}H_{10}OBrF$ $[M]^+$: 303.9899.

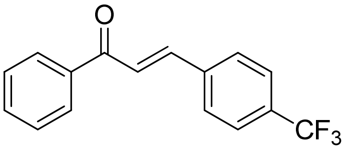

(*E*)-1-phenyl-3-(4-trifluoromethylphenyl)-prop-2-ene-1-one, 13e. White flaky powder; R_f = 0.48 (hexane/ ethyl acetate = 10:1); mp 123–124 °C {lit.[13] 129–131 °C};^{1}H NMR (400 MHz, $CDCl_3$) δ 7.51–7.55 (m, 2H), 7.58–7.63 (m, 2H), 7.68 (d, J = 8.3 Hz, 2H), 7.75 (d, J = 8.3 Hz, 2H), 7.81 (d, J = 15.8 Hz, 1H), 8.02–8.04 (m, 2H); ^{13}C NMR (100 MHz, $CDCl_3$) δ 123.8 (d, $^1J_{C,F}$ = 272.4 Hz), 124.2, 125.9 (q, $^3J_{C,F}$ = 3.8 Hz), 128.5, 128.6, 128.8, 131.9 (d, $^2J_{C,F}$ = 32.9 Hz), 133.2, 137.8, 138.2, 142.8, 190.0; ^{19}F NMR {H} (377 MHz, $CDCl_3$) δ –62.8; IR/cm^{-1}: 1663 m, 1637 s, 1609 m, 1596 m, 1576 m, 1449 m, 1417 m, 1365 m, 1320 s, 1288 m, 1220 m, 1190 m, 1167 s, 1155 s, 1128 m, 1105 s, 1066 m, 1034 s, 1016 s, 983 s, 966 s, 836 s, 779 s, 752 m, 737 s, 695 s, 686 s, 662 s; HRMS (ESI) *m/z*: found: 277.0841, calcd for $C_{16}H_{12}OF_3$ $[M+H]^+$: 277.0840.

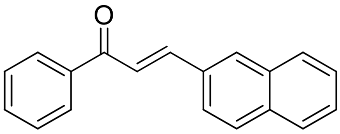

(*E*)-3-(naphthalen-3-yl)-1-phenylprop-2-ene-1-one, 13f. Pale yellow powder; R_f = 0.30 (hexane/ ethyl acetate = 10:1); mp 150–152 °C {lit.[14] 152–154 °C}; ^{1}H NMR (400 MHz, $CDCl_3$) δ 7.51–7.55 (m, 4H), 7.59–7.63 (m, 1H), 7.66 (d, J = 15.7 Hz, 1H), 7.81 (dd, J = 8.5, 1.8 Hz, 1H), 7.85–7.91 (m, 3H), 7.99 (d, J = 15.7 Hz, 1H), 8.05–8.08 (m, 3H); ^{13}C NMR (100 MHz, $CDCl_3$) δ 122.2, 123.7, 126.8, 127.4, 128.6, 128.7, 128.8, 130.7, 132.4, 132.8, 133.4, 134.4, 138.3, 145.0, 190.5; IR/cm^{-1}: 1658 m, 1599 m, 1589 m, 1576 m, 1514 m, 1447 m, 1392 m, 1361 m, 1336 m, 1311 m, 1295 m, 1272 m, 1258 m, 1222 m, 1210 m, 1187 m, 1177 m, 1110 m, 1033 m, 1014 m, 991 m, 984 m, 968 m, 857 m, 846 m, 824 m, 769 m, 782 m, 747 m, 707 m, 687 s, 658 s; HRMS (ESI) *m/z*: found: 259.1128, calcd for $C_{19}H_{15}O$ $[M+H]^+$: 259.1123.

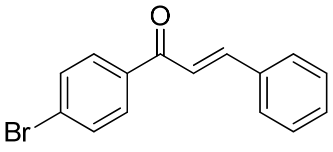

(*E*)-1-(4-bromophenyl)-3-phenylprop-2-ene-1-one, 13i. White powder; R_f = 0.35 (hexane/ ethyl acetate = 10:1); mp 97–98 °C {lit.[15] 90–92 °C}; ^{1}H NMR (400 MHz, $CDCl_3$) δ 7.42–7.44 (m, 3H), 7.48 (d, J = 15.7 Hz, 1H), 7.64–7.66 (m, 4H), 7.82 (d, J = 15.7 Hz, 1H), 7.88–7.90 (m, 2H), ^{13}C NMR (100 MHz, $CDCl_3$) δ 121.4, 127.9, 128.6, 129.0, 130.0, 130.8, 131.9, 134.7, 136.9, 145.4, 189.4; IR/cm^{-1}: 1657 m, 1599 m, 1583 m, 1576 m, 1514 m, 1496 m, 1482 m, 1448 m, 1396 m, 1335 m, 1321 m, 1291 m, 1216 m, 1179 m, 1159 m, 1107 m, 1068 m, 1035 m, 1004 m, 982 s, 892 m, 845 m, 826 m, 791 m, 784 m, 760 m, 747 m, 727 m, 706 m, 690 s, 664 s; HRMS (ESI) *m/z*: found: 287.0084, calcd for $C_{15}H_{12}OBr$ $[M+H]^+$: 287.0072.

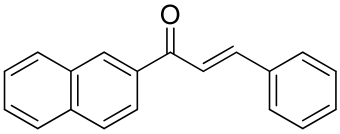

(*E*)-1-(naphthalene-2-yl)-3-phenylprop-2-ene-1-one, 13j. Pale yellow powder; R_f = 0.32 (hexane/ ethyl acetate = 10:1); mp 103–104 °C {lit.[16] 103–104 °C};^{1}H NMR (400 MHz, $CDCl_3$) δ 7.43–7.47 (m, 3H), 7.55–7.63 (m, 2H), 7.68–7.72 (m, 3H), 7.86–8.01 (m, 4H), 8.11 (dd, J = 8.6, 1.8 Hz, 1H), 8.54 (s, 1H); ^{13}C NMR (100 MHz, $CDCl_3$) δ 122.1, 124.5, 126.8, 127.9, 128.4, 128.5, 128.6, 129.0, 129.6, 130.0, 130.6, 132.6, 135.0, 135.5, 144.8, 190.3; IR/cm^{-1}: 1661 s, 1628 m, 1603 s, 1572 m, 1504 m, 1495 m, 1467 m, 1446 m, 1385 m, 1360 m, 1329 m, 1314 m, 1287 m, 1253 m, 1209 m, 1176 m, 1155 m, 1129 m, 1174 m, 1050 m, 984 s, 948 m, 918 m, 878 m, 861 m, 843 m, 820 s, 772 m, 750 s, 707 s, 685 s; HRMS (ESI) *m/z*: found: 259.1118, calcd for $C_{19}H_{15}O$ $[M+H]^+$: 259.1123.

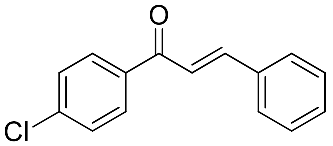

(*E*)-1-(4-chlorophenyl)-3-phenylprop-2-ene-1-one, 13k. White powder; R_f = 0.43 (hexane/ ethyl acetate = 10:1); mp 91–92 °C {lit.[15] 83–85 °C}; ^{1}H NMR (400 MHz, $CDCl_3$) δ 7.42–7.44 (m, 3H), 7.47–7.51 (m, 3H), 7.64–7.66 (m, 2H), 7.82 (d, J = 15.7 Hz, 1H), 7.97 (d, J = 8.7 Hz, 2H); ^{13}C NMR (100 MHz, $CDCl_3$) δ 121.5, 128.5, 129.0, 129.0, 129.9, 130.8, 134.7, 136.5, 139.2, 145.4, 189.2; IR/cm^{-1}: 1659 m, 1601 m, 1589 m, 1574 m, 1567 m, 1485 m, 1448 m, 1399 m, 1332 m, 1286 m, 1216 m, 1178 m, 1155 m, 1084 m, 1035 m, 1009 m, 982 s, 890 m, 872 m, 826 s, 795 m, 761 s, 729 m, 719 m, 689 s, 667 m; HRMS (ESI) *m/z*: found: 243.0576, calcd for $C_{15}H_{12}OCl$ $[M+H]^+$: 243.0577.

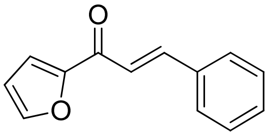

(*E*)-1-(furan-2-yl)-3-phenylprop-2-ene-1-one, 13l. Off-white crystals; R_f = 0.19 (hexane/ ethyl acetate = 10:1); mp 89–91 °C {lit.[16] 83–85 °C}; ^{1}H NMR (400 MHz, $CDCl_3$) δ 6.61 (dd, J = 3.9, 1.9 Hz, 1H), 7.34 (d, J = 3.6 Hz, 1H), 7.42–7.43 (m, 3H),7.46 (d, J = 15.9 Hz, 1H), 7.70–7.62 (m, 3H), 7.89 (d, J = 15.8 Hz, 1H); ^{13}C NMR (100 MHz, $CDCl_3$) δ 112.6, 117.6, 121.1, 128.6, 129.0, 130.6, 134.7, 144.0, 146.6, 153.7, 178.0; HRMS IR/cm^{-1}: 1655 s, 1601 s, 1574 s, 1561 m, 1497 m, 1481 m, 1463 s, 1448 s, 1393 s, 1338 s, 1308 m, 1289 m, 1251 m, 1202 m, 1162 s, 1086 m, 1077 m, 1050 s, 1028 s, 1013 s, 994 s, 978 m, 929 m, 884 m, 865 m, 839 m, 767 s, 756 s, 720 s, 687 s; (ESI) *m/z*: found: 199.0763, calcd for $C_{13}H_{11}O_2$ $[M+H]^+$: 199.0759.

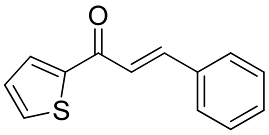

(*E*)-3-phenyl-1-(thiophen-2-yl)prop-2-ene-1-one, 13m. White crystals; R_f = 0.27 (hexane/ ethyl acetate = 10:1); mp 84–85 °C {lit.[16] 82–83 °C}; ^{1}H NMR (400 MHz, $CDCl_3$) δ 7.21–7.17 (m, 1H), 7.47–7.39 (m, 4H), 7.70–7.62 (m, 3H), 7.86 (d, J = 15.7 Hz, 1H), 7.87–7.88 (m, 1H); ^{13}C NMR (100 MHz, $CDCl_3$) δ 182.0, 145.5, 144.1, 134.7, 134.0, 131.9, 130.6, 129.0, 128.5, 128.3, 121.6; IR/cm^{-1}: 1648 s, 1590 s, 1575 m, 1519 m, 1498 m, 1447 m, 1412 s, 1356 m, 1334 s, 1306 m, 1286 m, 1241 m, 1219 s, 1153 m, 1091 m, 1082 m, 1027

m, 994 m, 971 s, 857 m, 849 m, 759 s, 724 m, 734 s, 707 s, 680 s, 662 m; HRMS (ESI) *m/z*: found: 215.0541, calcd for $C_{13}H_{11}OS$ $[M+H]^+$: 215.0531.

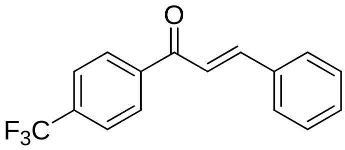

(*E*)-3-phenyl-1-(4-trifluoromethylphenyl)prop-2-ene-1-one, 13n. Pale-yellow crystals; R_f = 0.49 (hexane/ ethyl acetate = 10:1); mp 114–116 °C {lit.[15] 114–116 °C};^{1}H NMR (400 MHz, $CDCl_3$) δ 7.39–7.43 (m, 3H), 7.48 (d, J = 15.7 Hz, 1H), 7.62–7.64 (m, 2H), 7.74 (d, J = 8.2 Hz, 2H), 7.82 (d, J = 15.7 Hz, 1H), 8.08 (d, J = 8.2 Hz, 2H); ^{13}C NMR (100 MHz, $CDCl_3$) δ 121.5, 123.7 (d, $^1J_{C,F}$ = 272.7 Hz), 125.7 (q, $^3J_{C,F}$ = 3.7 Hz), 128.6, 128.8, 129.1, 131.0, 134.0 (d, $^2J_{C,F}$ = 33.2 Hz), 134.5, 141.0, 146.2, 189.7; ^{19}F NMR {H} (377 MHz, $CDCl_3$) δ –63.0; IR/cm^{-1}: 1665 m, 1601 m, 1573 m, 1511 m, 1499 m, 1451 m, 1409 m, 1317 m, 1290m, 1215 m, 1159 m, 1112 s, 1063 s, 1036 s, 997 m, 985 m, 892 s, 876 m, 839 s, 772 s, 762 m, 746 s, 698 m, 688 s, 674 s; HRMS (ESI) *m/z*: found: 277.0849, calcd for $C_{16}H_{12}OF_3$ $[M+H]^+$: 277.0840.

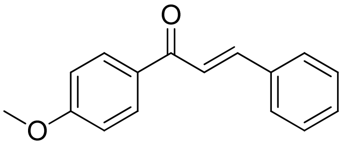

(*E*)-1-(4-methoxyphenyl)-3-phenylprop-2-ene-1-one, 13p. White crystals; R_f = 0.22 (hexane/ ethyl acetate = 10:1); mp 98–100 °C {lit.[15] 94–96 °C}; ^{1}H NMR (400 MHz, $CDCl_3$) δ 3.89 (s, 3H, CH_3O), 6.98–7.00 (m, 2H), 7.39–7.45 (m, 3H), 7.64–7.66 (m, 2H), 7.81 (d, J = 15.7 Hz, 1H), 7.55 (d, J = 15.7 Hz, 1H), 8.01–8.08 (m, 2H); ^{13}C NMR (100 MHz, $CDCl_3$) δ 55.5, 113.9, 121.8, 128.4, 128.9, 130.4, 130.8, 131.1, 135.1, 144.0, 163.4, 188.7; IR/cm^{-1}: 1656 s, 1599 s, 1574 m, 1513 m, 1492 m, 1448 m, 1417 m, 1356 m, 1335 s, 1319 m, 1309 m, 1288 m, 1257 s, 1220 s, 1185 m, 1160 m, 1091 m, 1107 m, 1036 m, 1015 m, 983 m, 970 m, 845 m, 829 m, 761 m, 745 m, 689 s; HRMS (ESI) *m/z*: found: 239.1077, calcd for $C_{16}H_{15}O_2$ $[M+H]^+$: 239.1072.

(*E*)-1-phenyl-3-(pyridine-2 and 3-yl)prop-2-ene-1-ones 13g, 13h were prepared according to the reported procedure.[17] *General procedure*: Acetophenone (4.2 mmol) was added dropwise under cooling (0–5 °C) and stirring to a solution of corresponding pyridinecarboxaldehyde (8.4 mmol) in 1 ml of methanol and 1.7 ml of 10% aq. NaOH. After complete addition, the reaction mixture was stirred for 2 h keeping the temperature at below 10 °C. The resulting suspension was diluted with water; the precipitate was collected by vacuum filtration, washed thoroughly with water, dried and recrystallized from pentane/ ether mixture.

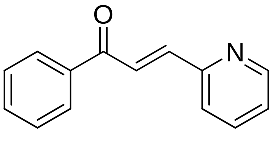

(*E*)-1-phenyl-3-(pyridine-2-yl)prop-2-ene-1-one, 13g. Pale yellow crystals; R_f = 0.50 (hexane/ ethyl acetate = 1:1); mp 59–60 °C (pentane/ ether) {lit.[17] 60–61

°C};^{1}H NMR (400 MHz, $CDCl_3$) δ 7.31 (dd, J = 7.6, 4.9 Hz, 1H), 7.48–7.53 (m, 3H), 7.58–7.62 (m, 1H), 7.74 (td, J = 7.7, 1.8 Hz, 1H), 7.79 (d, J = 15.2 Hz, 1H), 8.09–8.15 (m, 3H), 8.69–8.71 (m, 1H); ^{13}C NMR (100 MHz, $CDCl_3$) δ 124.5, 125.5, 125.6, 128.7, 128.8, 137.0, 137.8, 142.8, 150.2, 153.2, 190.5; IR/cm^{-1}: 1664 s, 1612 s, 1591 s, 1578 s, 1563 s, 1489 m, 1472 m, 1445 m, 1430 s, 1327 s, 1316 s, 1301 s, 1291 m, 1249 s, 1215 m, 1185 m, 1178 s, 1151 s, 1092 s, 1049 m, 1030 m, 1014 m, 991 m, 971 m, 897 m, 869 m, 846 m, 797 m, 756 s, 689 s, 666 m; HRMS (ESI) *m/z*: found: 210.0907, calcd for $C_{14}H_{12}NO$ $[M+H]^+$: 210.0919.

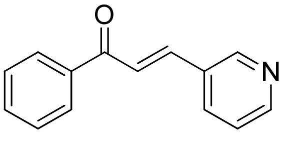

(*E*)-1-phenyl-3-(pyridine-3-yl)prop-2-ene-1-one, 13h. Pale yellow crystals; R_f = 0.21 (hexane/ ethyl acetate = 1:1); mp 100–101 °C (pentane/ ether) {lit.[17] 101–102 °C}; ^{1}H NMR (400 MHz, $CDCl_3$) δ 7.35 (dd, J = 7.9, 4.8 Hz, 1H), 7.49–7.53 (m, 2H), 7.58–7.63 (m, 2H),7.78 (d, J = 15.8 Hz, 1H), 7.95 (dt, J = 7.9, 2.0 Hz, 1H), 8.02–8.04 (m, 2H), 8.62 (dd, J = 4.8, 1.6 Hz, 1H), 8.85 (d, J = 2.1 Hz, 1H); ^{13}C NMR (100 MHz, $CDCl_3$) δ 123.8, 128.6, 128.8, 130.7, 133.2, 134.6, 137.7, 141.0, 150.0, 151.1, 189.9; IR/cm^{-1}: 1658 m, 1601 m, 1579 m, 1565 m, 1514 m, 1473 m, 1447 m, 1423 m, 1415 m, 1337 m, 1309 m, 1219 m, 1186 m, 1122 m, 1106 m, 1041 m, 1014 m, 982 m, 869 m, 855 m, 7845 m, 811 m, 766 m, 746 m, 706 m, 679 s; HRMS (ESI) *m/z*: found: 210.0929, calcd for $C_{14}H_{12}NO$ $[M+H]^+$: 210.0919.

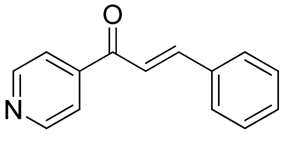

(*E*)-3-phenyl-1-(pyridine-4-yl)prop-2-ene-1-one, 13o.[18] Benzaldehyde (4.7 mmol, 0.5 g) was added to a solution of 10% NaOH (2.5 mL) at 0 °C. Then 4-acetylpyridine (4.7 mmol, 0.57 g) was slowly added dropwise. The solution was stirred at 0 °C for 1 h, allowed to warm up to r.t. and stirred for another 2 h. The resulting suspension was diluted with water; the solid was collected by vacuum filtration and recrystallized from ethanol to afford **13o** as pale-yellow crystals; R_f = 0.26 (hexane/ ethyl acetate = 1:1); mp 86–87 °C (EtOH) {lit.[10] 86–87 °C}; ^{1}H NMR (400 MHz, $CDCl_3$) δ 7.84 (d, J = 15.8 Hz, 4H), 7.80–7.76 (m, 2H), 7.66 (dd, J = 6.7, 2.9 Hz, 2H), 7.49–7.39 (m, 1H), 8.87–8.82 (m, 2H); ^{13}C NMR (100 MHz, $CDCl_3$) δ 121.2, 121.6, 128.7, 129.1, 131.2, 134.3, 144.4, 146.9, 150.8, 189.9; IR/cm^{-1}: 1664 s, 1600 s, 1573 s, 1548 m, 1496 m, 1448 m, 1408 m, 1339 m, 1315 m, 1290 m, 1219 m, 1207 m, 1160 m, 1093 m, 1073 m, 1043 s, 985 s, 891 m, 875 m, 825 s, 763 s, 741 s, 699 s, 683 s, 664 s; HRMS (ESI) *m/z*: found: 210.0911, calcd for $C_{14}H_{12}NO$ $[M+H]^+$: 210.0919.

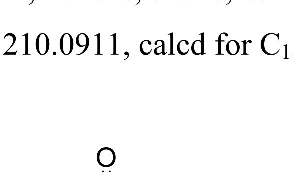

(*E*)-3-phenyl-1-(pyridine-4-yl)prop-2-ene-1-one, 13q.[19] 10% aq. NaOH solution (2.5 ml) was added dropwise to a mixture of 3-acetylpyridine (1.0 g, 8.33 mmol) and benzaldehyde (0.95 g, 9 mmol) in water (50 ml) under vigorous stirring. The stirring was continued for 8 h at room temperature. The precipitated solid was

filtered off, washed several times with water, dried, and recrystallized from ethanol to afford **13q** as pale-yellow crystals; R_f = 0.29 (hexane/ ethyl acetate = 1:1); mp 71–72 °C (EtOH) {lit.[16] 70–72 °C}; ^{1}H NMR (400 MHz, $CDCl_3$) δ 7.44–7.47 (m, 4H), 7.50 (d, J = 15.7 Hz, 1H), 7.65–7.68 (m, 2H), 7.86 (d, J = 15.7 Hz, 1H), 8.30 (dt, J = 8.0, 2.0 Hz, 1H), 8.81 (dd, J = 4.9, 1.8 Hz, 1H), 9.24 (d, J = 2.3 Hz, 1H); ^{13}C NMR (100 MHz, $CDCl_3$) δ 121.3, 123.7, 128.7, 129.1, 131.1, 133.5, 134.4, 136.0, 146.1, 149.7, 153.2, 189.1; R/cm^{-1}: 1664 s, 1601 s, 1584 s, 1496 m, 1484 m, 1449 m, 1414 m, 1350 m, 1330 m, 1317 m, 1290 m, 1231 m, 1205 m, 1114 m, 1085 m, 1048 m, 1017 s, 1000 m, 983 m, 821 m, 797 m, 750 m, 710 s, 688 s, 678 s; HRMS (ESI) *m/z*: found: 210.0924, calcd for $C_{14}H_{12}NO$ $[M+H]^+$: 210.0919.

Michael addition of glycine Schiff base 1a to vinyl ketones 11

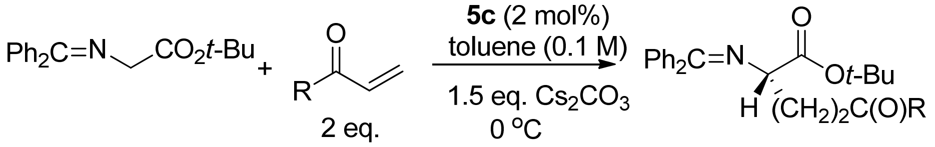

1a **11a-f** **12a-f**

General procedure: tert-Butyl glycinate benzophenone Schiff base **1a** (15.0 mg, 0.05 mmol, 1.0 equiv.), **5c** (0.52 mg, 1.02 µmol, 2 mol%) and Cs_2CO_3 (24.9 mg, 0.08 mmol, 1.5 equiv.) were placed in a Schlenk tube; dry toluene (0.5 mL) was added and the reaction mixture was stirred at 0 °C for 10 min followed by the addition of the corresponding vinyl ketone **11** (0.10 mmol, 2.0 equiv) *via* syringe in one portion. The reaction mixture was effectively stirred (1000 rpm) at 0 °C and monitored by TLC (hexane/ ethyl acetate = 10:1). After indicated time (Table 2), upon complete consumption of **11**, the reaction mixture was directly loaded onto a short silica gel column, followed by gradient elution with hexane/ethyl acetate mixtures.

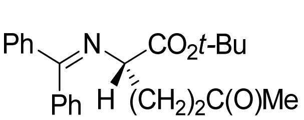

(*S*)-*tert*-Butyl-2-[(diphenylmethylidene)amino]-5-oxohexanoate, 12a. Colorless oil; yield 85%; R_f = 0.18 (hexane/EA = 8:1); $[\alpha]_D^{26}$ = –72.6 (c 0.91, $CHCl_3$) at 93%*ee*; ^{1}H NMR (400 MHz, $CDCl_3$) δ 1.43 (s, 9H, *t*-Bu), 2.12 (s, 3H, Me), 2.12–2.17 (m, 2H, CH_2), 2.44–2.59 (m, 2H, CH_2), 3.96 (t, 1H, J = 6.1 Hz, CH), 7.15–7.18 (m, 2H), 7.31–7.45 (m, 6H), 7.63–7.64 (m, 2H); ^{13}C NMR (100 MHz, $CDCl_3$) δ 27.7 (CH_2), 28.1 ($C(CH_3)_3$), 29.9 (Me), 39.9 (CH_2), 64.7 (CH), 81.2 ($C(CH_3)_3$), 127.7, 128.0, 128.5, 128.6, 128.8, 130.3, 136.5 (Cq), 139.5 (Cq), 170.5 (Cq), 171.0 (Cq), 208.3 (C=O); IR/cm^{-1}: 2976 w, 2929 w, 1720 m, 1623 m, 1598 m, 1576 m, 1445 m, 1367 m, 1283 m, 1253 m, 1149 s, 1093 m, 1074 m, 1029m, 966 m, 847 m, 781 m, 752 m, 697 s; HRMS (ESI) *m/z*: found: 366.2082, calcd for $C_{23}H_{28}NO_3$ $[M+H]^+$: 366.2069; HPLC analysis:

Chiralcel OD-H column; hexane/ IPA = 95:5; flow rate = 1.0 mL/min; λ = 254 nm; 26 °C; t_R = 6.68, 7.36 (major) min.

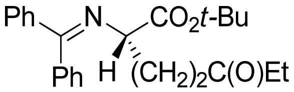

(*S*)-*tert*-Butyl-2-[(diphenylmethylidene)amino]-5-oxoheptanoate, 12b. Colorless oil; yield 92%; R_f = 0.19 (hexane/EA = 8:1); $[\alpha]_D^{26}$ = –56.5 (c 1.06, $CHCl_3$) at 90% *ee*; ^{1}H NMR (400 MHz, $CDCl_3$) δ 1.01 (t, 1H, *J* = 7.3 Hz, CH_2CH_3), 1.43 (s, 9H, *t*Bu), 2.12–2.18 (m, 2H, CH_2), 2.39–2.57 (m, 4H, CH_2CH_3+CH_2), 3.95 (t, 1H, *J* = 6.1 Hz, CH), 7.15–7.17 (m, 2H), 7.30–7.46 (m, 6H), 7.62–7.64 (m, 2H); ^{13}C NMR (100 MHz, $CDCl_3$) δ 7.8 (CH_2CH_3), 27.8 (CH_2), 28.1 ($C(CH_3)_3$), 35.9 (CH_2), 38.5 (CH_2), 64.8 (CH), 81.1 ($C(CH_3)_3$), 127.7, 128.0, 128.5, 128.6, 128.8, 130.3, 136.5 (Cq), 139.5 (Cq), 170.5 (Cq), 171.0 (Cq), 211.0 (C=O); IR/cm^{-1}: 2975 m, 2925 m, 2852 m, 1730 m, 1715 m, 1623 m, 1598 m, 1576 m, 1446 m, 1392 m, 1367 m, 1286 m, 1254 m, 1148 s, 1029 m, 847 m, 810 m, 781 m, 697 s; HRMS (ESI) *m/z*: found: 380.2217, calcd for $C_{24}H_{30}NO_3$ $[M+H]^+$: 380.2226; HPLC analysis: Chiralcel OD-H column; hexane/ IPA = 95:5; flow rate = 1.0 mL/min; λ = 254 nm; 26 °C; t_R = 5.13, 6.39 (major) min.

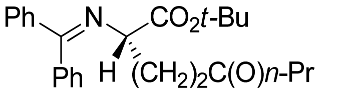

(*S*)-*tert*-Butyl-2-[(diphenylmethylidene)amino]-5-oxooctanoate, 12c. Colorless oil; yield 95%; R_f = 0.21 (hexane/EA = 8:1); $[\alpha]_D^{20}$ = –63.1 (c 1.11, $CHCl_3$) at 92%*ee*; ^{1}H NMR (400 MHz, $CDCl_3$) δ 0.88 (t, *J* = 7.4 Hz, 3H, CH_2CH_3), 1.43 (s, 9H, *t*-Bu), 1.51–1.60 (m, 2H), 2.12–2.17 (m, 2H, CH_2), 2.37 (t, *J* =7.4 Hz, 2H), 2.40–2.55 (m, 2H), 3.95 (t, *J* = 6.1 Hz, 1H, CH), 7.14–7.19 (m, 2H), 7.30–7.34 (m, 2H), 7.37–7.47 (m, 4H), 7.62–7.65 (m, 2H); ^{13}C NMR (100 MHz, $CDCl_3$) δ 13.8 (CH_2CH_3), 17.2 (CH_2), 27.8 (CH_2), 28.1 ($C(CH_3)_3$), 38.9 (CH_2), 44.7 (CH_2), 64.8 (CH), 81.1 ($C(CH_3)_3$), 127.7, 128.0, 128.5, 128.6, 128.8, 130.3, 136.5 (Cq), 139.5 (Cq), 170.4 (Cq), 171.0 (Cq), 210.6 (C=O); IR/cm^{-1}: 2967 m, 2933 m, 2875 w, 1730 m, 1714 m, 1623 m, 1598 m, 1577 m, 1491 m, 1446 m, 1367 m, 1315 m, 1287 m, 1252 m, 1148 s, 1100 m, 1029 m, 962 m, 847 m, 781 m, 753 m, 697 s; HRMS (ESI) *m/z*: found: 394.2377, calcd for $C_{25}H_{32}NO_3$ $[M+H]^+$: 394.2382; HPLC analysis: Chiralcel OD-H column; hexane/ IPA = 95:5; flow rate = 1.0 mL/min; λ = 254 nm; 18 °C; t_R = 4.83, 5.36 (major) min.

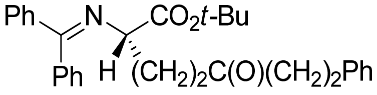

(*S*)-*tert*-Butyl-2-[(diphenylmethylidene)amino]-5-oxo-7-phenylheptanoate, 12d. Colorless oil; yield 94%; R_f = 0.10 (hexane/EA = 10:1); $[\alpha]_D^{20}$ = –48.6 (c 0.73, $CHCl_3$) at 85%*ee*; ^{1}H NMR (400 MHz, $CDCl_3$) δ 1.42 (s, 9H, *t*-Bu), 2.12–2.18 (m, 2H), 2.40–2.56 (m, 2H), 2.70–2.76 (m, 2H), 2.80–2.87 (m, 2H), 3.94 (t, *J* = 6.0, 1H), 7.13–7.19 (m, 5H), 7.23–7.27 (m, 2H), 7.30–7.34 (m, 2H), 7.37–7.39 (m, 1H), 7.40–7.45 (m, 3H), 7.61–7.64 (m, 2H); ^{13}C NMR (100 MHz, $CDCl_3$) δ 27.7 (CH_2), 28.1 ($C(CH_3)_3$), 29.7 (CH_2), 39.1

(CH_2), 44.3 (CH_2), 64.8 (CH), 81.2 (*C*(CH_3)$_3$), 126.1, 127.7, 128.1, 128.3, 128.48, 128.51, 128.6, 128.8, 130.4, 136.5 (Cq), 139.5 (Cq), 141.1 (Cq), 170.5 (Cq), 171.0 (Cq), 209.4 (C=O); IR/cm^{-1}: 3061 w, 3026 w, 2976 m, 2929 m, 1720 s, 1720 m, 1623 m, 1600 m, 1577 m, 1494 m, 1477 m, 1446 m, 1410 m, 1367 m, 1315 m, 1286 m, 1252 m, 1147 s, 1102 m, 1077 m, 1020 m, 1001 m, 912 m, 849 m, 781 m, 749 m, 732 m, 696 s; HRMS (ESI) *m/z*: found: 456.2527, calcd for $C_{30}H_{34}NO_3$ $[M+H]^+$: 456.2539; HPLC analysis: Chiralpak AS-H column; hexane/ IPA = 90:10; flow rate = 1.0 mL/min; λ = 254 nm; 18 °C; t_R = 4.87 (major), 5.92 min.

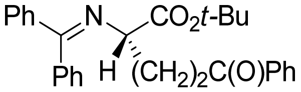

(*S*)-*tert*-Butyl-2-[(diphenylmethylidene)amino]-5-phenyl-5-oxopentanoate, 12e. Colorless oil; yield 82%; R_f = 0.15 (hexane/EA = 9:1); $[\alpha]_D^{20}$ = –32.8 (c 0.61, $CHCl_3$) at 80%*ee*; ^1H NMR (400 MHz, $CDCl_3$) δ 1.45 (s, 9H, *t*Bu), 2.24–2.39 (m, 2H, CH_2), 3.02 (ddd, *J* = 17.1, 8.2, 6.5, 1H), 3.13 (ddd, *J* = 17.1, 8.5, 6.8, 1H), 4.07 (dd, *J* = 6.8, 5.4, 1H), 7.13–7.15 (m, 2H), 7.29–7.33 (m, 2H), 7.36–7.46 (m, 6H), 7.52–7.57 (m, 1H), 7.63–7.66 (m, 2H), 7.93–7.95 (m, 2H); ^{13}C NMR (100 MHz, $CDCl_3$) δ 28.1, 28.2 (CH_2), 34.7 (CH_2), 64.8 (CH), 81.2 (*C*(CH_3)$_3$), 127.7, 128.0, 128.1, 128.48, 128.55, 128.59, 128.8, 130.3, 132.9, 136.5 (Cq), 136.9 (Cq), 139.5 (Cq), 170.6 (Cq), 171.1 (Cq), 199.7 (C=O); IR/cm^{-1}: 3060 w, 2976 m, 2932 m, 1728 m, 1684 m, 1622 m, 1598 m, 1578 m, 1447 m, 1367 m, 1315 m, 1273 m, 1252 m, 1222 m, 1147 s, 1078 m, 1029 m, 996 m, 916 m, 847 m, 780 m, 693 s; HRMS (ESI) *m/z*: found: 428.2239, calcd for $C_{28}H_{30}NO_3$ $[M+H]^+$: 428.2226; HPLC analysis: Chiralcel OD-H column; hexane/ IPA = 95:5; flow rate = 1.0 mL/min; λ = 254 nm; 18 °C; t_R = 5.64, 6.92 (major) min.

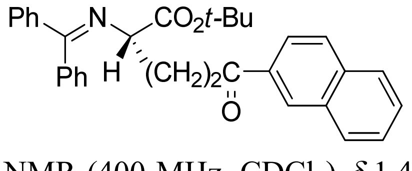

(*S*)-*tert*-Butyl-2-[(diphenylmethylidene)amino]-5-(naphthalene-2-yl)-5-oxopentanoate, 12f. Colorless oil; yield 76%; R_f = 0.16 (hexane/EA = 10:1); $[\alpha]_D^{20}$ = –20.4 (c 0.8, $CHCl_3$) at 83%*ee*; ^1H NMR (400 MHz, $CDCl_3$) δ 1.46 (s, 9H, *t*-Bu), 2.35–2.41 (m, 2H), 3.11–3.19 (m, 1H), 3.26–3.34 (m, 1H), 4.12 (t, *J* =6.0 Hz, 2H), 7.13–7.16 (m, 2H), 7.29–7.33 (m, 2H), 7.36–7.44 (m, 4H), 7.52–7.61 (m, 2H), 7.65–7.67 (m, 2H), 7.87 (dd, *J* = 8.3, 3.4 Hz, 2H), 7.93 (d, *J* = 7.9 Hz, 1H), 8.01 (dd, *J* = 8.6, 1.7 Hz, 1H), 8.48 (s, 1H); ^{13}C NMR (100 MHz, $CDCl_3$) δ 28.1 (C(*C*H$_3$)$_3$), 28.5, 34.8 (CH_2), 64.8 (CH), 81.2 (*C*(CH_3)$_3$), 124.0, 126.7, 127.8, 128.0, 128.4, 128.5, 128.6, 128.8, 129.6, 129.8, 130.3, 132.5 (Cq), 134.2 (Cq), 135.6 (Cq), 136.5 (Cq), 139.5 (Cq), 170.7 (Cq), 171.1 (Cq), 199.7 (C=O); IR/cm^{-1}: 3059 w, 2976 m, 2932 w, 1727 s, 1679 s, 1624 m, 1597 m, 1576 m, 1468 m, 1445 m, 1391 m, 1367 m, 1315 m, 1277 m, 1255 m, 1221 m, 1147 s, 1124 s, 1076 m, 1028 m, 1002 m, 942 m, 910 m, 862 m, 847 m, 820 m, 781 m, 748 s, 696 s; HRMS (ESI) *m/z*: found: 478.2381, calcd for $C_{32}H_{32}NO_3$ $[M+H]^+$:

478.2382; HPLC analysis: Chiralcel OD-H column; hexane/ IPA = 90:10; flow rate = 0.5 mL/min; λ = 254 nm; 18 °C; t_R = 11.46, 12.89 (major) min.

Michael addition of glycine Schiff base 1a to chalcones 13

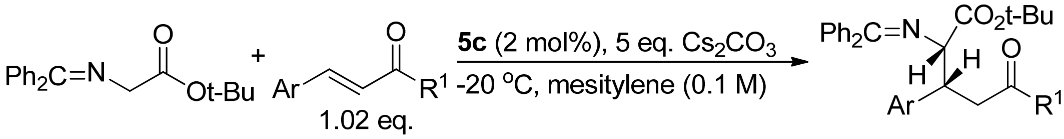

1a **13a-o** **14a-o**

General procedure: tert-Butyl glycinate benzophenone Schiff base **1a** (15.0 mg, 0.05 mmol, 1.0 equiv.), **5c** (0.52 mg, 1.02 μmol, 2 mol%) and Cs_2CO_3 (83.1 mg, 0.26 mmol, 5.0 equiv.) were placed in a Schlenk tube; dry mesitylene (0.5 mL) was added and the reaction mixture was stirred at –20 °C for 10 min followed by the addition of the corresponding chalcone (0.051 mmol, 1.02 equiv). The reaction mixture was effectively stirred (1000 rpm) at –20 °C and monitored by TLC (hexane/ ethyl acetate = 10:1). After indicated time (Table 3), upon complete consumption of **13**, the reaction mixture was directly loaded onto a short silica gel column, followed by gradient elution with hexane/ethyl acetate mixtures.

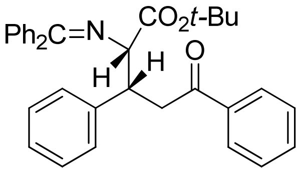

(2*S*,3*R*)-*tert*-Butyl-2-((diphenylmethylene)amino)-5-oxo-3,5-diphenylpentanoate, 14a. Colorless oil; yield 98%; R_f = 0.23 (hexane/EA = 9:1); $[\alpha]_D^{18}$ = –81.7 (c 1.05, CH_2Cl_2) at 93%*ee*; ^{1}H NMR (400 MHz, $CDCl_3$) δ 1.31 (s, 9H, *t*-Bu), 3.60 (dd, J = 16.9, 3.5 Hz, 1H), 3.75 (dd, J = 16.9, 9.7 Hz, 1H), 4.12–4.22 (m, 2H), 6.70 (d, J = 7.2 Hz, 2H), 7.10–7.18 (m, 5H), 7.29–7.55 (m, 9H), 7.66–7.68 (m, 2H), 7.96–7.97 (m, 2H); ^{13}C NMR (100 MHz, $CDCl_3$) δ 27.9, 40.0 (CH_2), 44.8, 70.9, 81.3 ($C(CH_3)_3$), 126.6, 127.5, 128.0, 128.1, 128.2, 128.4, 128.5, 128.6, 128.9, 130.3, 132.8, 136.3 (Cq), 137.2 (Cq), 139.4 (Cq), 141.3 (Cq), 171.0 (Cq), 171.1 (Cq), 198.7 (C=O); IR/cm^{-1}: 3061 w, 3029 w, 2977 m, 2929 m, 1727 m, 1685 m, 1622 m, 1597 m, 1578 m, 1493 m, 1447 m, 1392 m, 1368 m, 1285 m, 1253 m, 1148 s, 1078 m, 1003 m, 910 m, 846 m, 780 m, 749 m, 732 m, 693 s; HRMS (ESI) *m/z*: found: 504.2531, calcd for $C_{34}H_{34}NO_3$ $[M+H]^+$: 504.2539; HPLC analysis: Chiralpak AD-H column; hexane/ IPA = 90:10; flow rate = 1.0 mL/min; λ = 254 nm; 18 °C; t_R = 6.94, 8.81 (major) min.

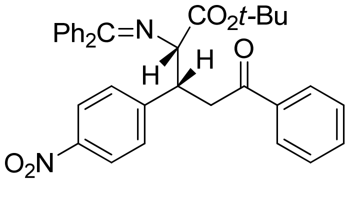

(2*S*,3*R*)-*tert*-Butyl-2-((diphenylmethylene)amino)-5-oxo-3-(*p*-nitrophenyl)-5-phenylpentanoate, 14b. Colorless oil; yield 96%; R_f = 0.14 (hexane/EA = 9:1); $[\alpha]_D^{18}$ = –45.2 (c 1.00, CH_2Cl_2) at 90%*ee*; ^{1}H NMR (400 MHz, $CDCl_3$) δ 1.36 (s, 9H, *t*-Bu), 3.68 (dd, J = 17.6, 3.4 Hz,

1H), 3.89 (dd, J = 17.6, 10.6 Hz, 1H), 4.18 (d, J = 4.6 Hz, 1H), 4.26–4.30 (m, 1H), 6.73 (d, J = 7.0 Hz, 2H), 7.31–7.48 (m, 10H), 7.55–7.59 (m, 1H), 7.65–7.67 (m, 2H), 7.96–7.98 (m, 2H), 8.04–8.06 (m, 2H); ^{13}C NMR (100 MHz, $CDCl_3$) δ 27.9, 39.5 (CH_2), 44.4, 70.0, 82.0 ($C(CH_3)_3$), 123.3, 127.3, 128.1, 128.2, 128.4, 128.7, 128.8, 129.5, 130.7, 133.3, 135.9 (Cq), 136.7 (Cq), 138.9 (Cq), 146.6 (Cq), 149.7 (Cq), 169.4 (Cq), 171.9 (Cq), 197.9 (C=O); IR/cm^{-1}: 3062 w, 2977 m, 2931 m, 1726 m, 1684 m, 1598 m, 1579 m, 1519 s, 1447 m, 1392 m, 1368 m, 1344 m, 1316 m, 1288 m, 1252 m, 1146 s, 1110 m, 1003 m, 910 m, 856 m, 780 m, 750 m, 727 m, 692 s; HRMS (ESI) *m/z*: found: 549.2396, calcd for $C_{34}H_{33}N_2O_5$ $[M+H]^+$: 549.2389; HPLC analysis: Chiralcel OD-H column; hexane/ IPA = 90:10; flow rate = 1.0 mL/min; λ = 254 nm; 18 °C; t_R = 6.47, 8.56 (major) min.

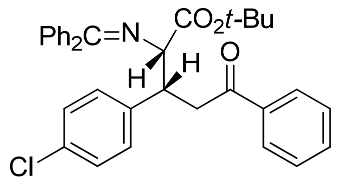

(2*S*,3*R*)-*tert*-Butyl-2-((diphenylmethylene)amino)-5-oxo-3-(*p*-chlorophenyl)-5-phenylpentanoate, 14c. Colorless oil; yield 94%; R_f = 0.22 (hexane/EA = 9:1); $[\alpha]_D^{18}$ = –70.9 (c 1.02, CH_2Cl_2) at 91%*ee*; ^{1}H NMR (400 MHz, $CDCl_3$) δ 1.34 (s, 9H, *t*-Bu), 3.60 (dd, J = 17.1, 2.9 Hz, 1H), 3.75 (dd, J = 17.1, 9.7 Hz, 1H), 4.13–4.19 (m, 2H), 6.74 (d, J = 6.5 Hz, 2H), 7.08 (d, J = 8.4 Hz, 2H), 7.14 (d, J = 8.4 Hz, 2H), 7.32–7.56 (m, 9H), 7.67 (d, J = 7.4 Hz, 2H), 7.96 (d, J = 7.4 Hz, 2H); ^{13}C NMR (100 MHz, $CDCl_3$) δ 27.9, 39.8 (CH_2), 44.1, 70.6, 81.6 ($C(CH_3)_3$), 127.4, 128.1, 128.17, 128.24, 128.3, 128.5, 128.6, 128.8, 129.9, 130.5, 132.3 (Cq), 133.0, 136.2 (Cq), 137.0 (Cq), 139.2 (Cq), 140.0 (Cq), 169.8 (Cq), 171.5 (Cq), 198.4 (C=O); IR/cm^{-1}: 3056 w, 3003 w, 2976 m, 2927 m, 1731 m, 1674 m, 1621 m, 1597 m, 1579 m, 1492 m, 1446 m, 1371 m, 1316 m, 1283 m, 1262 m,1235 ,m, 1144 s, 1109 m, 1090 m, 909 m, 837 m, 814 m, 783 m, 754 m, 77 m, 719 m, 692 s; HRMS (ESI) *m/z*: found: 538.2134, calcd for $C_{34}H_{33}NO_3Cl$ $[M+H]^+$: 538.2149; HPLC analysis: Chiralcel OD-H column; hexane/ IPA = 90:10; flow rate = 1.0 mL/min; λ = 254 nm; 18 °C; t_R = 4.41, 5.55 (major) min.

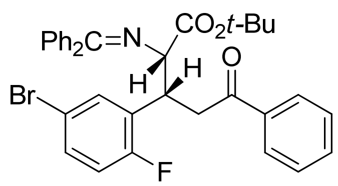

(2*S*,3*R*)-*tert*-Butyl-2-((diphenylmethylene)amino)-5-oxo-3-(5-bromo-2-fluoro)-5-phenylpentanoate, 14d. Colorless oil; yield 94%; R_f = 0.28 (hexane/EA = 9:1); $[\alpha]_D^{18}$ = –43.5 (c 1.26, CH_2Cl_2) at 93%*ee*; ^{1}H NMR (400 MHz, $CDCl_3$) δ 1.32 (s, 9H, *t*-Bu), 3.59 (dd, J = 17.4, 3.9 Hz, 1H), 3.73 (dd, J = 17.4, 10.2 Hz, 1H), 4.24 (d, J = 5.9 Hz, 1H), 4.40 (ddd, J = 10.2, 5.9, 3.9 Hz, 1H), 6.70–6.87 (m, 3H), 7.21–7.25 (m, 1H), 7.31–7.47 (m, 9H), 7.53–7.57 (m, 1H), 7.65–7.67 (m, 2H), 7.96–7.98 (m, 2H); ^{13}C NMR (100 MHz, $CDCl_3$) δ 27.9, 38.6, 39.0 (CH_2), 68.8, 81.7 ($C(CH_3)_3$), 116.2 (d, $^4J_{C,F}$ = 2.3 Hz, C–Br), 117.2 (d, $^2J_{C,F}$ = 24.4 Hz), 127.5, 128.1 (d, ^3J = 6.2 Hz), 128.4, 128.6, 128.7, 128.9, 130.6, 130.8 (d, $^2J_{C,F}$ = 15.6 Hz, Cq), 130.9 (d, $^3J_{C,F}$ = 8.4 Hz), 133.1, 133.2, 136.2 (Cq), 138.0 (d, $^1J_{C,F}$ = 236.5 Hz, C–F), 158.8 (Cq), 161.2 (Cq), 169.6 (Cq), 172.0 (Cq), 197.9 (C=O); ^{19}F {^{1}H} NMR (377

MHz, $CDCl_3$) δ –118.1; IR/cm^{-1}: 3060 w, 2978 m, 2932 w, 1727 s, 1685 m, 1622 m, 1597 m, 1578 m, 1483 s, 1447 m, 1393 m, 1368 m, 1316 m, 1287 m, 1238 s, 1148 s, 1112 m, 1003 m, 909 m, 877 m, 814 m, 780 m, 747 s, 731 s, 693 s; HRMS (ESI) *m/z*: found: 600.1545, calcd for $C_{34}H_{32}NO_3BrF$ $[M+H]^+$: 600.1550; HPLC analysis: Chiralcel OD-H column; hexane/ IPA = 95:5; flow rate = 1.0 mL/min; λ = 254 nm; 18 °C; t_R = 10.30, 11.37 (major) min.

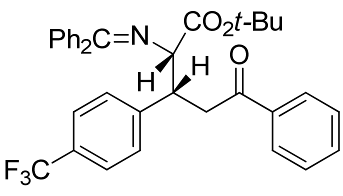

(2*S*,3*R*)-*tert*-Butyl-2-((diphenylmethylene)amino)-5-oxo-3-(p-trifluoromethyl)-5-phenylpentanoate, 14e. Colorless oil; yield 92%; R_f = 0.24 (hexane/EA = 9:1); $[\alpha]_D^{18}$ = –93.5 (c 1.15, CH_2Cl_2) at 91%*ee*; ^{1}H NMR (400 MHz, $CDCl_3$) δ 1.34 (s, 9H, *t*-Bu), 3.67 (dd, *J* = 17.4, 3.6 Hz, 1H), 3.84 (dd, *J* = 17.4, 10.4 Hz, 1H), 4.16 (d, *J* = 4.7 Hz, 1H), 4.22–4.27 (m, 1H), 6.68 (d, *J* = 7.0 Hz, 2H), 7.25–7.59 (m, 13H), 7.66–7.68 (m, 2H), 7.96–7.98 (m, 2H); ^{13}C NMR (100 MHz, $CDCl_3$) δ 27.9, 39.4 (CH_2), 44.4, 70.4, 81.7 (*C*(CH_3)$_3$), 124.2 (d, $^1J_{C,F}$ = 271.4 Hz), 125.1 (q, $^3J_{C,F}$ = 3.5 Hz), 127.3, 128.2, 128.3, 128.5, 128.6, 128.8, 128.9, 130.6, 133.1, 136.0 (Cq), 136.9 (Cq), 139.1 (Cq), 145.8 (Cq), 169.7 (Cq), 171.6 (Cq), 198.2 (C=O); ^{19}F {^{1}H} NMR (377 MHz, $CDCl_3$) δ –62.4; IR/cm^{-1}: 3061 w, 2978 m, 2932 m, 1727 m, 1686 m, 1619 m, 1598 m, 1579 m, 1448 m, 1422 m, 1393 m, 1369 m, 1324 s, 1287 m, 1253 m, 1150 s, 1117 s, 1068 s, 1017 m, 1003 m, 911 m, 842 m, 780 m, 762 m, 732 m, 694 s; HRMS (ESI) *m/z*: found: 572.2399, calcd for $C_{35}H_{33}NO_3F_3$ $[M+H]^+$: 572.2413; HPLC analysis: Chiralcel OD-H column; hexane/ IPA = 90:10; flow rate = 1.0 mL/min; λ = 254 nm; 18 °C; t_R = 4.06, 4.69 (major) min.

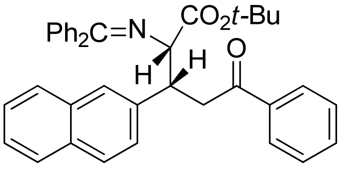

(2*S*,3*R*)-*tert*-Butyl-2-((diphenylmethylene)amino)-5-oxo-3-(2'-naphtyl)-5-phenylpentanoate, 14f. Colorless oil; yield 96%; R_f = 0.16 (hexane/EA = 9:1); $[\alpha]_D^{18}$ = –45.6 (c 1.14, CH_2Cl_2) at 85%*ee*; ^{1}H NMR (400 MHz, $CDCl_3$) δ 1.30 (s, 9H, *t*-Bu), 3.70 (dd, *J* = 17.1, 3.6 Hz, 1H), 3.90 (dd, *J* = 17.1, 10.3 Hz, 1H), 4.27 (d, *J* = 4.9 Hz, 1H), 4.34–4.39 (m, 1H), 6.61 (d, *J* = 6.9 Hz, 2H), 7.19 (t, *J* = 7.6 Hz, 2H), 7.29–7.46 (m, 9H), 7.52–7.73 (m, 7H), 7.98 (d, *J* = 7.5 Hz, 2H); ^{13}C NMR (100 MHz, $CDCl_3$) δ 27.9, 40.0 (CH_2), 44.8, 70.9, 81.4 (*C*(CH_3)$_3$), 125.3, 125.7, 126.9, 127.3, 127.4, 127.6, 127.7, 128.0, 128.16, 128.22, 128.3, 128.5, 128.9, 130.4, 132.4 (Cq), 132.9, 133.3 (Cq), 136.2 (Cq), 137.1 (Cq), 138.9 (Cq), 139.4 (Cq), 170.1 (Cq), 171.3 (Cq), 198.6 (C=O); IR/cm^{-1}: 3059 m, 2978 m, 2929 m, 1727 m, 1685 m, 1623 m, 1598 m, 1579 m, 1447 m, 1393 m, 1368 m, 1315 m, 1287 m, 1256 m, 1146 s, 1074 m, 1003 m, 908 m, 846 m, 780 m, 729 s, 692 s; HRMS (ESI) *m/z*: found: 554.2695, calcd for $C_{38}H_{36}NO_3$ $[M+H]^+$: 554.2695; HPLC analysis: Chiralcel OD-H column; hexane/ IPA = 90:10; flow rate = 1.0 mL/min; λ = 254 nm; 18 °C; t_R = 5.05, 6.67 (major) min.

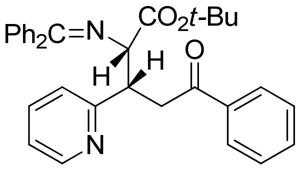

(2*S*,3*S*)-*tert*-Butyl-2-((diphenylmethylene)amino)-5-oxo-3-(2'-pyridyl)-5-phenylpentanoate, 14g. Colorless oil; yield 96%; R_f = 0.64 (hexane/EA = 1:1); $[\alpha]_D^{18}$ = –60.4 (c 1.3, CH_2Cl_2) at 93%*ee*; ^{1}H NMR (400 MHz, $CDCl_3$) δ 1.31 (s, 9H, *t*-Bu), 3.48 (dd, *J* = 17.4, 3.1 Hz, 1H), 4.09 (dd, *J* = 17.4, 10.6 Hz, 1H), 4.34 (ddd, *J* = 10.6, 5.8, 3.1 Hz, 1H), 4.43 (d, *J* = 5.8 Hz, 1H), 6.87 (d, *J* = 6.4 Hz, 2H), 7.00–7.03 (m, 1H), 7.22–7.24 (m, 1H), 7.29–7.54 (m, 10H), 7.62–7.64 (m, 2H), 7.98–8.00 (m, 2H), 8.37–8.39 (m, 1H); ^{13}C NMR (100 MHz, $CDCl_3$) δ 27.9, 39.0 (CH_2), 46.5, 69.9, 81.3 (*C*(CH_3)$_3$), 121.4, 124.1, 127.7, 128.0, 128.2, 128.3, 128.5, 128.6, 129.0, 130.3, 132.9, 135.9, 136.3 (Cq), 137.2 (Cq), 139.6 (Cq), 148.8, 160.8 (Cq), 170.1 (Cq), 171.3 (Cq), 199.0 (C=O); IR/cm^{-1}: 3060 w, 2978 m, 2930 w, 1726 s, 1683 m, 1621 m, 1592 m, 1571 m, 1473 m, 1447 m, 1436 m, 1393 m, 1368 m, 1316 m, 1287 m, 1252 m, 1147 s, 1074 m, 1029 m, 997 m, 923 m, 846 m, 782 m, 770 m, 749 s, 693 s; HRMS (ESI) *m/z*: found: 505.2481, calcd for $C_{33}H_{33}N_2O_3$ $[M+H]^+$: 505.2491; HPLC analysis: Chiralcel OD-H column; hexane/ IPA = 90:10; flow rate = 1.0 mL/min; λ = 254 nm; 18 °C; t_R = 5.65, 11.26 (major) min.

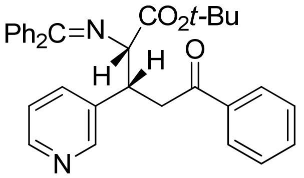

(2*S*,3*R*)-*tert*-Butyl-2-((diphenylmethylene)amino)-5-oxo-3-(4'-pyridyl)-5-phenylpentanoate, 14h. Colorless oil; yield 98%; R_f = 0.45 (hexane/EA = 1:1); $[\alpha]_D^{18}$ = –73.9 (c 1.1, CH_2Cl_2) at 93%*ee*; ^{1}H NMR (400 MHz, $CDCl_3$) δ 1.34 (s, 9H, *t*-Bu), 3.65 (dd, *J* = 17.3, 3.3 Hz, 1H), 3.79 (dd, *J* = 17.3, 9.8 Hz, 1H), 4.16–4.22 (m, 2H), 6.76 (d, *J* = 6.7 Hz, 2H), 7.11 (dd, *J* = 7.6, 4.9 Hz, 1H), 7.32–7.57 (m, 10H), 7.68 (d, *J* = 7.5 Hz, 2H), 7.95 (d, *J* = 7.5 Hz, 2H), 8.40 (d, *J* = 4.4 Hz, 1H), 8.43 (s, 1H); ^{13}C NMR (100 MHz, $CDCl_3$) δ 27.9, 39.6 (CH_2), 42.3, 70.3, 81.8 (*C*(CH_3)$_3$), 123.0, 127.3, 128.1, 128.4, 128.6, 128.9, 130.6, 133.1, 136.1 (Cq), 136.2, 136.9 (Cq), 137.0 (Cq), 139.0 (Cq), 148.0, 150.1, 169.6 (Cq), 171.8 (Cq), 198.1 (C=O); IR/cm^{-1}: 3059 w,2978 m, 2928 m, 1726 m, 1684 m, 1622 m, 1597 m, 1576 m, 1478 m, 1447 m, 1426 m, 1393 m, 1368 m, 1286 m, 1252 m, 1214, 1147 s, 1027 m, 1002 m, 913 m, 845 m, 779 m, 752 m, 730 m, 693 s; HRMS (ESI) *m/z*: found: 505.2483, calcd for $C_{33}H_{33}N_2O_3$ $[M+H]^+$: 505.2491; HPLC analysis: Chiralcel OD-H column; hexane/ IPA = 98:2; flow rate = 1.0 mL/min; λ = 254 nm; 18 °C; t_R = 33.46, 37.14 (major) min.

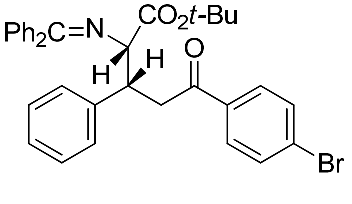

(2*S*,3*R*)-*tert*-Butyl-2-((diphenylmethylene)amino)-5-oxo-3-phenyl-5-(*p*-bromophenyl)pentanoate, 14i. Colorless oil; yield 88%; R_f = 0.26 (hexane/EA = 9:1); $[\alpha]_D^{14}$ = –42.5 (c 1.00, CH_2Cl_2) at 85%*ee*; ^{1}H NMR (400 MHz, $CDCl_3$) δ 1.31 (s, 9H, *t*-Bu), 3.59 (dd, *J* = 16.7, 3.5 Hz, 1H), 3.65–3.74 (m, 1H), 4.11–4.18 (m, 2H), 6.68 (d, *J* = 7.0 Hz, 2H), 7.11–7.19 (m, 5H), 7.25–7.43 (m,

6H), 7.56–7.58 (m, 2H), 7.65–7.67 (m, 2H), 7.82–7.84 (m, 2H); ^{13}C NMR (100 MHz, $CDCl_3$) 27.9, 40.0 (CH_2), 44.8, 70.8, 81.4 (*C*(CH_3)$_3$), 126.7, 127.5, 128.0 (Cq), 128.1, 128.2, 128.4, 128.5, 128.8, 129.8, 130.4, 131.8, 135.9 (Cq), 136.2 (Cq), 139.3 (Cq), 141.2 (Cq), 170.0 (Cq), 171.3 (Cq), 197.8 (C=O); IR/cm^{-1}: 3061 w, 3029 w, 2978 m, 2931 w, 1726 m, 1686 m, 1622 m, 1585 m, 1489 m, 1454 m, 1395 m, 1368 m, 1315 m, 1287 m, 1252 m, 1149 s, 1070 m, 1030 m, 1002 m, 908 m, 843 m, 810 m, 780 m, 729 s, 696 s; HRMS (ESI) *m/z*: found: 582.1627, calcd for $C_{34}H_{33}NO_3Br$ $[M+H]^+$: 582.1644; HPLC analysis: Chiralcel OD-H column; hexane/ IPA = 90:10; flow rate = 1.0 mL/min; λ = 254 nm; 18 °C; t_R = 5.12, 6.07 (major) min.

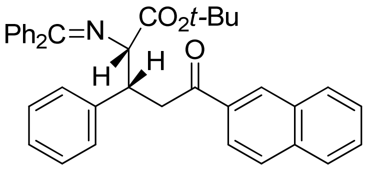

(2*S*,3*R*)-*tert*-Butyl-2-((diphenylmethylene)amino)-5-oxo-3-phenyl-5-(2'-naphtyl)pentanoate, 14j. Colorless oil; yield 92%; R_f = 0.31 (hexane/EA = 9:1); $[\alpha]_D^{18}$ = –17.2 (c 1.08, CH_2Cl_2) at 86%*ee*; ^{1}H NMR (400 MHz, $CDCl_3$) δ 1.33 (s, 9H, *t*-Bu), 3.74 (dd, *J* = 16.7, 3.8 Hz, 1H), 3.90 (dd, *J* = 16.7, 9.9 Hz, 1H), 4.21 (d, *J* = 5.0 Hz, 1H), 4.24–4.28 (m, 1H), 6.72 (d, *J* = 6.9 Hz, 2H), 7.11–7.17 (m, 5H), 7.29–7.43 (m, 6H), 7.53–7.61 (m, 2H), 7.69–7.71 (m, 2H), 7.84–7.86 (m, 2H), 7.98–8.01 (m, 2H), 8.55 (s, 1H); ^{13}C NMR (100 MHz, $CDCl_3$) δ 27.9, 40.0 (CH_2), 44.9, 71.0, 81.4 (*C*(CH_3)$_3$), 124.1, 126.6, 126.7, 127.5, 127.8, 128.1, 128.1, 128.2, 128.23, 128.3, 128.4, 128.6, 128.9, 129.6, 129.9, 130.4, 132.6, 134.5, 135.5, 136.3 (Cq), 139.4 (Cq), 141.4 (Cq), 170.1 (Cq), 171.3 (Cq), 198.7 (C=O); IR/cm^{-1}: 3060 m, 3029 w, 2978 m, 2928 w, 1726 m, 1679 m, 1625 m, 1597 m, 1575 m, 1493 m, 1469 m, 1446 m, 1392 m, 1368 m, 1315 m, 1280 m, 1258 m, 123 m, 1147 s, 1124 m, 1029 m, 908 m, 861 m, 846 m, 818 m, 774 m, 732 m, 696 s; HRMS (ESI) *m/z*: found: 554.2665, calcd for $C_{38}H_{36}NO_3$ $[M+H]^+$: 554.2695; HPLC analysis: Chiralcel OD-H column; hexane/ IPA = 90:10; flow rate = 1.0 mL/min; λ = 254 nm; 18 °C; t_R = 5.70, 6.85 (major) min.

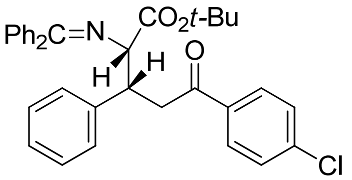

(2*S*,3*R*)-*tert*-Butyl-2-((diphenylmethylene)amino)-5-oxo-3-phenyl-5-(*p*-chlorophenyl)pentanoate, 14k. Colorless oil; yield 90%; R_f = 0.28 (hexane/EA = 9:1); $[\alpha]_D^{18}$ = –63.4 (c 0.98, CH_2Cl_2) at 87%*ee*; ^{1}H NMR (400 MHz, $CDCl_3$) δ 1.32 (s, 9H, *t*-Bu), 3.60 (dd, *J* = 16.7, 3.3 Hz, 1H), 3.66–3.73 (m, 1H), 4.12–4.18 (m, 2H), 6.68 (d, *J* = 6.9 Hz, 2H), 7.11–7.18 (m, 5H), 7.28–7.42 (m, 8H), 7.66 (d, *J* = 7.2 Hz, 2H), 7.91 (d, *J* = 8.5 Hz, 2H); ^{13}C NMR (100 MHz, $CDCl_3$) δ 27.9, 40.0 (CH_2), 44.8, 70.8, 81.4 (*C*(CH_3)$_3$), 126.7, 127.5, 128.1, 128.2, 128.4, 128.5, 128.80, 128.82, 129.7, 130.4, 135.5 (Cq), 136.2 (Cq), 139.2 (Cq), 139.3 (Cq), 141.2 (Cq), 170.0 (Cq), 171.3 (Cq), 197.6 (C=O); IR/cm^{-1}: 3062 w, 3029 w, 2978 m, 2929 m, 1726 m, 1686 m, 1623 m, 1589 m, 1572 m, 1490 m, 1446 m, 1399 m, 1368 m, 1315 m, 1286 m, 1252 m, 1147 s, 1091 m, 1030 m, 1003 m, 908 m, 844

m, 811 m, 730 s, 694 s; HRMS (ESI) *m/z*: found: 538.2141, calcd for $C_{34}H_{33}NO_3Cl$ $[M+H]^+$: 538.2149; HPLC analysis: Chiralcel OD-H column; hexane/ IPA = 90:10; flow rate = 1.0 mL/min; λ = 254 nm; 18 °C; t_R = 4.75, 5.30 (major) min.

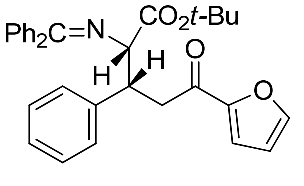

(2*S*,3*R*)-*tert*-Butyl-2-((diphenylmethylene)amino)-5-oxo-3-phenyl-5-(2'-furyl)pentanoate, 14l. Colorless oil; yield 87%; R_f = 0.18 (hexane/EA = 9:1); $[\alpha]_D^{18}$ = –93.1 (c 0.96, CH_2Cl_2) at 90%*ee*; ^{1}H NMR (400 MHz, $CDCl_3$) δ 1.31 (s, 9H, *t*-Bu), 3.40 (dd, *J* = 16.3, 4.1 Hz, 1H), 3.58 (dd, *J* = 16.3, 9.8 Hz, 1H), 4.14–4.21 (m, 2H), 6.47–6.48 (m, 1H), 6.73 (d, *J* = 6.8 Hz, 2H), 7.10–7.18 (m, 6H), 7.30–7.42 (m, 6H), 7.52–7.53 (m, 1H), 7.65–7.67 (m, 2H); ^{13}C NMR (100 MHz, $CDCl_3$) δ 27.9, 40.0 (CH_2), 44.7, 70.9, 81.3 (*C*(CH_3)$_3$), 112.1, 117.0, 126.6, 127.6, 128.0, 128.1, 128.2, 128.4, 128.6, 128.9, 130.3, 136.3 (Cq), 139.4 (Cq), 141.1 (Cq), 146.0, 152.9 (Cq), 169.9 (Cq), 171.1 (Cq), 187.8 (C=O); IR/cm^{-1}: 3029 w, 2978 m, 2928 m, 1726 m, 1675 m, 1622 m, 1598 m, 1569 m, 1494 m, 1468 m, 1393 m, 1368 m, 1315 m, 1286 m, 1258 m, 1228 m, 1148 s, 1080 m, 1026 m, 909 m, 883 m, 846 m, 762 m, 730 m, 696 s; HRMS (ESI) *m/z*: found: 494.2318, calcd for $C_{32}H_{32}NO_4$ $[M+H]^+$: 494.2331; HPLC analysis: Chiralcel OD-H column; hexane/ IPA = 90:10; flow rate = 1.0 mL/min; λ = 254 nm; 18 °C; t_R = 6.12, 8.55 (major) min.

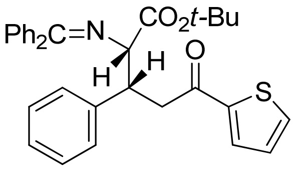

(2*S*,3*R*)-*tert*-Butyl-2-((diphenylmethylene)amino)-5-oxo-3-phenyl-5-(2'-thienyl)pentanoate, 14m. Colorless oil; yield 93%; R_f = 0.14 (hexane/EA = 9:1); $[\alpha]_D^{18}$ = –93.0 (c 1.23, CH_2Cl_2) at 85%*ee*; ^{1}H NMR (400 MHz, $CDCl_3$) δ 1.32 (s, 9H, *t*-Bu), 3.52 (dd, *J* = 16.3, 3.6 Hz, 1H), 3.67 (dd, *J* = 16.3, 9.7 Hz, 1H), 4.15–4.21 (m, 2H), 6.70 (d, *J* = 6.8 Hz, 2H), 7.10–7.18 (m, 6H), 7.29–7.43 (m, 6H), 7.57 (d, *J* = 4.8 Hz, 1H), 7.67 (d, *J* = 7.2 Hz, 2H), 7.82 (d, *J* = 3.2 Hz, 1H); ^{13}C NMR (100 MHz, $CDCl_3$) δ 27.9, 40.8 (CH_2), 45.0, 70.9, 81.4 (*C*(CH_3)$_3$), 126.7, 127.5, 127.98, 128.05, 128.16, 128.22, 128.4, 128.6, 128.9, 130.4, 131.9, 133.3, 136.3 (Cq), 139.4 (Cq), 141.1 (Cq), 144.6 (Cq), 170.0 (Cq), 171.2 (Cq), 191.6 (C=O); IR/cm^{-1}: 3029, 2978 w, 2928 w, 1726 m, 1662 m, 1623 m, 1598 m, 1576 m, 1518 m, 1493 m, 1446 m, 1415 m, 1368 m, 1287 m, 1251 m, 1146 s, 1081 m, 973 m, 942 m, 909 m, 727 s, 695 s; HRMS (ESI) *m/z*: found: 510.2107, calcd for $C_{32}H_{32}NO_3S$ $[M+H]^+$: 510.2103; HPLC analysis: Chiralcel OD-H column; hexane/ IPA = 90:10; flow rate = 1.0 mL/min; λ = 254 nm; 18 °C; t_R = 5.28, 7.45 (major) min.

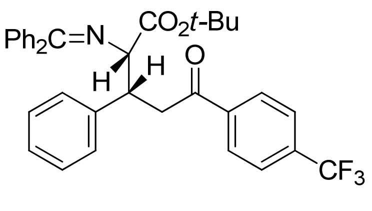

(2*S*,3*R*)-*tert*-Butyl-2-((diphenylmethylene)amino)-5-oxo-3-phenyl-5-(*p*-trifluoromethylphenyl)pentanoate, 14n. Colorless oil; yield 90%; R_f = 0.31 (hexane/EA = 9:1); $[\alpha]_D^{18}$ = –65.1 (c 0.96, CH_2Cl_2) at 83%*ee*; ^1H NMR (400 MHz, $CDCl_3$) δ 1.32 (s, 9H, *t*-Bu), 3.64–3.79 (m, 2H), 4.09–4.24 (m, 2H), 6.68 (d, J = 6.9 Hz, 2H), 7.11–7.20 (m, 5H), 7.26–7.43 (m, 7H), 7.66 (d, J = 7.3 Hz, 2H), 7.70 (d, J = 8.2 Hz, 2H), 8.06 (d, J = 8.2 Hz, 2H); ^{13}C NMR (100 MHz, $CDCl_3$) δ 27.9, 40.3 (CH_2), 44.7, 70.7, 81.5 ($C(CH_3)_3$), 123.7 (d, $^1J_{C,F}$ = 273.7 Hz), 125.60 (q, $^3J_{C,F}$ = 3.1 Hz), 126.8, 127.4, 128.1, 128.2, 128.4, 128.51, 128.54, 128.8, 130.4, 134.1 (d, $^2J_{C,F}$ = 33.9 Hz), 136.2 (Cq), 139.3 (Cq), 139.8 (Cq), 141.1 (Cq), 170.0 (Cq), 171.4 (Cq), 197.9 (C=O); ^{19}F {^1H} NMR (377 MHz, $CDCl_3$) δ –63.0; IR/cm^{-1}: 3062 w, 2978 m, 2930 m, 1726 m, 1692 m, 1622 m, 1598 m, 1578 m, 1493 m, 1447 m, 1410 m, 1367 m, 1321 s, 1288 m, 1254 m, 1148 s, 1129 s, 1110 m, 1066 m, 1028 m, 1006 m, 909 m, 846 m, 731 m, 696 s; HRMS (ESI) *m/z*: found: 572.2415, calcd for $C_{35}H_{33}NO_3F_3$ $[M+H]^+$: 572.2413; HPLC analysis: Chiralcel OD-H column; hexane/ IPA = 95:5; flow rate = 0.5 mL/min; λ = 254 nm; 18 °C; t_R = 11.79, 12.79 (major) min.

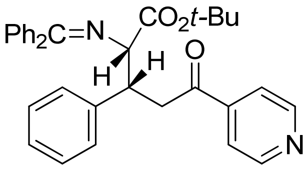

(2*S*,3*R*)-*tert*-Butyl-2-((diphenylmethylene)amino)-5-oxo-3-phenyl-5-(4-pyridyl)pentanoate, 14o. Colorless oil; yield 96%; R_f = 0.28 (hexane/EA = 1:1); $[\alpha]_D^{18}$ = –78.1 (c 1.0, CH_2Cl_2) at 88%*ee*; ^1H NMR (400 MHz, $CDCl_3$) δ 1.32 (s, 9H, *t*-Bu), 3.68–3.71 (m, 2H), 4.13–4.18 (m, 2H), 6.67 (d, J = 7.0 Hz, 2H), 7.10–7.46 (m, 11H), 7.65–7.67 (m, 2H), 7.72–7.73 (m, 2H), 8.77–8.78 (m, 2H); ^{13}C NMR (100 MHz, $CDCl_3$) δ 27.9, 40.4 (CH_2), 44.6, 70.6, 81.5 ($C(CH_3)_3$), 121.2, 126.8, 127.4, 128.1, 128.2, 128.3, 128.4, 128.5, 128.8, 130.5, 136.2 (Cq), 139.2 (Cq), 140.9 (Cq), 142.9 (Cq), 150.9, 169.9 (Cq), 171.5 (Cq), 198.4 (C=O); IR/cm^{-1}: 3060 w, 3029 w, 2978 m, 2931 w, 1726 m, 1697 m, 1621 m, 1597 m, 1576 m, 1557 m, 1448 m, 1408 m, 1368 m, 1336 m, 1315 m, 1286 m, 1253 m, 1219 m, 1148 s, 1082 m, 1064 m, 1030 m, 1012 m, 910 m, 845 m, 805 m, 780 m, 763 m, 731 m, 696 s; HRMS (ESI) *m/z*: found: 505.2475, calcd for $C_{33}H_{33}N_2O_3$ $[M+H]^+$: 505.2491; HPLC analysis: Chiralcel OD-H column; hexane/ IPA = 95:5; flow rate = 1.0 mL/min; λ = 254 nm; 18 °C; t_R = 13.14, 15.11 (major) min.

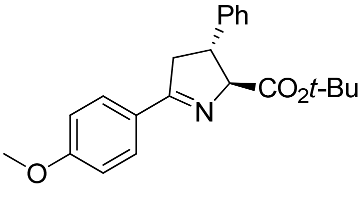

(2*S*,3*R*)-*tert*-Butyl-5-(4-methoxyphenyl)-3-phenyl-3,4-dihydro-2H-pyrrole-2-carboxylate, 15a. *tert*-Butyl glycinate benzophenone Schiff base **1a** (30.0 mg, 0.10 mmol, 1.0 equiv.), **5c** (1.03 mg, 2.0 μmol, 2 mol%) and Cs_2CO_3 (166 mg, 0.50 mmol, 5.0 equiv.) were placed in a Schlenk tube; dry mesitylene (1.0 mL) was added and the resulting suspension was stirred at –20 °C

for 10 min followed by the addition of the chalcone **13p** (24.8 mg, 0.104 mmol, 1.02 equiv). The reaction mixture was effectively stirred (1000 rpm) at –20 °C and monitored by TLC (hexane/ ethyl acetate = 10:1). After 5 h upon complete consumption of **1a** the solvent was removed in high vacuum (0.05 mmHg). The residue was dissolved in dry THF (2 mL), cooled down to 0 °C and 1N aq. HCl (1 mL) was added dropwise. After 1.5 h stirring at 0 °C the reaction mixture was quenched with solid $NaHCO_3$ to pH = 7–8, extracted with CH_2Cl_2 (3×5 mL), the combined organic phases were washed with brine (3 mL), dried over $MgSO_4$, concentrated *in vacuo* and purified by column chromatography on silica gel (hexane/EA = 4:1) to afford the desired product **15a** (30.1 mg, 84%) as clear viscous oil. R_f = 0.23 (hexane/EA = 4:1); $[\alpha]_D^{20}$ = +28.6 (c 1.15, CH_2Cl_2) at 76%*ee*; ^{1}H NMR (400 MHz, $CDCl_3$) δ 1.47 (s, 9H, *t*-Bu), 3.12 (ddd, *J* = 17.0, 6.1, 1.6 Hz, 1H), 3.59 (ddd, *J* = 17.0, 9.6, 1.6 Hz, 1H), 3.73 (dt, *J* = 9.6, 6.1 Hz, 1H), 3.86 (s, 3H, OMe), 4.81 (dt, *J* = 6.1, 1.6 Hz, 1H), 6.92–6.95 (m, 2H), 7.22–7.26 (m, 3H), 7.30–7.33 (m, 2H), 7.85–7.89 (m, 2H); ^{13}C NMR (100 MHz, $CDCl_3$) δ 28.1 , 44.6 (CH_2), 47.1, 55.4, 81.4 (Cq), 83.2, 113.8, 126.6 (Cq), 126.8, 127.0, 128.8, 129.8, 143.8 (Cq), 161.9 (Cq), 171.8 (Cq), 174.0 (Cq); IR/cm^{-1}: 2976 m, 2963 m, 1725 s, 1606 s, 1573 m, 1512 m, 1497 m, 1419 m, 1392 m, 1366 m, 1348 m, 1332 m, 1312 m, 1249 s, 1212 m, 1172 m, 1148 s, 1110 m, 1042 m, 1028 m, 834 s, 813 m, 801 m, 793 m, 756 m; HRMS (ESI) *m/z*: found: 352.1899, calcd for $C_{22}H_{26}NO_3$ $[M+H]^+$: 352.1913; HPLC analysis: Chiralpak AD-H column; hexane/ IPA = 90:10; flow rate = 1.0 mL/min; λ = 254 nm; 18 °C; t_R = 17.42 (major), 22.01 min.

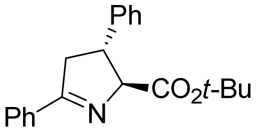

Gram-scale experiment with low catalyst loading:

(2*S*,3*R*)-*tert*-Butyl-3,5-diphenyl-3,4-dihydro-2H-pyrrole-2-carboxylate, 15b. *tert*-Butyl glycinate benzophenone Schiff base **1a** (1.00 g, 3.39 mmol, 1.0 equiv.), **5c** (8.60 mg, 0.017 mmol, 0.5 mol%) and Cs_2CO_3 (5.52 g, 17.0 mmol, 5.0 equiv.) were placed in a Schlenk tube; dry mesitylene (33 mL) was added and the resulting suspension was stirred at –20 °C for 10 min followed by the addition of the diphenylchalcone **13a** (0.72 g, 3.46 mmol, 1.02 equiv). The reaction mixture was effectively stirred (1000 rpm) at –20 °C and monitored by TLC (hexane/ ethyl acetate = 10:1). After 9 h upon complete consumption of **1a**, the reaction mixture was immediately filtered through a glass filter, the solid residue was washed with hexane (10 mL); the combined solvent was removed in high vacuum (0.05 mmHg). The residue was dissolved in dry THF (25 mL), cooled down to 0 °C and 1N aq. HCl (25 mL) was added dropwise. After 2 h stirring at 0 °C the reaction mixture was quenched with solid $NaHCO_3$ to pH = 7–8, extracted with CH_2Cl_2 (3×20 mL), the combined organic phases were washed with brine (10 mL), dried over $MgSO_4$, concentrated *in vacuo* and purified by column chromatography on silica gel (hexane/ ethyl acetate = 8:1) to afford the desired product **15b** (0.97 g, 89%) as white powder. R_f = 0.25 (hexane/EA = 8:1); mp = 68–69 °C; $[\alpha]_D^{20}$ = +51.3 (c 1.10, CH_2Cl_2) at 90%*ee*; ^{1}H NMR (400 MHz, $CDCl_3$) δ 1.47 (s, 9H, *t*-Bu), 3.15 (ddd,

J = 17.2, 6.1, 1.6 Hz, 1H), 3.62 (ddd, J = 17.2, 9.4, 1.6 Hz, 1H), 3.76 (dt, J = 9.4, 6.1 Hz, 1H), 4.85 (dt, J = 6.1, 1.6 Hz, 1H), 7.22–7.26 (m, 3H), 7.30–7.34 (m, 2H), 7.40–7.50 (m, 3H), 7.91–7.93 (m, 2H); ^{13}C NMR (100 MHz, $CDCl_3$) δ 28.1, 44.6 (CH_2), 47.0, 81.5 (Cq), 83.3, 126.8, 127.0, 128.1, 128.5, 128.8, 131.0, 133.8 (Cq), 143.6 (Cq), 171.5 (Cq), 174.7 (Cq); IR/cm^{-1}: 3008 w, 2983 m, 2932 m, 1722 s, 1683 m, 1614 m, 1576 m, 1494 m, 1474 m, 1448 m, 1393 m, 1367 m, 1359 m, 1342 m, 1248 s, 1221 m, 1197 m, 1145 s, 1079 m, 1051 m, 1026 m, 976 m, 924 m, 854 m, 841 m, 799 m, 758 s, 706 s; HRMS (ESI) *m/z*: found: 322.1783, calcd for $C_{21}H_{24}NO_2$ $[M+H]^+$: 322.1807; HPLC analysis: Chiralpak AD-H column; hexane/ IPA = 90:10; flow rate = 1.0 mL/min; λ = 254 nm; 18 °C; t_R = 7.73 (major), 9.57 min.

Single recrystallization of the obtained product (0.97 g) from hexane (25 mL) gave **17** (0.82 g) from mother liquid with 98%*ee* as white foam; $[\alpha]_D^{20}$ = +58.0 (c 1.12, CH_2Cl_2).

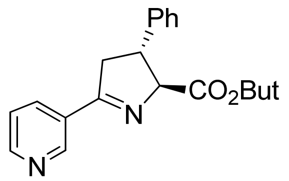

(2*S*,3*R*)-*tert*-Butyl-3-phenyl-5-(pyridin-3-yl)-3,4-dihydro-2H-pyrrole-2-carboxylate, 15c. *tert*-Butyl glycinate benzophenone Schiff base **1a** (60.0 mg, 0.20 mmol, 1.0 equiv.), **5c** (2.06 mg, 4.0 μmol, 2 mol%) and Cs_2CO_3 (331 mg, 1.02 mmol, 5.0 equiv.) were placed in a Schlenk tube; dry mesitylene (2.0 mL) was added and the resulting suspension was stirred at –20 °C for 10 min followed by the addition of the chalcone **13q** (43.4 mg, 0.207 mmol, 1.02 equiv). The reaction mixture was effectively stirred (1000 rpm) at –20 °C and monitored by TLC (hexane/ ethyl acetate = 10:1). After 3 h upon complete consumption of **1a** the solvent was removed in high vacuum (0.05 mmHg). The residue was dissolved in dry THF (3 mL), cooled down to 0 °C and 1N aq. HCl (1.5 mL) was added dropwise. After 1.5 h stirring at 0 °C the reaction mixture was quenched with solid $NaHCO_3$ to pH = 7–8, extracted with CH_2Cl_2 (3×7 mL), the combined organic phases were washed with brine (5 mL), dried over $MgSO_4$, concentrated *in vacuo* and purified by column chromatography on silica gel (hexane/EA = 1:2) to afford the desired product **15c** (62.7 mg, 96%) as clear viscous oil; R_f = 0.28 (hexane/EA = 1:2); $[\alpha]_D^{25}$ = +53.4 (c 1.16, CH_2Cl_2) at 92%*ee*; ^{1}H NMR (400 MHz, $CDCl_3$) δ 1.48 (s, 9H, *t*-Bu), 3.16 (ddd, J = 17.2, 6.4, 1.9 Hz, 1H), 3.65 (ddd, J = 17.2, 9.6, 1.9 Hz, 1H), 3.80 (dt, J = 9.6, 6.4 Hz, 1H), 4.87 (dt, J = 6.4, 1.9 Hz, 1H), 7.22–7.28 (m, 3H), 7.32–7.40 (m, 3H), 8.30 (dt, J = 7.9, 1.9 Hz, 1H), 8.71 (dd, J = 4.9, 1.9 Hz, 1H), 9.04 (d, J = 1.9 Hz, 1H); ^{13}C NMR (100 MHz, $CDCl_3$) δ 28.1, 44.4 (CH_2), 46.9, 81.8 (Cq), 83.3, 123.5, 126.98, 127.01, 128.9, 129.5 (Cq), 135.2, 143.1 (Cq), 149.4, 151.9, 171.2 (Cq), 172.6 (Cq); IR/cm^{-1}: 3031 w, 2978 m, 2930 w, 1728 s, 1617 m, 1590 m, 1568 m, 1496 m, 1476 m, 1456 m, 1428 m, 1413 m, 1392 m, 1367 m, 1344 m, 1283 m, 1247 m, 1227 m, 1149 s, 1069 m, 1025 m, 966 m, 845 m, 808 m, 760 m, 731 m, 700 s; HRMS (ESI) *m/z*: found: 323.1764, calcd for

$C_{20}H_{23}N_2O_2$ [M+H]$^+$: 323.1760; HPLC analysis: Chiralpak AD-H column; hexane/ IPA = 90:10; flow rate = 1.0 mL/min; λ = 254 nm; 18 °C; t_R = 12.55, 16.08 (major) min.

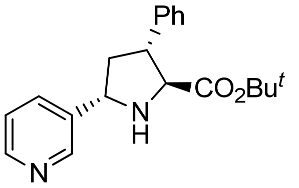

(2*S*,3*R*,5*S*)-*tert*-Butyl-3-phenyl-5-(pyridin-3-yl)pyrrolidine-2-carboxylate, 16. A solution of imine **15c** (59.0 mg, 0.18 mmol) in dry MeOH (2 mL) was cooled down to 0 °C, whereupon sodium borohydride (3×21 mg, 1.65 mmol) was added over 10 min, and the mixture was stirred at r.t. for 24 h. Upon completion (TLC), the mixture was concentrated *in vacuo*, diluted with water (2 mL) and extracted with CH_2Cl_2 (3×10 mL). The combined organic phases were washed with brine (5 mL), dried over $MgSO_4$ and concentrated *in vacuo*. The residue was purified by column chromatography (hexane/ ethyl acetate = 1:2) to afford **16** (48.5 mg, 82%) as white powder. R_f = 0.19 (hexane/EA = 1:2); mp = 79–80 °C; $[\alpha]_D^{20}$ = +2.9 (c 1.36, CH_2Cl_2) at 94%*ee*; ^{1}H NMR (500 MHz, $CDCl_3$) δ 1.39 (s, 9H, *t*-Bu), 1.97 (dt, *J* = 12.5, 10.8 Hz, 1H), 2.61 (ddd, *J* = 12.5, 7.4, 5.7 Hz, 1H), 2.80 (br s, 1H, NH), 3.39 (dt, *J* = 10.8, 7.4 Hz, 1H), 3.90 (d, *J* = 7.4 Hz, 1H), 4.54 (dd, *J* = 10.8, 5.7 Hz, 1H), 7.22–7.28 (m, 2H), 7.31–7.35 (m, 4H), 7.82–7.84 (m, 1H), 8.50 (dd, *J* = 4.8, 1.7 Hz, 1H), 8.67 (d, *J* = 2.2 Hz, 1H); ^{13}C NMR (125 MHz, $CDCl_3$) δ 28.0, 45.0 (CH_2), 51.0, 60.0, 67.7, 81.4 (Cq), 123.4, 126.8, 127.5, 128.5, 134.0, 139.4 (Cq), 142.3 (Cq), 148.55, 148.6, 174.1 (Cq); IR/cm^{-1}: 3332 m, 2991 m, 2966 m, 2930 w, 2902 m, 2836 w, 1725 s, 1579 m, 1495 m, 1478 m, 1457 m, 1428 m, 1392 m, 1367 m, 1341 m, 1315 m, 1297 m, 1254 m, 1220 m, 1152 s, 1123 s, 1074 m, 1021 m, 906 m, 846 m, 817 m, 799 m, 763 s, 745 m, 719 s, 699 s; HRMS (ESI) *m/z*: found: 325.1928, calcd for $C_{20}H_{25}N_2O_2$ [M+H]$^+$: 325.1916; HPLC analysis: Chiralpak AD-H column; hexane/IPA = 90:10; flow rate = 1.0 mL/min; λ = 254 nm; 18 °C; t_R = 13.72, 17.09 (major) min.

Determination of the absolute configuration of adducts 12 and 14

The absolute configuration of products **12** was established on the basis of the comparison of the literature data with the experimental values:

This work

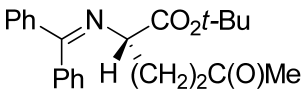

(*S*)-

$[\alpha]_D^{26}$ = –72.6 (c 0.91, $CHCl_3$) at 93%*ee*

HPLC analysis:
Chiralcel OD-H column
(hexane/ IPA = 95:5; 1.0 mL/min; 254 nm)
t_R = 6.7 (min), 7.4 (major) min

Literature[20]

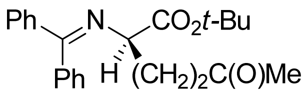

(*R*)-

$[\alpha]_D^{29}$ = +64.2 (c 1.30, $CHCl_3$) at 91%*ee*

HPLC analysis:
Chiralcel OD-H column
(hexane/ IPA = 92:8; 0.8 mL/min; 230 nm)
t_R = 6.5 (major), 7.2 (min) min

The absolute configuration of products **14** was established on the basis of the comparison of the literature data with the experimental values:

This work

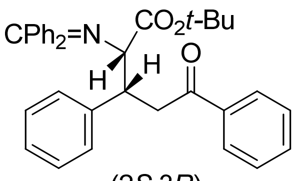

(2*S*,3*R*)-

$[\alpha]_D^{18}$ = –81.7 (c 1.05, CH_2Cl_2) at 93%*ee*

HPLC analysis:
Chiralpak AD-H column
(hexane/ IPA = 90:10; 1.0 mL/min; 254 nm)
t_R = 6.9 (min), 8.8 (major) min;

Literature[21]

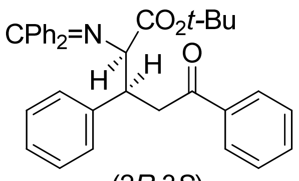

(2*R*,3*S*)-

$[\alpha]_D^{20}$ = +81.7 (*c* 1.0, CH_2Cl_2) at 93%*ee*

HPLC analysis:
Chiralpak AD-H column
(hexane/ IPA = 95:5; 0.5 ml/min; 254 nm)
t_R = 21.8 (major), 30.3 (min) min

Confirmation of the relative configuration of compound 15c by NOESY (500 MHz, $CDCl_3$, 23°C)

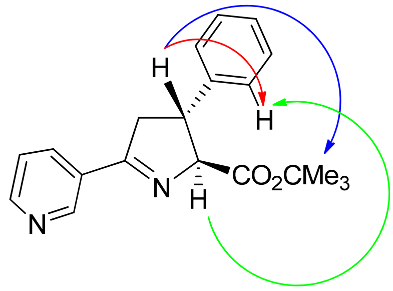

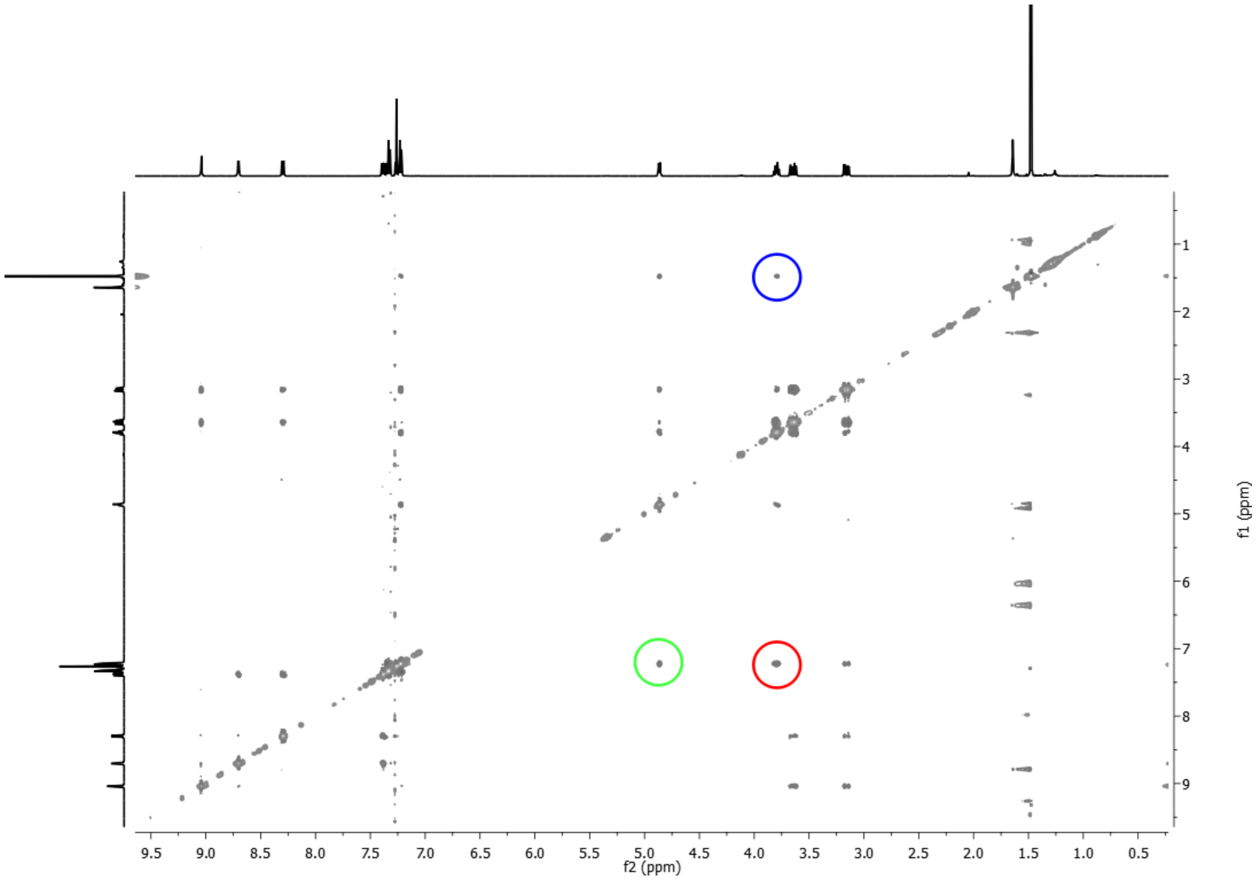

Confirmation of the relative configuration of compound 16 by NOESY (500 MHz, $CDCl_3$, 23 °C)

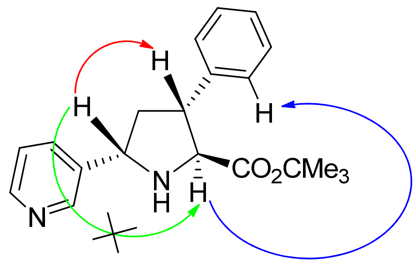

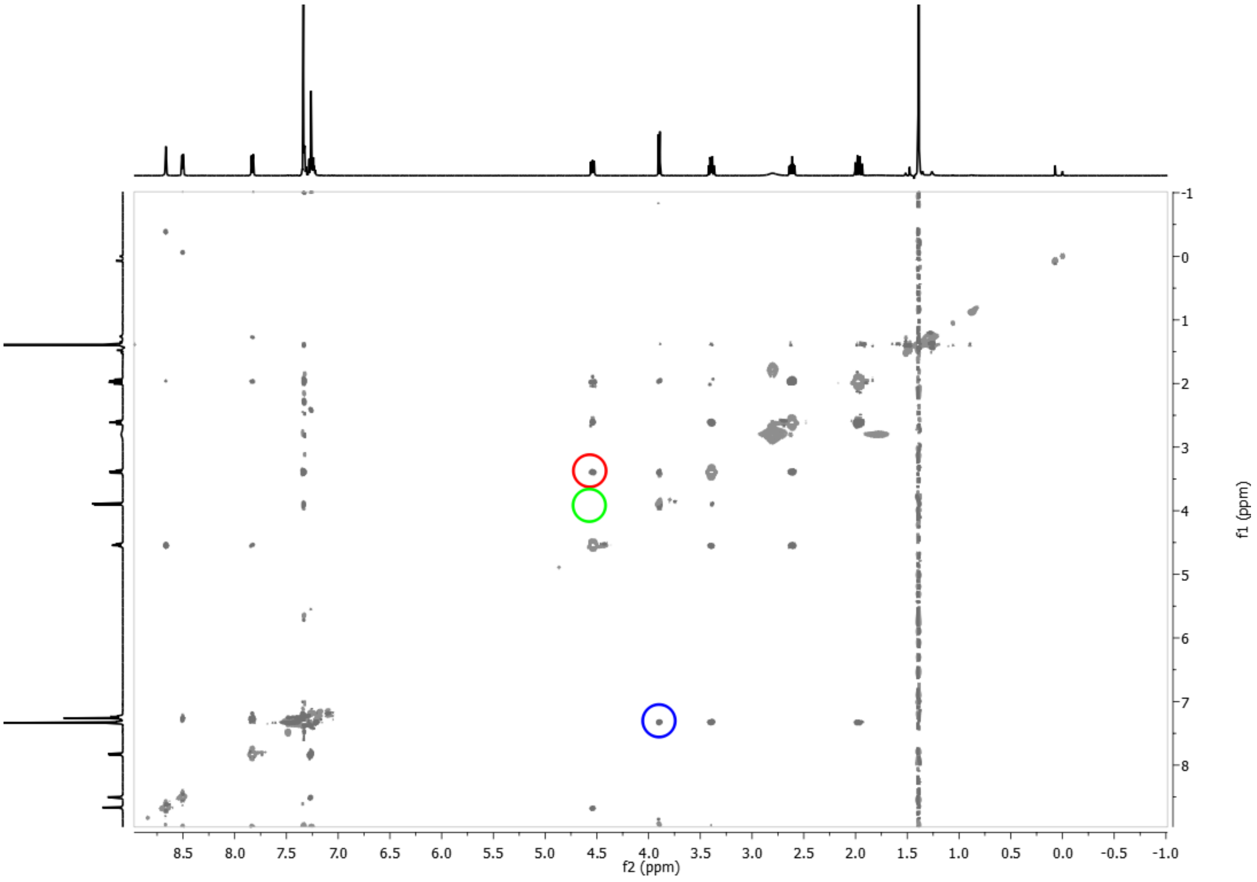

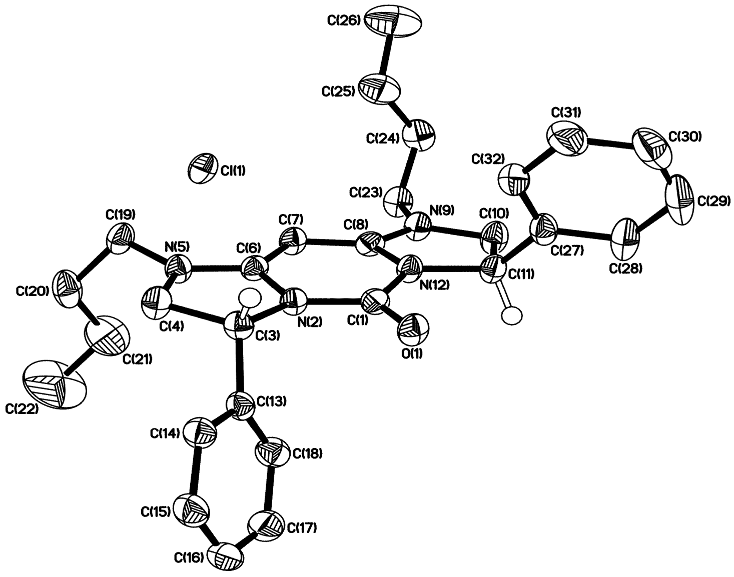

Crystal structure for (*R*,*R*)-**5b** (50% probability ellipsoids).

Crystal data for (*R*,*R*)-**5b**: $[C_{28}H_{35}N_4O](Cl)\cdot 2(C_2H_3N)$, *M* = 561.16, triclinic, *P*1 (no. 1), *a* = 8.7894(2), *b* = 8.7918(3), *c* = 10.9541(3) Å, α = 76.466(3), β = 73.128(3), γ = 78.647(2)°, *V* = 779.95(4) Å^3, *Z* = 1, ρ_{calcd} = 1.195 g cm^{-3}, $\mu(Cu_{K\alpha})$ = 1.344 mm^{-1}, *T* = 173 K, colourless blocks, Oxford Diffraction Xcalibur PX Ultra diffractometer; 5892 independent measured reflections (R_{int} = 0.0220), F^2 refinement,[1] R_1(obs) = 0.0266, wR_2(all) = 0.0670, 5755 independent observed absorption-corrected reflections [$|F_o| > 4\sigma(|F_o|)$, $2\theta_{max}$ = 145°], 364 parameters. The absolute structure of (*R*,*R*)-**5b** was determined by a combination of *R*-factor tests [R_1^+ = 0.0266, R_1^- = 0.0564] and by use of the Flack parameter [x^+ = 0.000(7), x^- = 1.021(7)]. CCDC 919960 contains the supplementary crystallographic data for this paper. These data can be obtained free of charge from the Cambridge Crystallographic Data Centre via www.ccdc.cam.ac.uk/data_request/cif.

[1] G.M. Sheldrick, *Acta Cryst.*, **2008**, *A64*, 112-122.

Table S8. Crystal data and structure refinement for **5b**.

Formula	$[C_{28}H_{35}N_4O](Cl) \times 2(C_2H_3N)$	
Molecular weight	561.16	
Temperature	173 K	
Diffractometer	OD Xcalibur PX Ultra	
Wavelength	1.54184 Å	
Crystal system, space group	Triclinic, P1	
Unit cell dimensions	a = 8.7894(2) Å	α = 76.466(3)°
	b = 8.7918(3) Å	β = 73.128(3)°
	c = 10.9541(3) Å	γ = 78.647(2)°
Volume, Z	779.95(4) Å^3, 1	
Density (calculated)	1.195 Mg/m^3	

Absorption coefficient	1.344 mm^{-1}
F(000)	300
Crystal colour / morphology	Colorless blocks
Crystal size	0.32 x 0.18 x 0.14 mm^3
θ range for data collection	5.23 to 72.52°
Index ranges	-10<=h<=10, -10<=k<=10, -13<=l<=13
Reflns collected / unique	16855 / 5892 [R(int) = 0.0220]
Reflns observed [F>4σ(F)]	5755
Absorption correction	Analytical
Max. and min. transmission	0.840 and 0.743
Refinement method	Full-matrix least-squares on F^2
Data / restraints / parameters	5892 / 3 / 364
Goodness-of-fit on F^2	1.048
Final R indices [F>4σ(F)]	R1 = 0.0266, wR2 = 0.0662<br>R1+ = 0.0266, wR2+ = 0.0662<br>R1- = 0.0564, wR2- = 0.1402
R indices (all data)	R1 = 0.0275, wR2 = 0.0670
Absolute structure parameter	x+ = 0.000(7), x- = 1.021(7)
Extinction coefficient	0.0303(12)
Largest diff. peak, hole	0.125, -0.128 eÅ^{-3}
Mean and maximum shift/error	0.000 and 0.000

Table S9. Bond length and angles for 5b.

Bond lengths [Å]	angles [°]	Bond lengths [Å]	angles [°]
O(1)-C(1)	1.2168(16)	N(2)-C(3)-C(4)	101.52(10)
C(1)-N(12)	1.3745(16)	C(13)-C(3)-C(4)	111.23(11)
C(1)-N(2)	1.3774(16)	N(5)-C(4)-C(3)	104.78(10)
N(2)-C(6)	1.3806(16)	C(6)-N(5)-C(4)	112.22(11)
N(2)-C(3)	1.4722(16)	C(6)-N(5)-C(19)	126.47(11)
C(3)-C(13)	1.5086(18)	C(4)-N(5)-C(19)	121.24(11)
C(3)-C(4)	1.5435(18)	N(5)-C(6)-N(2)	109.26(11)
C(4)-N(5)	1.4556(17)	N(5)-C(6)-C(7)	129.31(12)
N(5)-C(6)	1.3296(17)	N(2)-C(6)-C(7)	121.43(12)
N(5)-C(19)	1.4642(17)	C(8)-C(7)-C(6)	115.31(11)
C(6)-C(7)	1.3807(18)	N(9)-C(8)-C(7)	129.20(12)
C(7)-C(8)	1.3786(18)	N(9)-C(8)-N(12)	109.27(11)
C(8)-N(9)	1.3286(17)	C(7)-C(8)-N(12)	121.53(11)
C(8)-N(12)	1.3786(17)	C(8)-N(9)-C(10)	112.48(11)
N(9)-C(10)	1.4512(18)	C(8)-N(9)-C(23)	125.78(11)
N(9)-C(23)	1.4645(17)	C(10)-N(9)-C(23)	121.69(11)
C(10)-C(11)	1.5415(19)	N(9)-C(10)-C(11)	104.67(11)
C(11)-N(12)	1.4747(16)	N(12)-C(11)-C(27)	114.76(11)
C(11)-C(27)	1.511(2)	N(12)-C(11)-C(10)	101.67(10)
C(13)-C(18)	1.389(2)	C(27)-C(11)-C(10)	112.09(12)
C(13)-C(14)	1.3913(19)	C(1)-N(12)-C(8)	123.83(11)
C(14)-C(15)	1.389(2)	C(1)-N(12)-C(11)	123.38(10)
C(15)-C(16)	1.386(3)	C(8)-N(12)-C(11)	111.87(10)
C(16)-C(17)	1.381(2)	C(18)-C(13)-C(14)	119.71(13)
C(17)-C(18)	1.393(2)	C(18)-C(13)-C(3)	123.19(12)
C(19)-C(20)	1.509(2)	C(14)-C(13)-C(3)	116.90(12)
C(20)-C(21)	1.514(3)	C(15)-C(14)-C(13)	120.35(14)
C(21)-C(22)	1.530(3)	C(16)-C(15)-C(14)	119.90(15)
C(23)-C(24)	1.508(2)	C(17)-C(16)-C(15)	119.81(14)
C(24)-C(25)	1.524(3)	C(16)-C(17)-C(18)	120.71(15)
C(25)-C(26)	1.511(3)	C(13)-C(18)-C(17)	119.50(14)
C(27)-C(32)	1.385(2)	N(5)-C(19)-C(20)	112.34(12)
C(27)-C(28)	1.390(2)	C(19)-C(20)-C(21)	112.95(14)
C(28)-C(29)	1.387(3)	C(20)-C(21)-C(22)	112.2(2)
C(29)-C(30)	1.382(3)	N(9)-C(23)-C(24)	112.92(12)
C(30)-C(31)	1.376(3)	C(23)-C(24)-C(25)	112.82(13)
C(31)-C(32)	1.396(2)	C(26)-C(25)-C(24)	112.76(17)
N(40)-C(41)	1.134(2)	C(32)-C(27)-C(28)	119.44(15)
C(41)-C(42)	1.457(3)	C(32)-C(27)-C(11)	123.17(12)
N(50)-C(51)	1.134(2)	C(28)-C(27)-C(11)	117.27(14)
C(51)-C(52)	1.451(3)	C(29)-C(28)-C(27)	120.03(18)
O(1)-C(1)-N(12)	123.24(11)	C(30)-C(29)-C(28)	120.35(16)
O(1)-C(1)-N(2)	123.40(11)	C(31)-C(30)-C(29)	119.97(17)
N(12)-C(1)-N(2)	113.36(10)	C(30)-C(31)-C(32)	120.02(18)
C(1)-N(2)-C(6)	123.69(10)	C(27)-C(32)-C(31)	120.19(15)
C(1)-N(2)-C(3)	122.88(10)	N(40)-C(41)-C(42)	178.94(18)
C(6)-N(2)-C(3)	112.08(10)	N(50)-C(51)-C(52)	178.5(2)
N(2)-C(3)-C(13)	115.17(11)		

Optimization Tables

Optimization of conditions for the addition of vinyl ketones

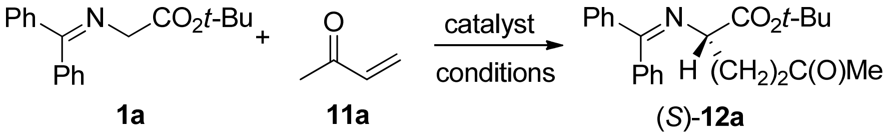

Table S1. Solvent screening.[a]

Entry	Solvent (0.1 M)	Time, min	Conversion,%[b]	%*ee*[c]
1	toluene	65	100	79
2	THF dry	80	100	15
3	CH_2Cl_2 dry	120	100	2
4	MeCN dry	120	100	racemic
5	Et_2O dry	60	100	12
6	o-xylene	45	100	82
7	p-xylene	45	100	78
8	mesitylene	45	100	55
9	$CHCl_3$	45	100	racemic
10	CF_3Ph	65	100	43
11	ClPh	45	100	67
12	EtPh	45	100	61

[a] Reaction was carried out with MVK (2 equiv.), **5a** (1 mol%), Cs_2CO_3 (1.5 equiv.), at r.t. [b] Determined by TLC: hexane/ether 2:1; R_f =0.48. [c] Determined by chiral HPLC: Chiralcel OD-H column; hexane/IPA = 95:5; flow rate = 1.0 mL/min; λ = 254 nm; t_R = 6.68, 7.36 (major) min.

Table S2. Optimization of concentration.[a]

Entry	Concentration, M (in toluene)	Time	Conversion,%[b]	%*ee*[c]
1	0.5	35 min	100	73
2	0.2	35 min	100	78
3	0.1	65 min	100	79
4	0.05	65 min	100	81
5	0.01	20 h	96[d]	49

[a] Reaction was carried out with MVK (2 equiv.), **5a** (1 mol%), Cs_2CO_3 (1.5 equv.), at r.t. [b] Determined by TLC: hexane/ether 2:1; R_f =0.48. [c] Determined by chiral HPLC: Chiralcel OD-H column; hexane/IPA = 95:5; flow rate = 1.0 mL/min; λ = 254 nm; t_R = 6.68, 7.36 (major) min. [d] Determined by ^{1}H NMR spectrum of the crude reaction mixture.

Table S3. Base screening.[a]

Entry	Solvent (0.1 M)	Base (equiv.)	T	Time	Conversion,%[b]	%*ee*[c]
1	toluene	Cs_2CO_3 (1.5)	r.t.	65 min	100	79
2	toluene	Na_2CO_3 (1.5)	r.t.	8 h	20[d]	62
3	toluene	K_2CO_3 (1.5)	r.t.	8 h	65[d]	70
4	toluene	K_3PO_4 (1.5)	r.t.	65	100	82
5	toluene	KF (1.5)	r.t.	8 h	25[d]	71
6	toluene	CsF (1.5)	r.t.	8 h	100	82
7	toluene	Rb_2CO_3 (1.5)	r.t.	2 h min	100	81
8	toluene	$CsOH{\times}H_2O$ (1.5)	r.t.	45 min	100	54
9	toluene	$Ca(OH)_2$ (1.5)	r.t.	8 h	15[d]	35
10	toluene	KOH (50% aq) (1.5)	r.t.	2 h	100	56
11	toluene	NaOH (50% aq) (1.5)	r.t.	45 min	100	76
12	toluene	Cs_2CO_3 (50% aq) (1.5)	r.t.	30 h	23[d]	41
13	o-xylene	K_3PO_4 (1.5)	r.t.	65 min	100	83
14	toluene	K_3PO_4 (1.5)	0 °C	30 h	43[d]	72
15	toluene	Cs_2CO_3 (1.5)	0 °C	4.5 h	100	84
16	o-xylene	Cs_2CO_3 (1.5)	0 °C	4.5 h	97[d]	85
17	toluene	–	r.t.	24 h	no reaction	–

[a] Reaction was carried out with MVK (2 equiv.), **5a** (1 mol%). [b] Determined by TLC: hexane/ether 2:1; R_f =0.48. [c] Determined by chiral HPLC: Chiralcel OD-H column; hexane/IPA = 95:5; flow rate = 1.0 mL/min; λ = 254 nm; t_R = 6.68, 7.36 (major) min. [d] Determined by ^{1}H NMR spectrum of the crude reaction mixture.

Table S4. Catalyst amount screening.[a]

Catalyst 5a loading, mol%	Time, min	Conversion,%[b]	%*ee*[c]
5	65	100	81
2	65	100	80
1	65	100	79
0.5	65	100	75
0.1	65	100	8

[a] Reaction was carried out with MVK (2 equiv.), Cs_2CO_3 (1.5 equiv.), in toluene (0.1 M), at r.t. [b] Determined by TLC: hexane/ether 2:1; R_f =0.48. [c] Determined by chiral HPLCL: Chiralcel OD-H column; hexane/IPA = 95:5; flow rate = 1.0 mL/min; λ = 254 nm; t_R = 6.68, 7.36 (major) min.

Table S5. Base amount and temperature screening.[a]

Entry	Catalyst (2 mol%)	Solvent (0.1 M)	Cs_2CO_3, equiv.	T	Time	Conversion (Yield),%[b]	%*ee*[c]
1	**5a**	toluene	5	r.t.	35 min	100	79
2	**5a**	toluene	3	r.t.	65 min	100	80
3	**5a**	toluene	1.5	r.t.	65 min	100	80
4	**5a**	toluene	0.5	r.t.	65 min	100	81
5	**5a**	toluene	0.1	r.t.	20 h	62[3]	68
6	**5c**	toluene	0.5	10 °C	2 h	100 (79)	92
7	**5c**	toluene	0.5	0 °C	8 h	92[3]	91
8	**5c**	o-xylene	0.5	0 °C	8 h	85[3]	90
9	**5c**	toluene	1.5	10 °C	2 h	100 (75)	92
10	**5c**	toluene	1.5	0 °C	5 h	100 (85)	93
7	**5c**	o-xylene	1.5	0 °C	5 h	100 (79)	93
11	**5c**	toluene	1.5	–20 °C	24 h	97 (76)	93

[a] Reaction was carried out with MVK (2 equiv.). [b] Determined by TLC: hexane/ether 2:1; R_f =0.48. [b] Determined by ^{1}H NMR spectrum of the crude reaction mixture. Value in parenthesis corresponds to isolated yield. [c] Determined by chiral HPLC: Chiralcel OD-H column; hexane/IPA = 95:5; flow rate = 1.0 mL/min; λ = 254 nm; t_R = 6.68, 7.36 (major) min.

Table S6. Optimization of the vinyl ketone amount.[a]

MVK, equiv.	Time	Conversion,%[b]	%*ee*[c]
4.0	35 min	100	78
2.0	65 min	100	81
1.2	2 h	100	78

[a] Reaction was carried out with **5a** (1 mol%), Cs_2CO_3 (1.5 equiv.), in toluene (0.1 M), at r.t. [b] Determined by TLC: hexane/ether 2:1; R_f = 0.48. [c] Determined by chiral HPLC: Chiralcel OD-H column; flow rate = 1.0 mL/min; λ = 254 nm; hexane/IPA = 95:5; t_R = 6.68 (min), 7.36 (major).

Optimization of the reaction conditions for the addition of chalcones

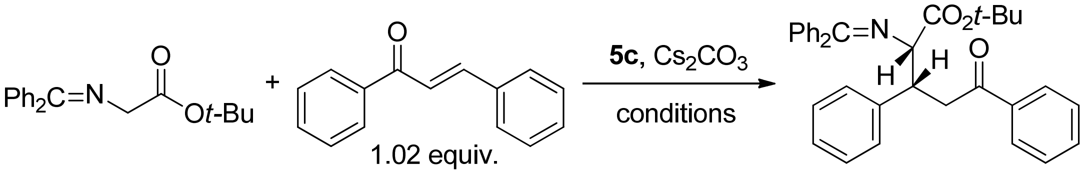

Table S7.

Entry	5c, mol%	Solvent (0.1 M)	Cs_2CO_3, equiv.	T, °C	Time, h	Conversion,%[a]	%*ee*[b]
1	2	toluene	1.5	r.t.	2.5	100	34
2	2	toluene	1.5	0	17	60[c]	80
3	2	toluene	1.5	40	1	100	37
4	2	toluene	2.5	0	10	90[c]	78
5	2	toluene	5.0	0	10	100	77
6	2	toluene	2.5	–20	17	67[c]	79
7	2	toluene	5.0	–20	17	92[c]	85
8	2	o-xylene	5.0	–20	17	72[c]	80
9	2	mesitylene	2.5	–20	10	100	89
10	2	mesitylene	5.0	–20	3	100	93
11	2	mesitylene	5.0	–40	4	100	93
12	5	mesitylene	5.0	–20	3	100	93
13[d]	5	mesitylene	5.0	–20	3	100	93
14	0.5	mesitylene	5.0	–20	9	100	90

[a] Determined by TLC: hexane/ether 2:1; R_f =0.48. [b] Determined by chiral HPLC: CHIRALPK AD-H column; 90% hexane/10% IPA; flow rate = 1.0 mL/min; λ = 254 nm; t_R = 6.9, 8.8 (major) min. [c] Determined by ^{1}H NMR spectrum of the crude reaction mixture. [d] 2.0 equiv. of **13a** was used.

References

1. (*a*) F. Freire, J. D. Fisk, A. J. Peoples, M. Ivancic, I. A. Guzei, S. H. Gellman, *J. Am. Chem. Soc.*, **2008**, *130*, 7839–7841; (*b*) H. Nemoto, H. Moriguchi, R. Ma, T. Kawamura, M. Kamiya, M. Shibuya, *Tetrahedron: Asymmetry*, **2007**, *18*, 383–389.

2. D. M. Shendage, R. Froehlich, G. Haufe, *Org. Lett.,* **2004**, *6*, 3675–3678.

3. C. Toniolo, G. M. Bonora, G. R. Sullivan, W. H. Bearden, J. D. Roberts, *J. Org. Chem.*, **1980**, *45*, 288–290.

4. A. R. Mitchell, S. B. H. Kent, M. Engelhard, Merrifield R. B., *J. Org. Chem.*, **1978**, *43*, 2845–2852.

5. G. L. Khatik, V. N. Kumar, A. Vipin, *Org. Lett.*, **2012**, *14*, 2442–2445.

6. B. Ramalingam, M. Neuburger, A. Pfaltz, *Synthesis*, **2007**, 572–582.

7. Z. L. Shen, K. K. K. Goh, H. L. Cheong, C. H. A. Wong, Y. C. Lai, Y. S. Yang, T. P. Loh, *J. Am. Chem. Soc.*, **2010**, *132*, 15852–15855.

8. J.-i. Matsuo, Y. Aizawa, *Chem. Commun.*, **2005**, 2399–2401.

9. X.-L. An, J.-R. Chen, C.-F. Li, F.-G. Zhang, Y.-Q. Zou, Y.-C. Guo, W.-J. Xiao, *Chem. Asian J.,* **2010**, *5*, 2258–2265.

10. S. Liu, L. S. Liebeskind, *J. Am. Chem. Soc*. **2008**, *130*, 6918–6919.

11. M. Y. Wani, F. Athar, A. R. Bhat, I. Choi, A. Azam, D. H. Lee, *Eur. J. Med. Chem.*, **2012**, *54*, 845–854

12. W.-B. Yi, C. Cai, *J. Fluorine Chem.*, **2009**, *130*, 484–487.

13. A. Stroba, W. Froehner, R. W. Hartmann, M. Engel, F. Schaeffer, V. Hindie, L. Lopez-Garcia, I. Adrian, B. R. M. Iris, *J. Med. Chem.*, **2009**, *52*, 4683–4693.

14. L. D. Chiaradia, A. Mascarello, M. N. S. Cordeiro, R. J. Nunes, R. A. Yunes, M. Purificacao, J. Vernal, M. E. Zenteno, A. Villarino, H. Terenzi, Bioorg. *Med. Chem. Lett.*, **2008**, *18*, 6227–6230.

15. X.-F. Wu, H. Neumann, A. Spannenberg, T. Schulz, H. Jiao, M. Beller, *J. Am. Chem. Soc.*, **2010**, *132*,14596–14602.

16. S. Attar, Z. O'Brien, M. L. Golden, H. Alhaddad, A. Calderon-Urrea, *Bioorg. Med. Chem.*, **2011**, *19*, 2055–2073.

17. M. G. Mamolo, V. Falagiani, L. Vio, E. Banfi, *Il Farmaco*, **1999**, *54*, 761–767.

18. A. Agarwal, K. Srivastava, S. K. Puri, P. M. S. Chauhan, *Bioorg. Med. Chem.*, **2005**, *13*, 6226–6232.

19. V. V. Zakharychev, A. V. Kuzenkov, *Chem. Het. Compd.*, **2007**, *43*, 989–995.

20. T. Ma, X. Fu, C. W. Kee, L. Zong, Y. Pan, C.-H. Tan, K.-W. Huang, *J. Am. Chem. Soc.*, **2011**, *133*, 2828–2831.

21. M.-Q. Hua, L. Wang, H.-F. Cui, J. Nie, X.-L. Zhang, J.-A. Ma, *Chem. Commun.*, **2011**, 1631–1633.

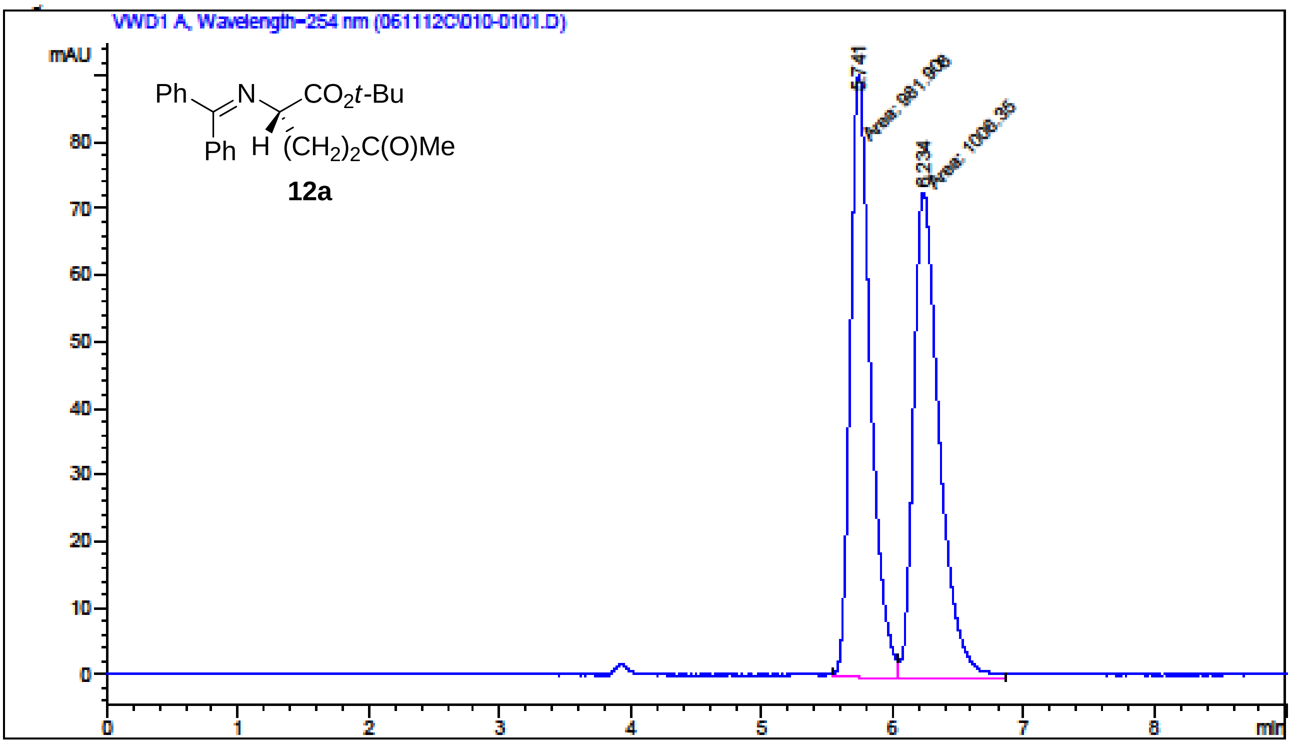

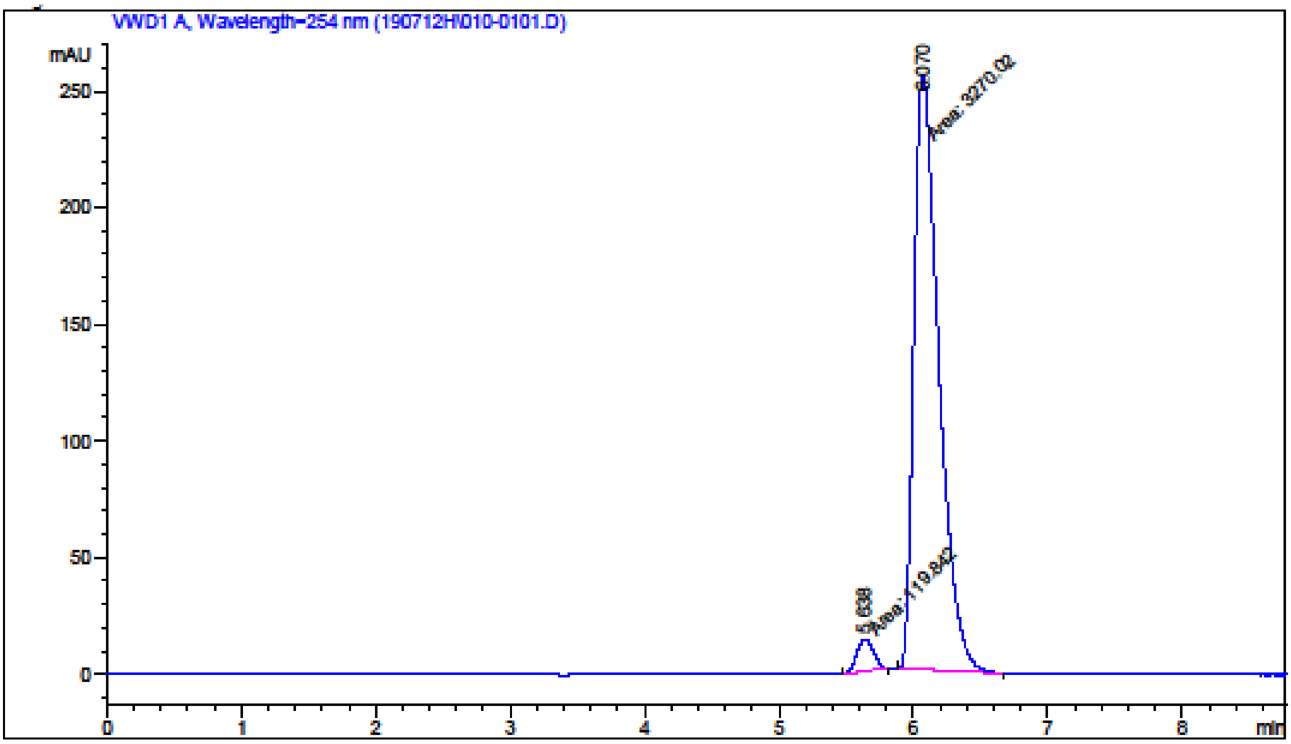

Peak #	RetTime [min]	Type	Width [min]	Area [mAU*s]	Height [mAU]	Area %
1	5.638	MM	0.1420	119.84155	14.06913	3.5353
2	6.070	MM	0.2134	3270.02124	255.37997	96.4647

93% *ee*

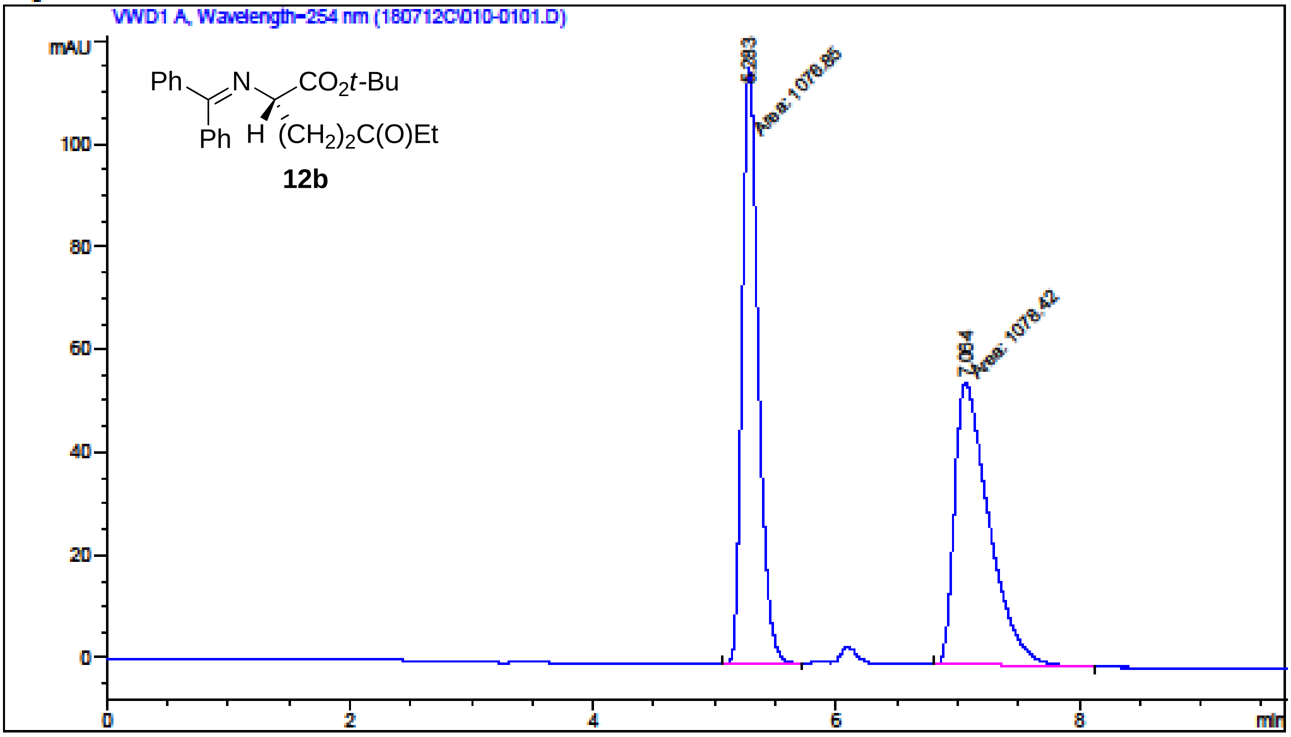

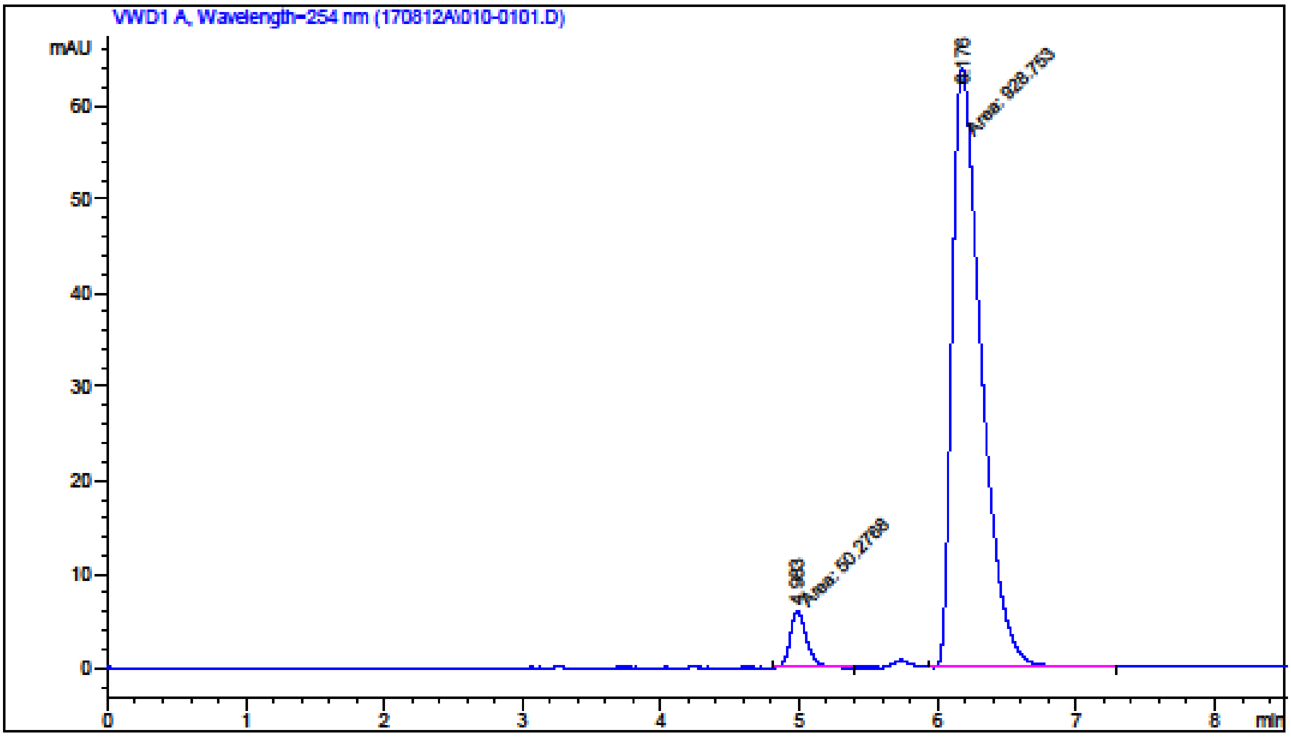

Peak #	RetTime [min]	Type	Width [min]	Area [mAU*s]	Height [mAU]	Area %
1	4.983	MM	0.1369	50.27677	6.12208	5.1354
2	6.176	MM	0.2418	928.75262	64.00349	94.8646

90%*ee*

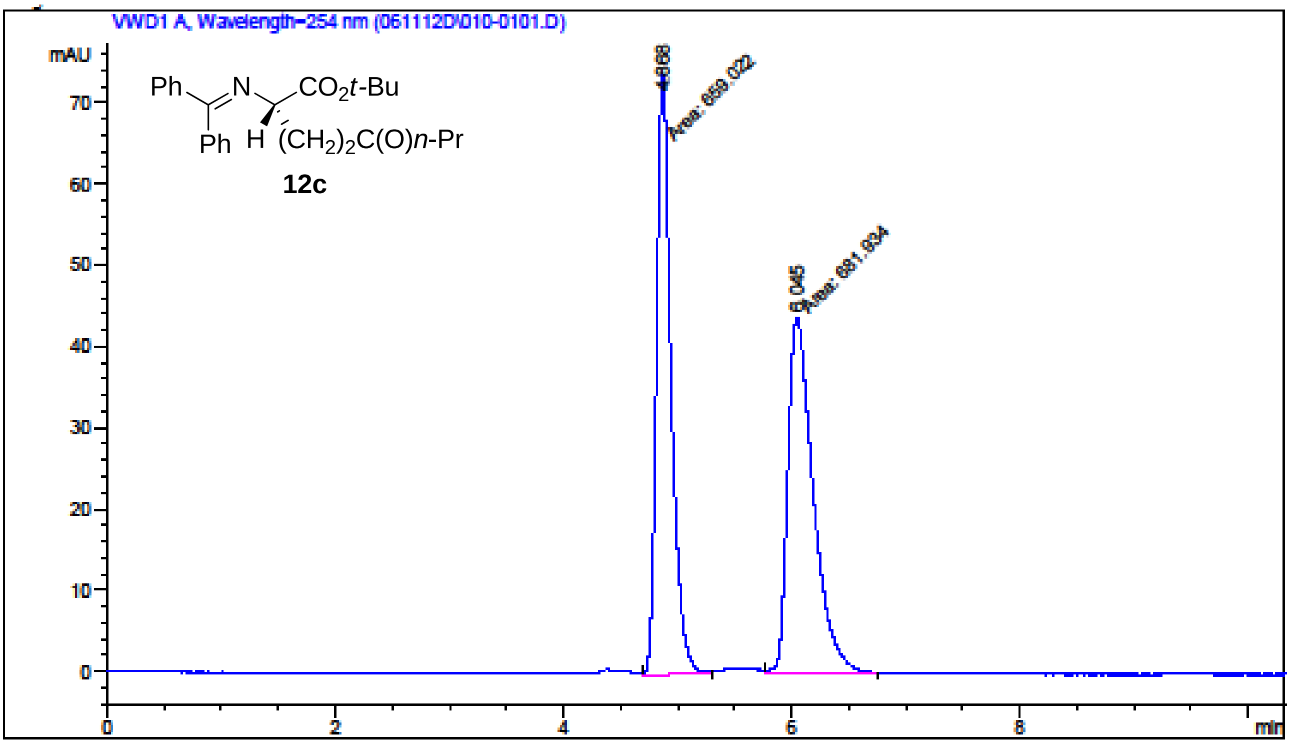

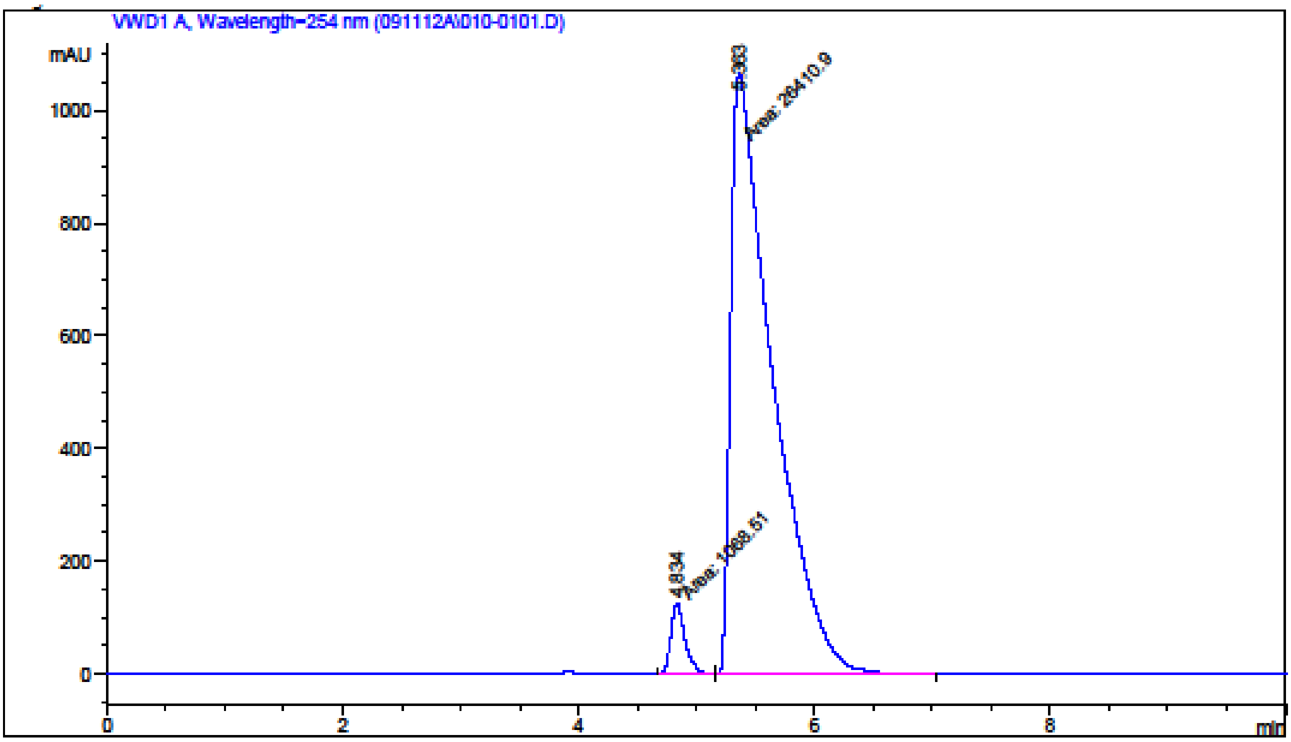

Peak #	RetTime [min]	Type	Width [min]	Area [mAU*s]	Height [mAU]	Area %
1	4.834	MM	0.1416	1068.51013	125.79137	3.8884
2	5.363	MM	0.4129	2.64109e4	1066.08960	96.1116

92%*ee*

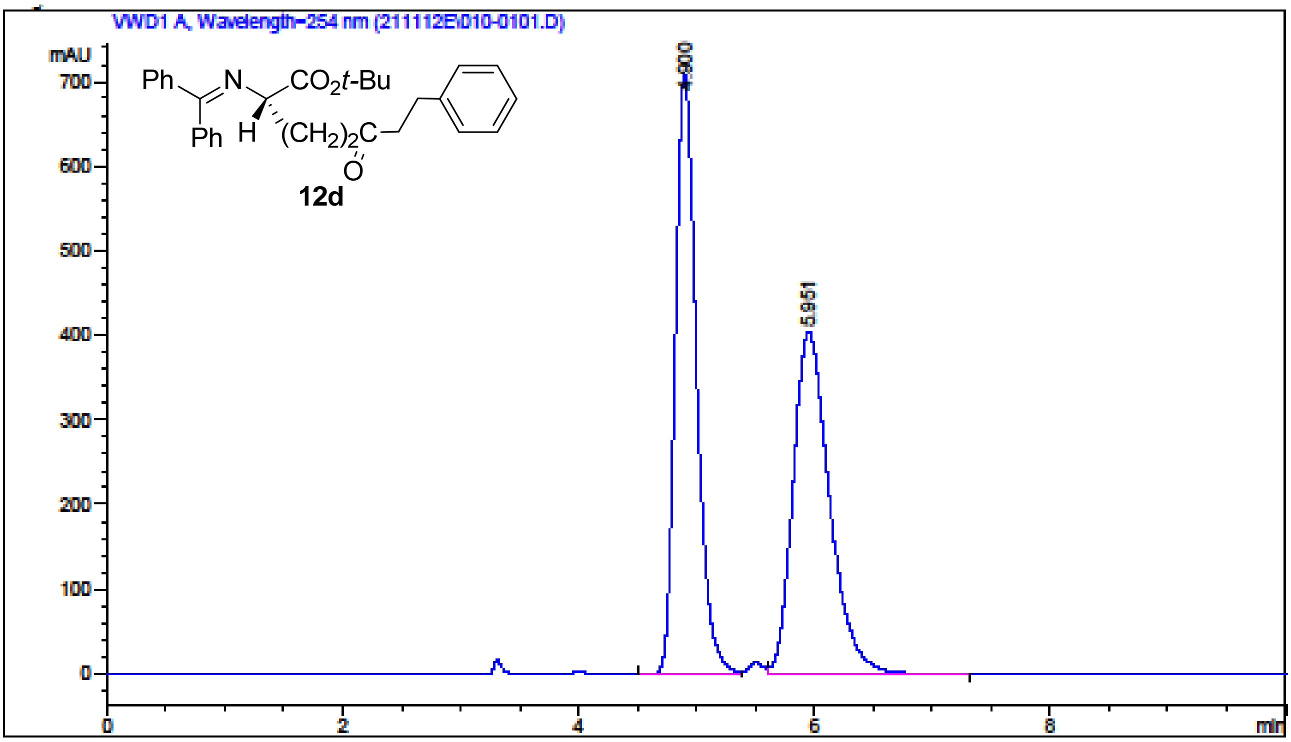

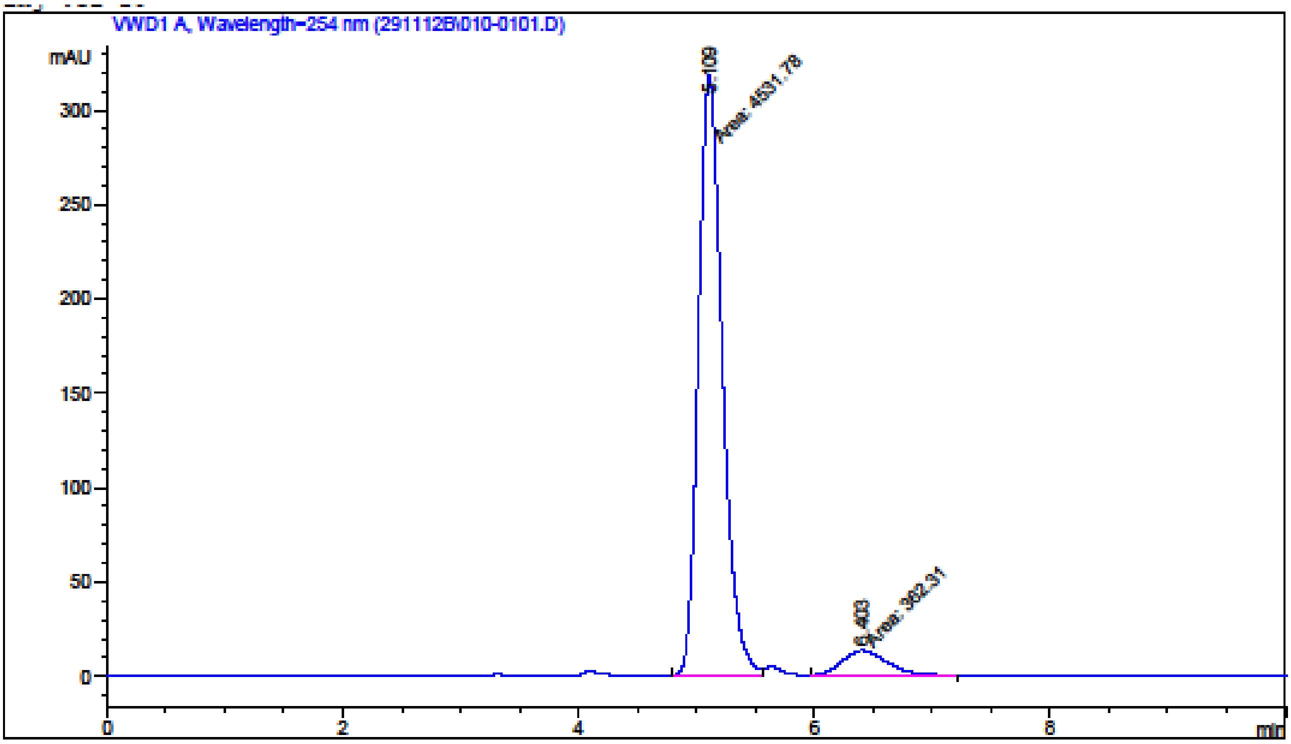

Peak #	RetTime [min]	Type	Width [min]	Area [mAU*s]	Height [mAU]	Area %
1	5.109	MM	0.2377	4531.78223	317.74960	92.5970
2	6.403	MM	0.4544	362.31049	13.28759	7.4030

85%*ee*

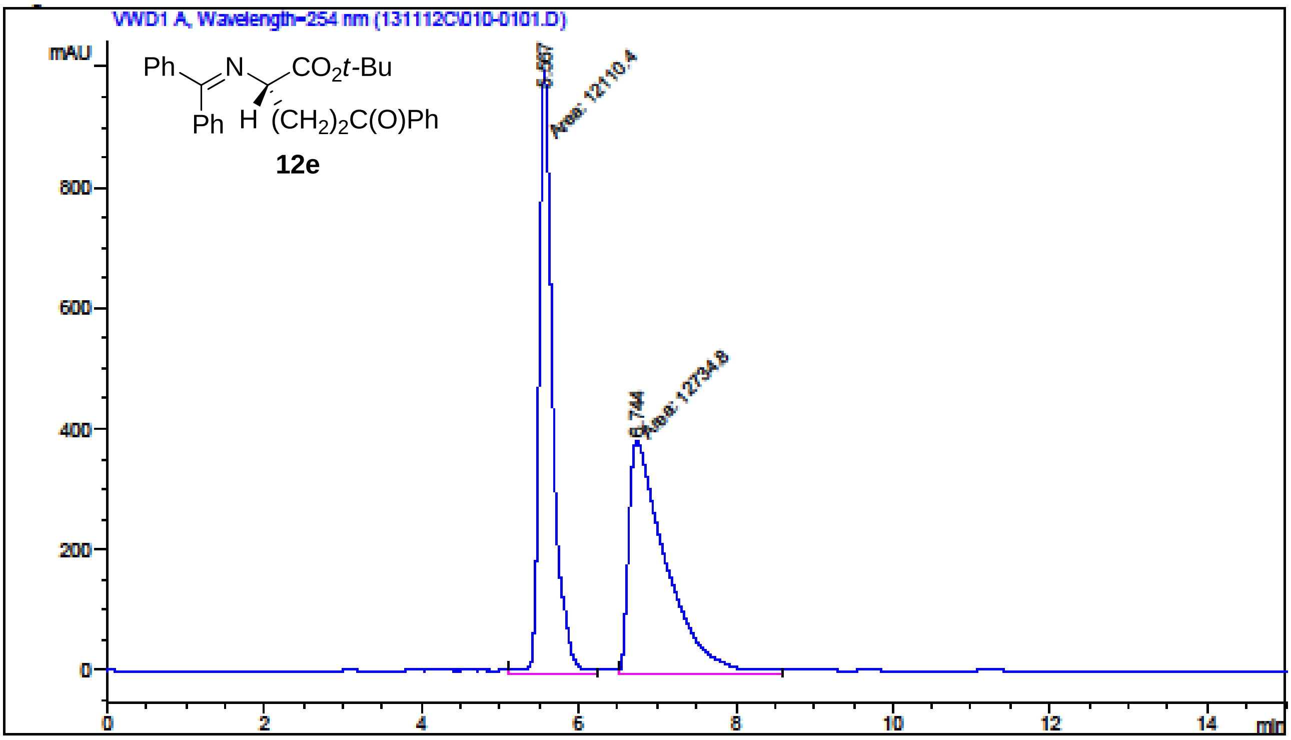

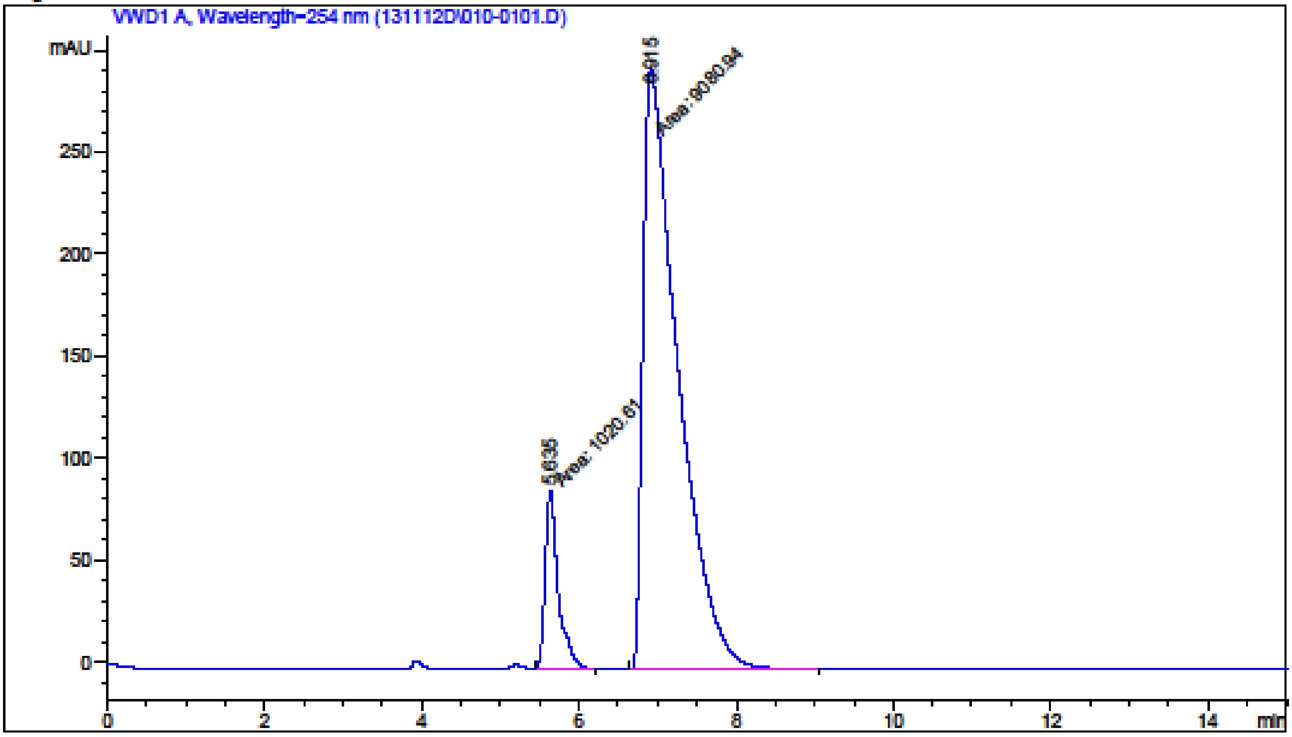

Peak #	RetTime [min]	Type	Width [min]	Area [mAU*s]	Height [mAU]	Area %
1	5.635	MM	0.1947	1020.61237	87.35086	10.1035
2	6.915	MM	0.5134	9080.93555	294.79886	89.8965

80%*ee*

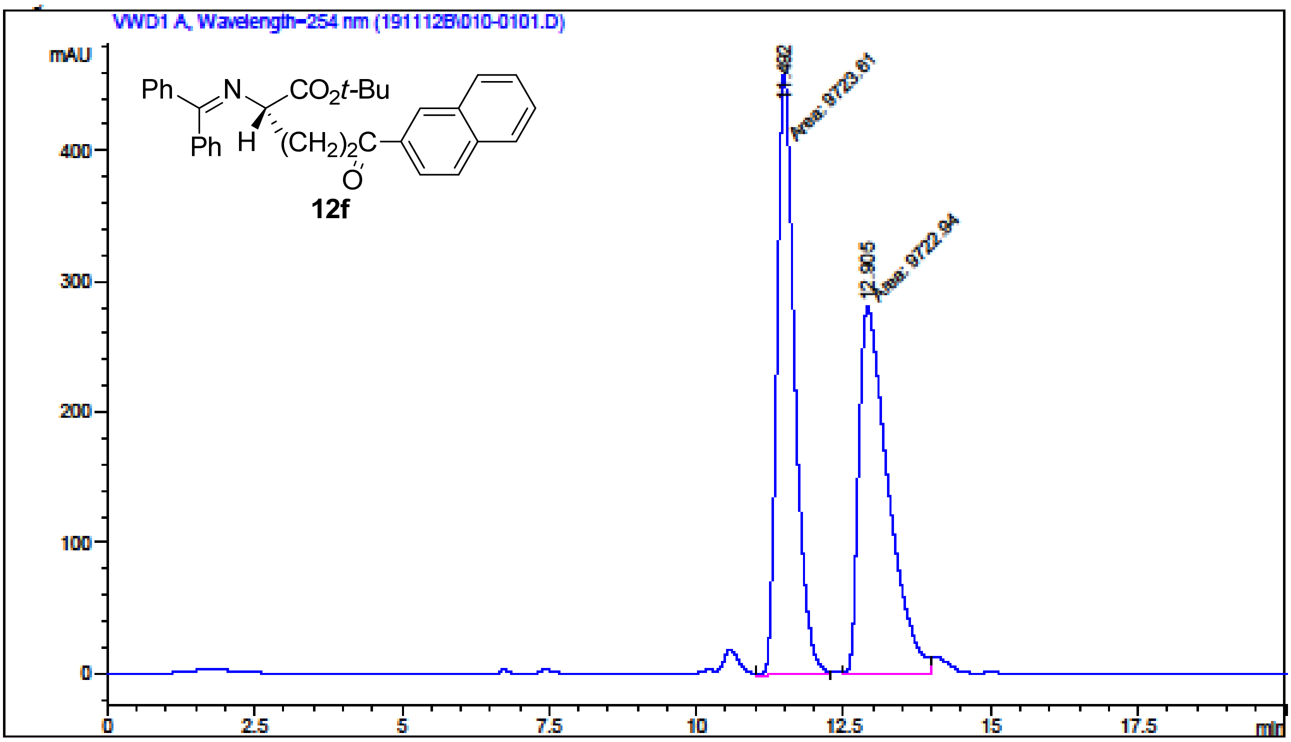

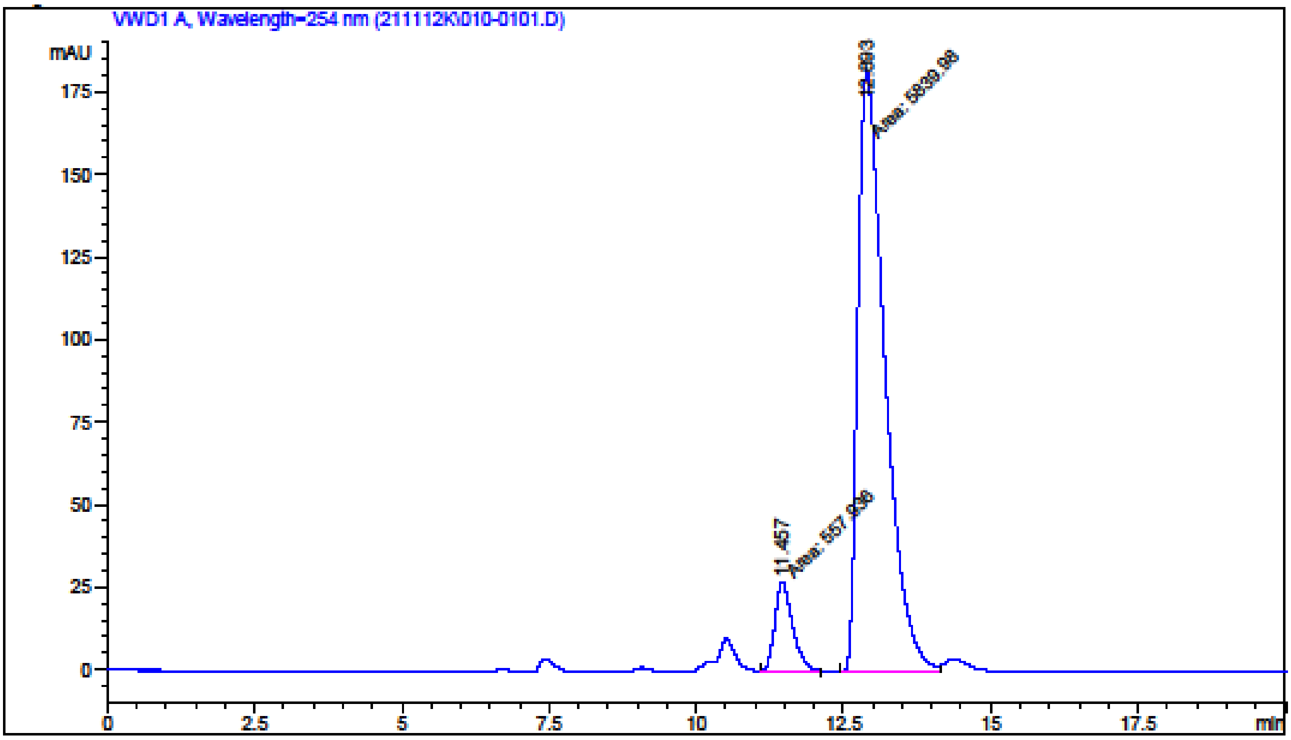

Peak #	RetTime [min]	Type	Width [min]	Area [mAU*s]	Height [mAU]	Area %
1	11.457	MM	0.3453	557.93591	26.92791	8.7206
2	12.893	MM	0.5343	5839.98438	182.18614	91.2794

83%*ee*

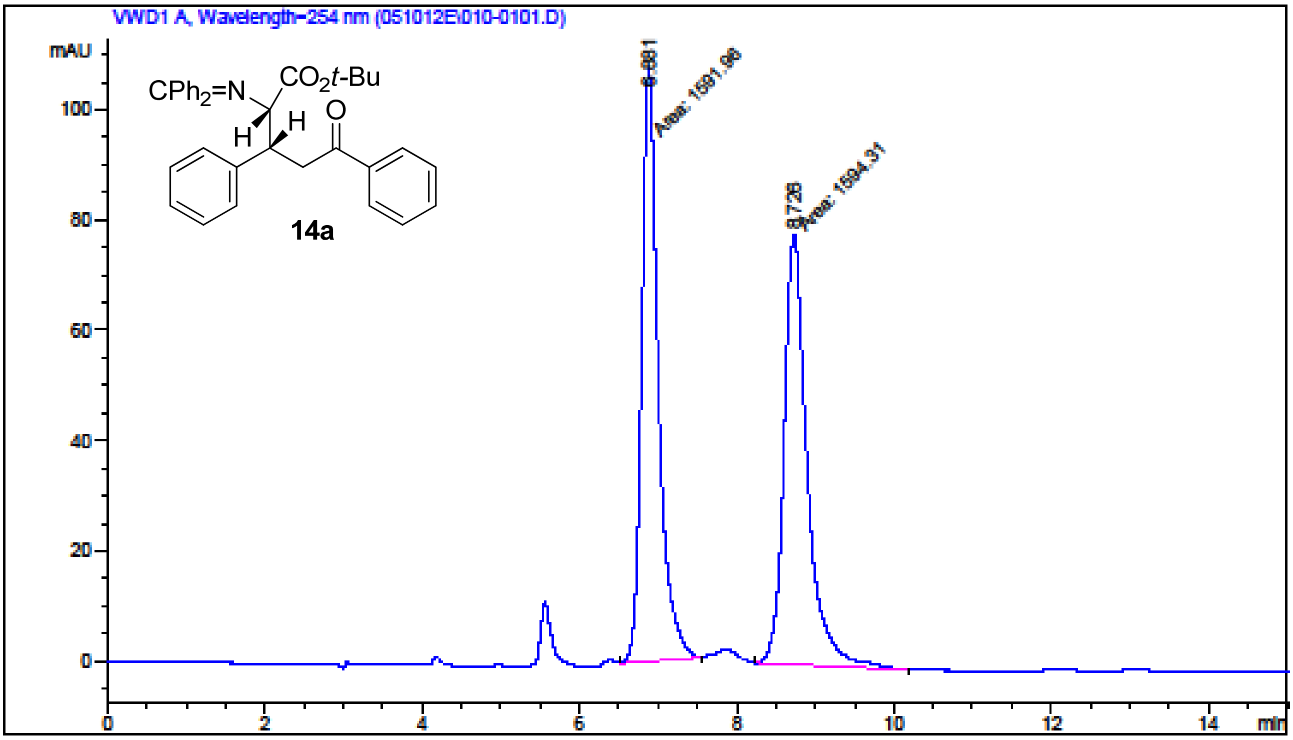

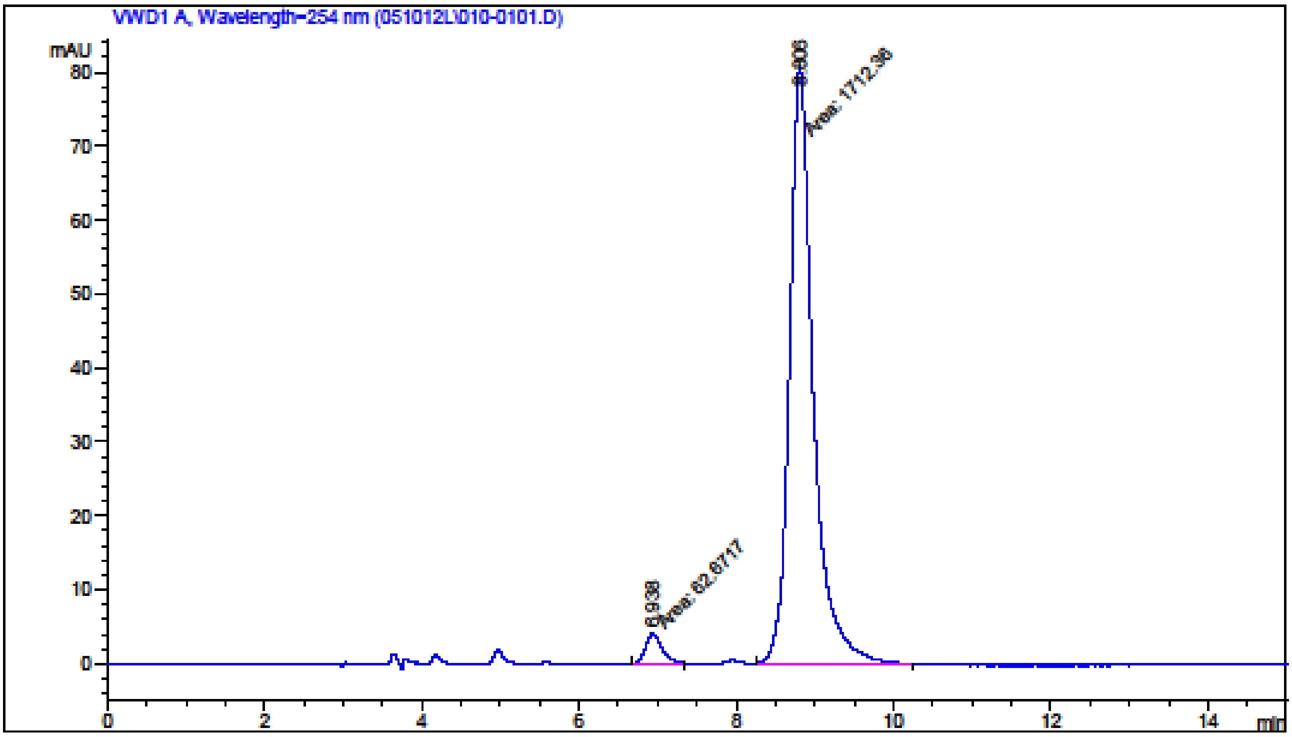

Peak #	RetTime [min]	Type	Width [min]	Area [mAU*s]	Height [mAU]	Area %
1	6.938	MM	0.2468	62.67175	4.23287	3.5307
2	8.806	MM	0.3546	1712.35803	80.49258	96.4693

93%*ee*

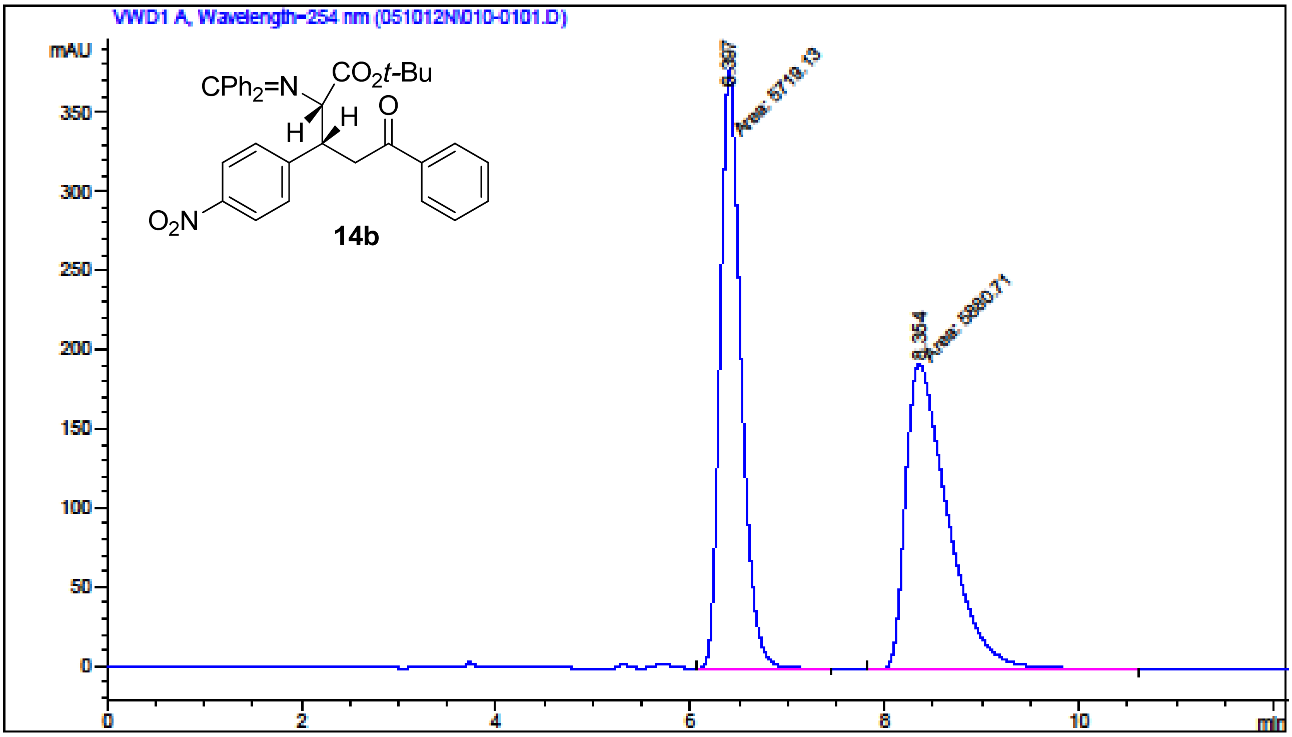

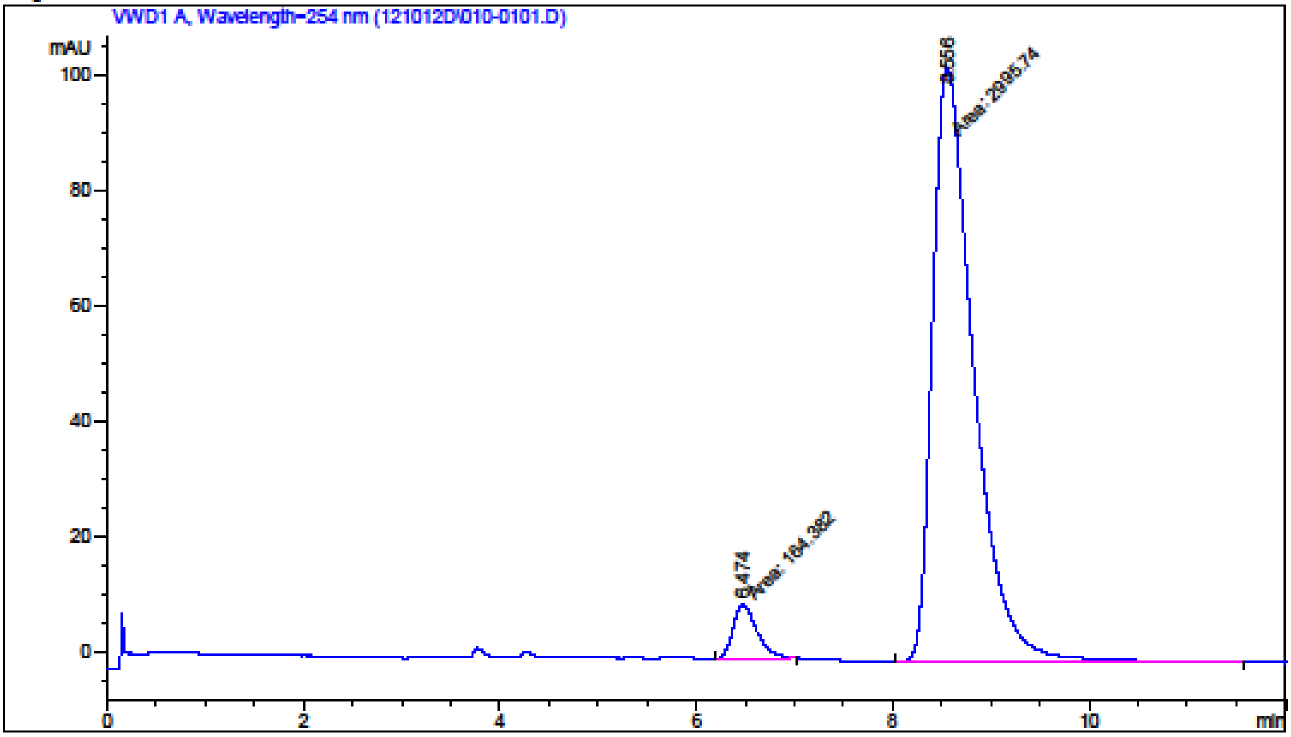

Peak #	RetTime [min]	Type	Width [min]	Area [mAU*s]	Height [mAU]	Area %
1	6.474	MM	0.2878	164.38188	9.51829	5.2018
2	8.556	MM	0.4859	2995.74365	102.74963	94.7982

90%*ee*

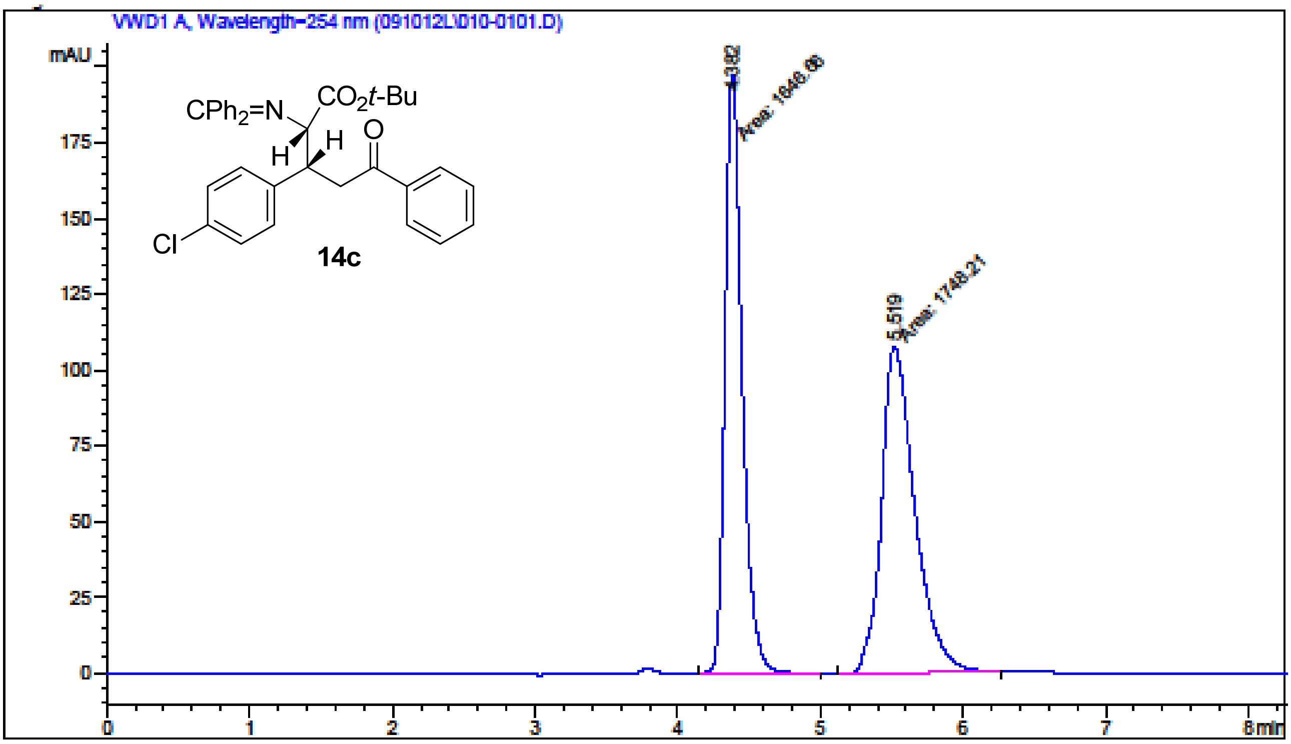

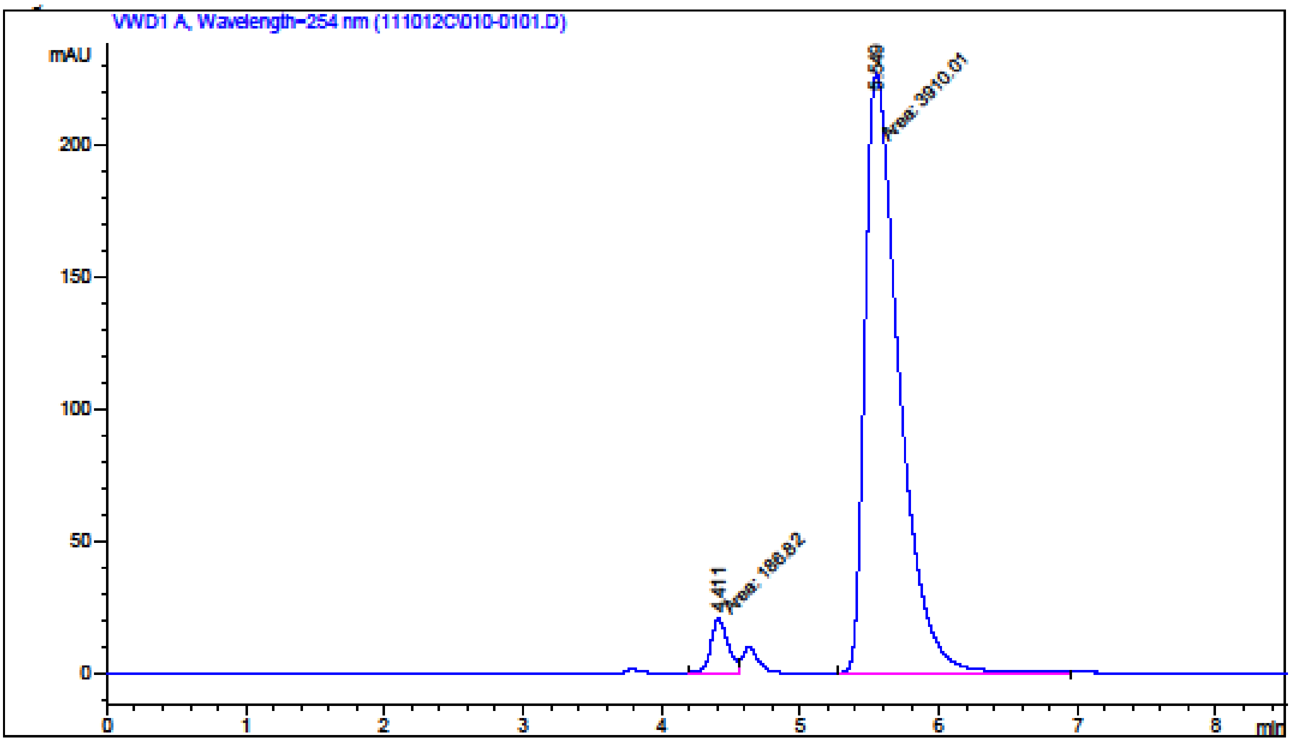

Peak #	RetTime [min]	Type	Width [min]	Area [mAU*s]	Height [mAU]	Area %
1	4.411	MM	0.1502	186.82001	20.72884	4.5601
2	5.549	MM	0.2869	3910.00513	227.12674	95.4399

91%*ee*

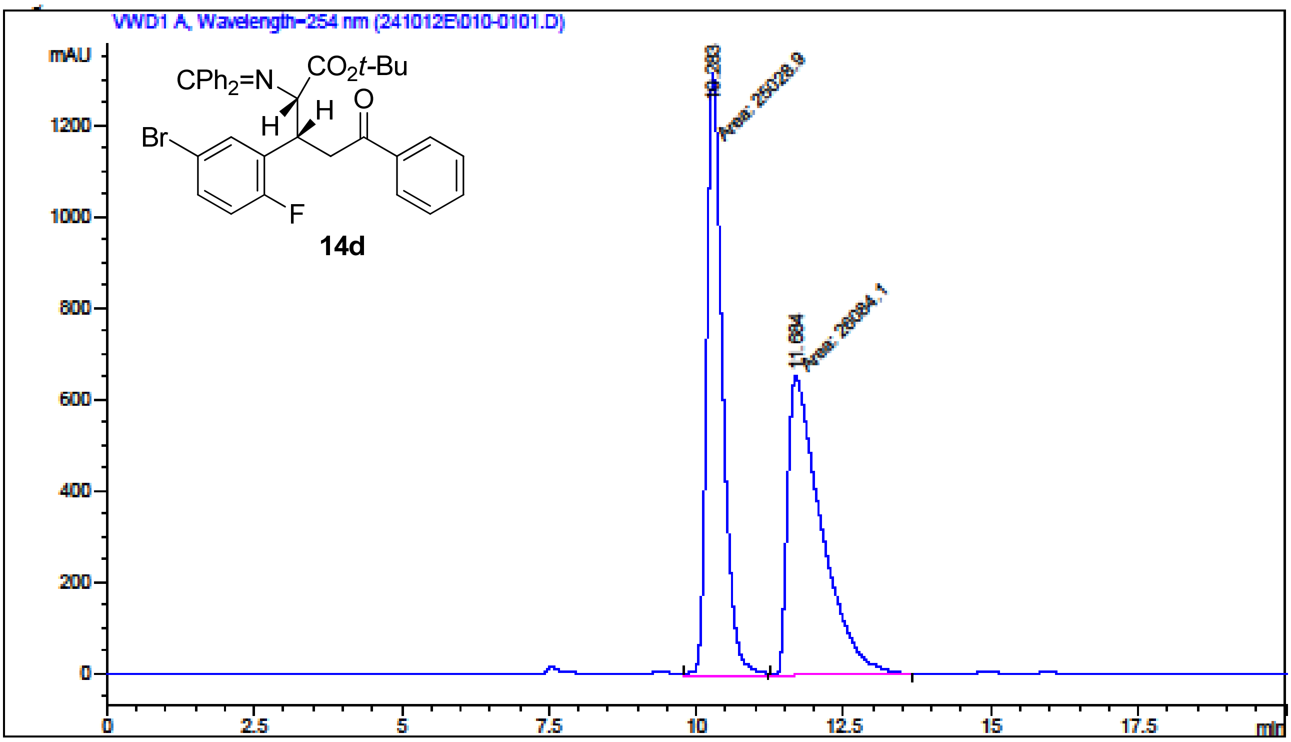

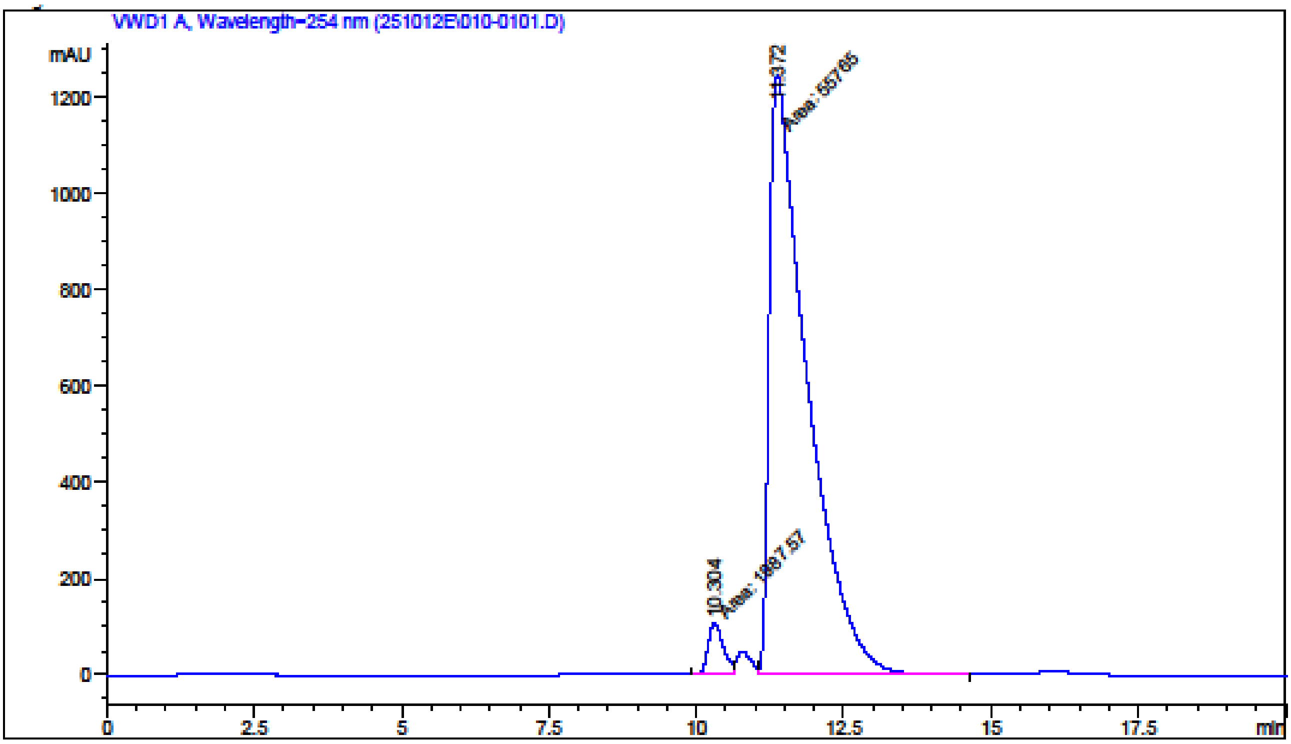

Peak #	RetTime [min]	Type	Width [min]	Area [mAU*s]	Height [mAU]	Area %
1	10.304	MM	0.3018	1987.57068	109.74610	3.4415
2	11.372	MM	0.7443	5.57650e4	1248.76208	96.5585

93%*ee*

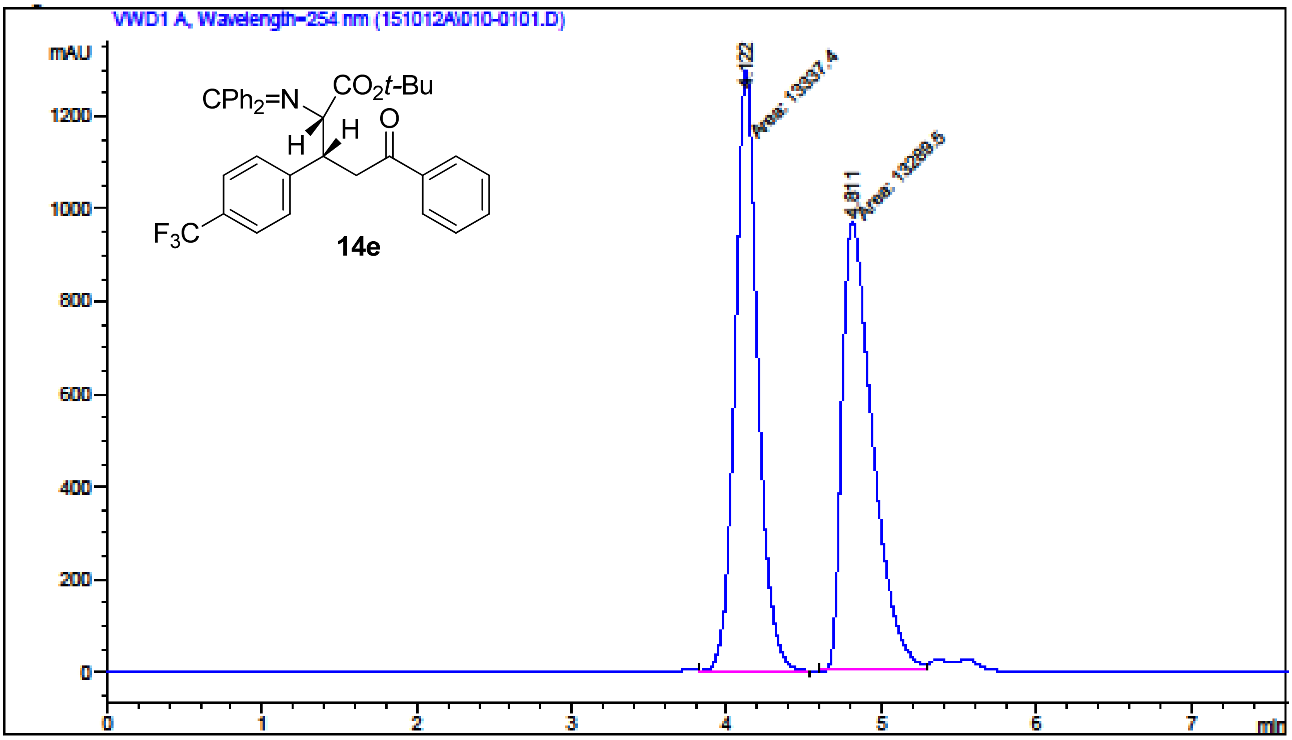

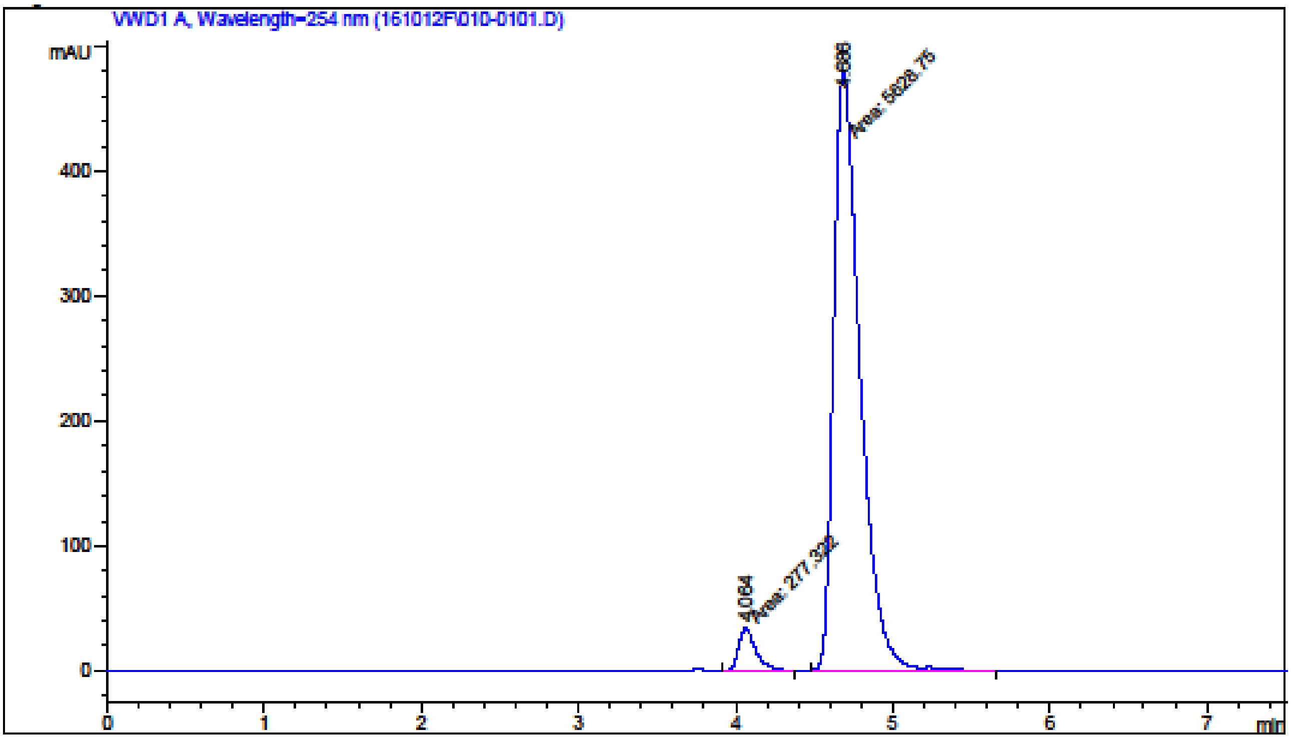

Peak #	RetTime [min]	Type	Width [min]	Area [mAU*s]	Height [mAU]	Area %
1	4.064	MM	0.1332	277.32193	34.70514	4.6955
2	4.686	MM	0.1954	5628.75244	480.08417	95.3045

91%*ee*

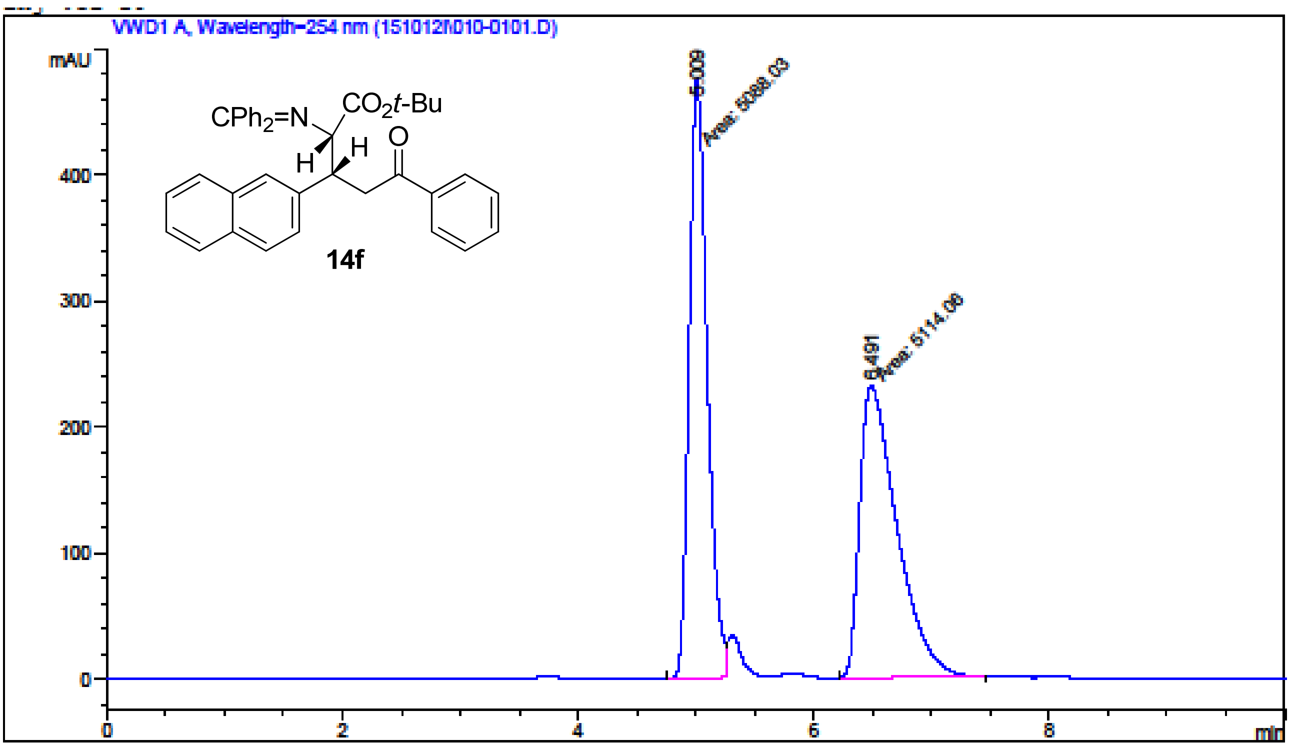

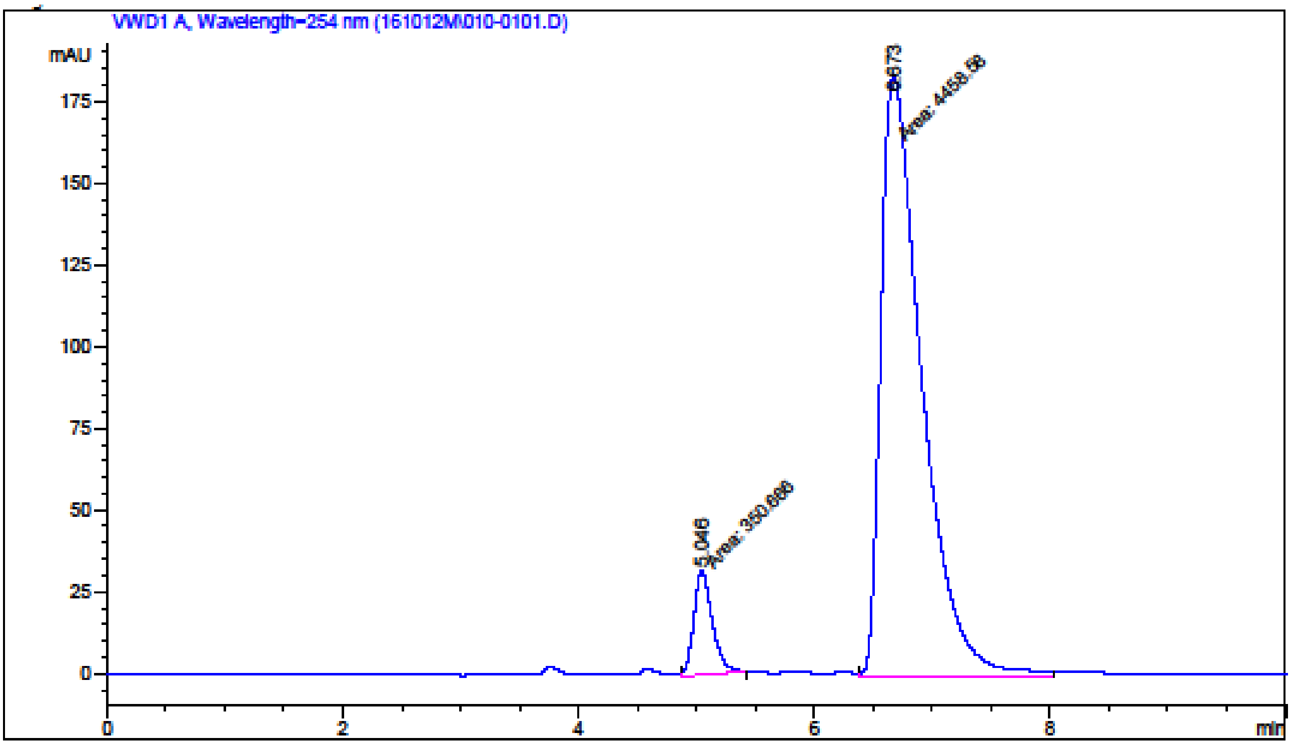

Peak #	RetTime [min]	Type	Width [min]	Area [mAU*s]	Height [mAU]	Area %
1	5.046	MM	0.1838	350.66614	31.79868	7.2915
2	6.673	MM	0.4033	4458.57910	184.26573	92.7085

85%*ee*

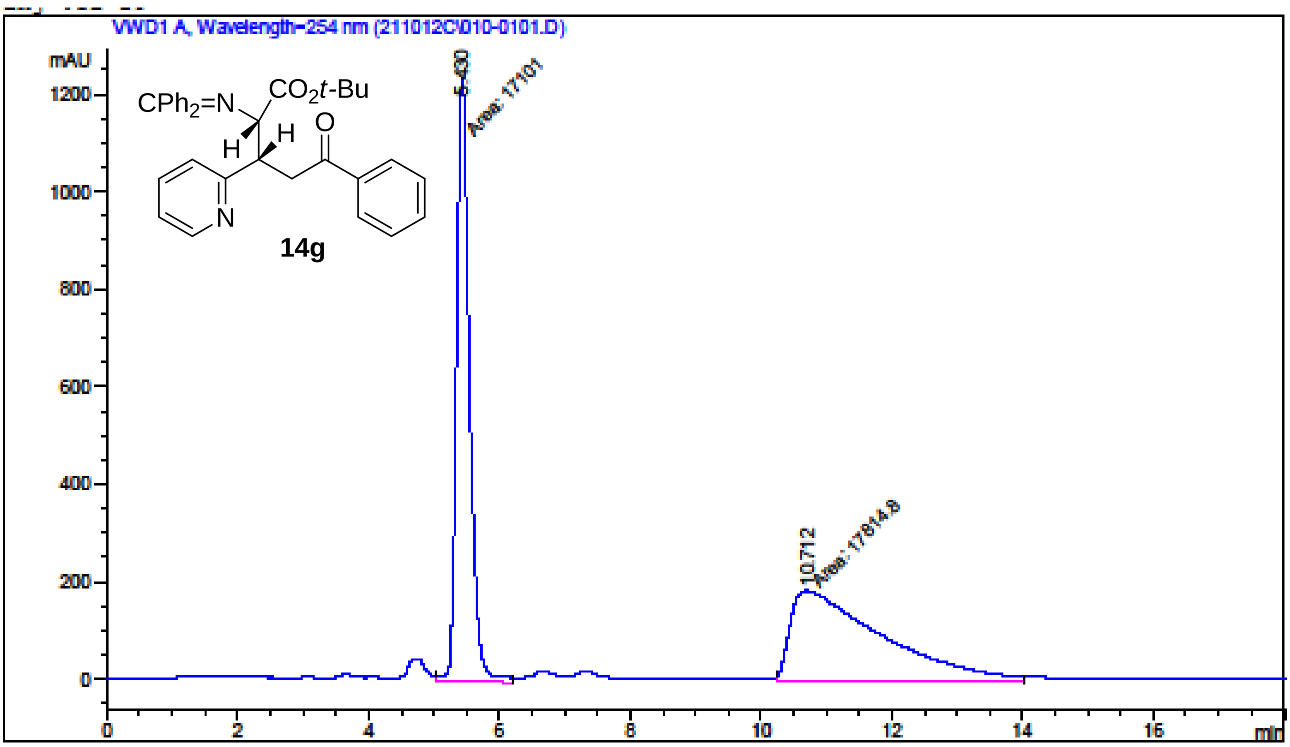

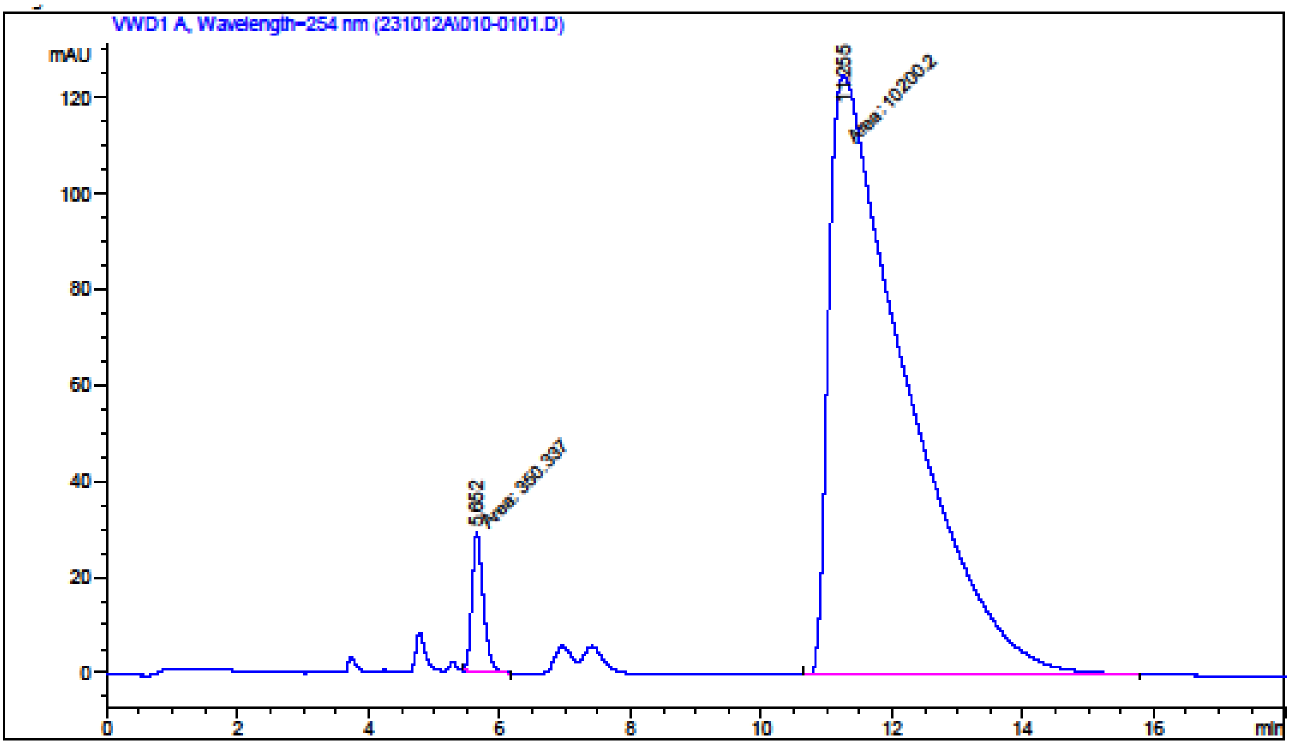

Peak #	RetTime [min]	Type	Width [min]	Area [mAU*s]	Height [mAU]	Area %
1	5.652	MM	0.2013	350.33740	29.00694	3.3205
2	11.255	MM	1.3602	1.02002e4	124.98445	96.6795

93%*ee*

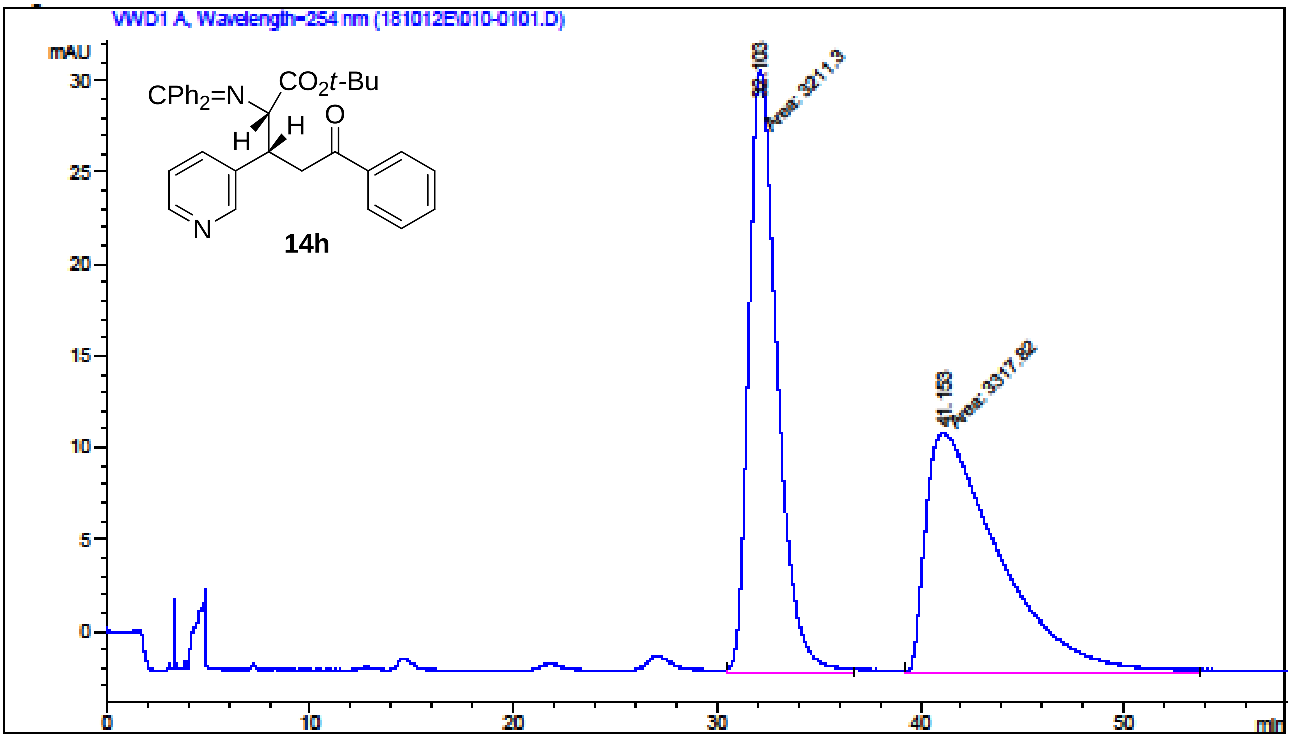

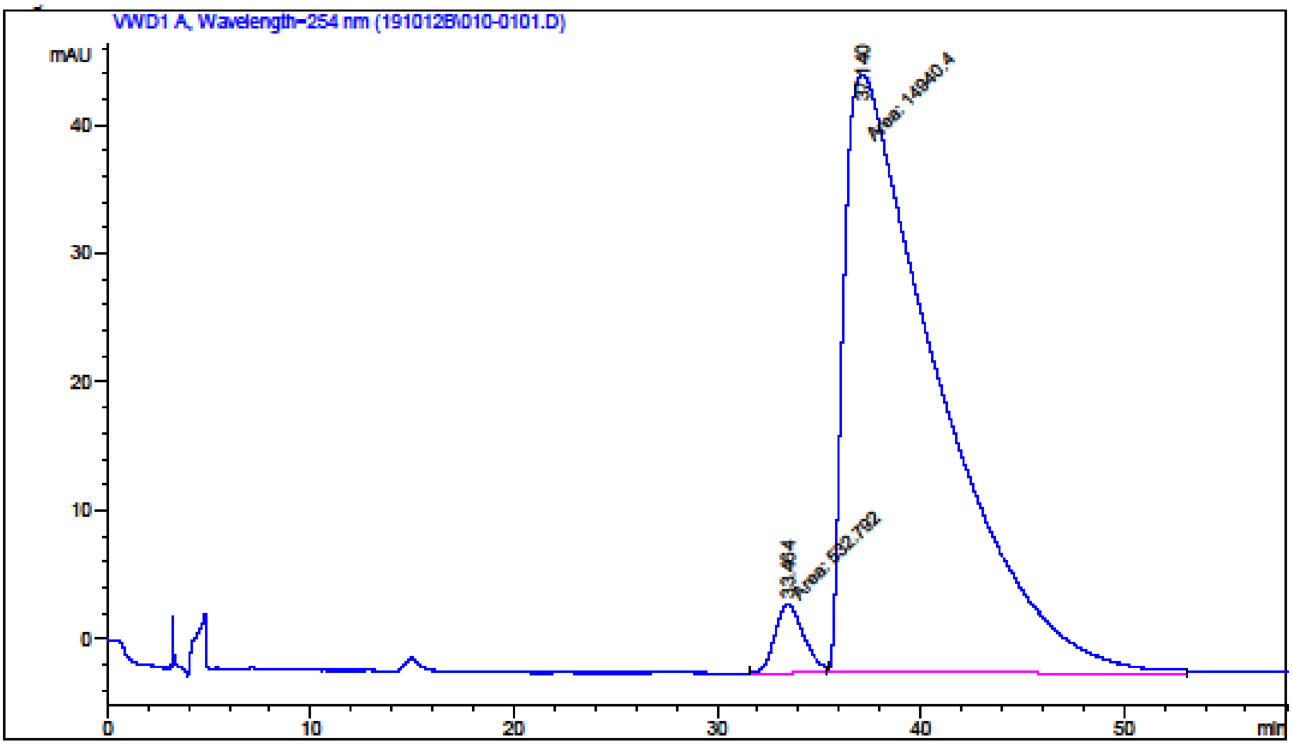

Peak #	RetTime [min]	Type	Width [min]	Area [mAU*s]	Height [mAU]	Area %
1	33.464	MM	1.6331	532.79175	5.43747	3.4433
2	37.140	MM	5.3515	1.49404e4	46.53058	96.5567

93%*ee*

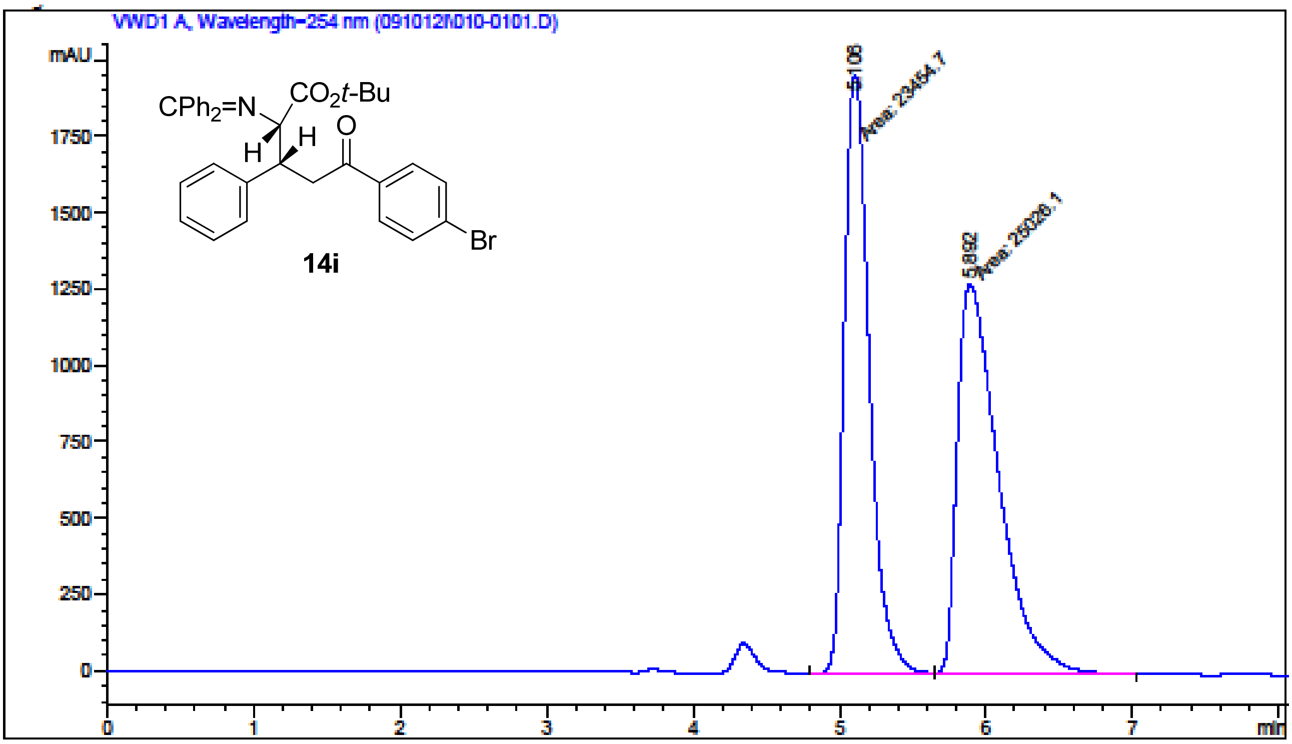

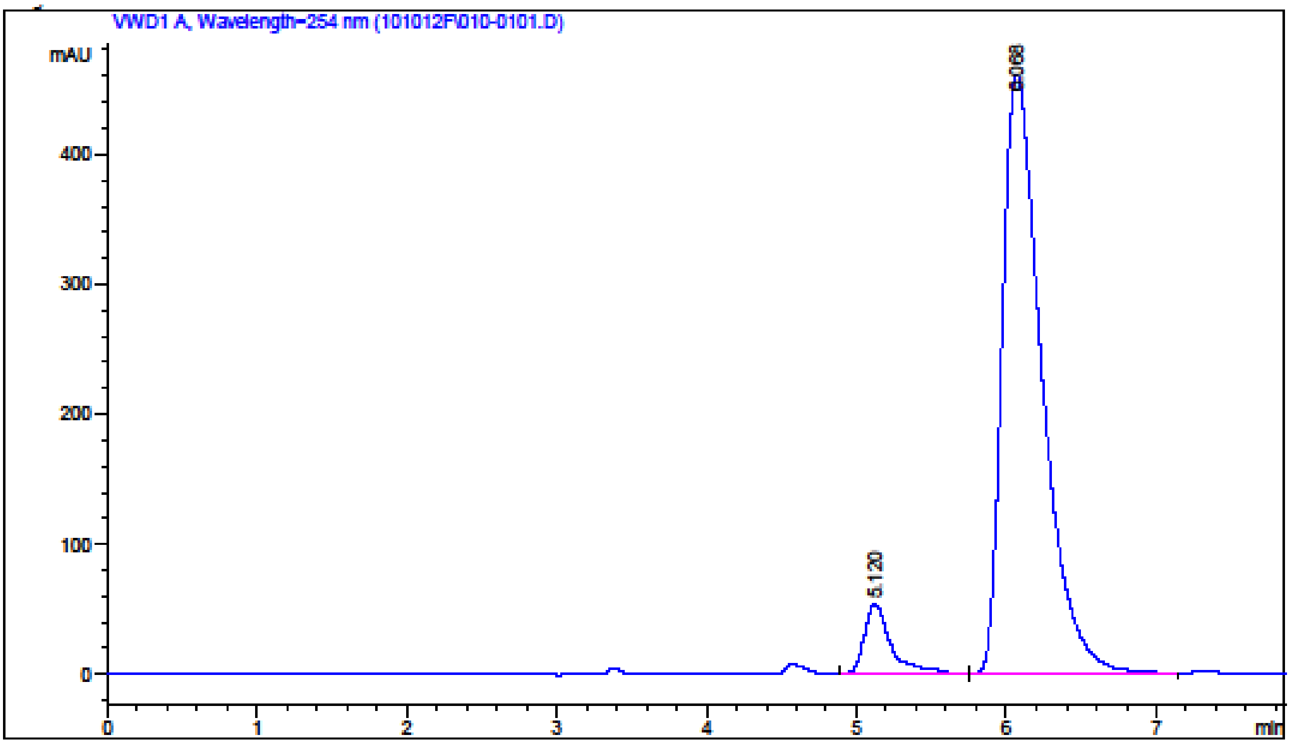

Peak #	RetTime [min]	Type	Width [min]	Area [mAU*s]	Height [mAU]	Area %
1	5.120	BV	0.1883	697.81183	54.51445	7.5671
2	6.068	VV	0.2763	8523.82227	461.98526	92.4329

85%*ee*

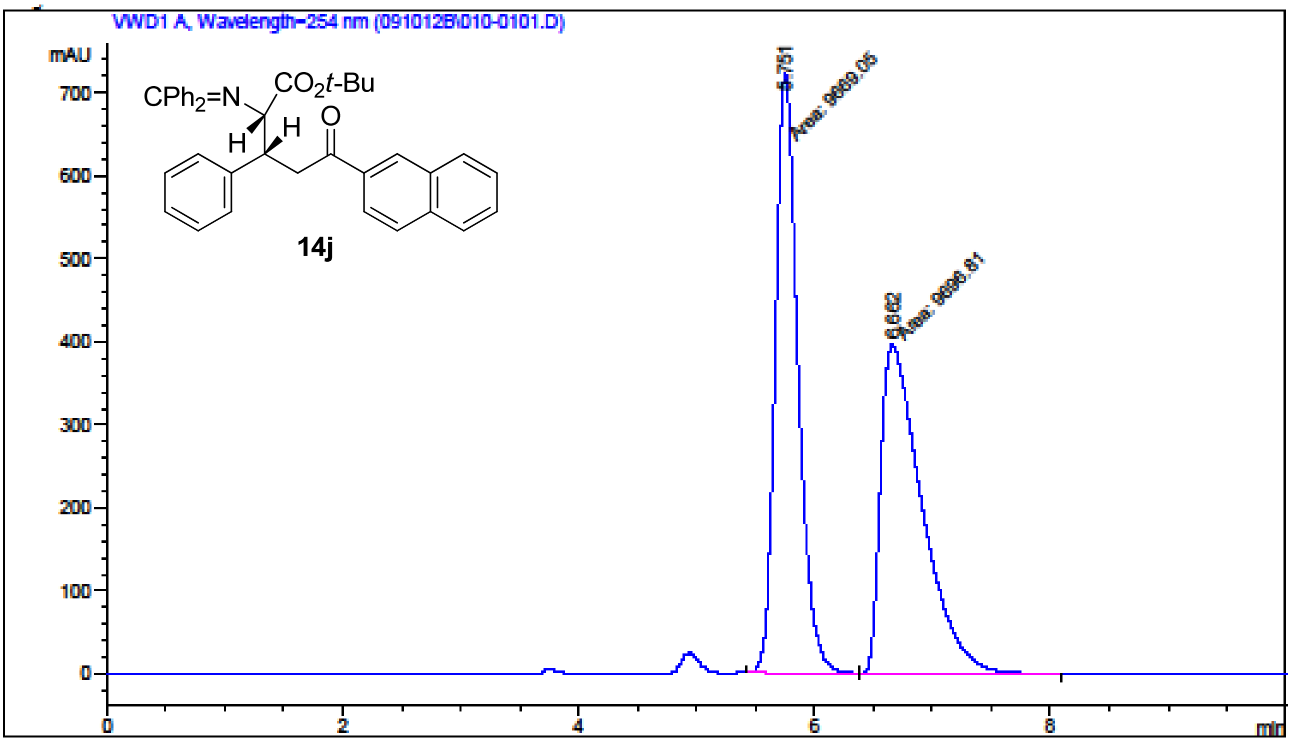

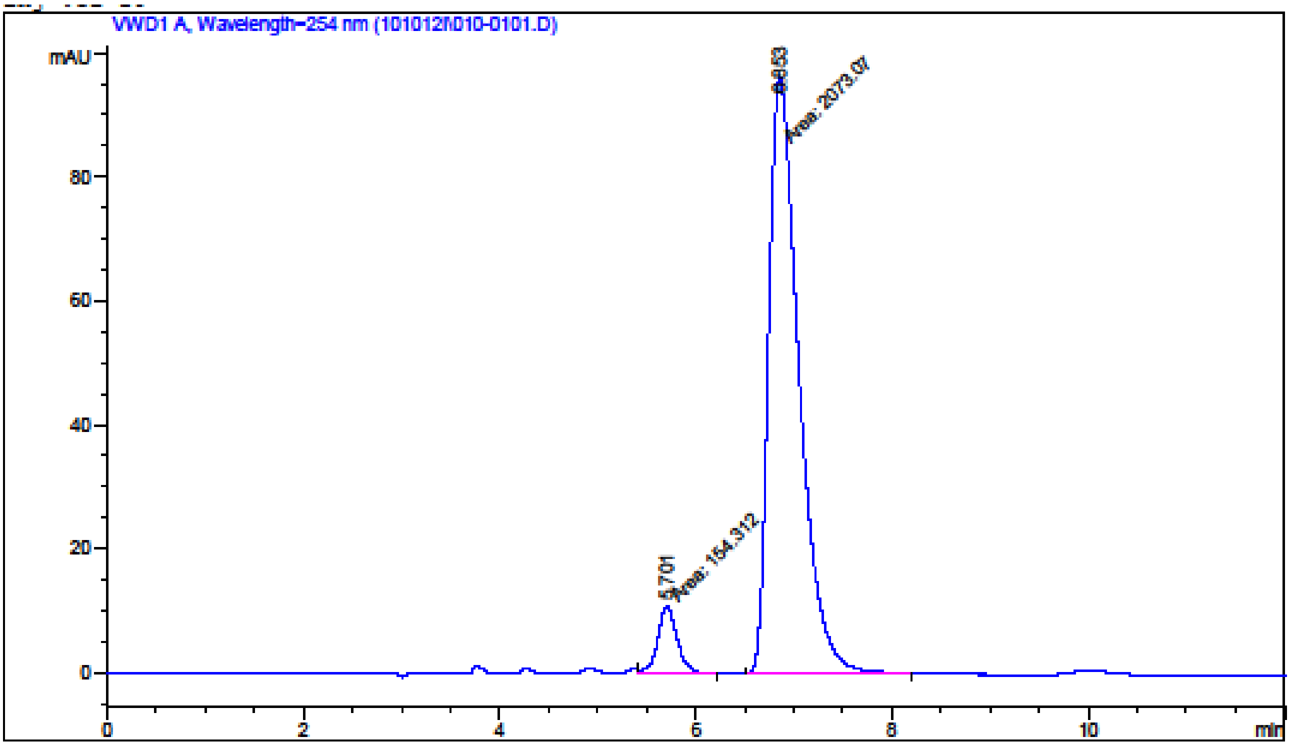

Peak #	RetTime [min]	Type	Width [min]	Area [mAU*s]	Height [mAU]	Area %
1	5.701	MM	0.2326	154.31216	11.05789	6.9280
2	6.853	MM	0.3584	2073.06689	96.39411	93.0720

86%*ee*

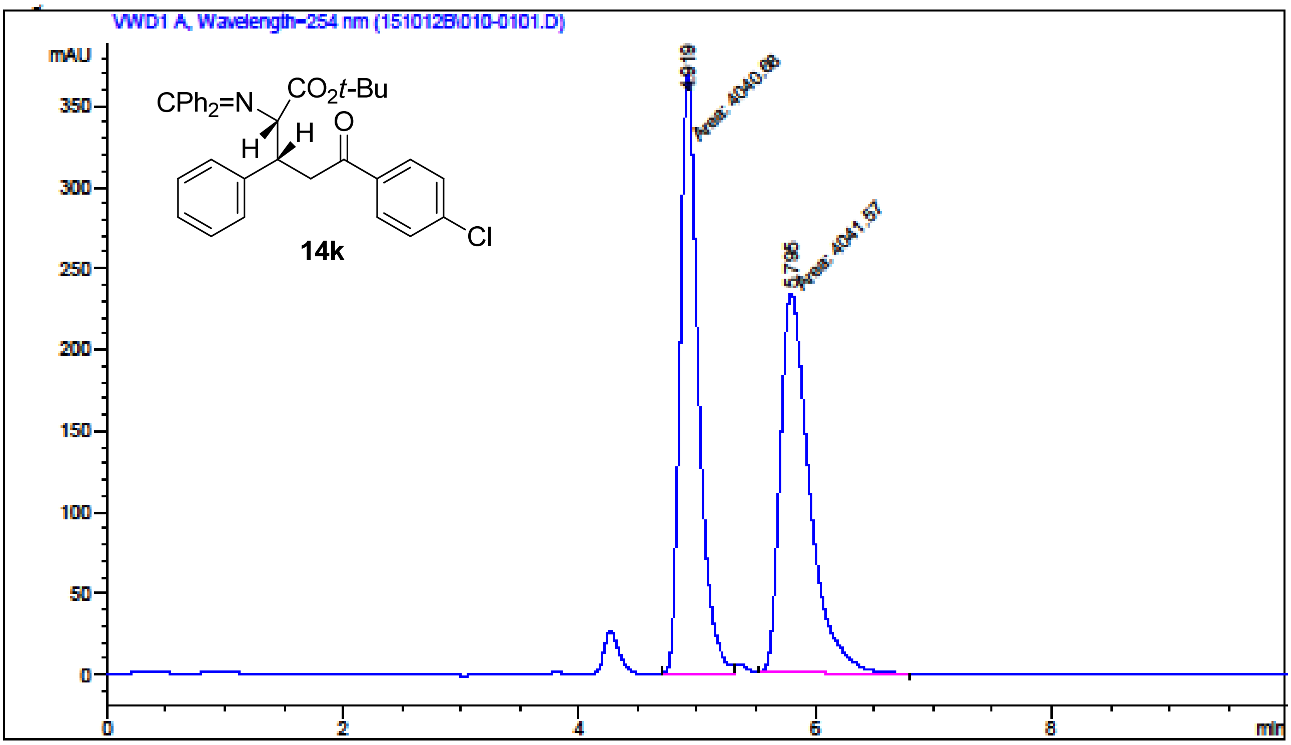

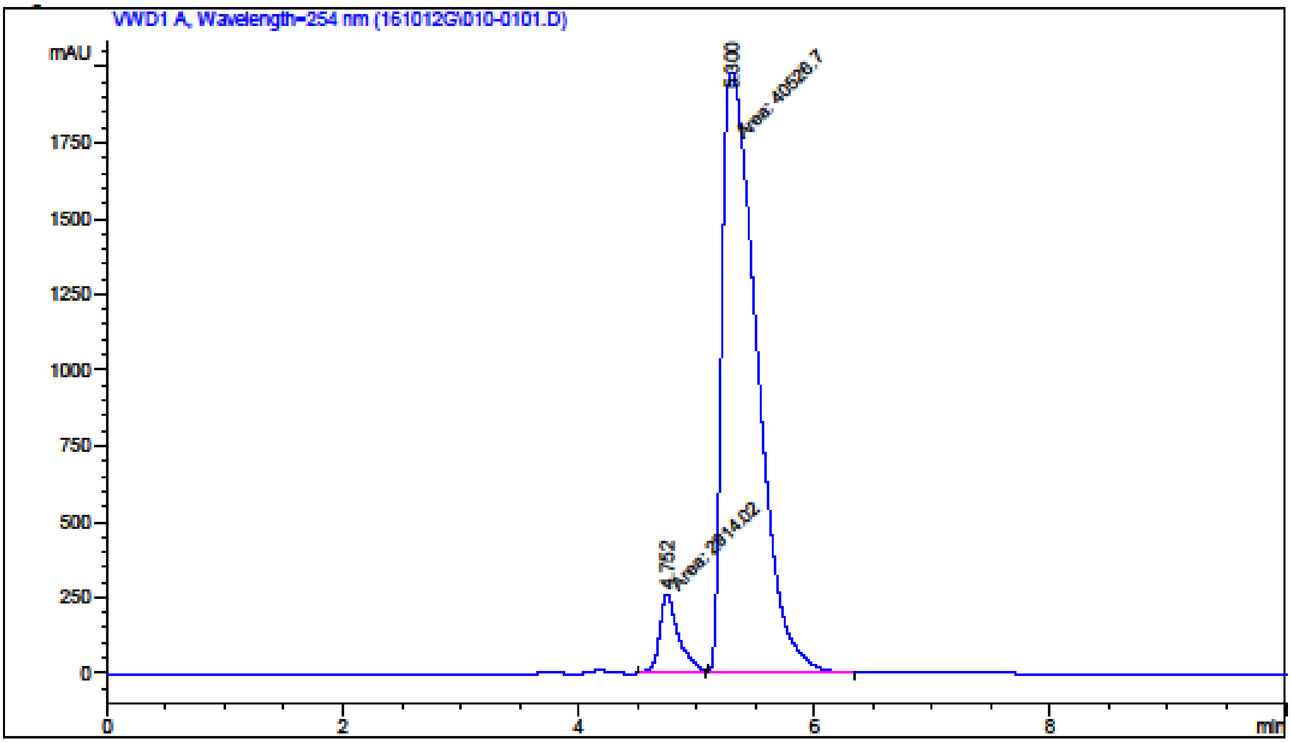

Peak #	RetTime [min]	Type	Width [min]	Area [mAU*s]	Height [mAU]	Area %
1	4.752	MM	0.1862	2914.01733	260.83157	6.7080
2	5.300	MM	0.3411	4.05267e4	1979.96960	93.2920

87%*ee*

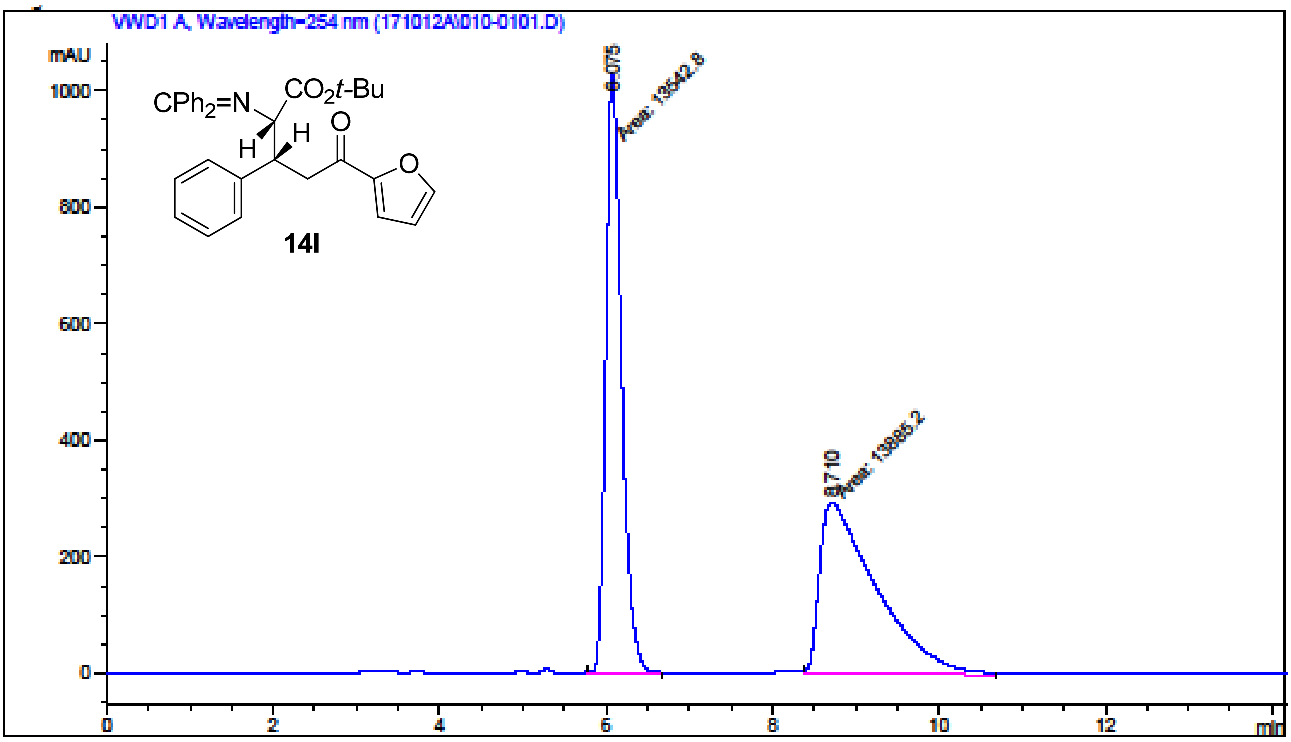

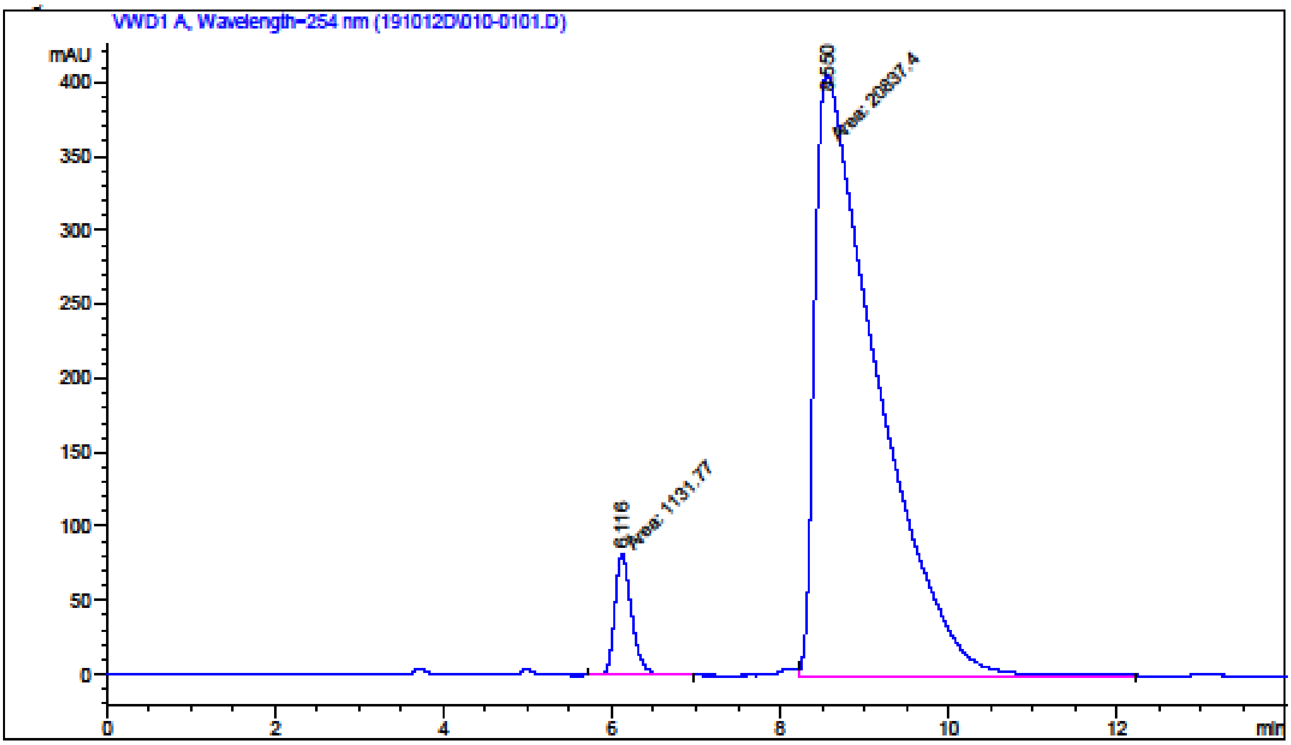

Peak #	RetTime [min]	Type	Width [min]	Area [mAU*s]	Height [mAU]	Area %
1	6.116	MM	0.2305	1131.76563	81.82841	5.1516
2	8.550	MM	0.8550	2.08374e4	406.16452	94.8484

90%*ee*

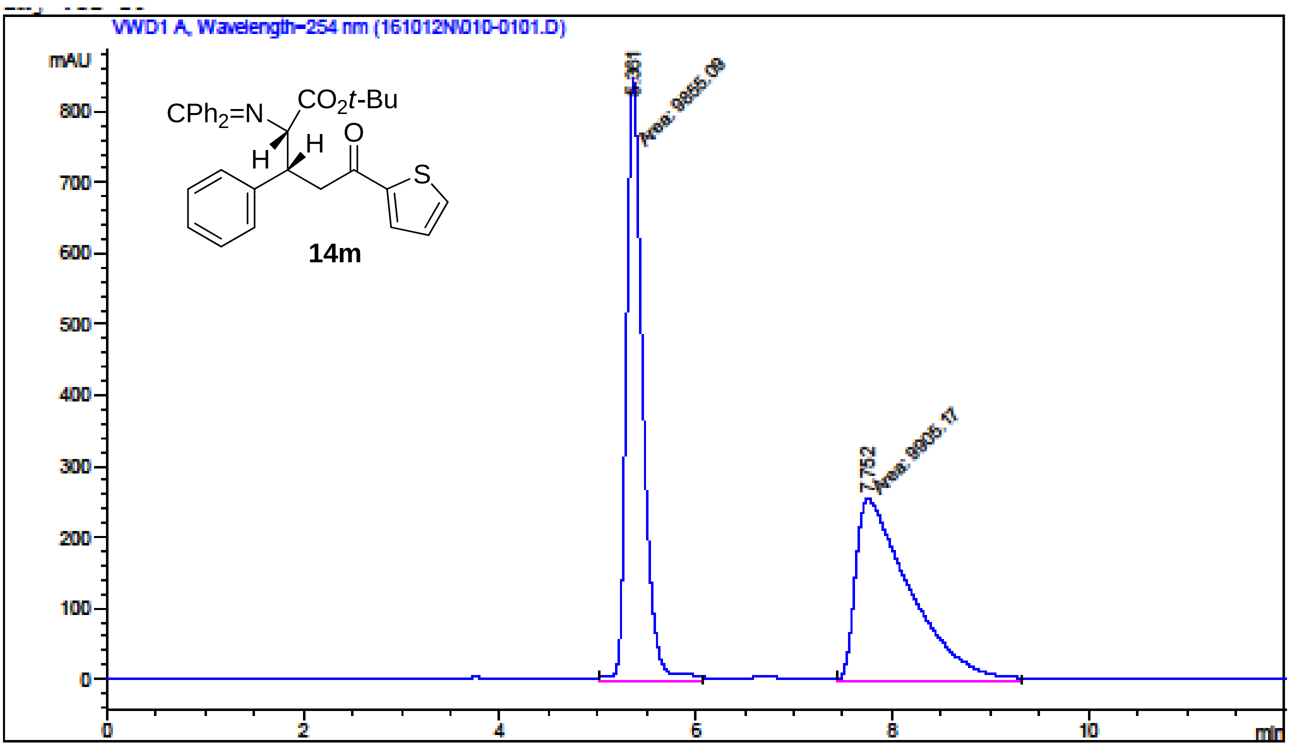

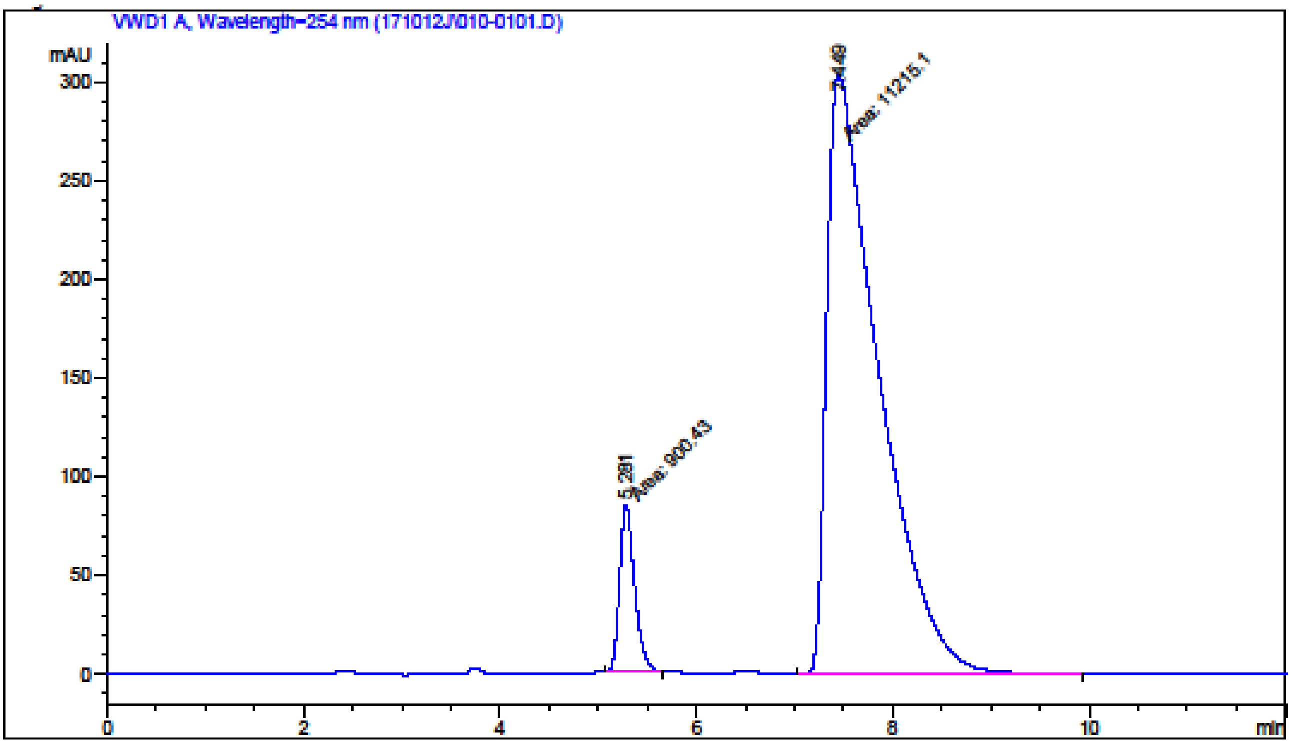

Peak #	RetTime [min]	Type	Width [min]	Area [mAU*s]	Height [mAU]	Area %
1	5.281	MM	0.1765	900.43018	85.00512	7.4320
2	7.449	MM	0.6146	1.12151e4	304.13290	92.5680

85%*ee*

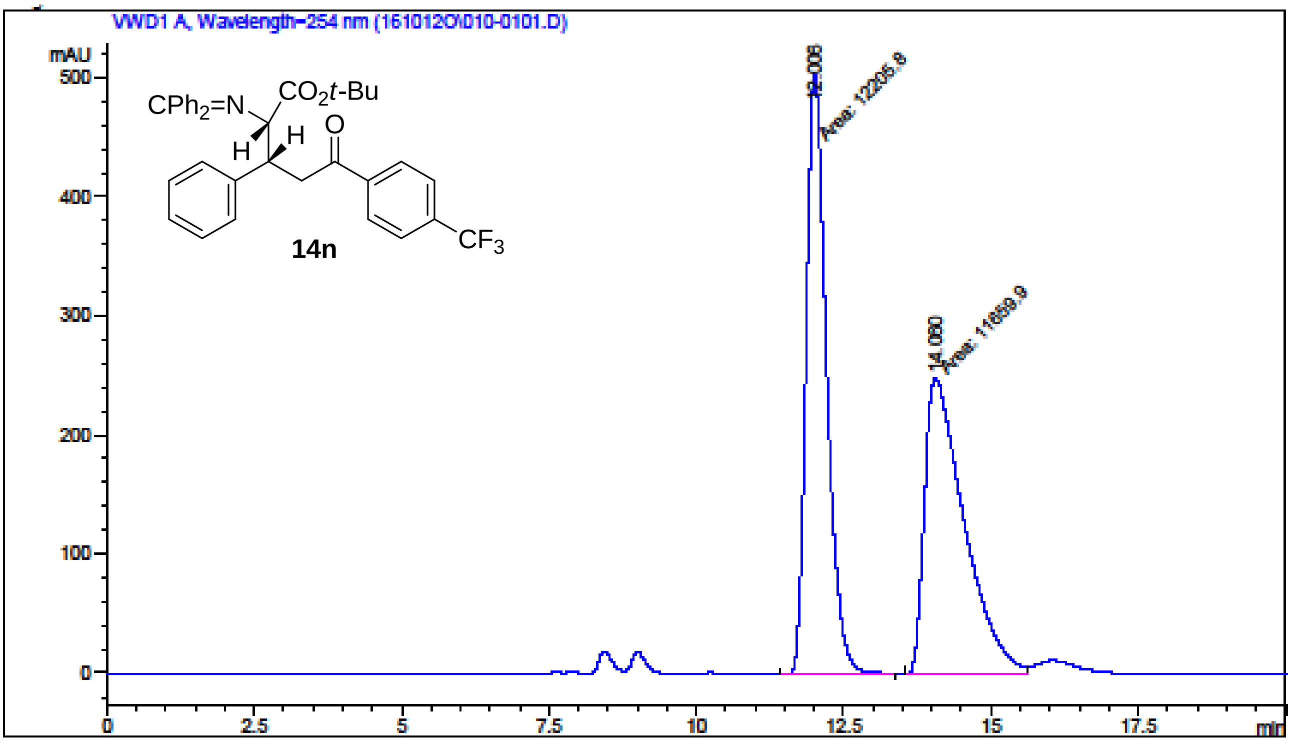

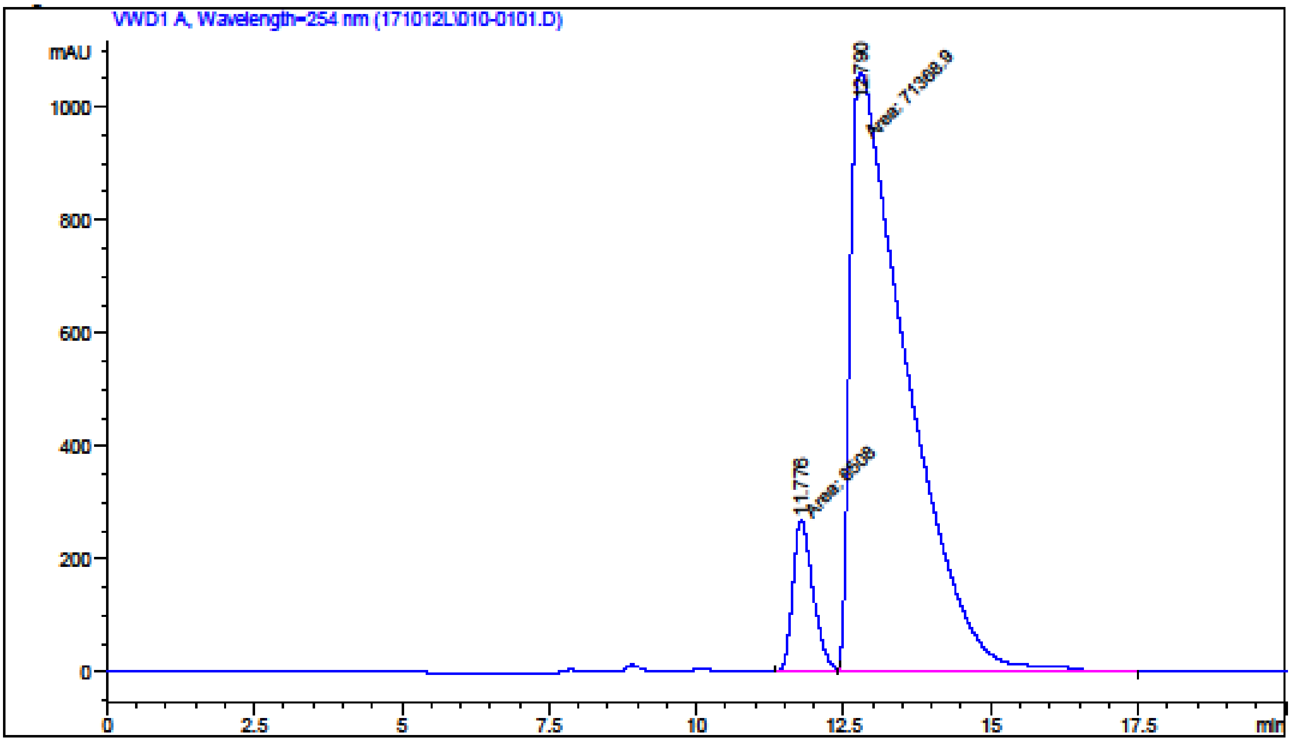

Peak #	RetTime [min]	Type	Width [min]	Area [mAU*s]	Height [mAU]	Area %
1	11.776	MM	0.4039	6507.99951	268.52405	8.3568
2	12.790	MM	1.1189	7.13689e4	1063.08875	91.6432

83%*ee*

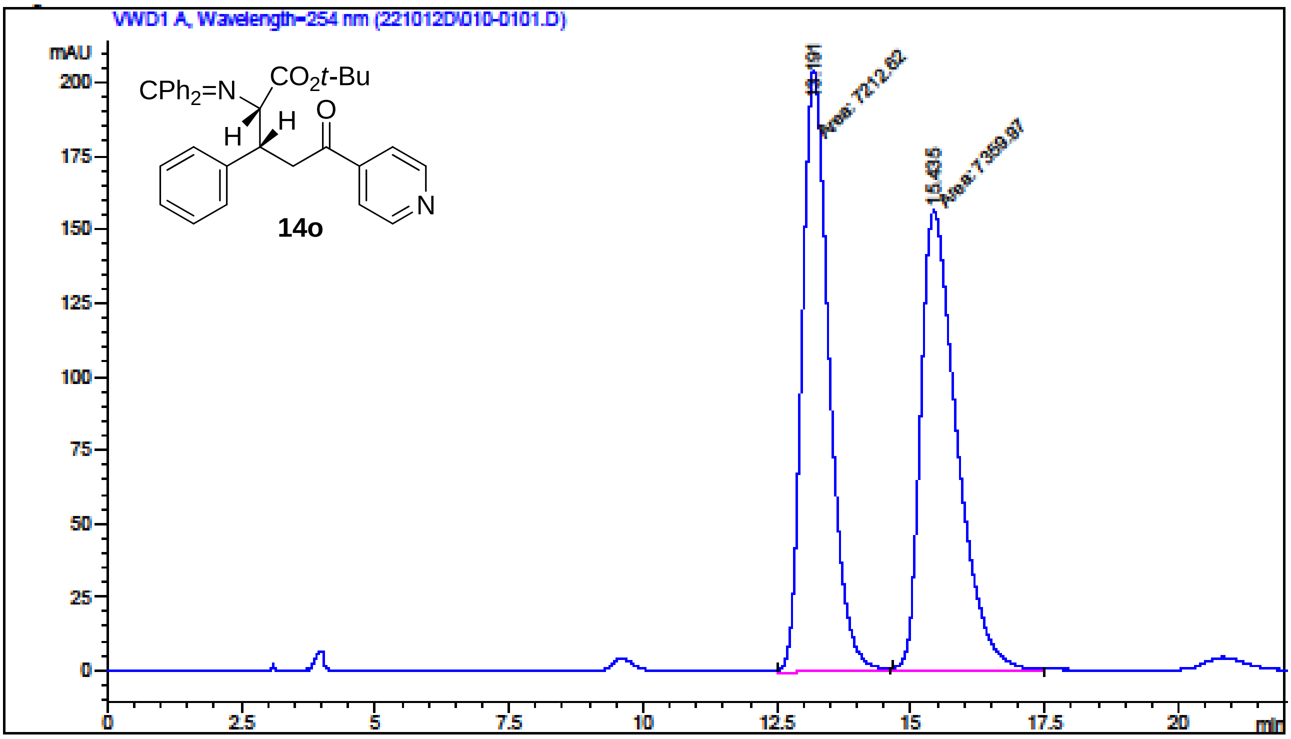

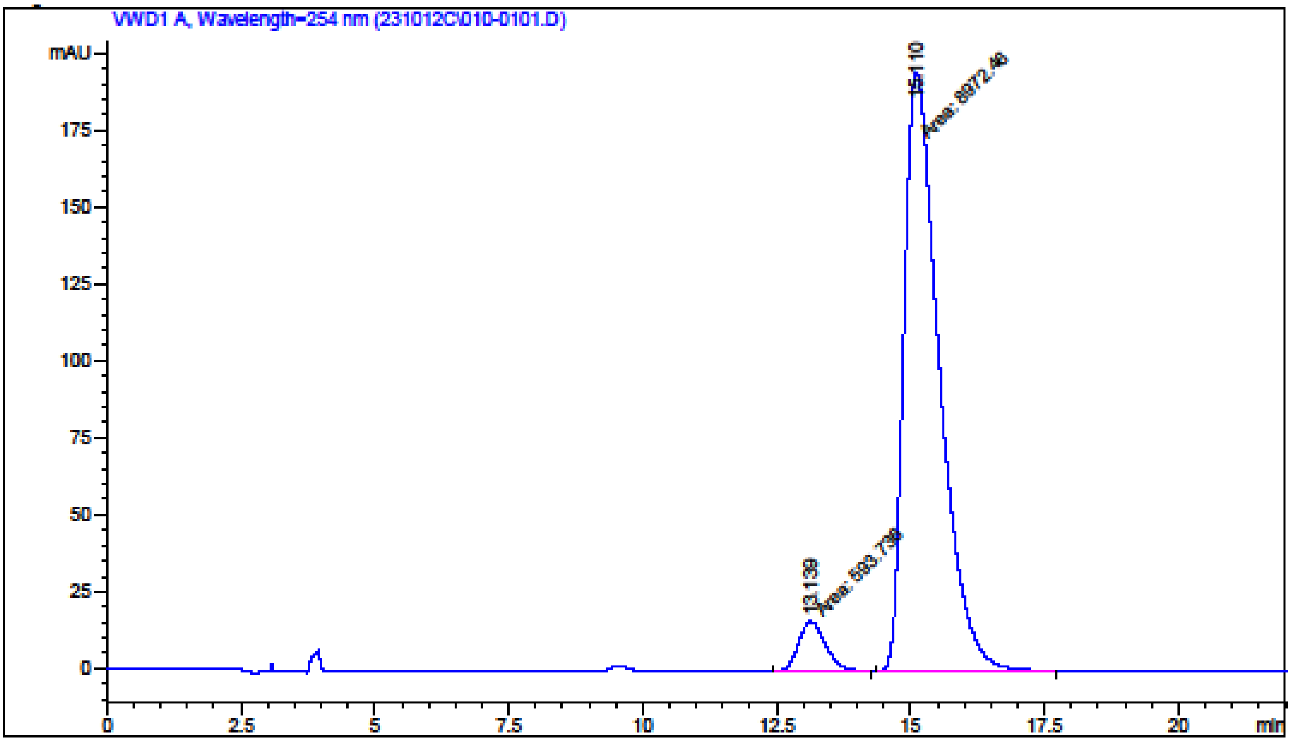

Peak #	RetTime [min]	Type	Width [min]	Area [mAU*s]	Height [mAU]	Area %
1	13.139	MM	0.5998	593.73602	16.49706	6.2066
2	15.110	MM	0.7671	8972.46484	194.95250	93.7934

88%*ee*

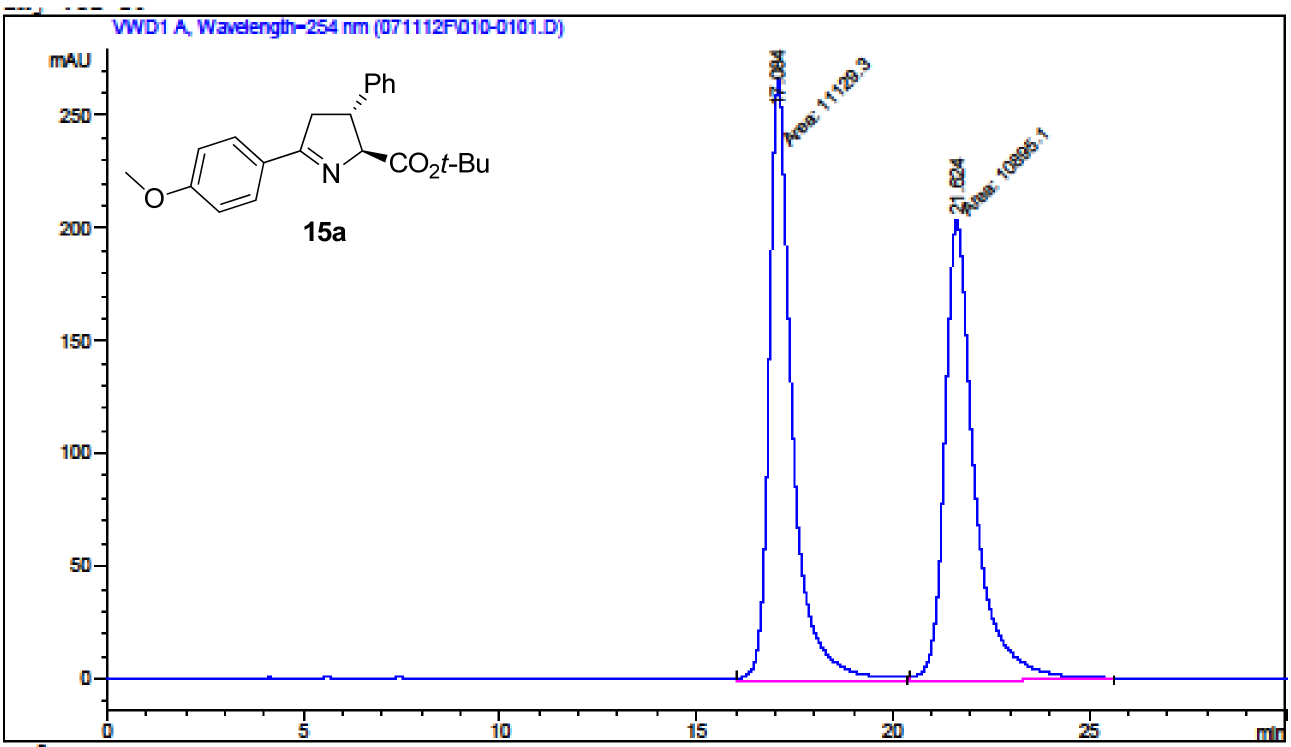

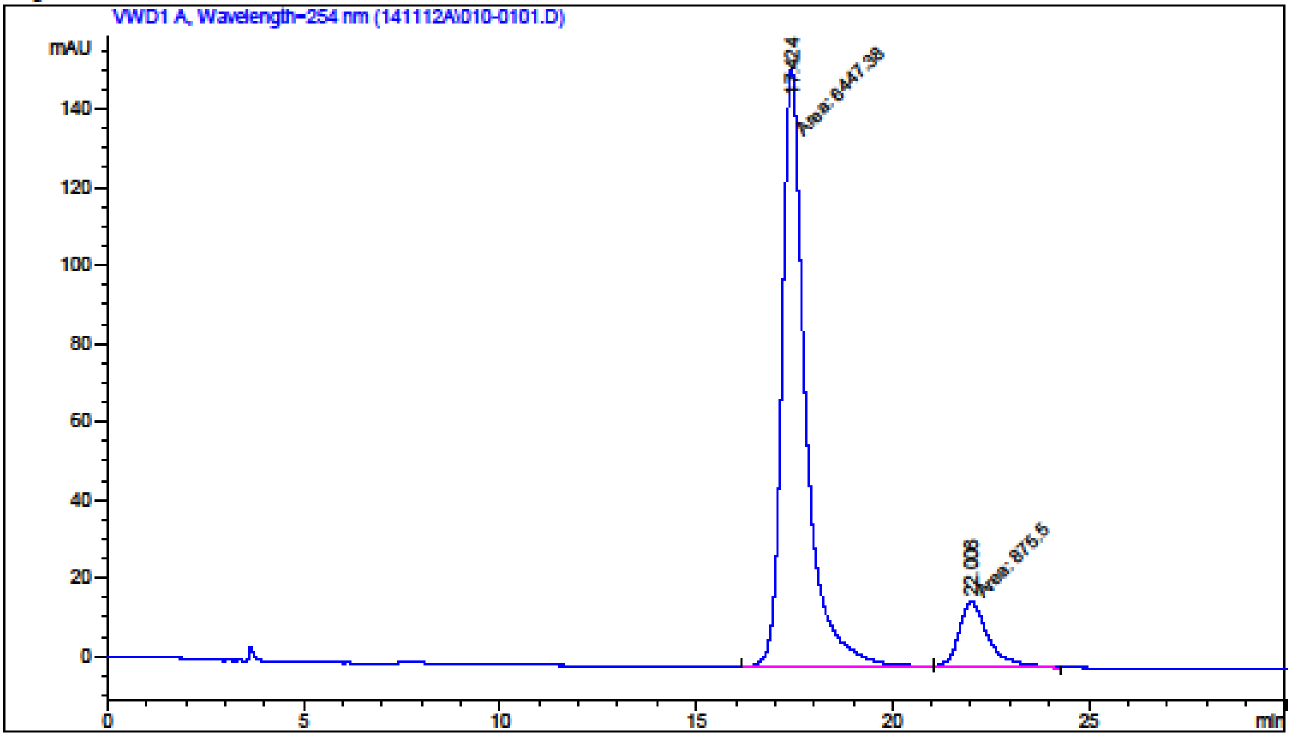

Peak #	RetTime [min]	Type	Width [min]	Area [mAU*s]	Height [mAU]	Area %
1	17.424	MM	0.7010	6447.37500	153.28888	88.0443
2	22.006	MM	0.8735	875.49988	16.70433	11.9557

76%*ee*

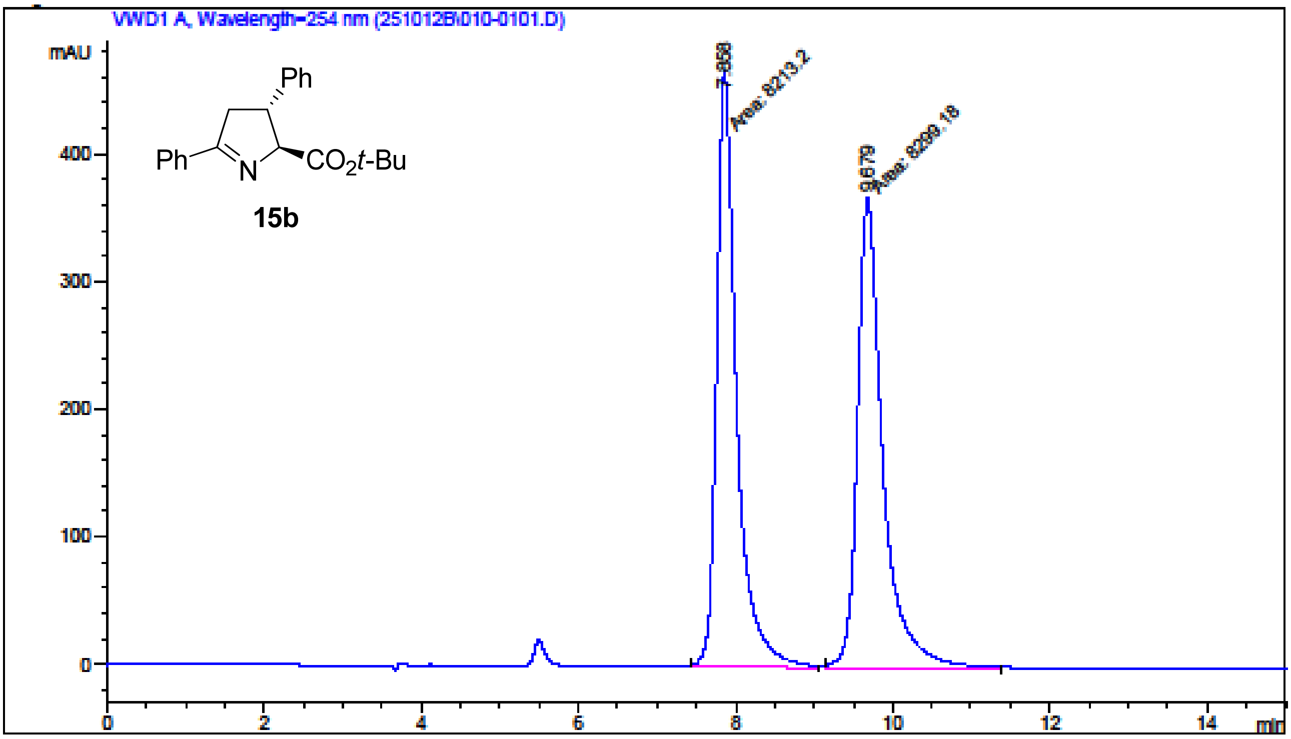

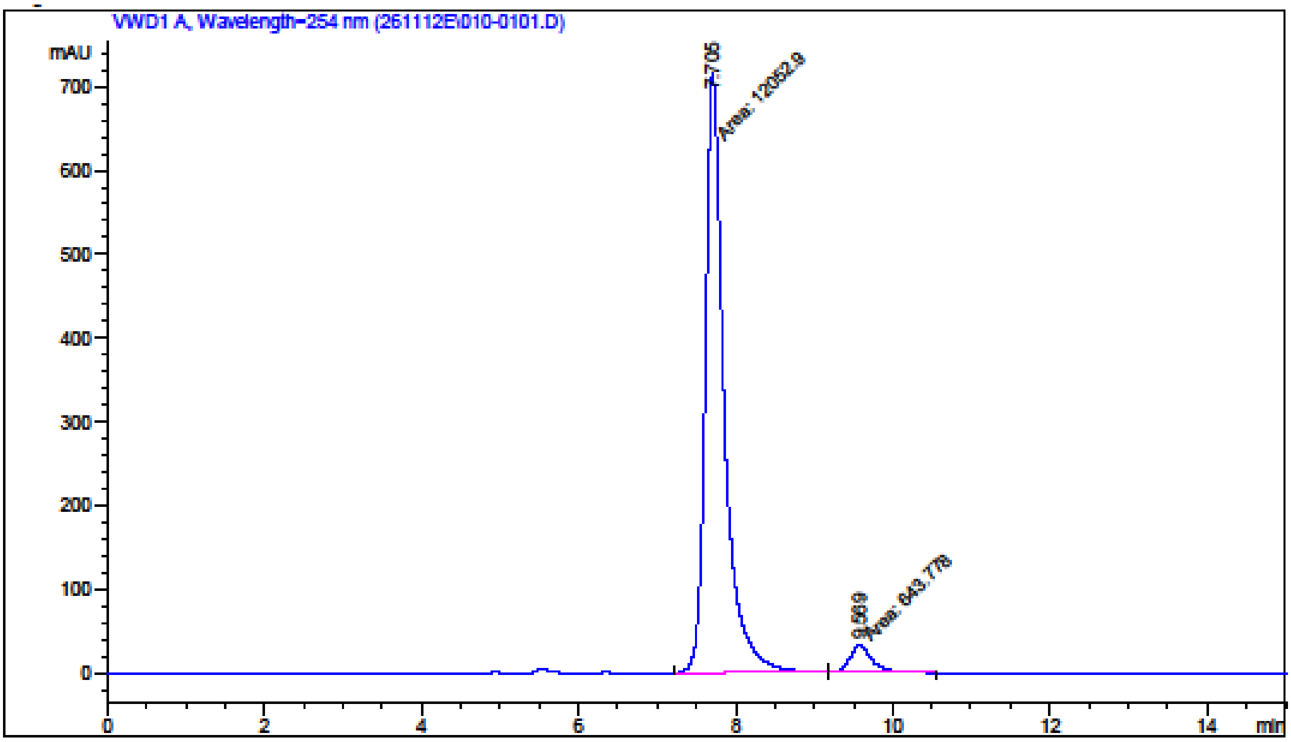

Peak #	RetTime [min]	Type	Width [min]	Area [mAU*s]	Height [mAU]	Area %
1	7.705	MM	0.2800	1.20529e4	717.54108	94.9296
2	9.569	MM	0.3319	643.77808	32.33134	5.0704

90%*ee*

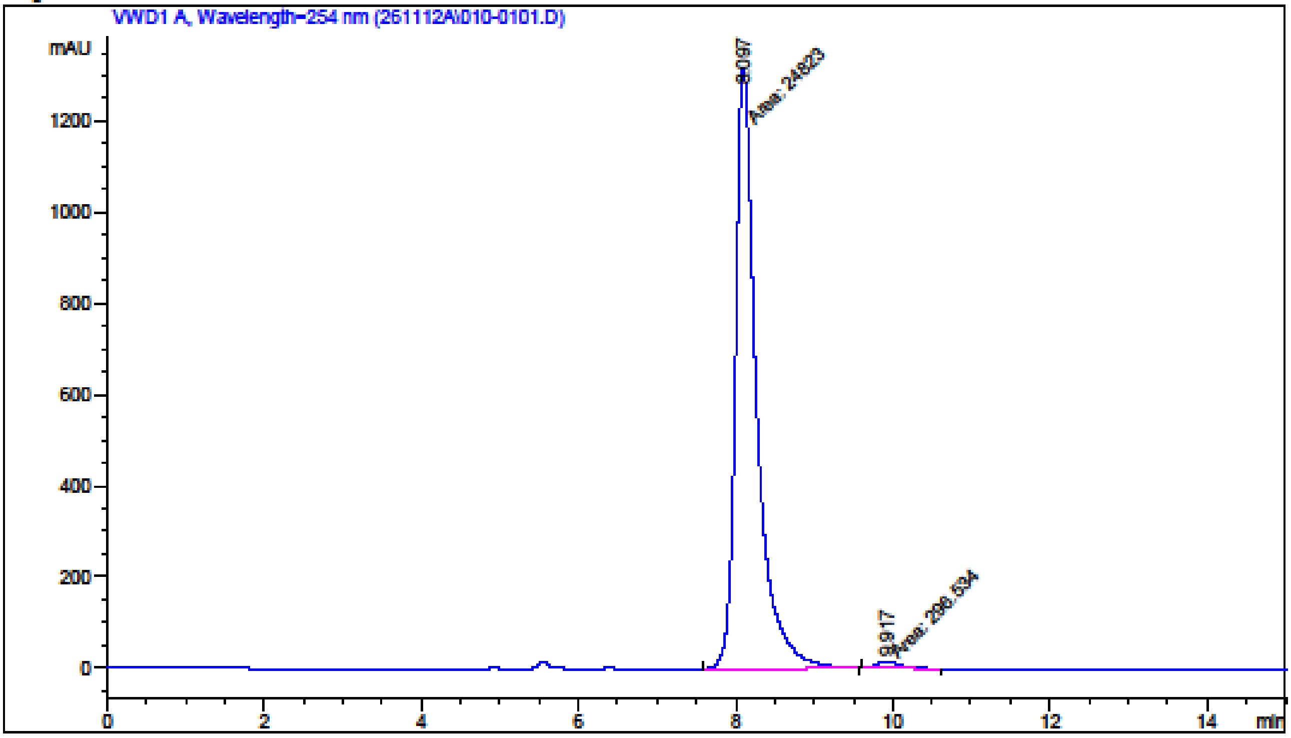

Peak #	RetTime [min]	Type	Width [min]	Area [mAU*s]	Height [mAU]	Area %
1	8.097	MM	0.3136	2.48230e4	1319.44849	98.8195
2	9.917	MM	0.3467	296.53366	14.25321	1.1805

98%*ee* (after recrystallisation)

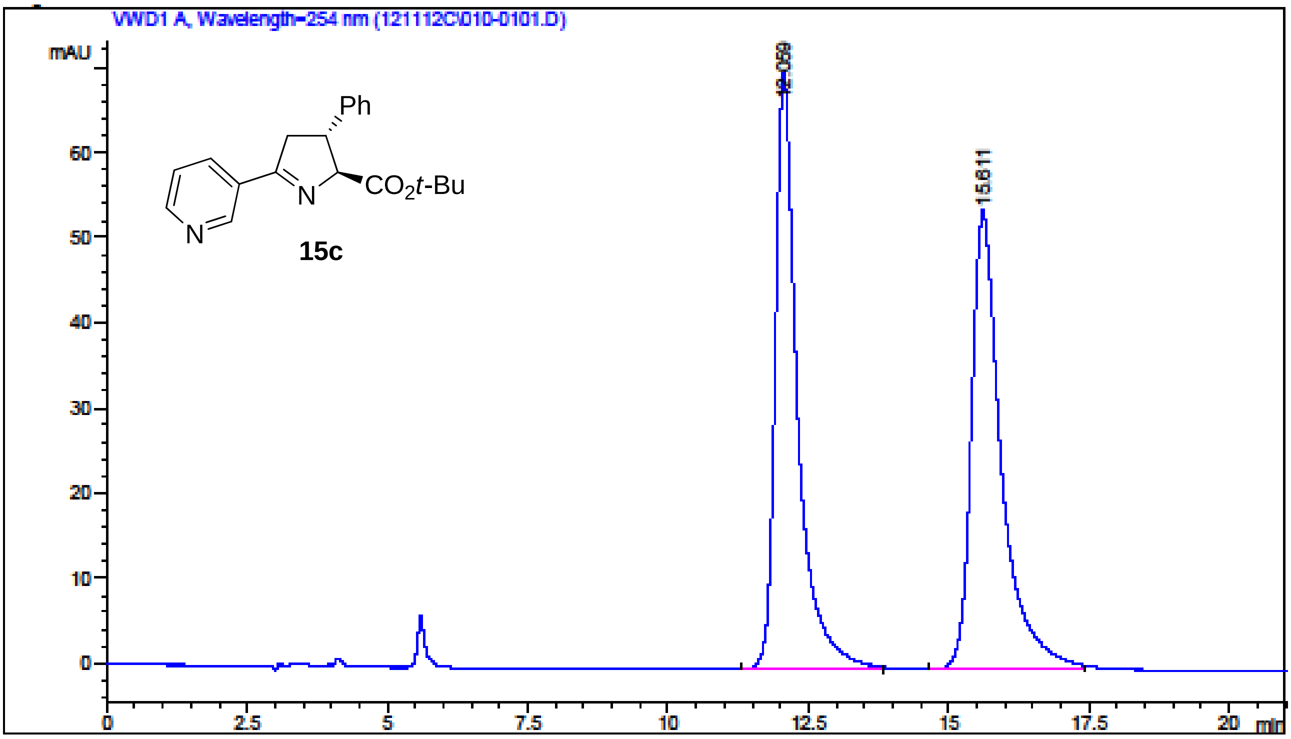

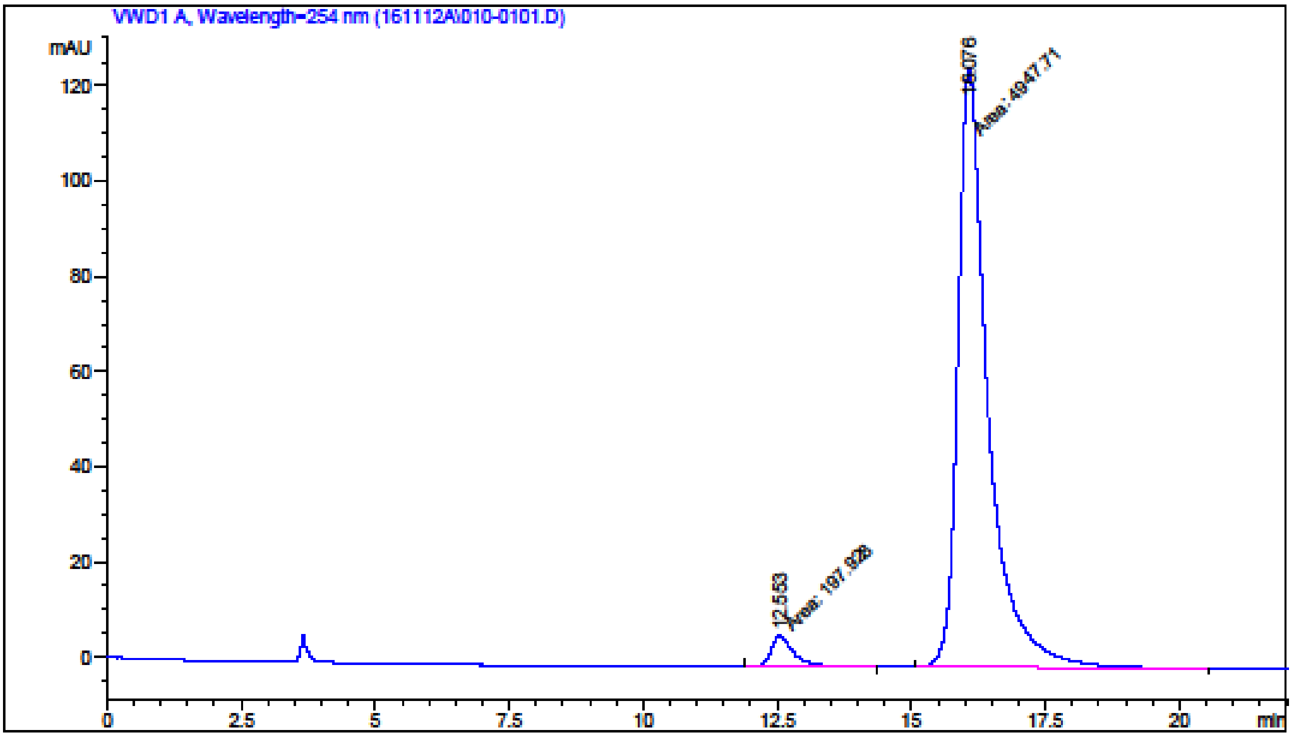

Peak #	RetTime [min]	Type	Width [min]	Area [mAU*s]	Height [mAU]	Area %
1	12.553	MM	0.5024	197.92775	6.56560	3.8465
2	16.076	MM	0.6550	4947.70801	125.90186	96.1535

92%*ee*

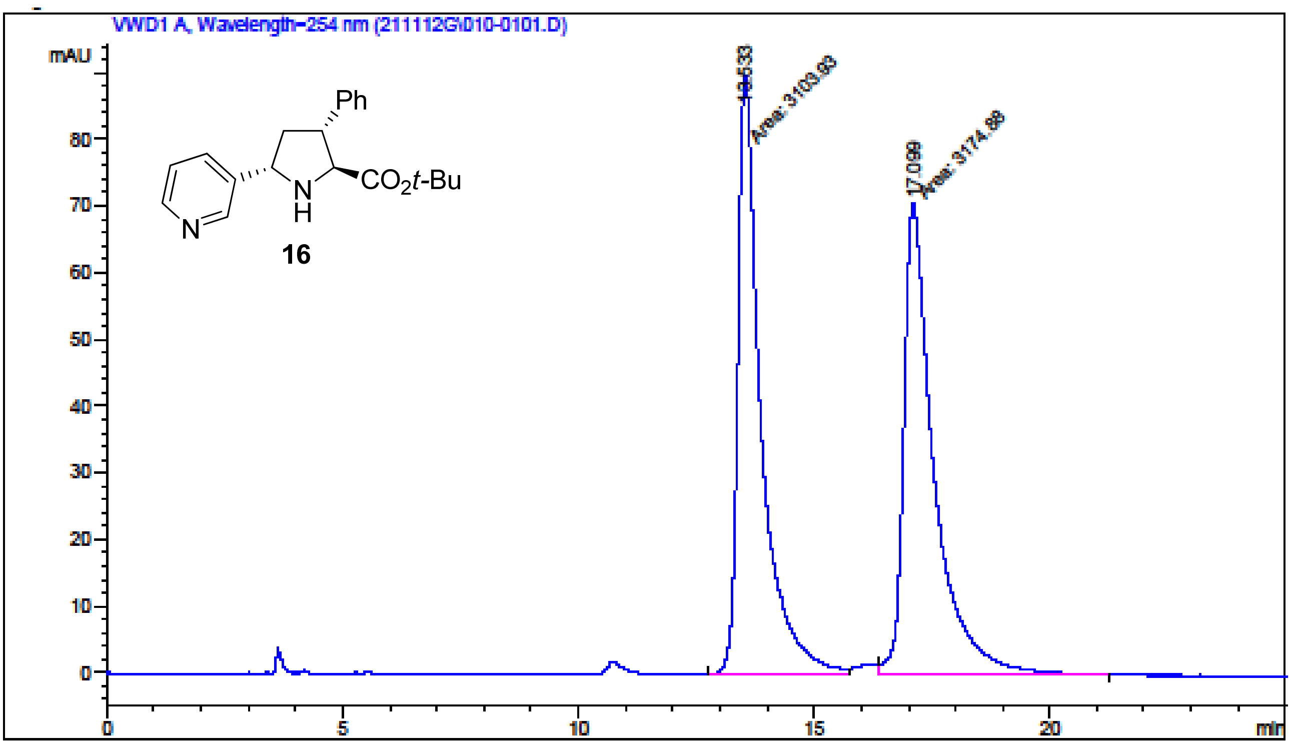

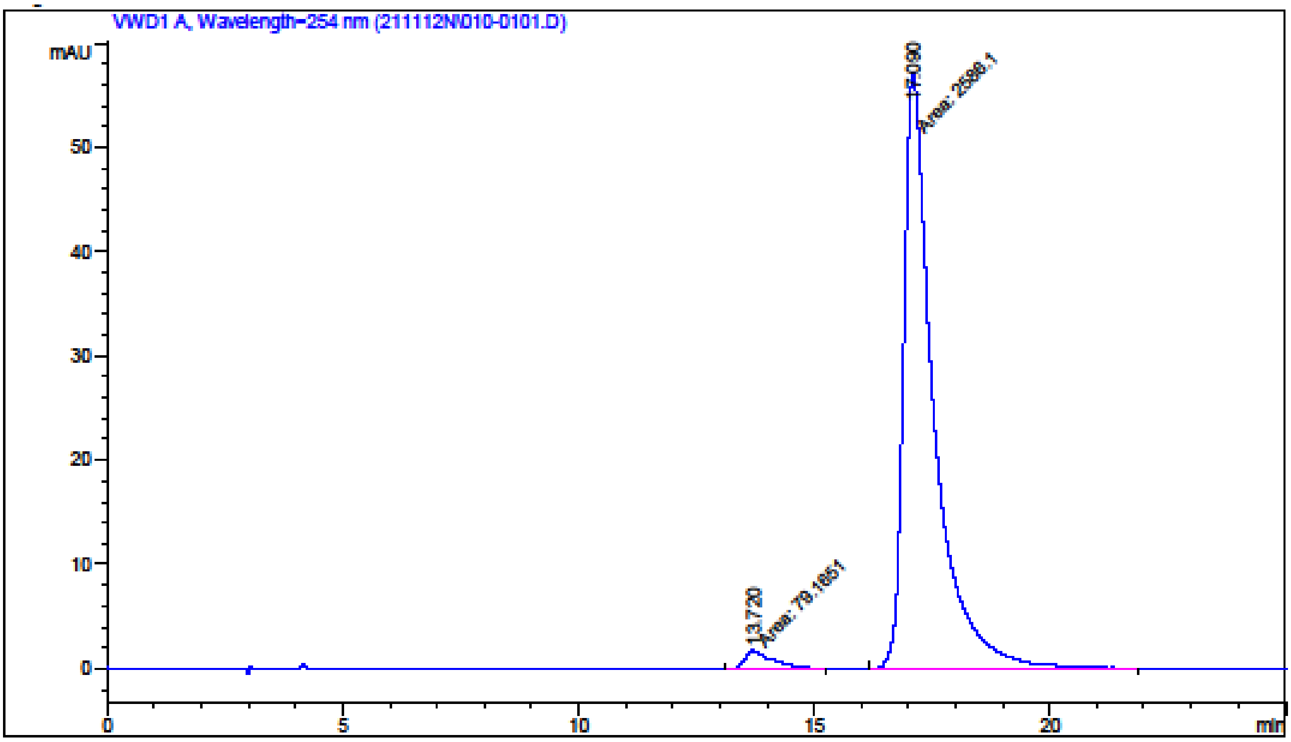

Peak #	RetTime [min]	Type	Width [min]	Area [mAU*s]	Height [mAU]	Area %
1	13.720	MM	0.7284	79.16513	1.81133	2.9703
2	17.090	MM	0.7526	2586.09888	57.26675	97.0297

94%*ee*

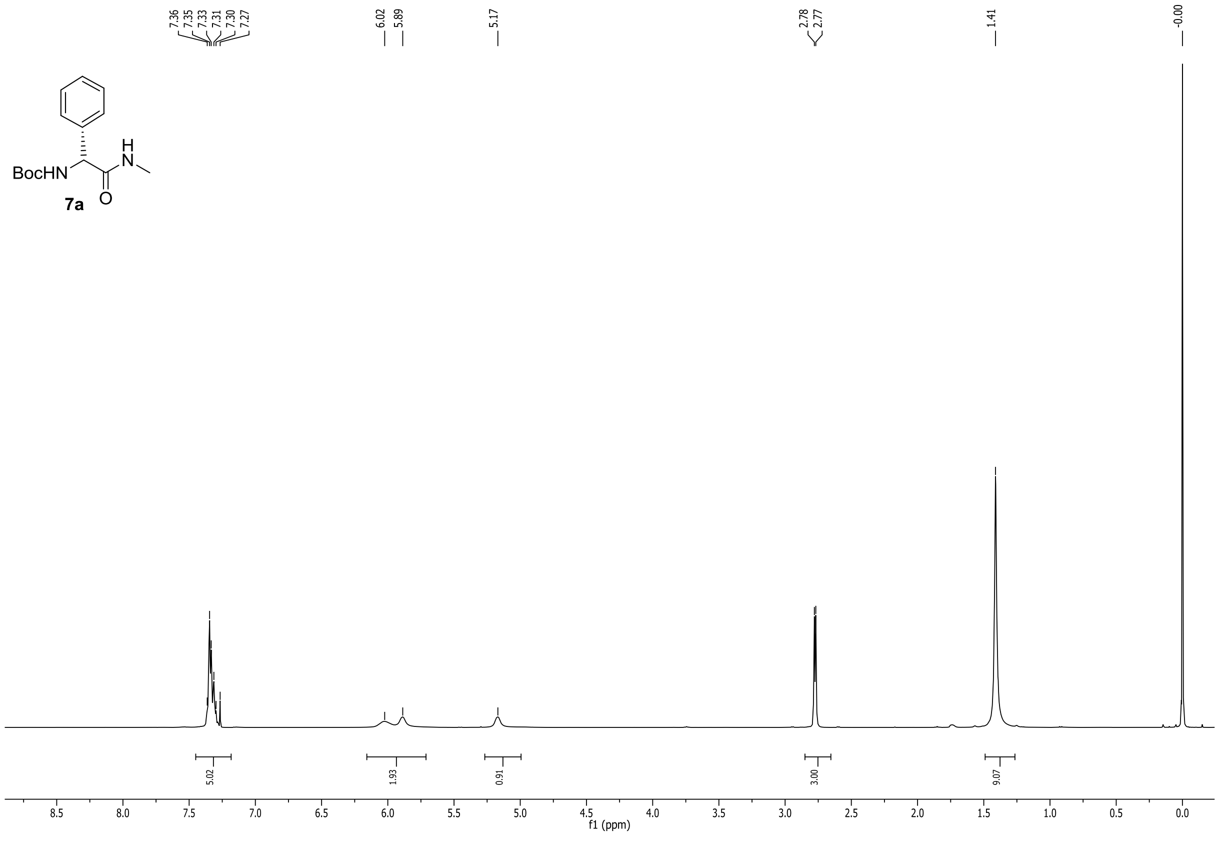

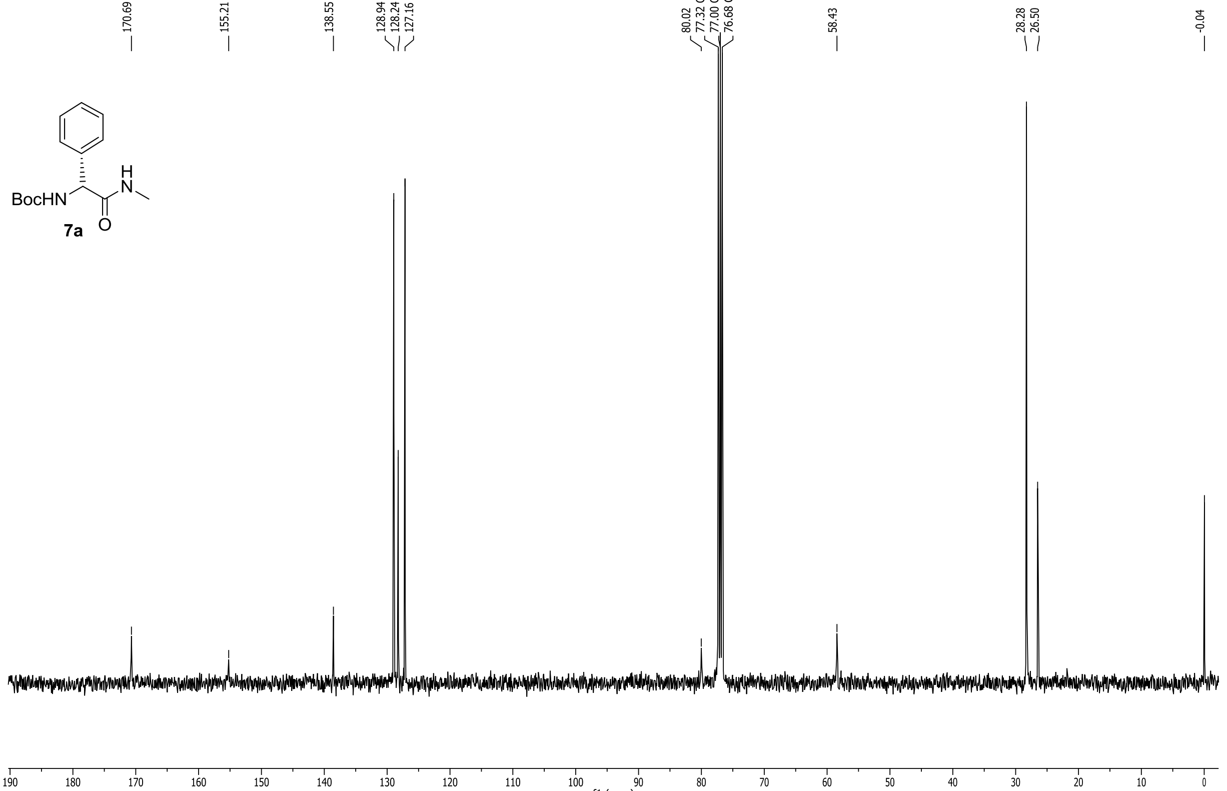

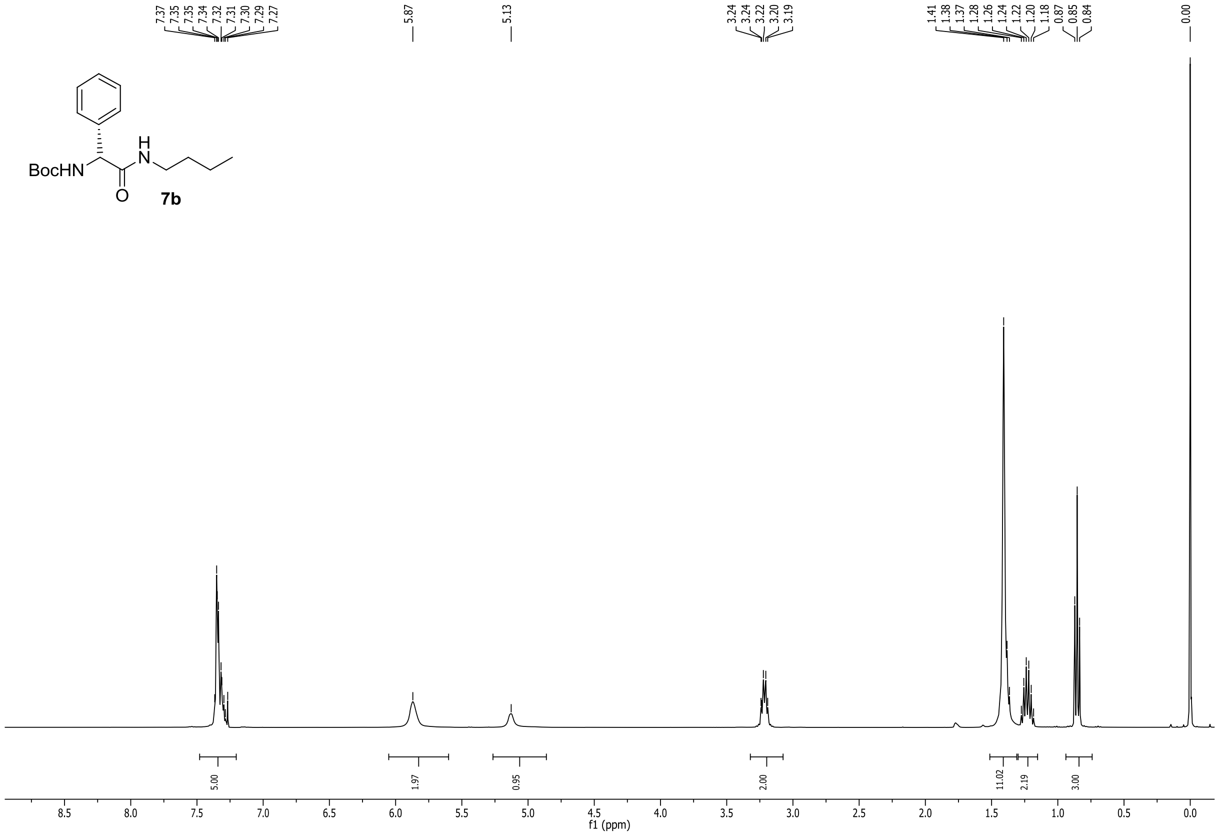
7.37 7.35 7.35 7.34 7.32 7.31 7.30 7.29 7.27
5.87
5.13
3.24 3.24 3.22 3.20 3.19
1.41 1.38 1.37 1.28 1.26 1.24 1.22 1.20 1.18 0.87 0.85 0.84
0.00
BocHN
H
N
O
7b
5.00
1.97
0.95
2.00
11.02
2.19
3.00
f1 (ppm)

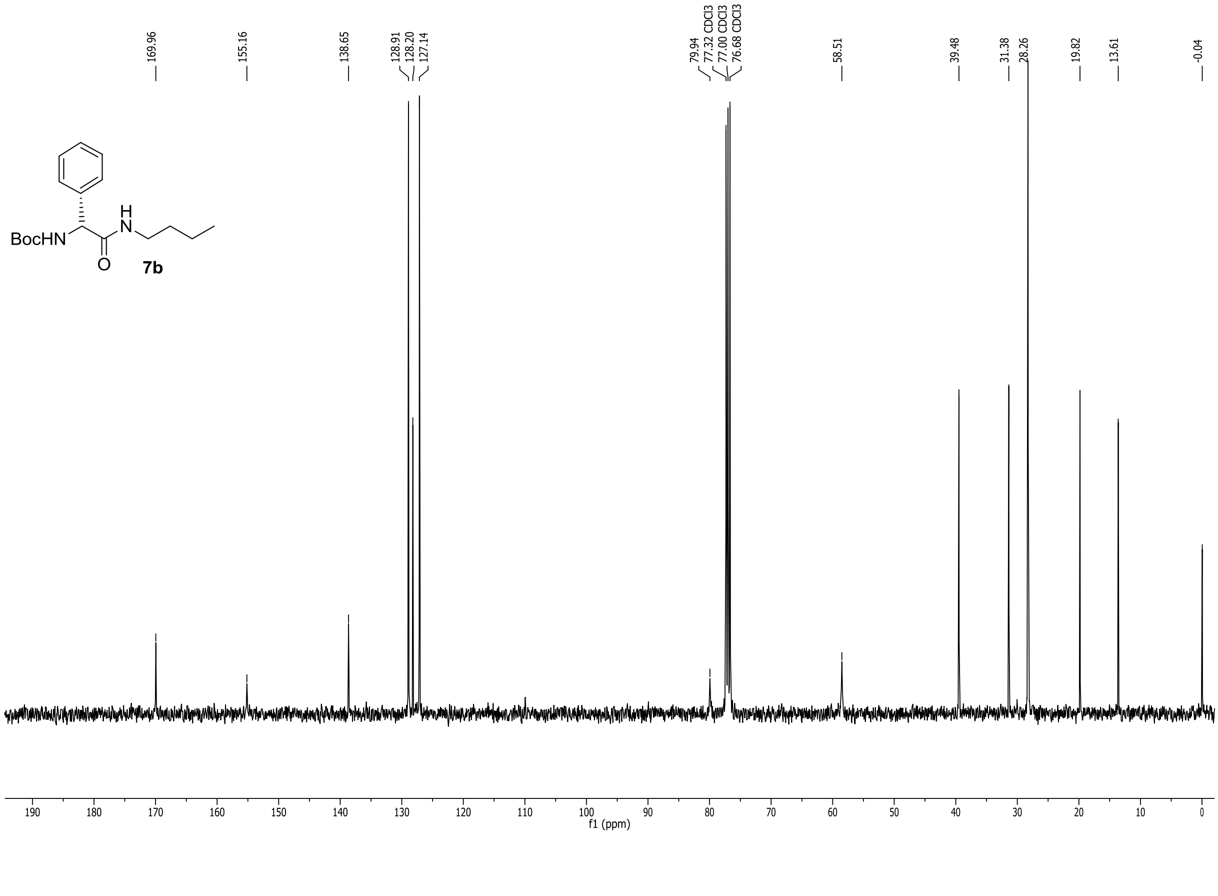
169.96
155.16
138.65
128.91 128.20 127.14
79.94 77.32 CDCl3 77.00 CDCl3 76.68 CDCl3
58.51
39.48
31.38 28.26
19.82
13.61
-0.04
BocHN
H
N
O
7b
f1 (ppm)

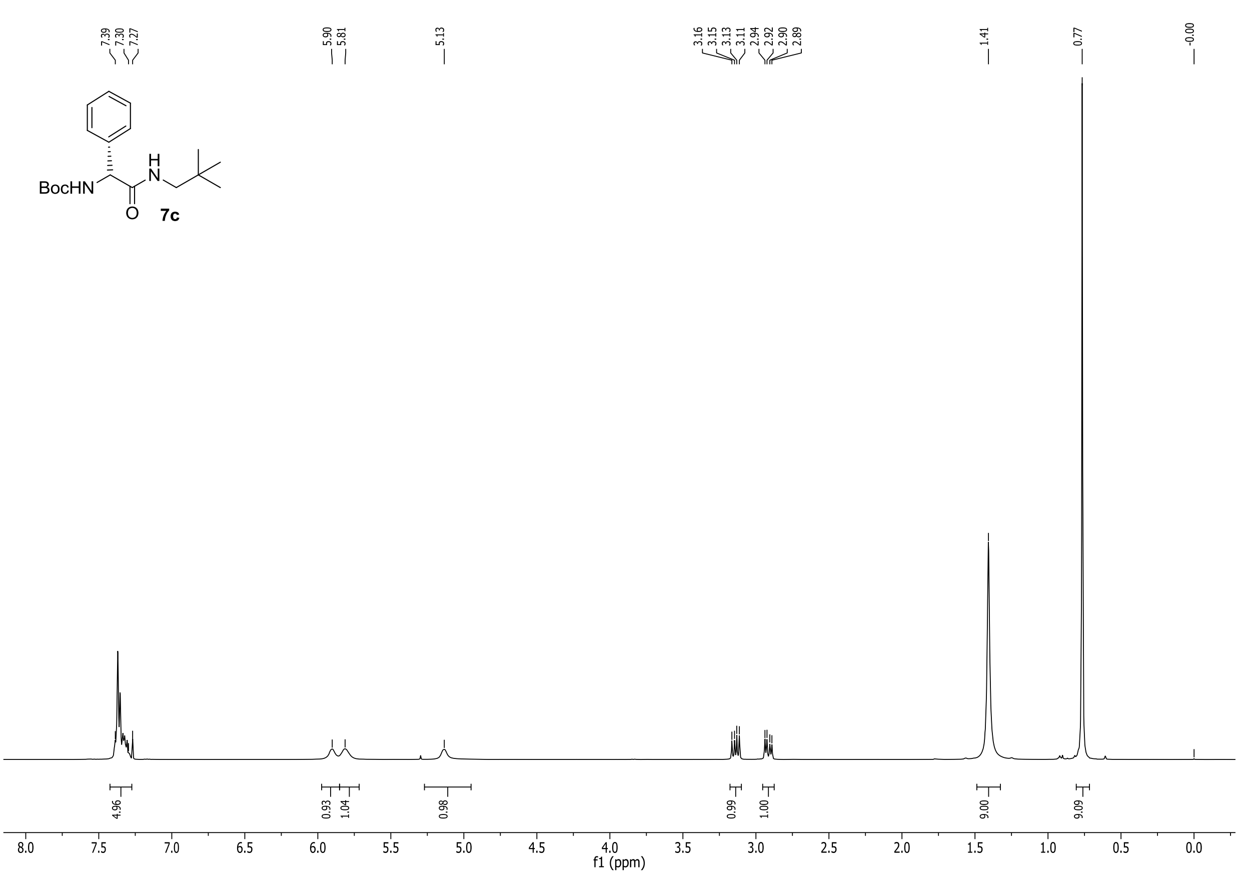
7.39
7.30
7.27
5.90
5.81
5.13
3.16
3.15
3.13
3.11
2.94
2.92
2.90
2.89
1.41
0.77
-0.00
BocHN
7c
4.96
0.93
1.04
0.98
0.99
1.00
9.00
9.09
f1 (ppm)

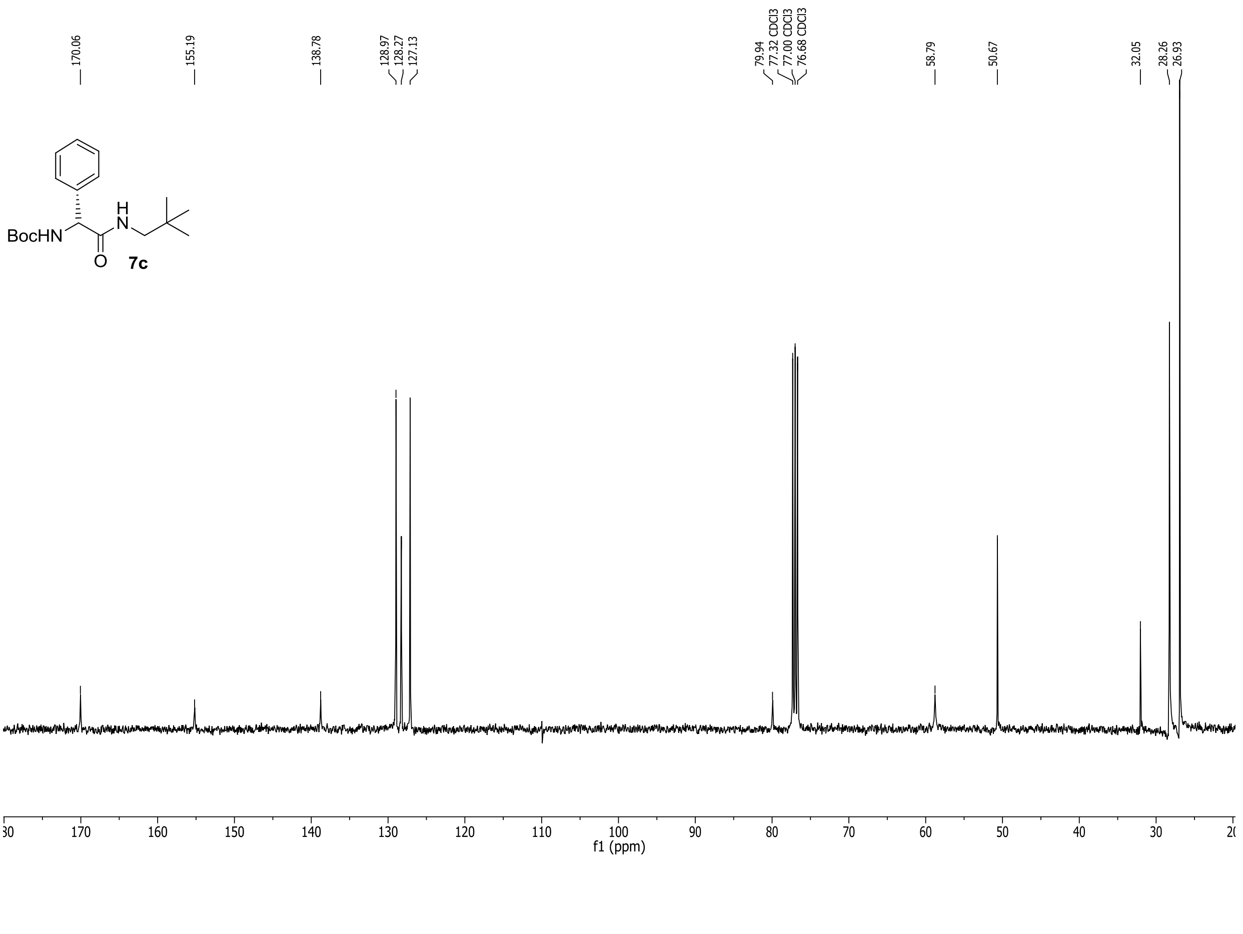
170.06
155.19
138.78
128.97
128.27
127.13
79.94
77.32 CDCl3
77.00 CDCl3
76.68 CDCl3
58.79
50.67
32.05
28.26
26.93
BocHN
7c
f1 (ppm)

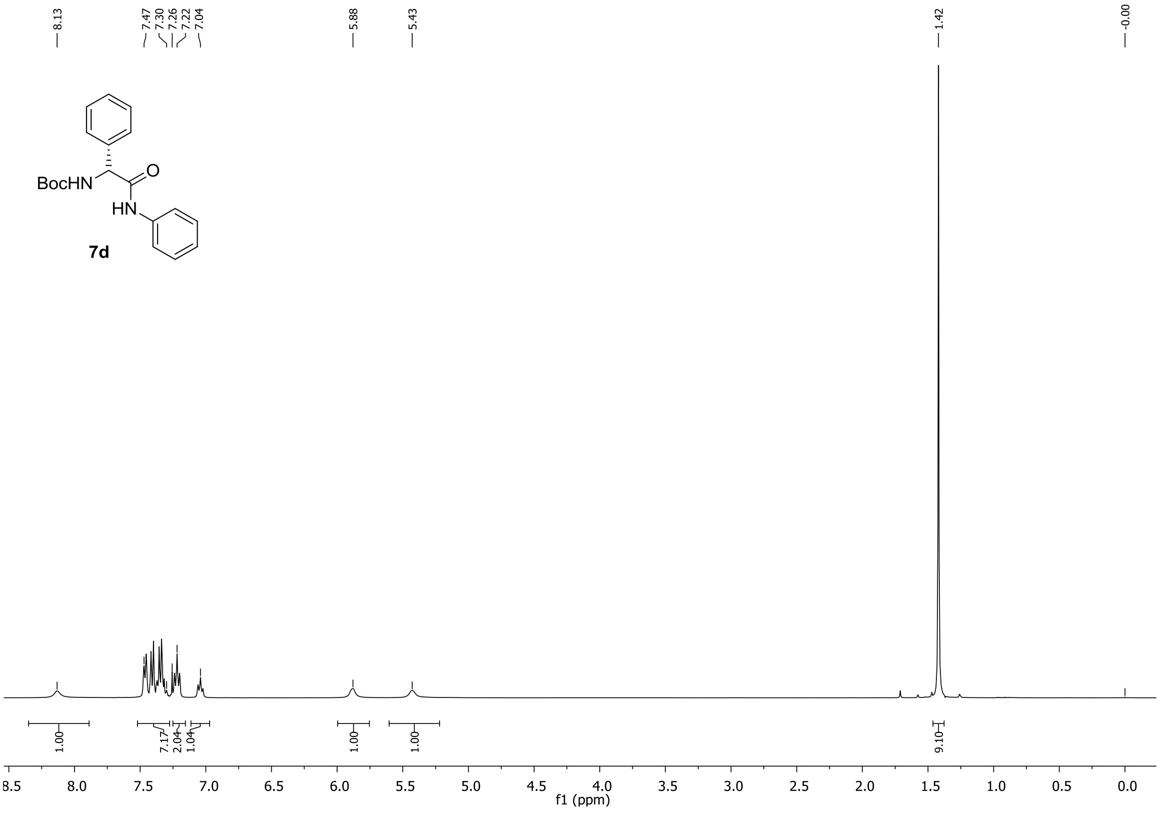
8.13
7.47
7.30
7.26
7.22
7.04
5.88
5.43
1.42
-0.00
BocHN
O
HN
7d
1.00
7.17
2.04
1.04
1.00
1.00
9.10
f1 (ppm)

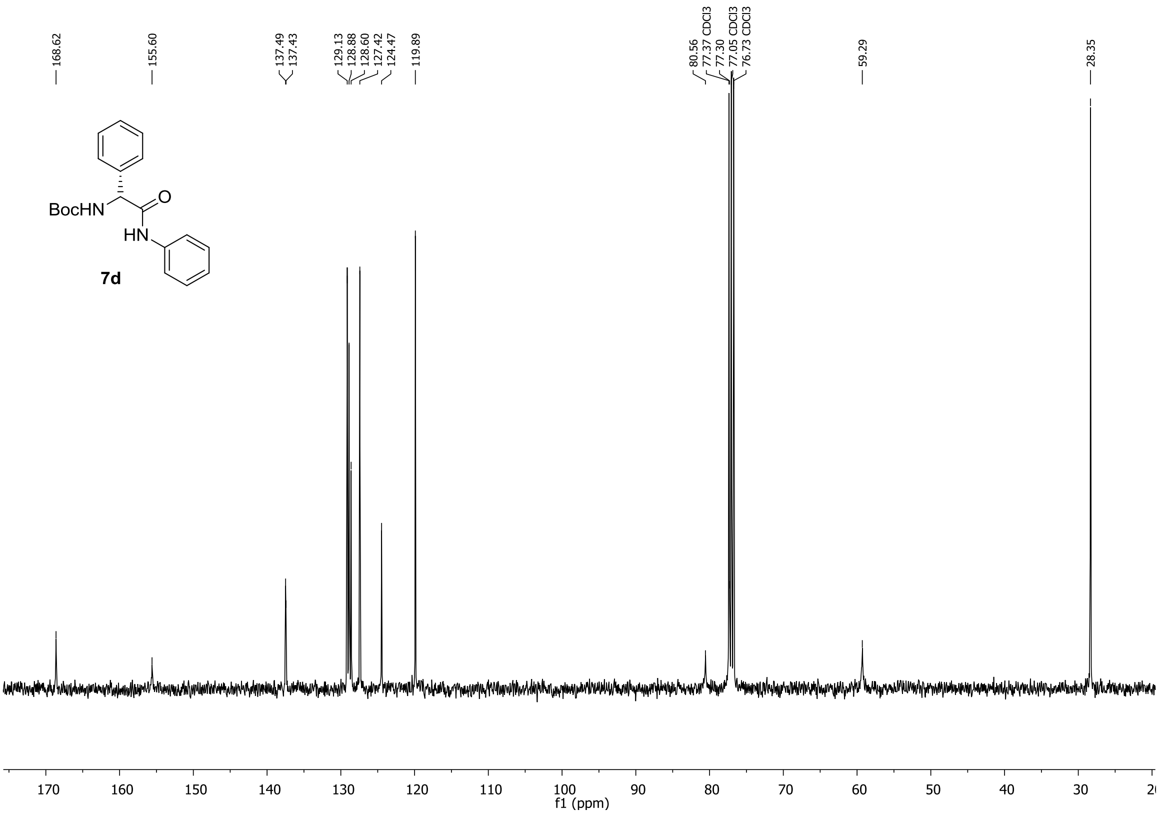
168.62
155.60
137.49
137.43
129.13
128.88
128.60
127.42
124.47
119.89
80.56
77.37 CDCl3
77.30
77.05 CDCl3
76.73 CDCl3
59.29
28.35
BocHN
O
HN
7d
f1 (ppm)

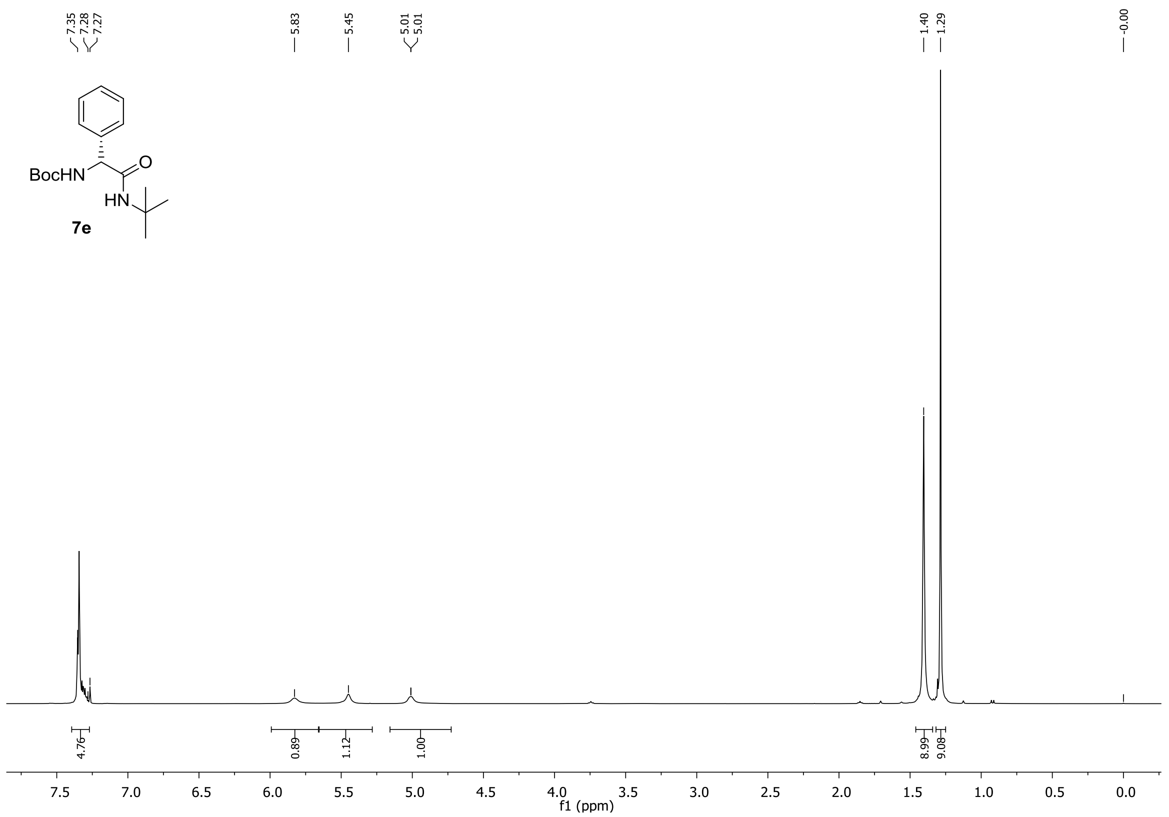
7.35
7.28
7.27
5.83
5.45
5.01
5.01
1.40
1.29
-0.00
BocHN
O
HN
7e
4.76
0.89
1.12
1.00
8.99
9.08
f1 (ppm)

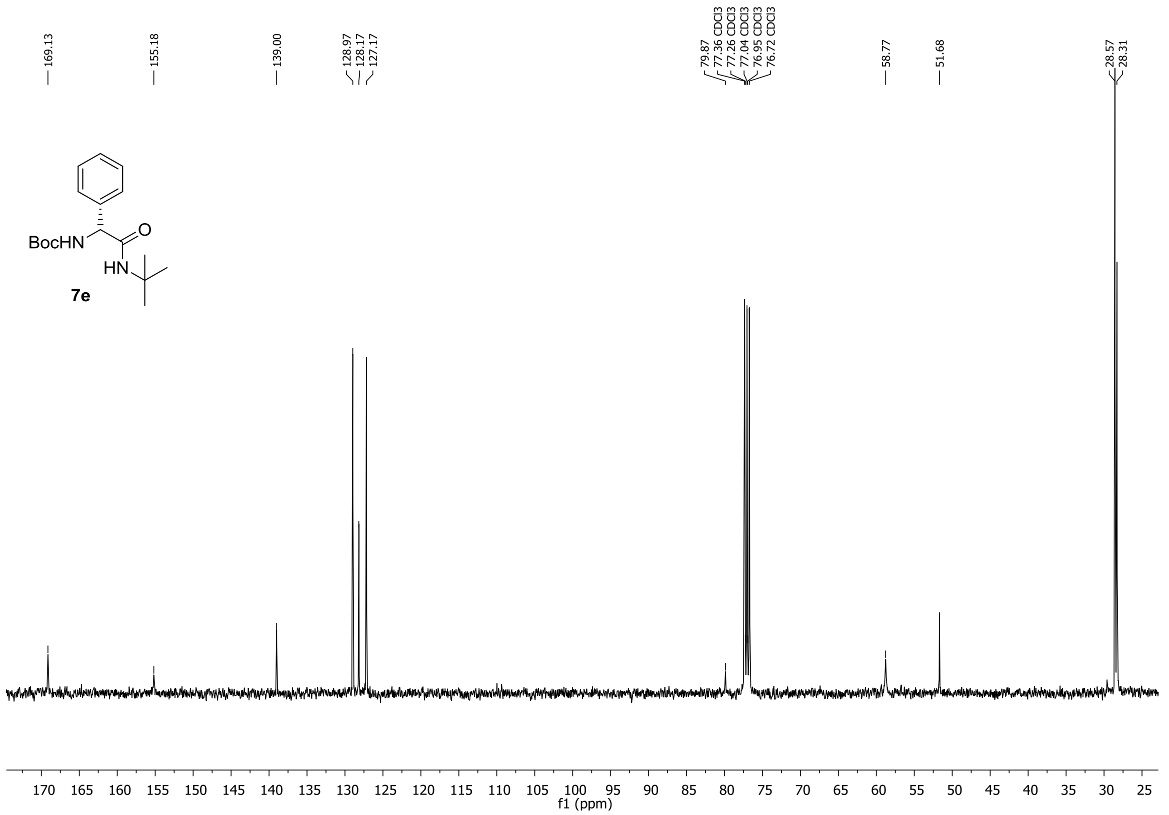
169.13
155.18
139.00
128.97
128.17
127.17
79.87
77.36 CDCl3
77.26 CDCl3
77.04 CDCl3
76.95 CDCl3
76.72 CDCl3
58.77
51.68
28.57
28.31
BocHN
O
HN
7e
f1 (ppm)

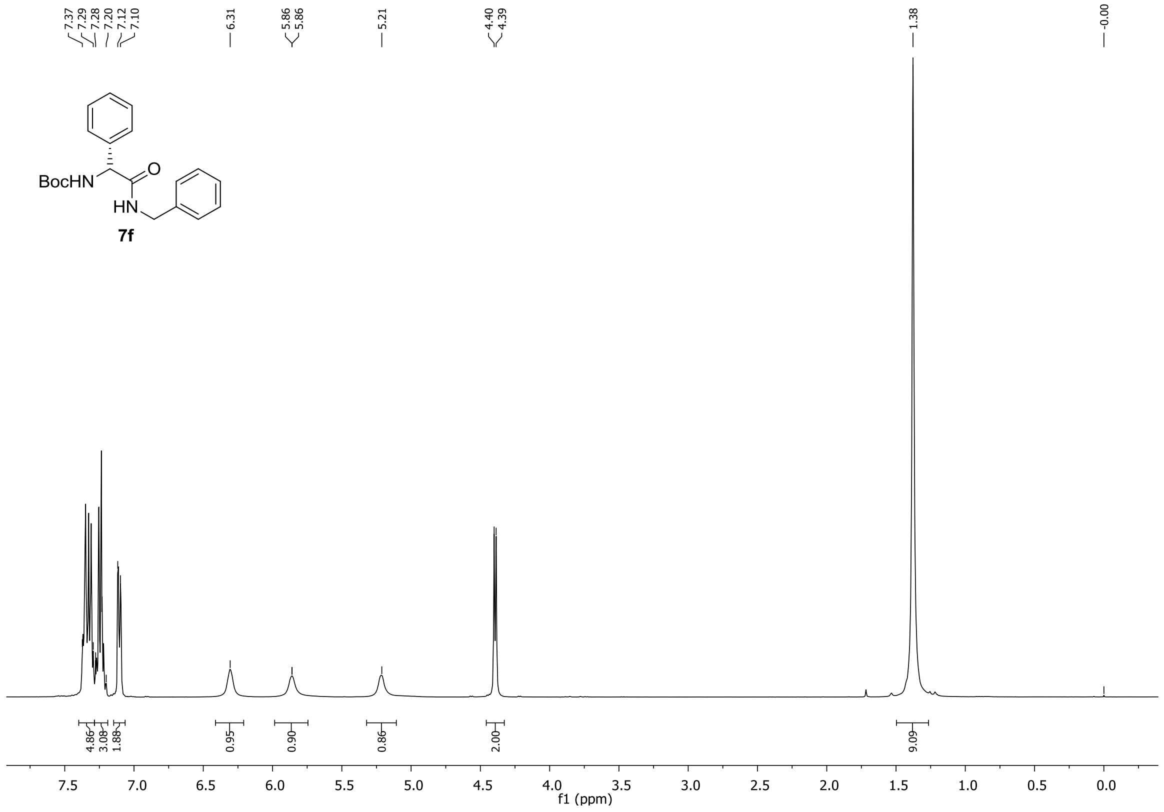
7.37
7.29
7.28
7.20
7.12
7.10
6.31
5.86
5.86
5.21
4.40
4.39
1.38
-0.00
BocHN
O
HN
7f
4.86
3.08
1.88
0.95
0.90
0.86
2.00
9.09
7.5 7.0 6.5 6.0 5.5 5.0 4.5 4.0 3.5 3.0 2.5 2.0 1.5 1.0 0.5 0.0
f1 (ppm)

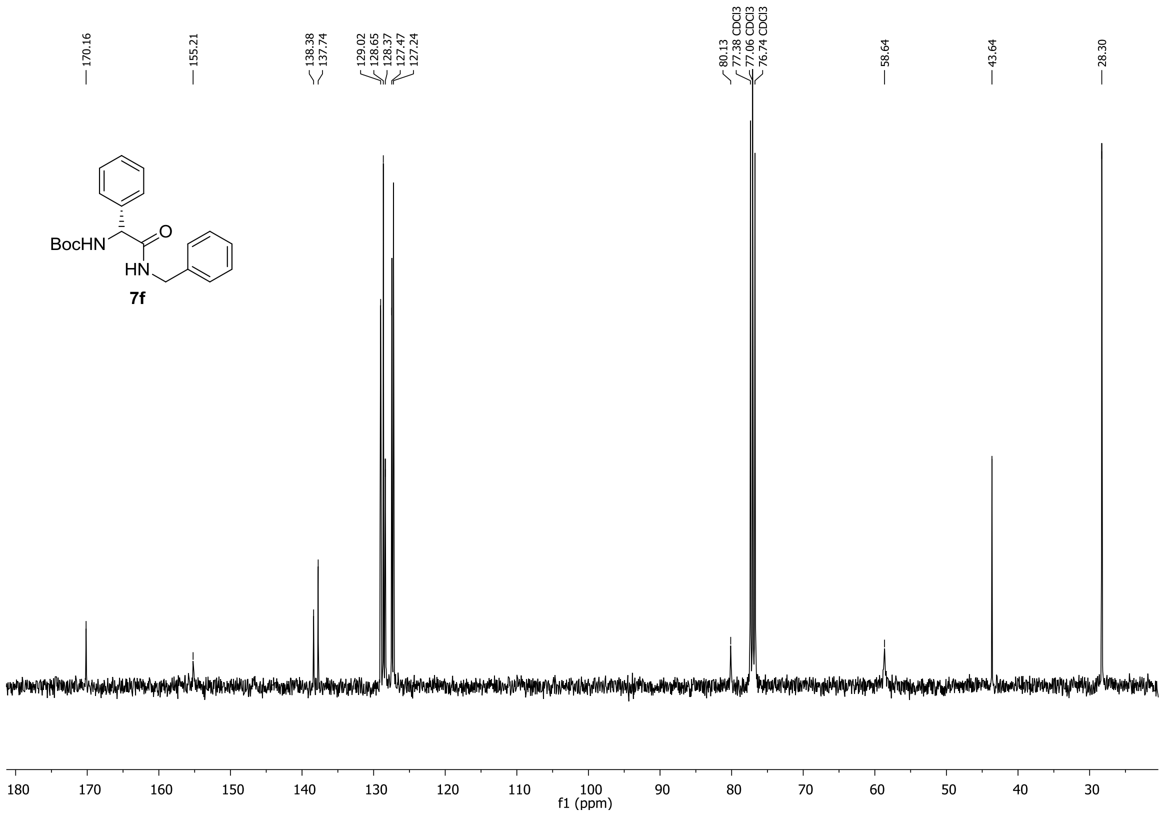
170.16
155.21
138.38
137.74
129.02
128.65
128.37
127.47
127.24
80.13
77.38 CDCl3
77.06 CDCl3
76.74 CDCl3
58.64
43.64
28.30
BocHN
O
HN
7f
180 170 160 150 140 130 120 110 100 90 80 70 60 50 40 30
f1 (ppm)

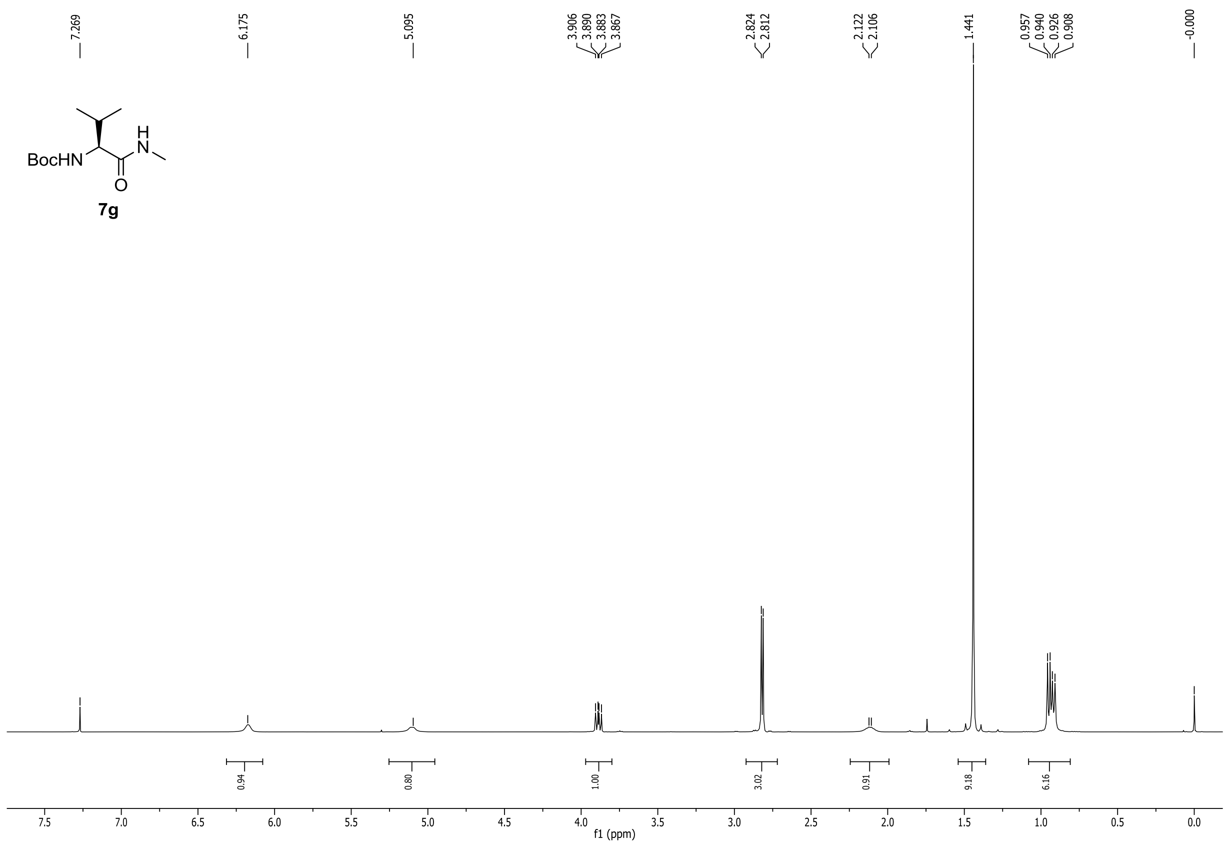

7.269
6.175
5.095
3.906
3.890
3.883
3.867
2.824
2.812
2.122
2.106
1.441
0.957
0.940
0.926
0.908
-0.000
BocHN
H
N
O
7g
0.94
0.80
1.00
3.02
0.91
9.18
6.16
f1 (ppm)

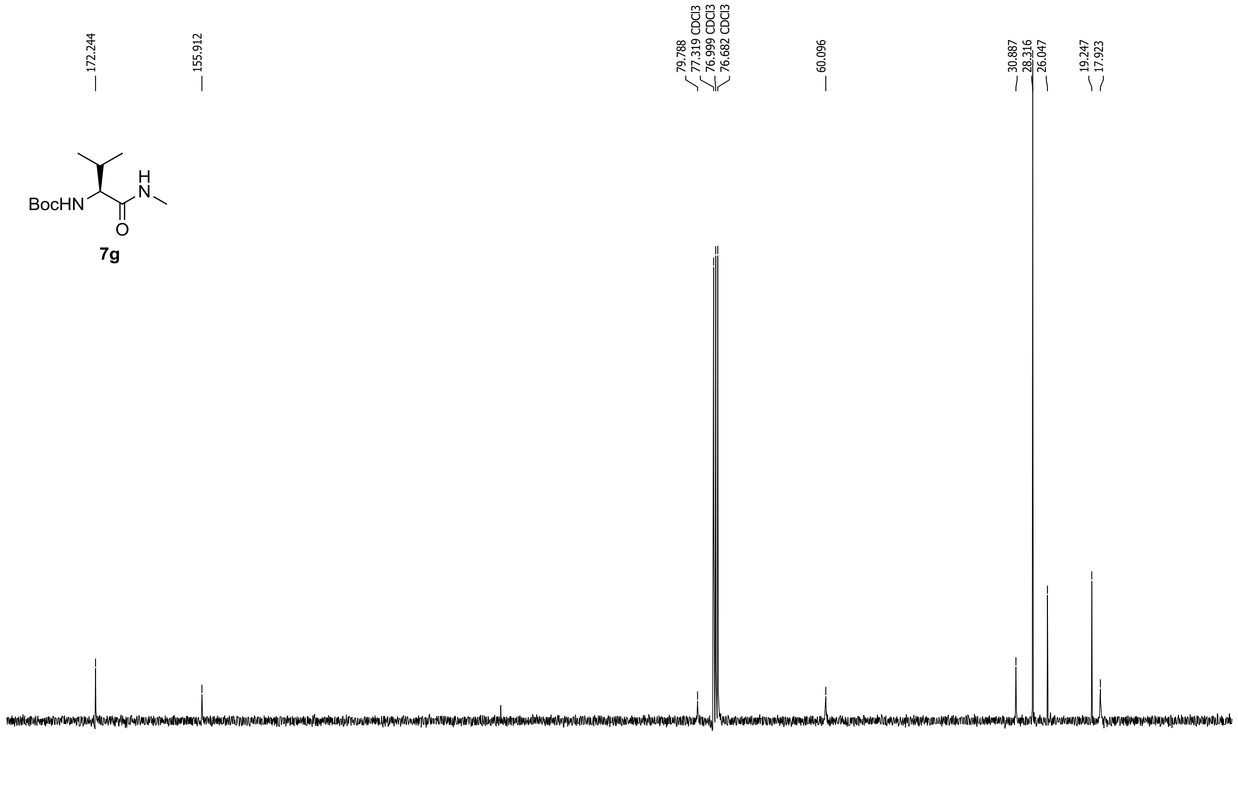

172.244
155.912
79.788
77.319 CDCl3
76.999 CDCl3
76.682 CDCl3
60.096
30.887
28.316
26.047
19.247
17.923
BocHN
H
N
O
7g
f1 (ppm)

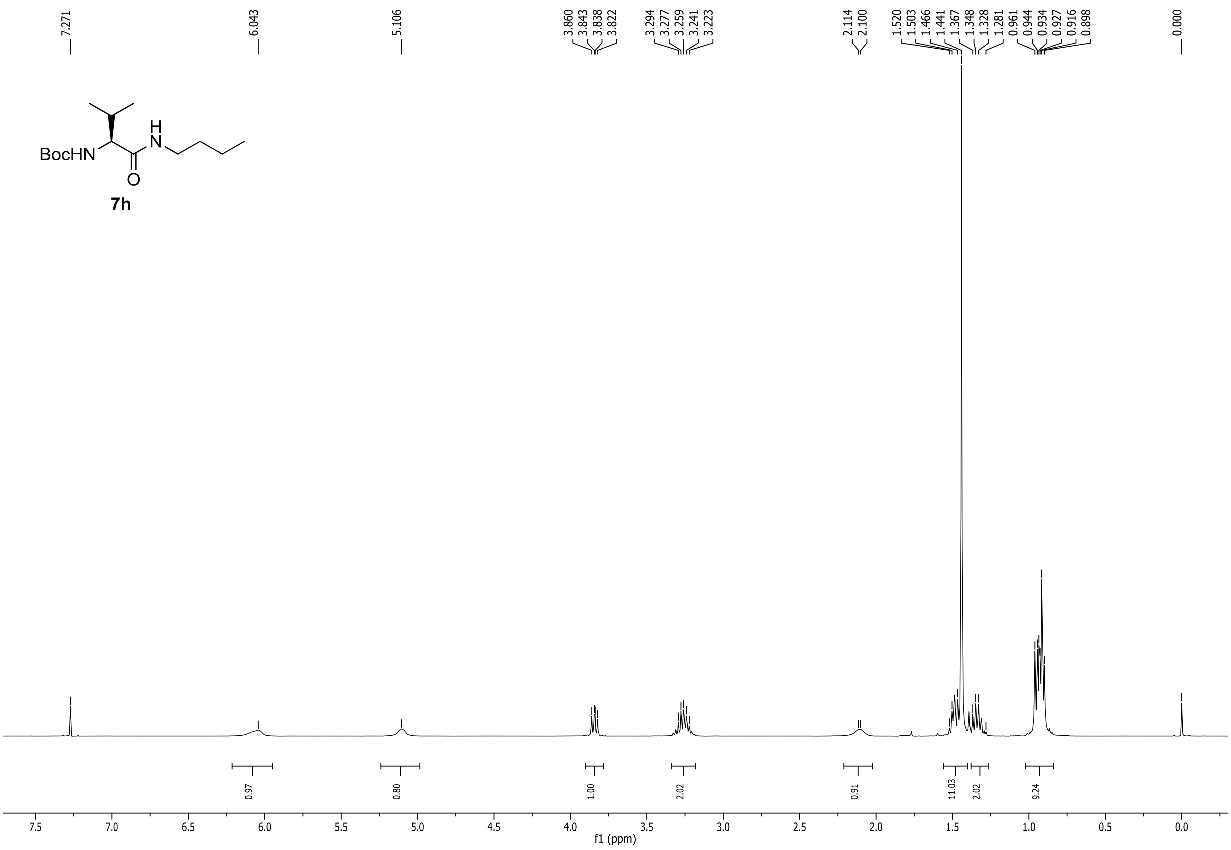
7.271
6.043
5.106
3.860 3.843 3.838 3.822
3.294 3.277 3.259 3.241 3.223
2.114 2.100
1.520 1.503 1.466 1.441 1.367 1.348 1.328 1.281 0.961 0.944 0.934 0.927 0.916 0.898
0.000
BocHN
H
N
O
7h
0.97
0.80
1.00
2.02
0.91
11.03
2.02
9.24
f1 (ppm)

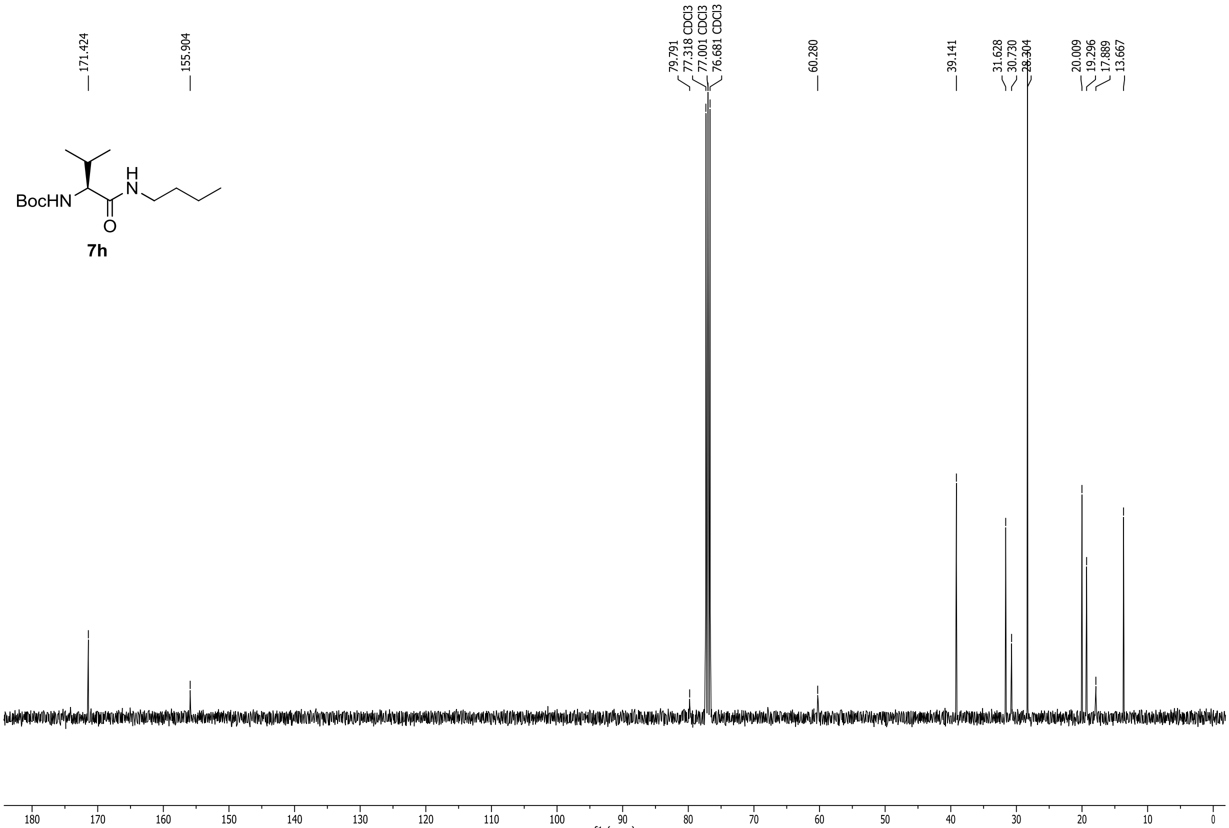
171.424
155.904
79.791
77.318 CDCl3
77.001 CDCl3
76.681 CDCl3
60.280
39.141
31.628
30.730
28.304
20.009
19.296
17.889
13.667
BocHN
H
N
O
7h
f1 (ppm)

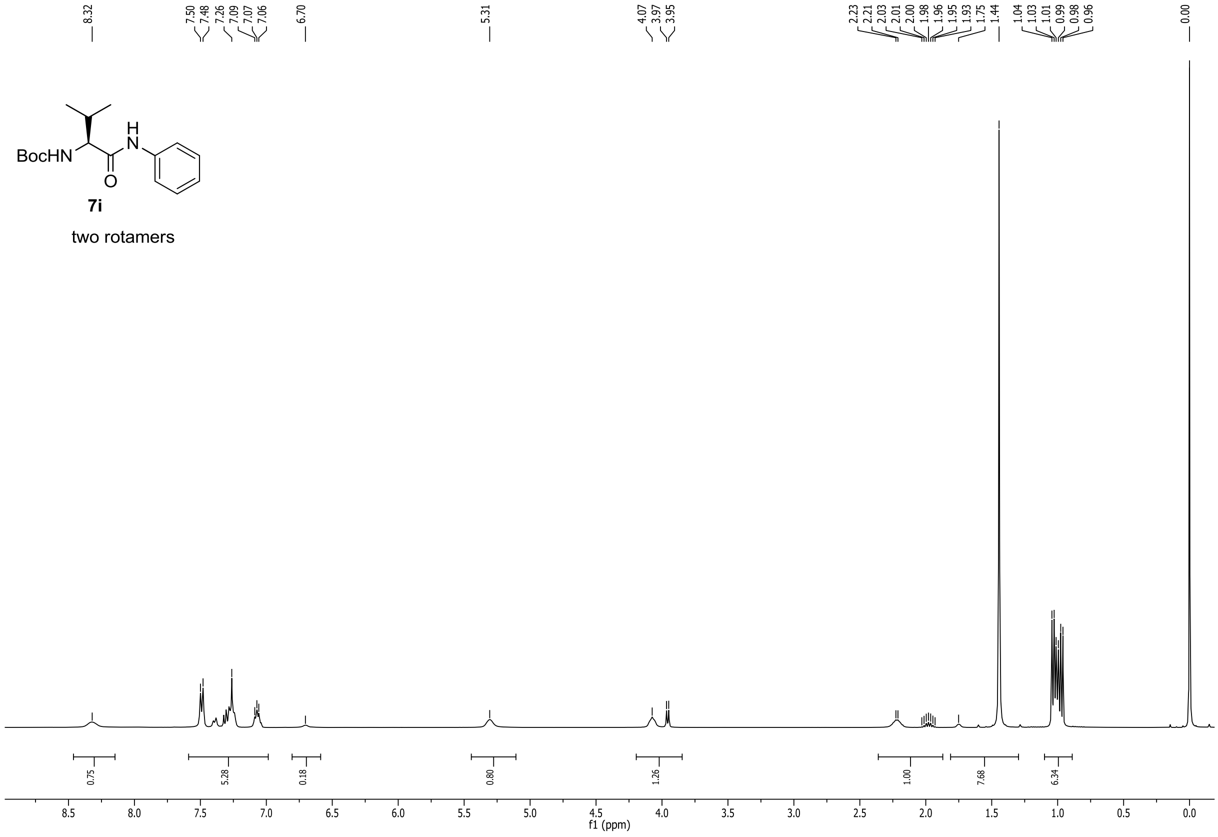
8.32
7.50
7.48
7.26
7.09
7.07
7.06
6.70
5.31
4.07
3.97
3.95
2.23
2.21
2.03
2.01
2.00
1.98
1.96
1.95
1.93
1.75
1.44
1.04
1.03
1.01
0.99
0.98
0.96
0.00
BocHN
H
N
O
7i
two rotamers
0.75
5.28
0.18
0.80
1.26
1.00
7.68
6.34
f1 (ppm)

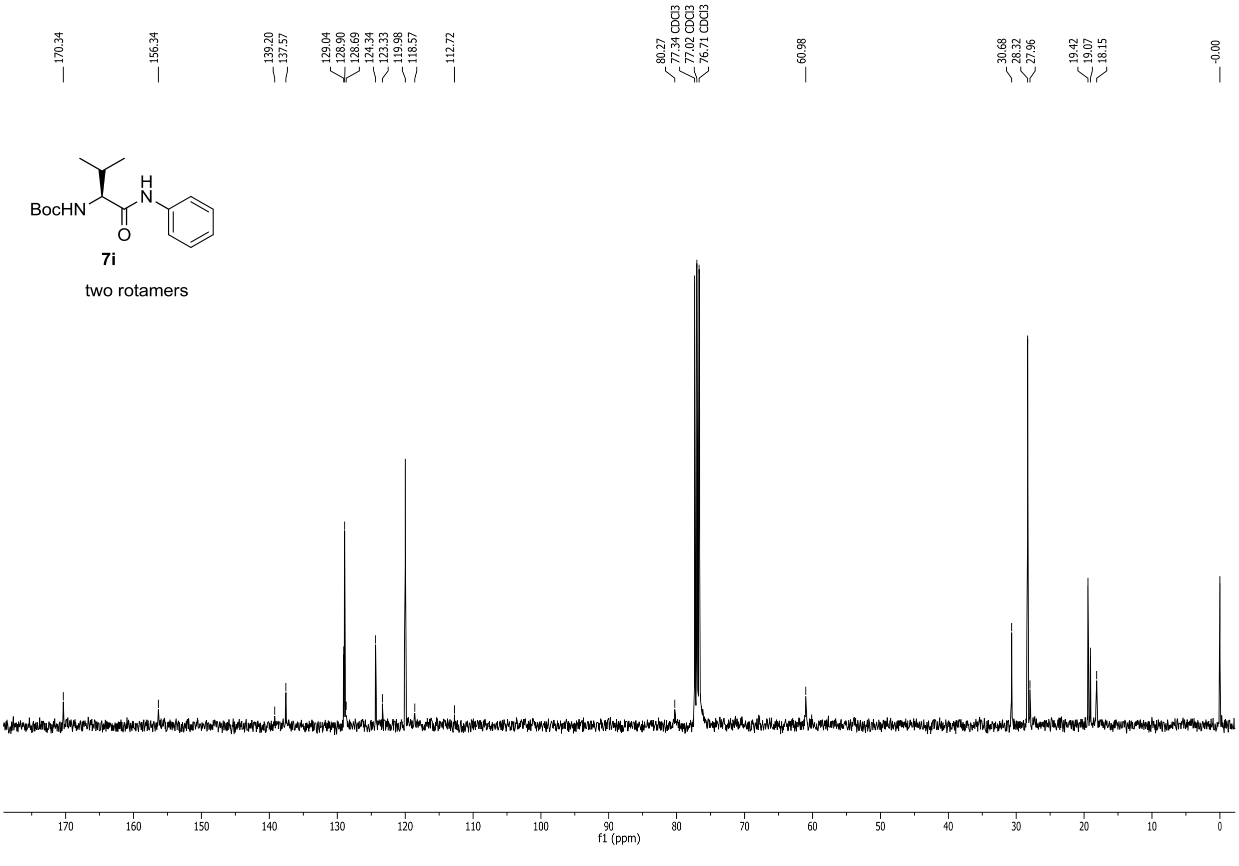
170.34
156.34
139.20
137.57
129.04
128.90
128.69
124.34
123.33
119.98
118.57
112.72
80.27
77.34 CDCl3
77.02 CDCl3
76.71 CDCl3
60.98
30.68
28.32
27.96
19.42
19.07
18.15
-0.00
BocHN
H
N
O
7i
two rotamers
f1 (ppm)

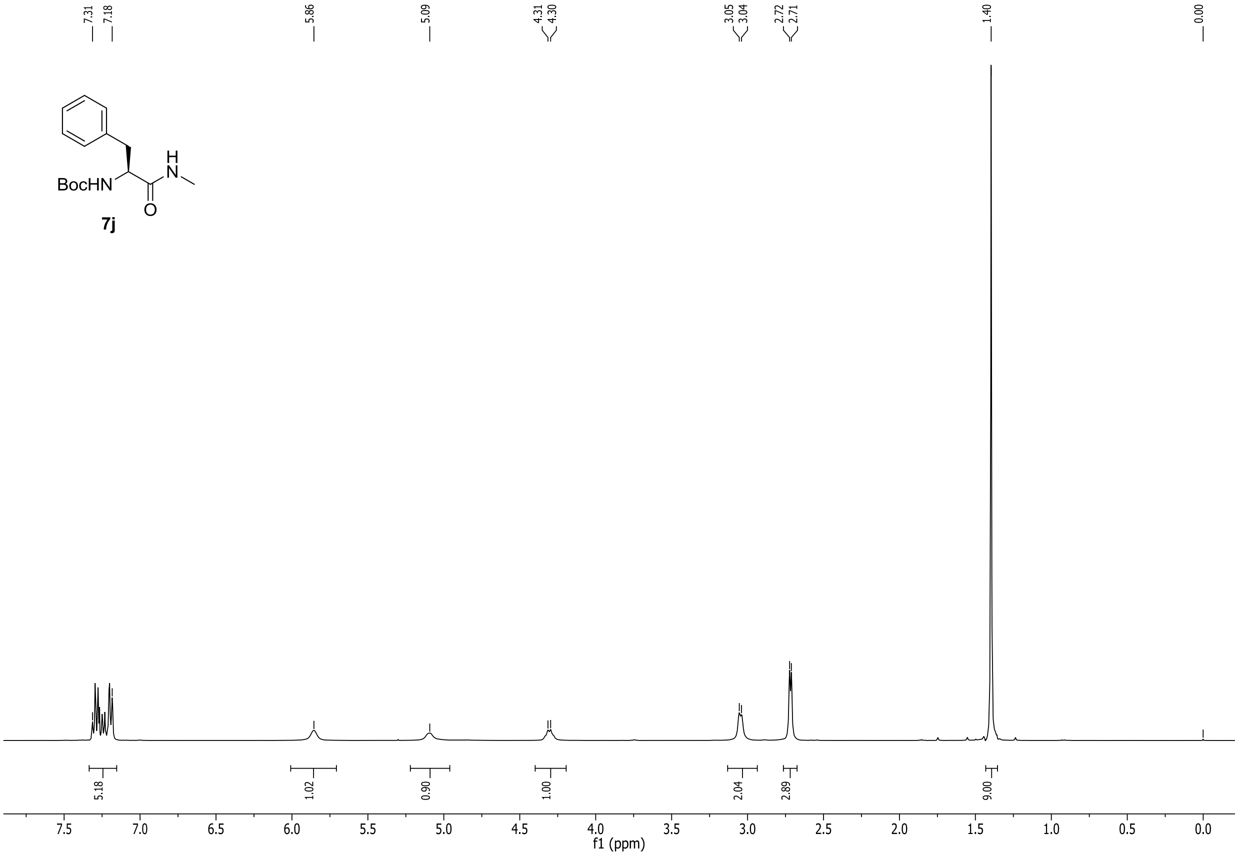
7.31
7.18
5.86
5.09
4.31
4.30
3.05
3.04
2.72
2.71
1.40
0.00
BocHN
H
N
O
7j
5.18
1.02
0.90
1.00
2.04
2.89
9.00
7.5
7.0
6.5
6.0
5.5
5.0
4.5
4.0
3.5
3.0
2.5
2.0
1.5
1.0
0.5
0.0
f1 (ppm)

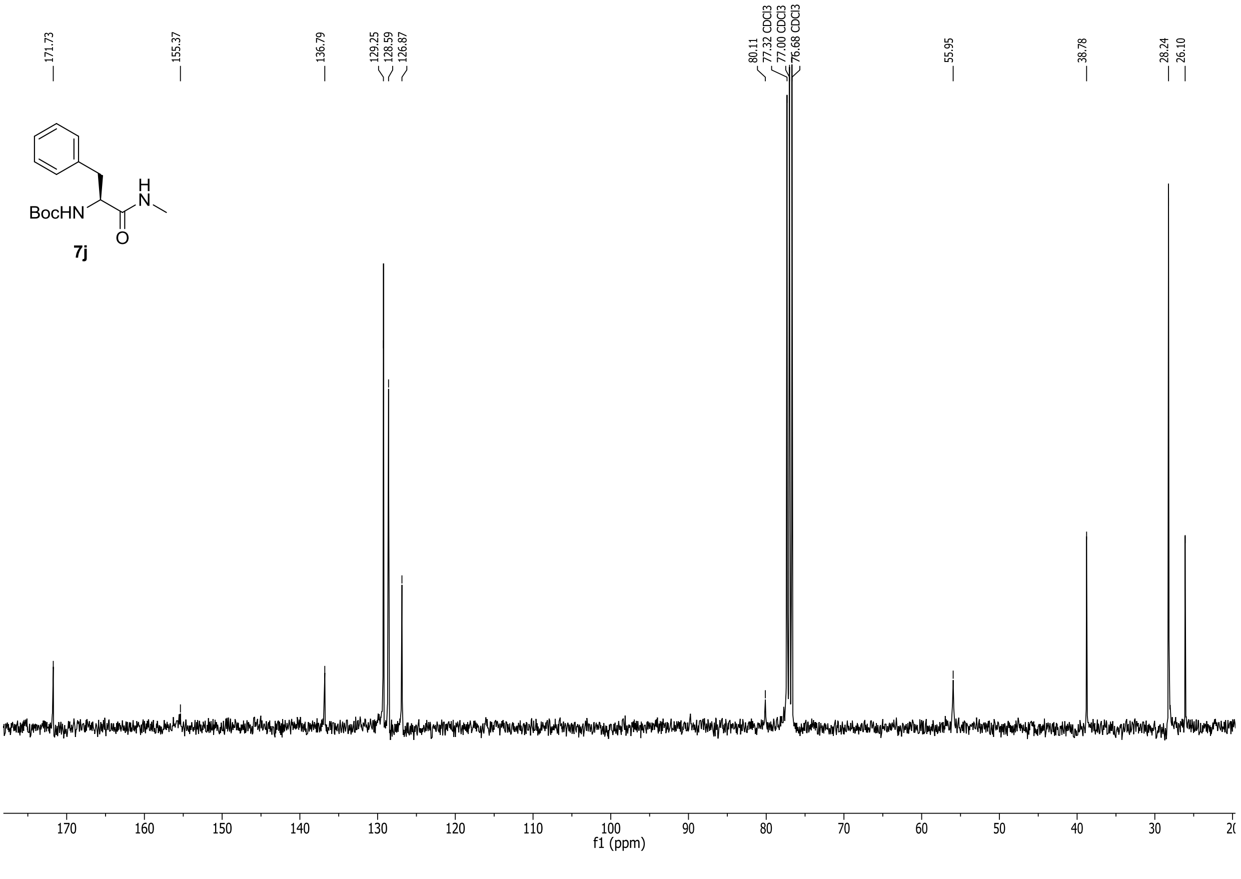
171.73
155.37
136.79
129.25
128.59
126.87
80.11
77.32 CDCl3
77.00 CDCl3
76.68 CDCl3
55.95
38.78
28.24
26.10
BocHN
H
N
O
7j
170
160
150
140
130
120
110
100
90
80
70
60
50
40
30
f1 (ppm)

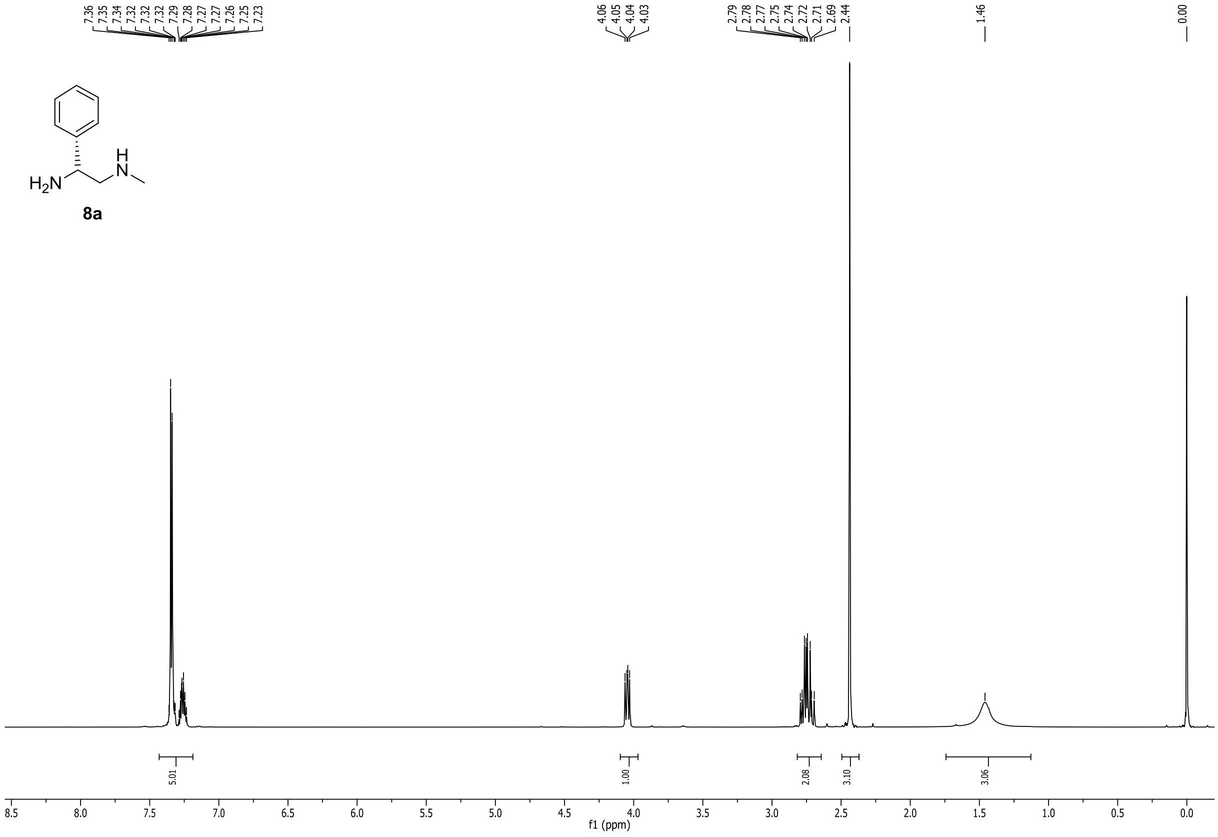
7.36 7.35 7.34 7.32 7.32 7.32 7.29 7.28 7.27 7.27 7.26 7.25 7.23
4.06 4.05 4.04 4.03
2.79 2.78 2.77 2.75 2.74 2.72 2.71 2.69 2.44
1.46
0.00
H
H2N
N
8a
5.01
1.00
2.08
3.10
3.06
8.5 8.0 7.5 7.0 6.5 6.0 5.5 5.0 4.5 4.0 3.5 3.0 2.5 2.0 1.5 1.0 0.5 0.0
f1 (ppm)

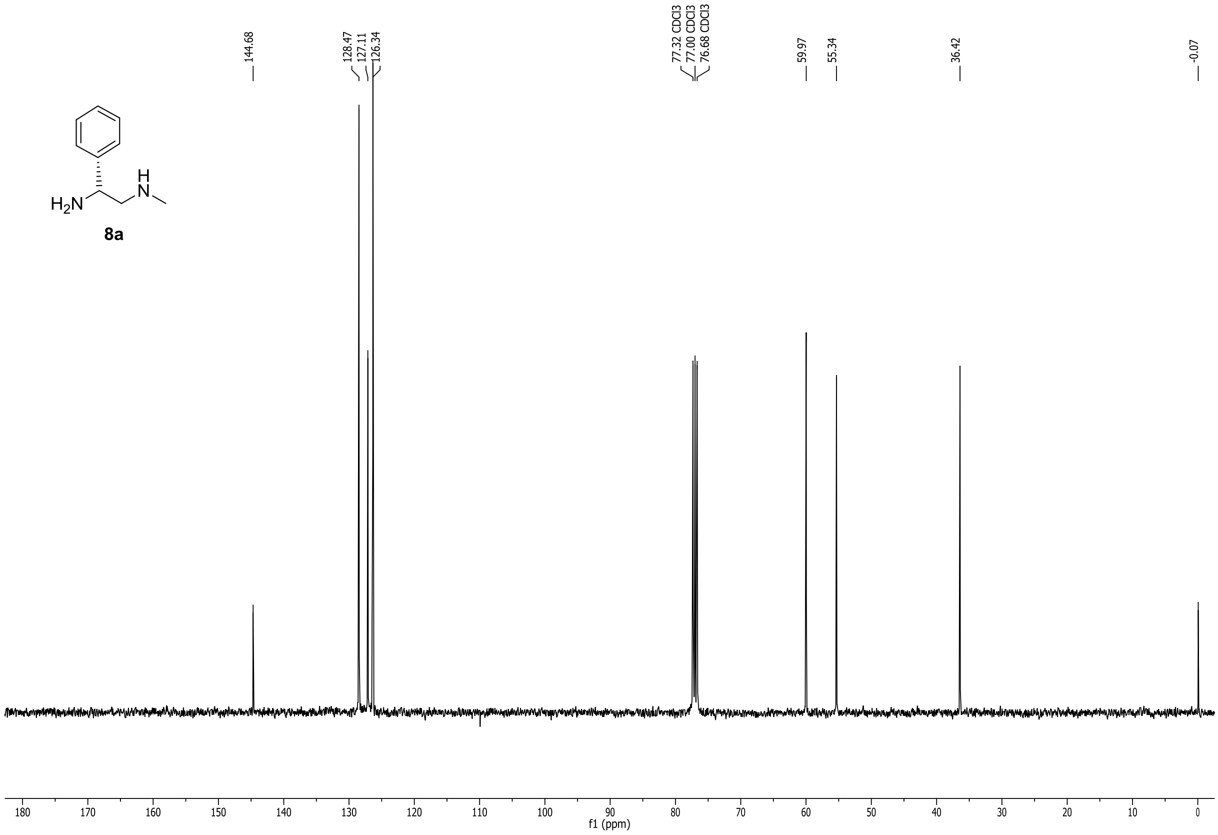
144.68
128.47 127.11 126.34
77.32 CDCl3 77.00 CDCl3 76.68 CDCl3
59.97
55.34
36.42
-0.07
H
H2N
N
8a
180 170 160 150 140 130 120 110 100 90 80 70 60 50 40 30 20 10 0
f1 (ppm)

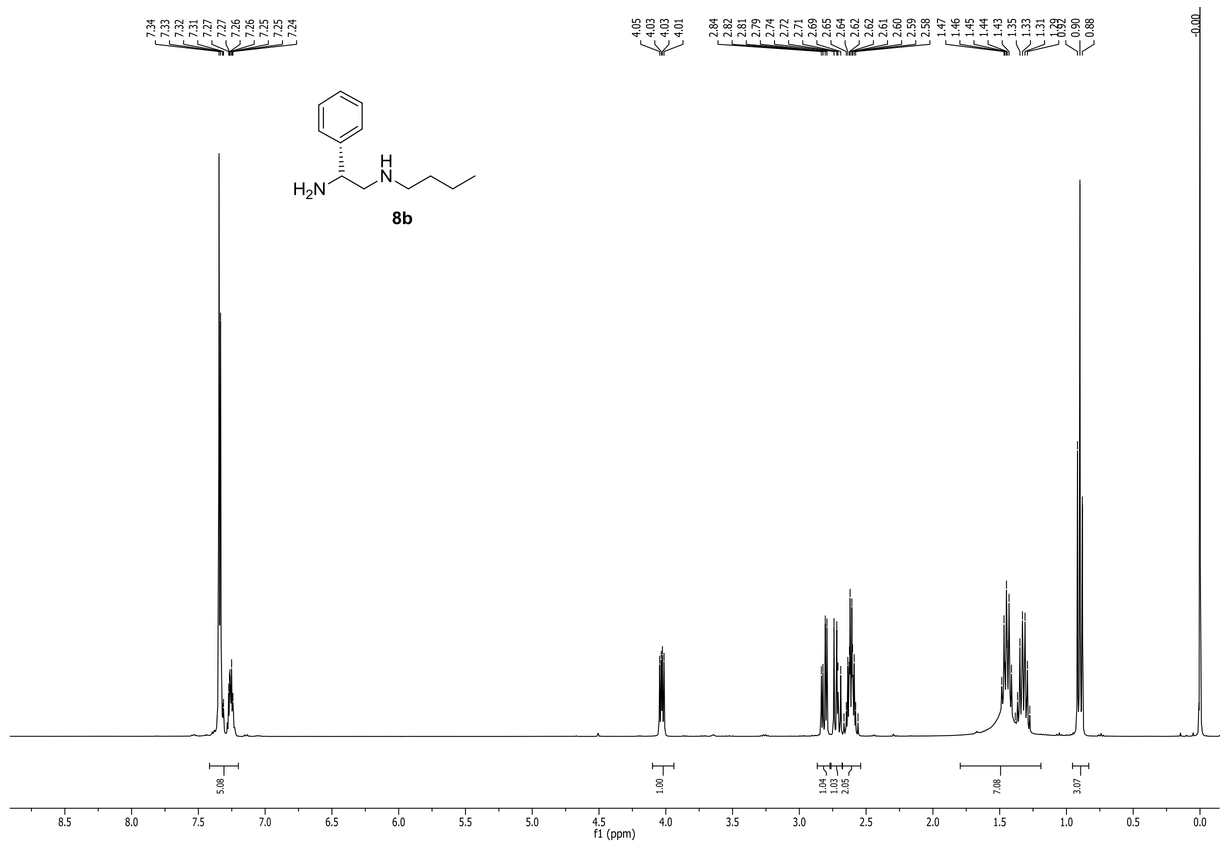

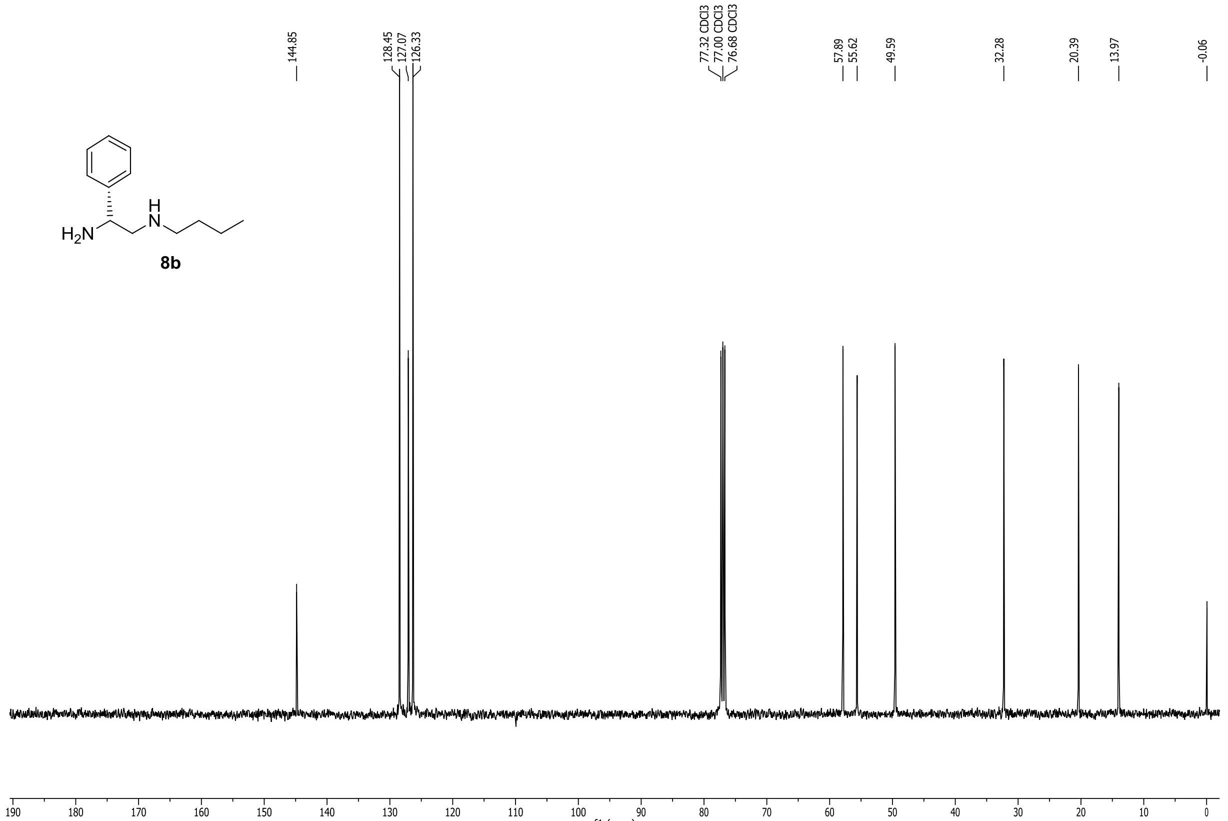

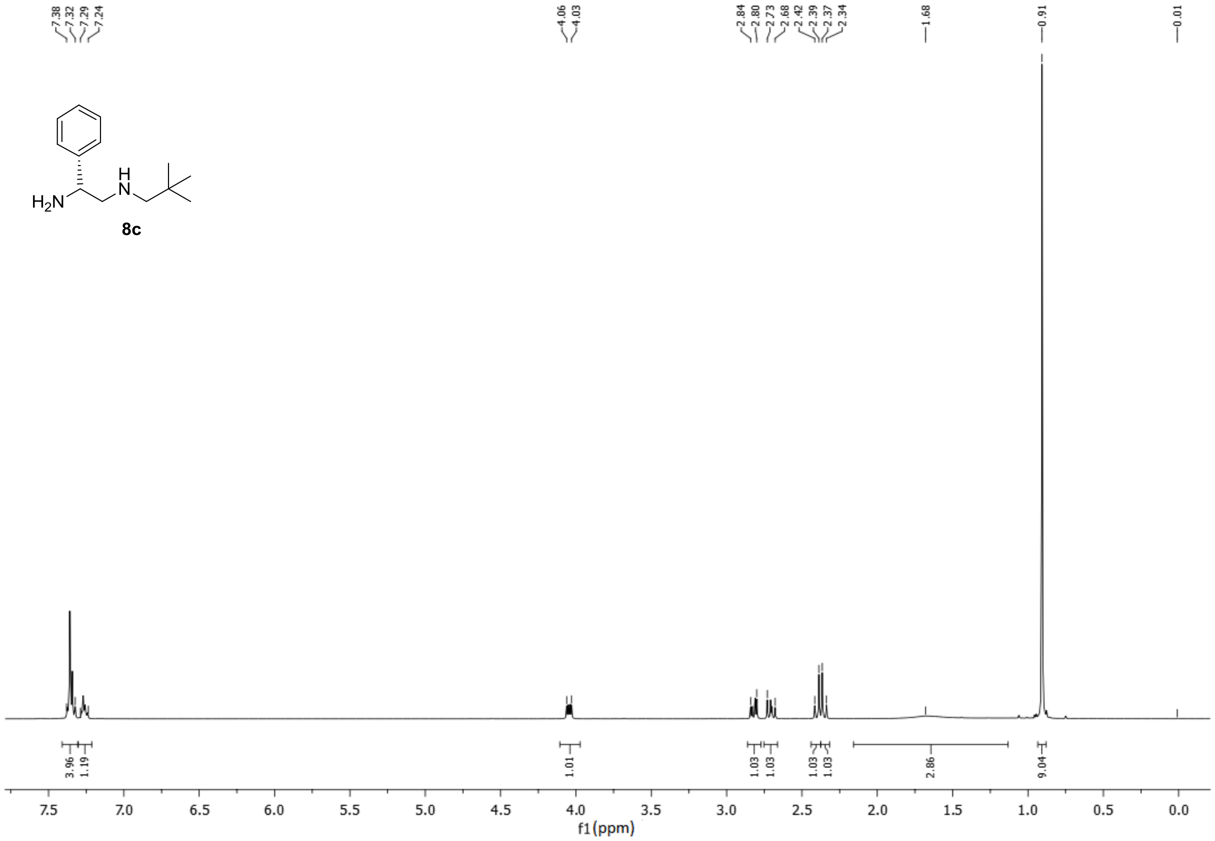
7.38
7.32
7.29
7.24
4.06
4.03
2.84
2.80
2.73
2.68
2.42
2.39
2.37
2.34
1.68
0.91
0.01
H_2N
H
N
8c
3.96
1.19
1.01
1.03
1.03
1.03
1.03
2.86
9.04
f1 (ppm)

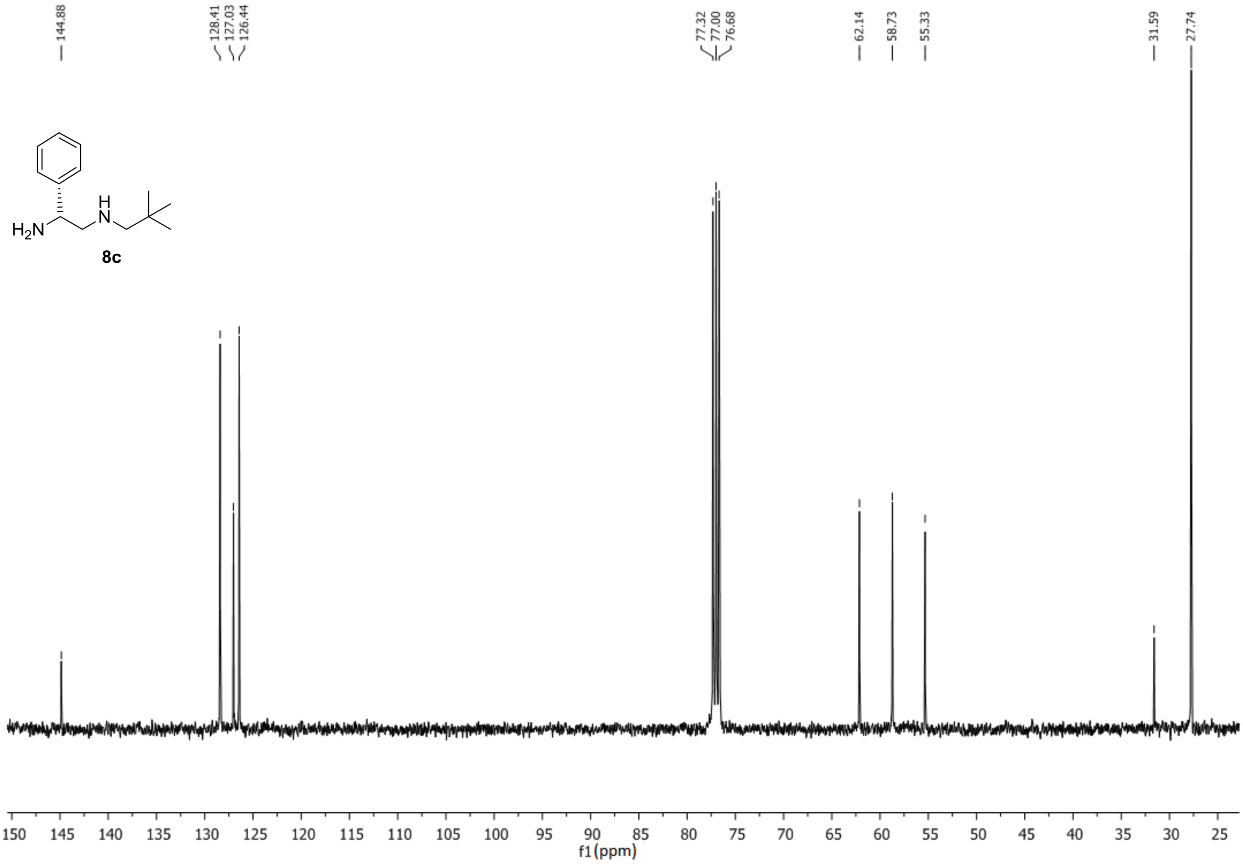
144.88
128.41
127.03
126.44
77.32
77.00
76.68
62.14
58.73
55.33
31.59
27.74
H_2N
H
N
8c
f1 (ppm)

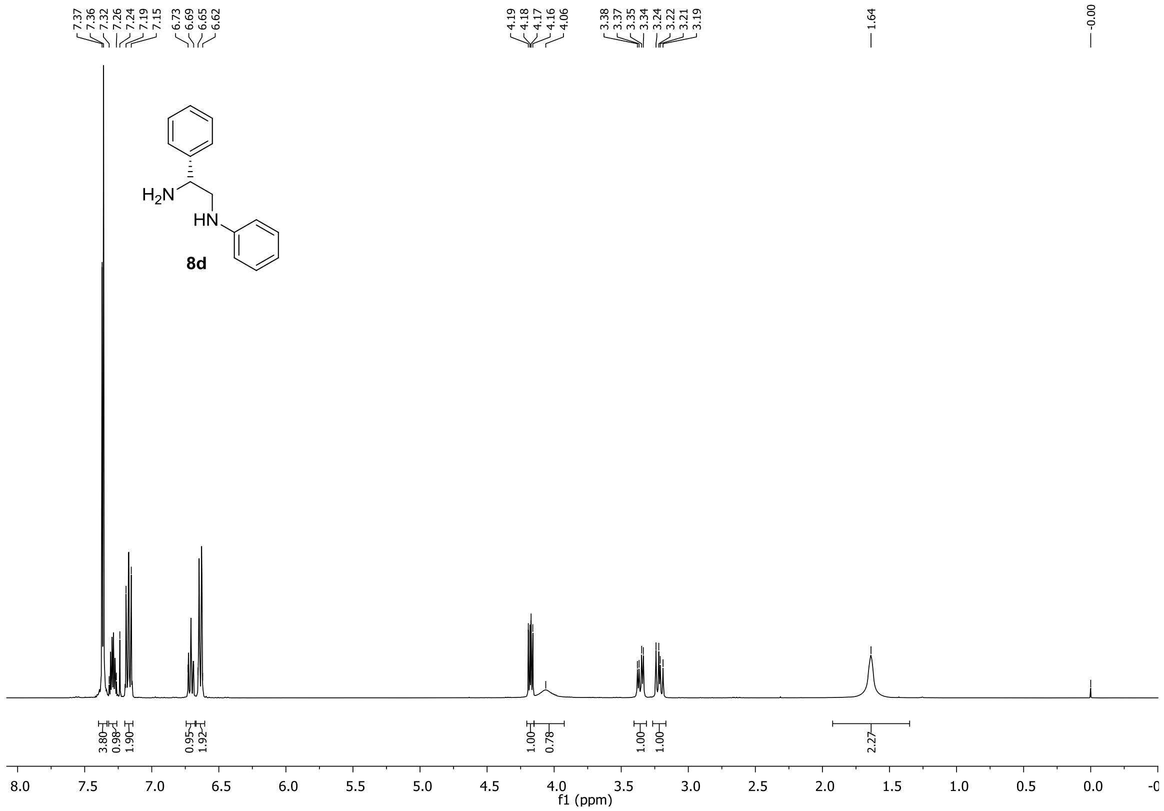
7.37
7.36
7.32
7.26
7.24
7.19
7.15
6.73
6.69
6.65
6.62
4.19
4.18
4.17
4.16
4.06
3.38
3.37
3.35
3.34
3.24
3.22
3.21
3.19
1.64
-0.00
H2N
HN
8d
3.80
0.98
1.90
0.95
1.92
1.00
0.78
1.00
1.00
2.27
f1 (ppm)

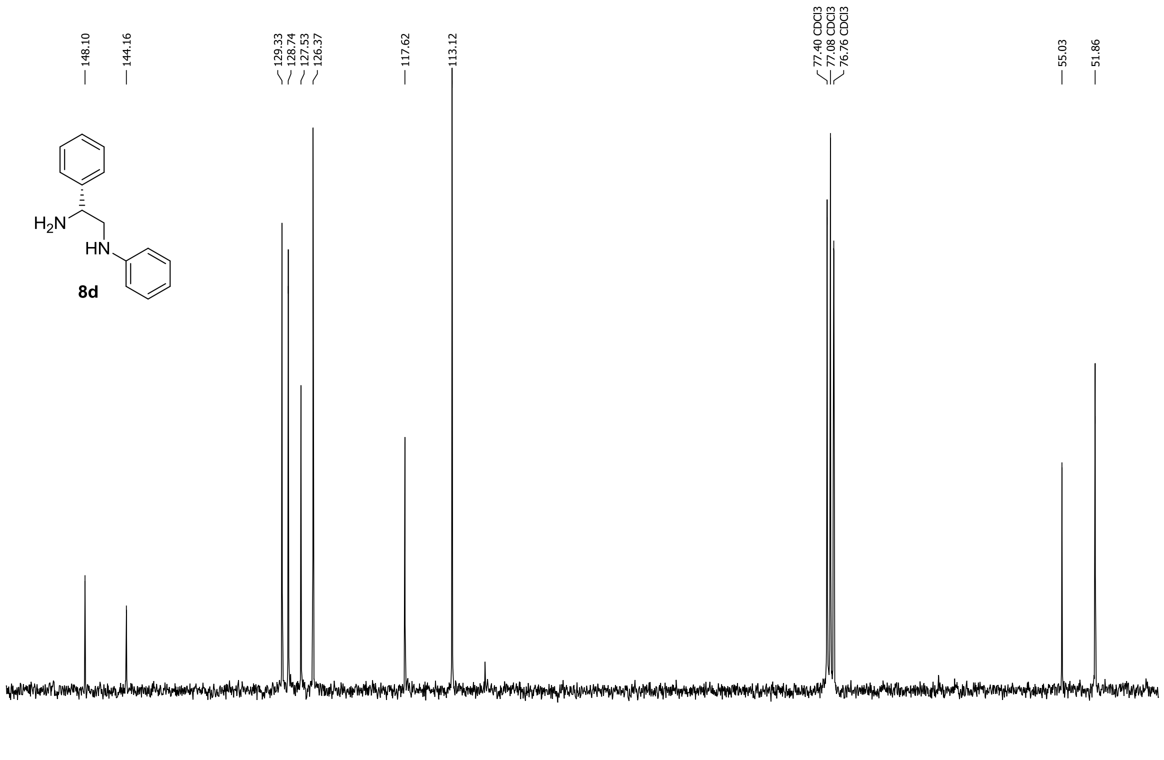
148.10
144.16
129.33
128.74
127.53
126.37
117.62
113.12
77.40 CDCl3
77.08 CDCl3
76.76 CDCl3
55.03
51.86
H2N
HN
8d
f1 (ppm)

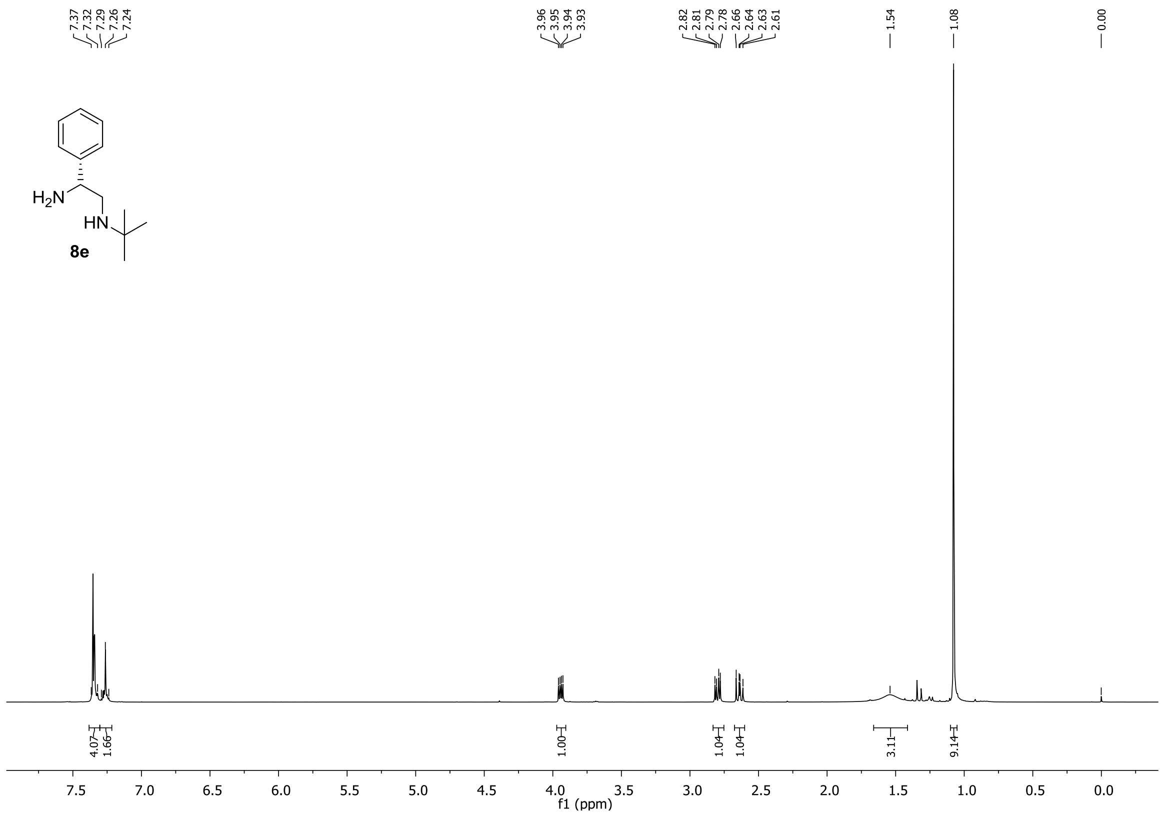

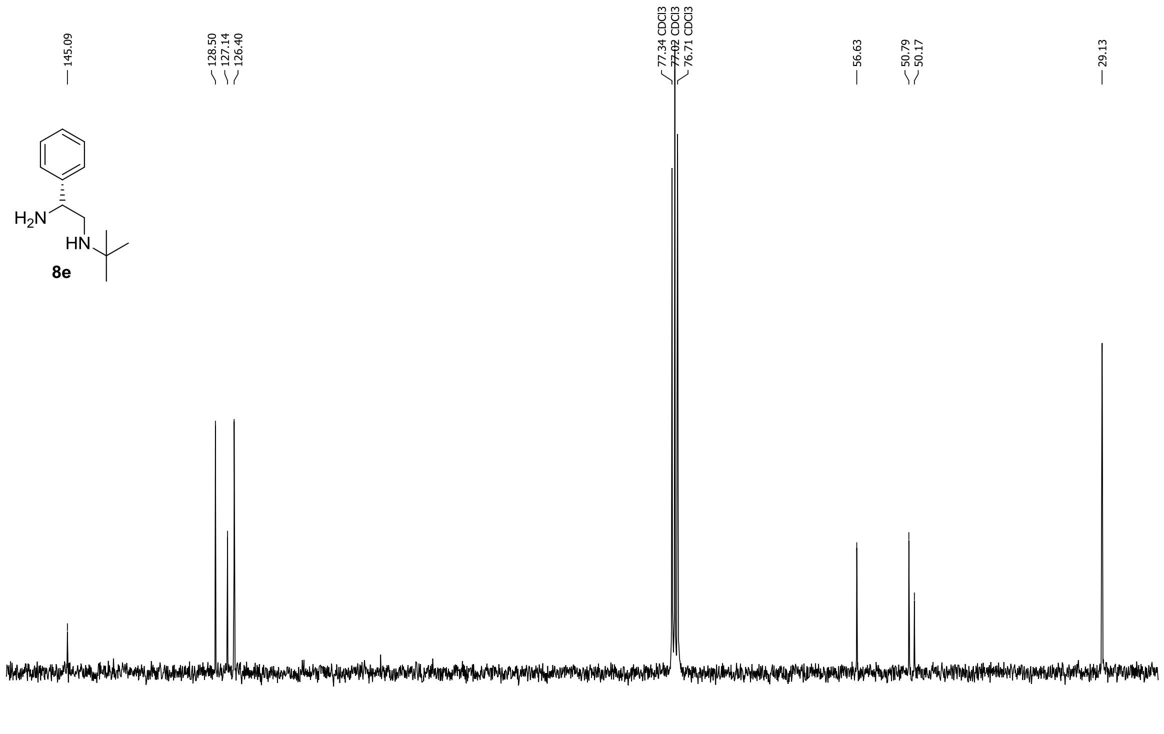

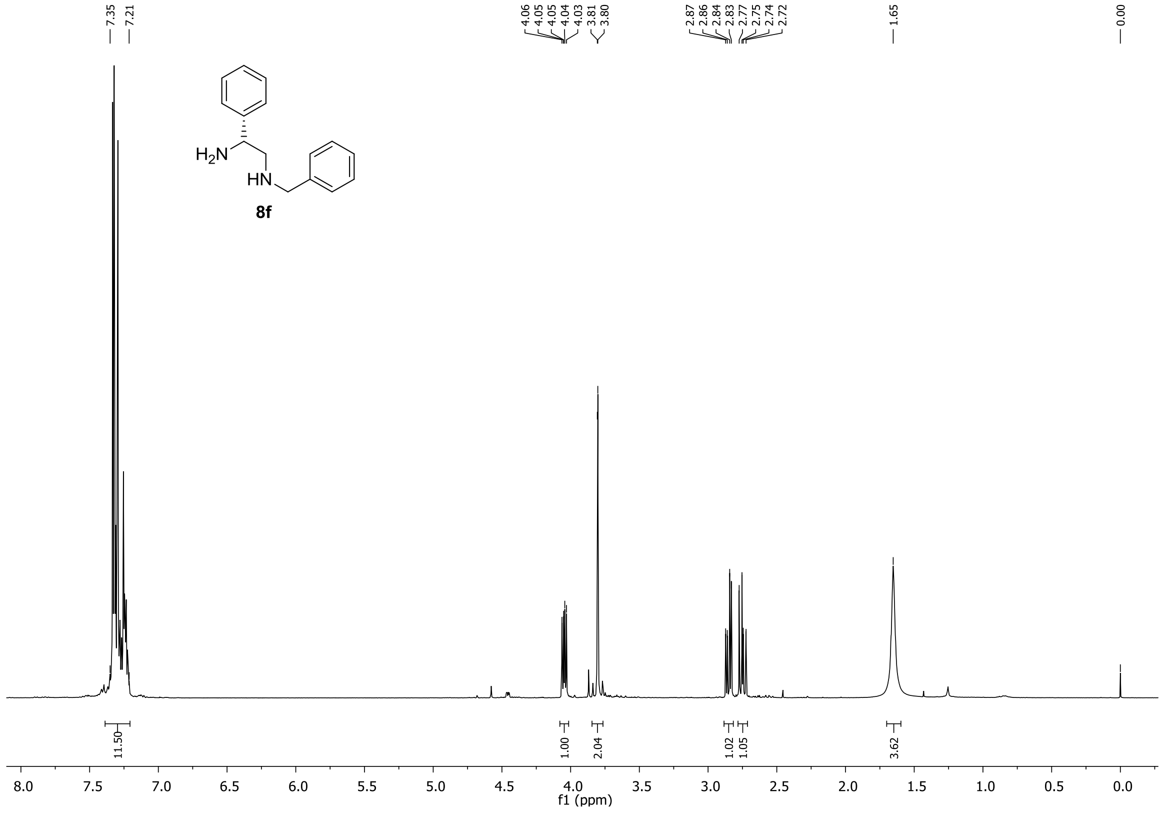
7.35
7.21
4.06
4.05
4.05
4.04
4.03
3.81
3.80
2.87
2.86
2.84
2.83
2.77
2.75
2.74
2.72
1.65
0.00
H2N
HN
8f
11.50
1.00
2.04
1.02
1.05
3.62
f1 (ppm)

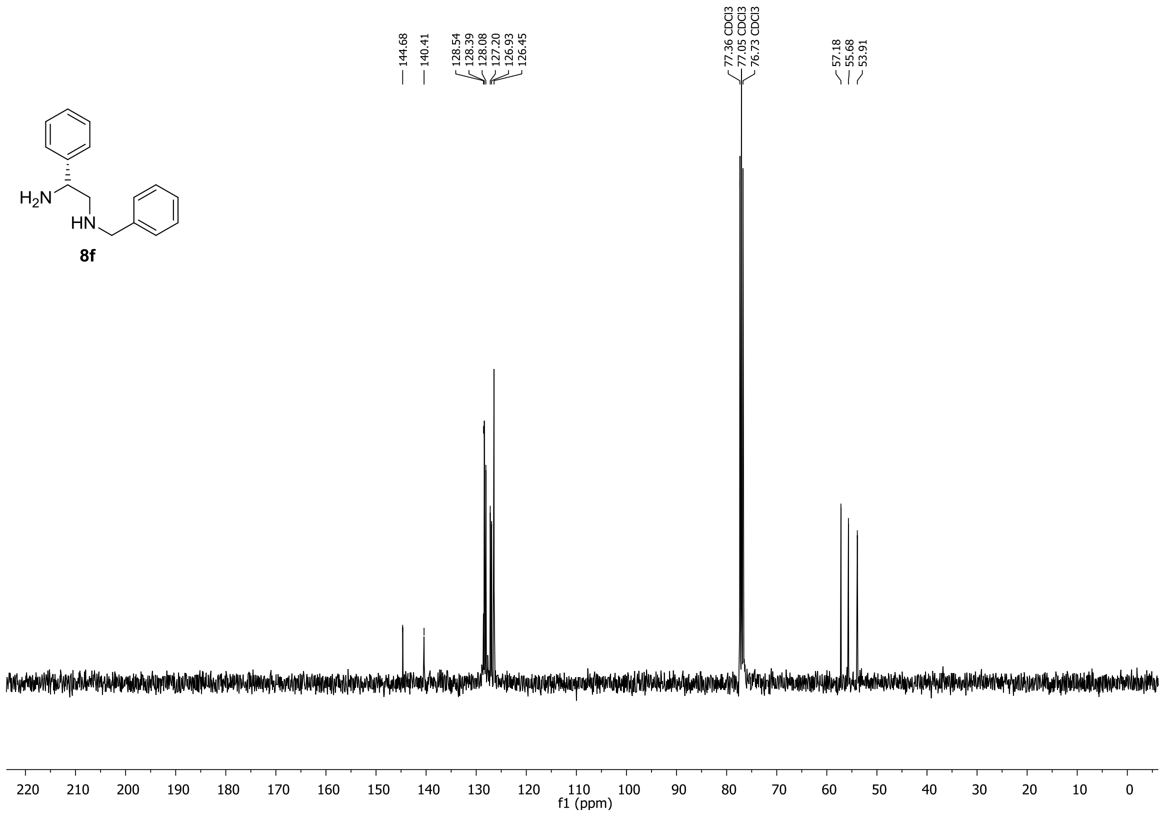
144.68
140.41
128.54
128.39
128.08
127.20
126.93
126.45
77.36 CDCl3
77.05 CDCl3
76.73 CDCl3
57.18
55.68
53.91
H2N
HN
8f
f1 (ppm)

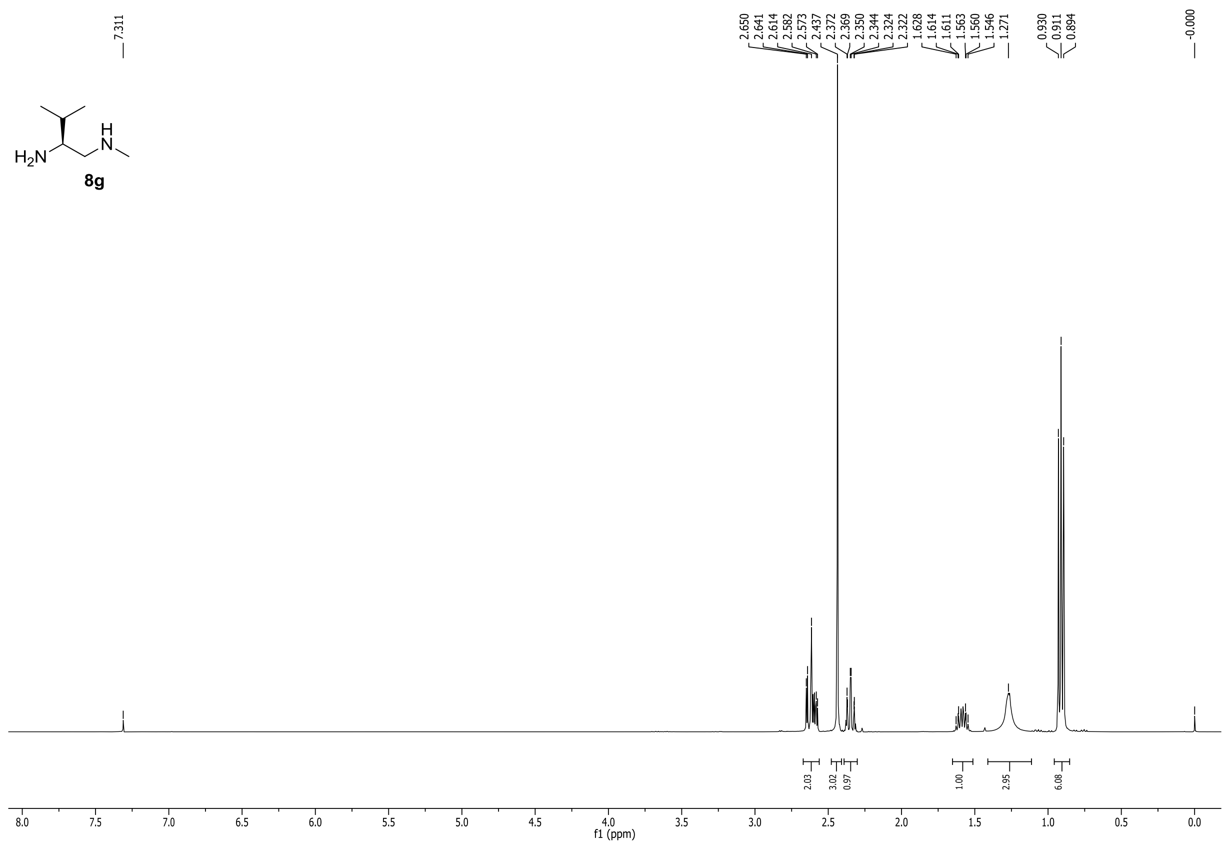
7.311
2.650
2.641
2.614
2.582
2.573
2.437
2.372
2.369
2.350
2.344
2.324
2.322
1.628
1.614
1.611
1.563
1.560
1.546
1.271
0.930
0.911
0.894
-0.000
H2N
H
N
8g
2.03
3.02
0.97
1.00
2.95
6.08
f1 (ppm)

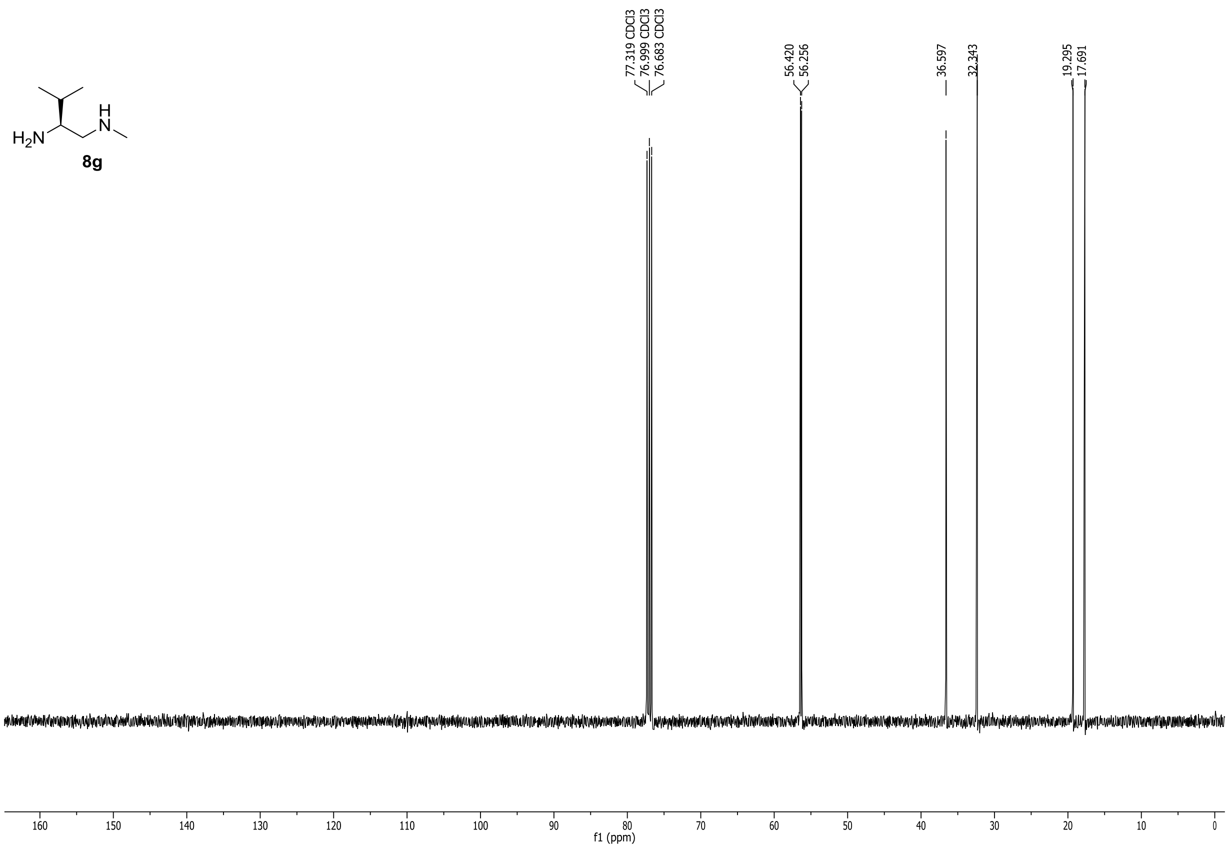
77.319 CDCl3
76.999 CDCl3
76.683 CDCl3
56.420
56.256
36.597
32.343
19.295
17.691
H2N
H
N
8g
f1 (ppm)

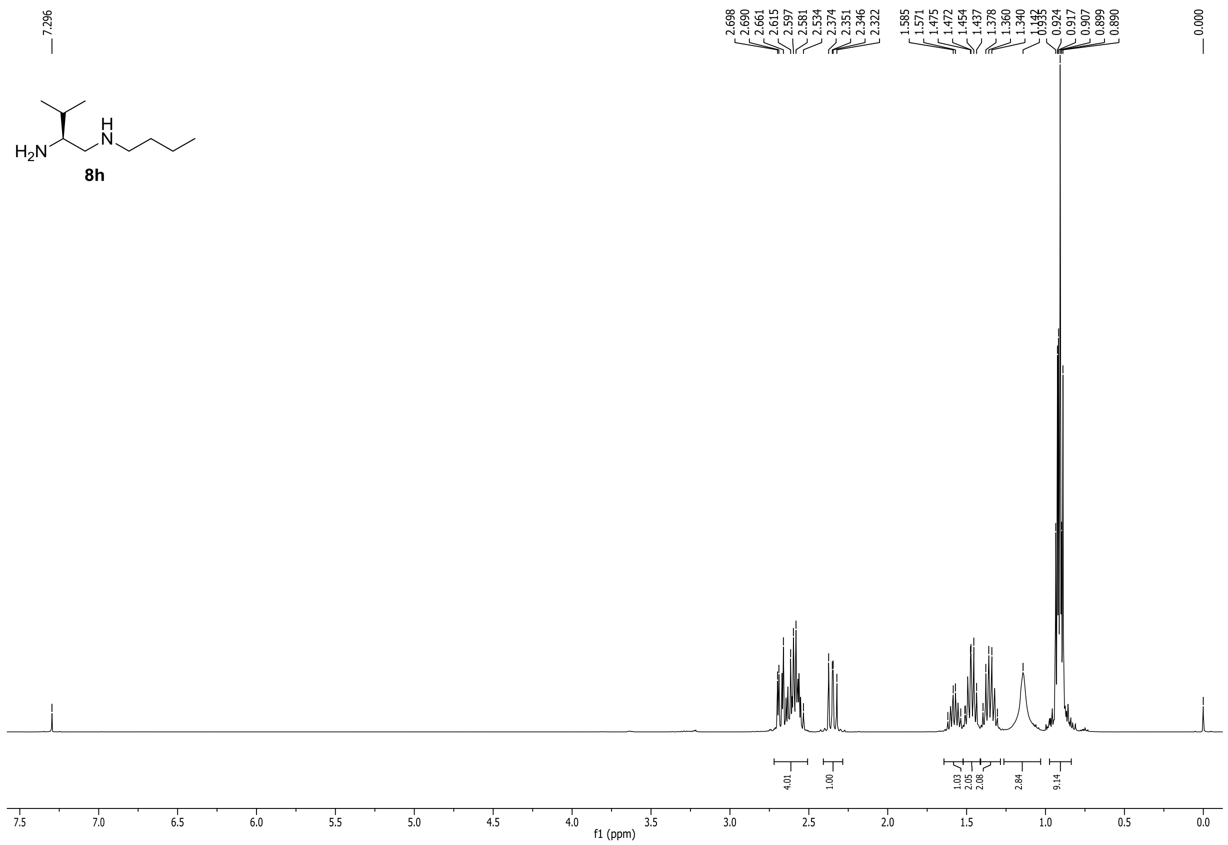
7.296
2.698
2.690
2.661
2.615
2.597
2.581
2.534
2.374
2.351
2.346
2.322
1.585
1.571
1.475
1.472
1.454
1.437
1.378
1.360
1.340
1.142
0.935
0.924
0.917
0.907
0.899
0.890
0.000
H
H_2N
N
8h
4.01
1.00
1.03
2.05
2.08
2.84
9.14
f1 (ppm)

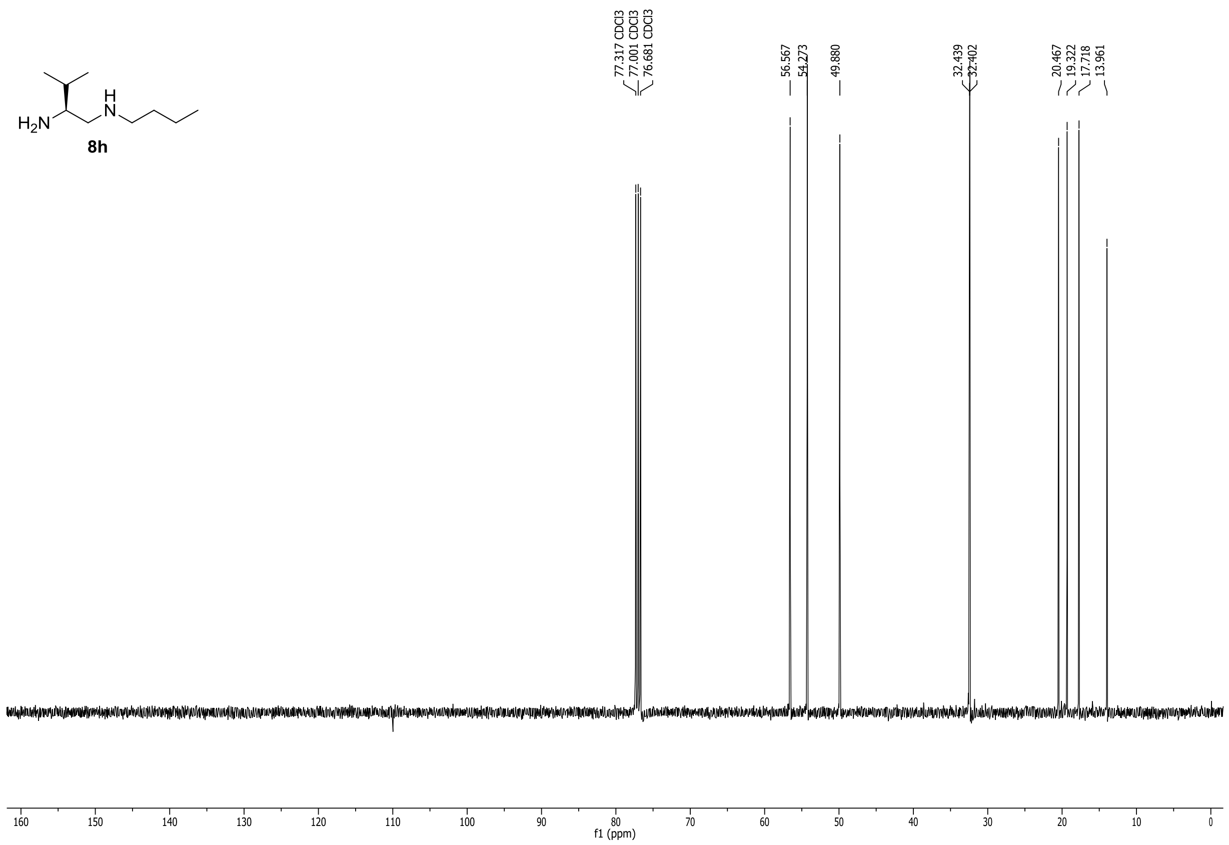
77.317 CDCl3
77.001 CDCl3
76.681 CDCl3
56.567
54.273
49.880
32.439
32.402
20.467
19.322
17.718
13.961
H
H_2N
N
8h
f1 (ppm)

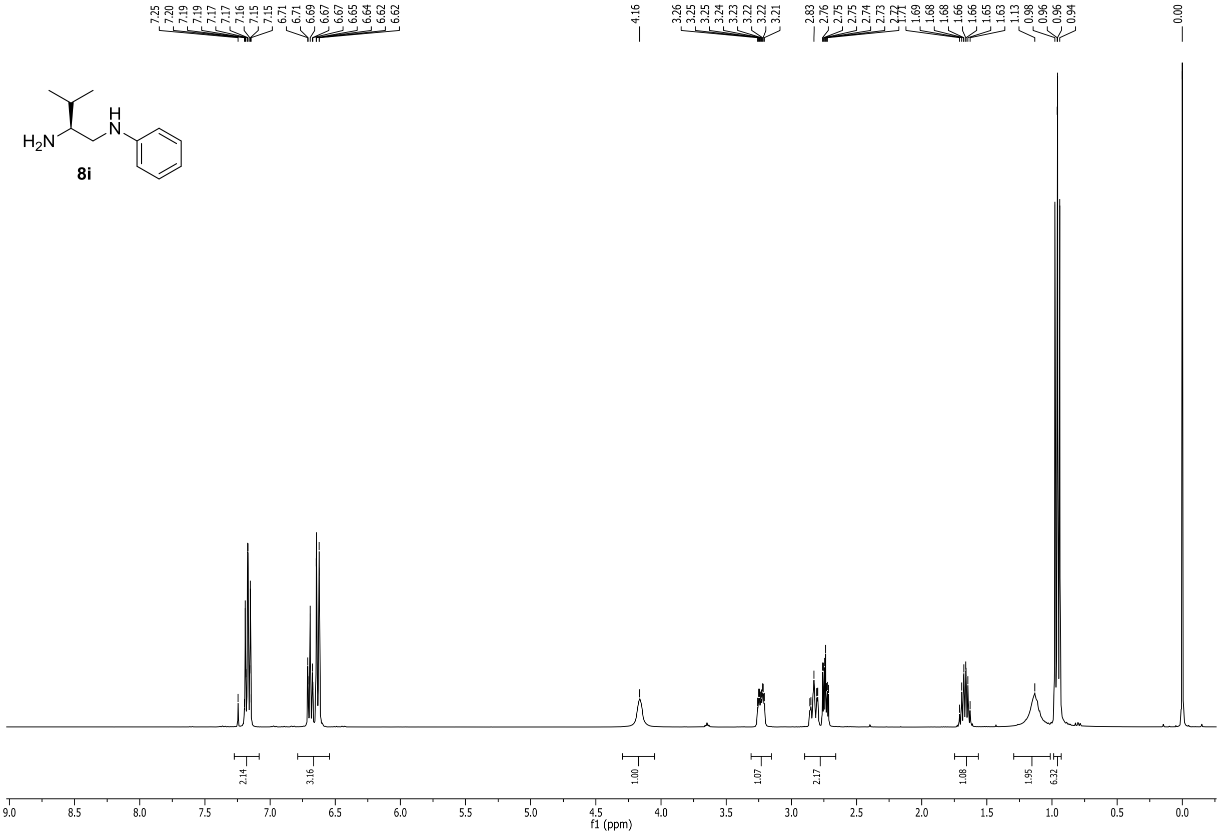
H₂N
H
N
8i
7.25 7.20 7.19 7.19 7.17 7.17 7.16 7.15 7.15 6.71 6.71 6.69 6.67 6.67 6.65 6.64 6.62 6.62
4.16
3.26 3.25 3.25 3.24 3.23 3.23 3.22 3.22 3.21
2.83 2.76 2.75 2.75 2.74 2.73 2.72 1.71 1.69 1.68 1.68 1.66 1.66 1.65 1.63 1.13 0.98 0.96 0.96 0.94
0.00
2.14 3.16 1.00 1.07 2.17 1.08 1.95 6.32
f1 (ppm)

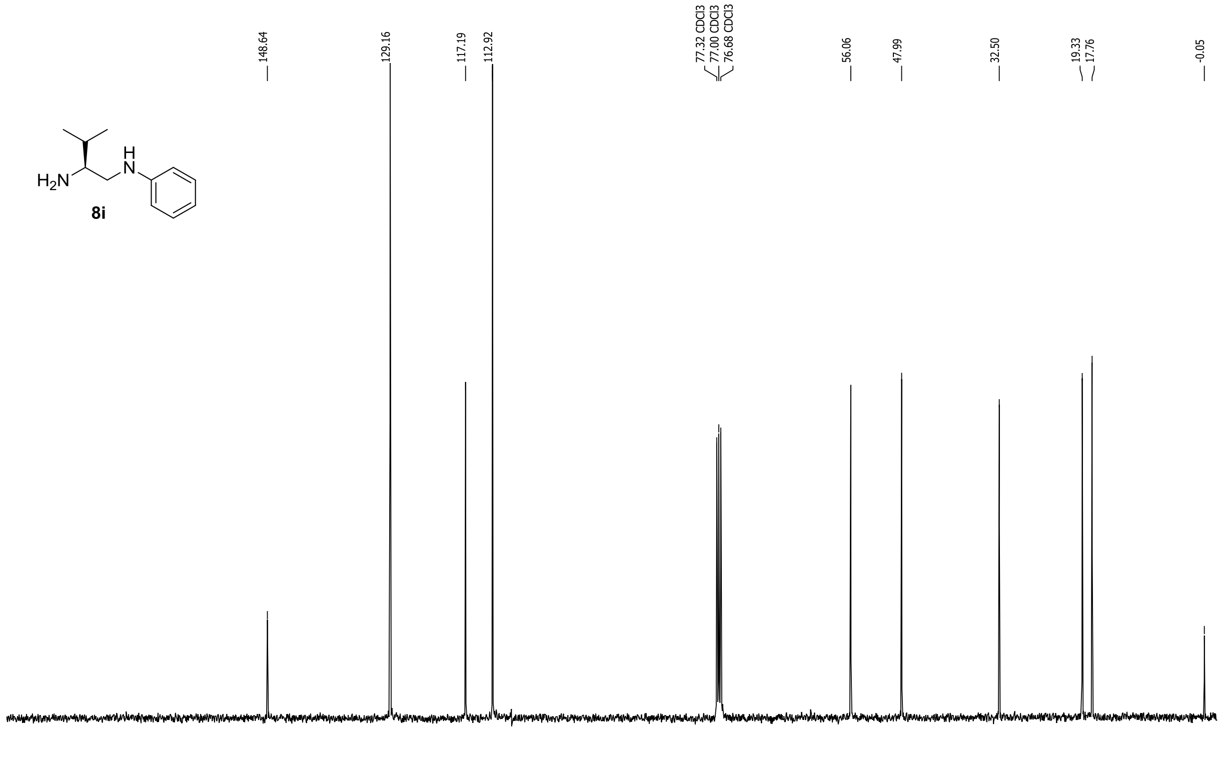
H₂N
H
N
8i
148.64
129.16
117.19
112.92
77.32 CDCl3
77.00 CDCl3
76.68 CDCl3
56.06
47.99
32.50
19.33
17.76
-0.05
f1 (ppm)

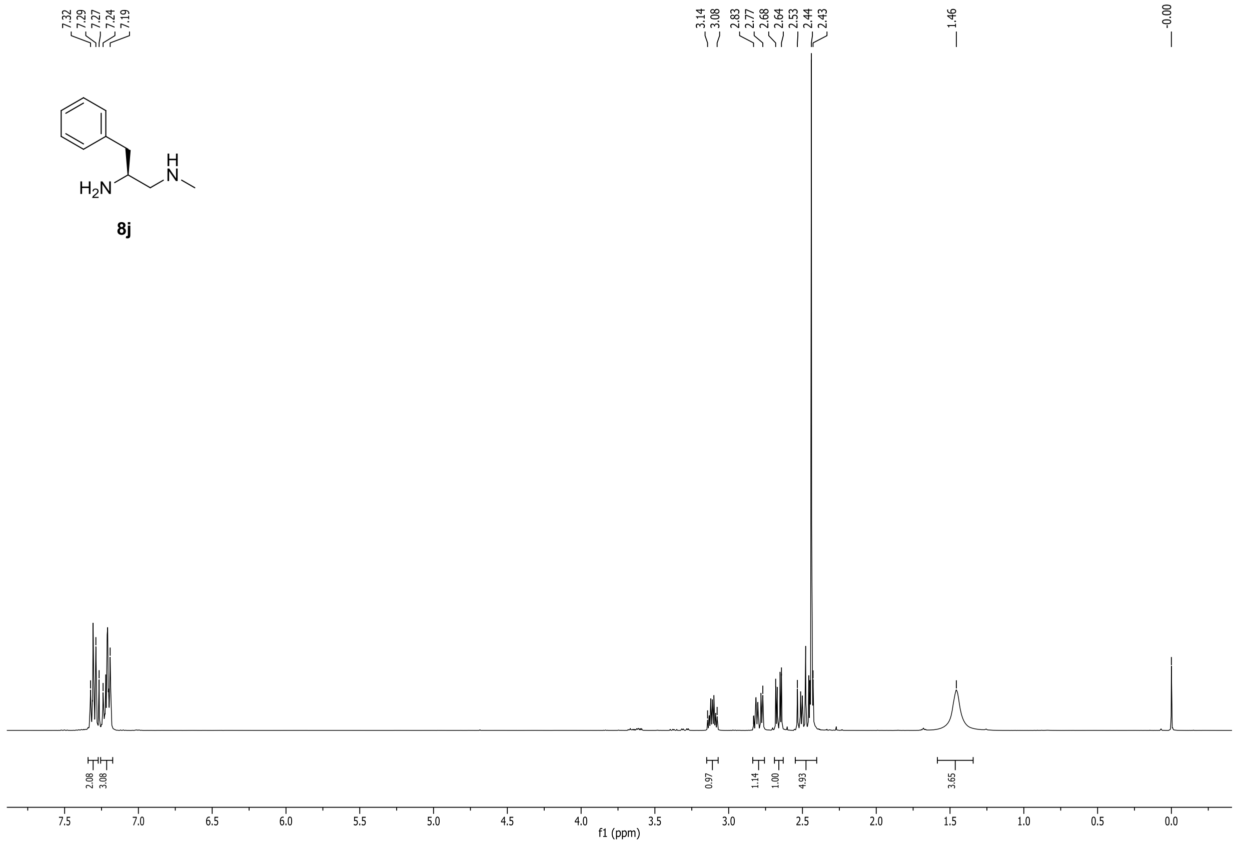
7.32
7.29
7.27
7.24
7.19
3.14
3.08
2.83
2.77
2.68
2.64
2.53
2.44
2.43
1.46
-0.00
H
H_2N
N
8j
2.08
3.08
0.97
1.14
1.00
4.93
3.65
7.5 7.0 6.5 6.0 5.5 5.0 4.5 4.0 3.5 3.0 2.5 2.0 1.5 1.0 0.5 0.0
f1 (ppm)

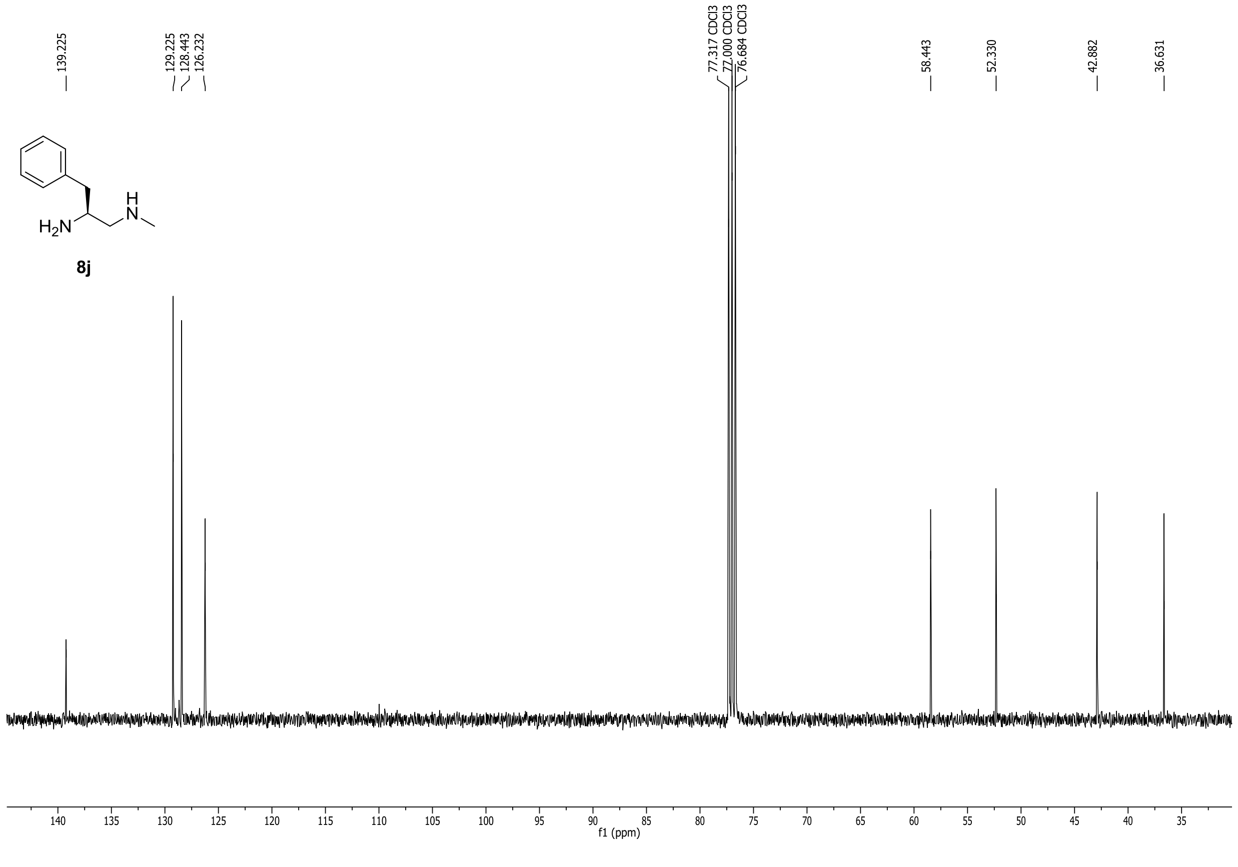
139.225
129.225
128.443
126.232
77.317 CDCl3
77.000 CDCl3
76.684 CDCl3
58.443
52.330
42.882
36.631
H
H_2N
N
8j
140 135 130 125 120 115 110 105 100 95 90 85 80 75 70 65 60 55 50 45 40 35
f1 (ppm)

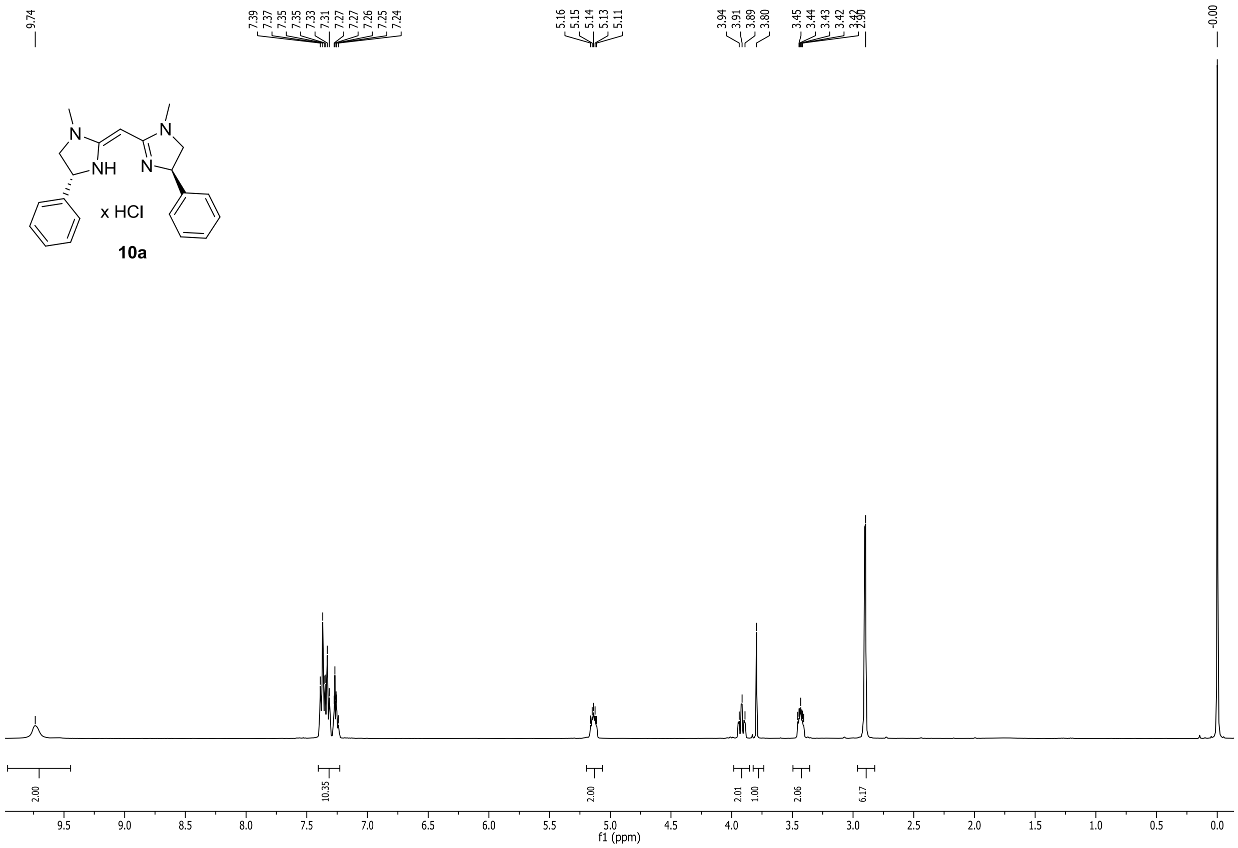

x HCl
10a
f1 (ppm)

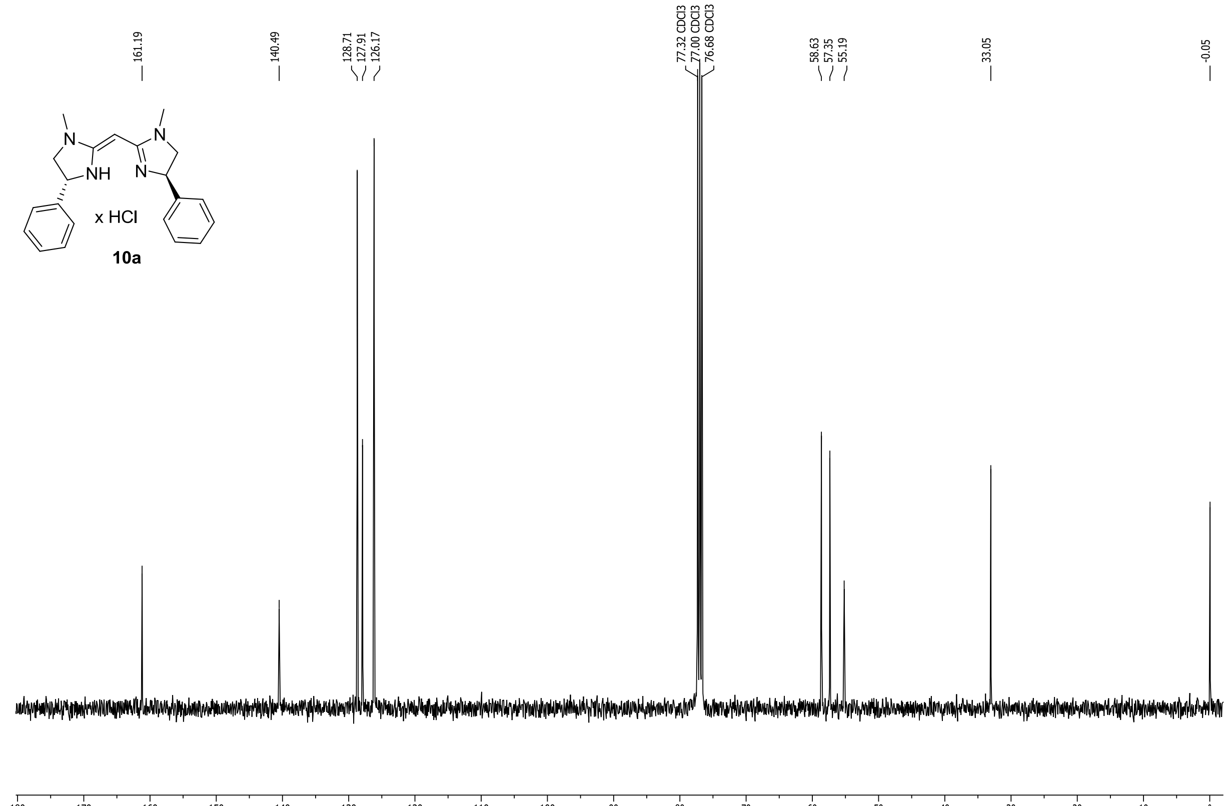

x HCl
10a
f1 (ppm)

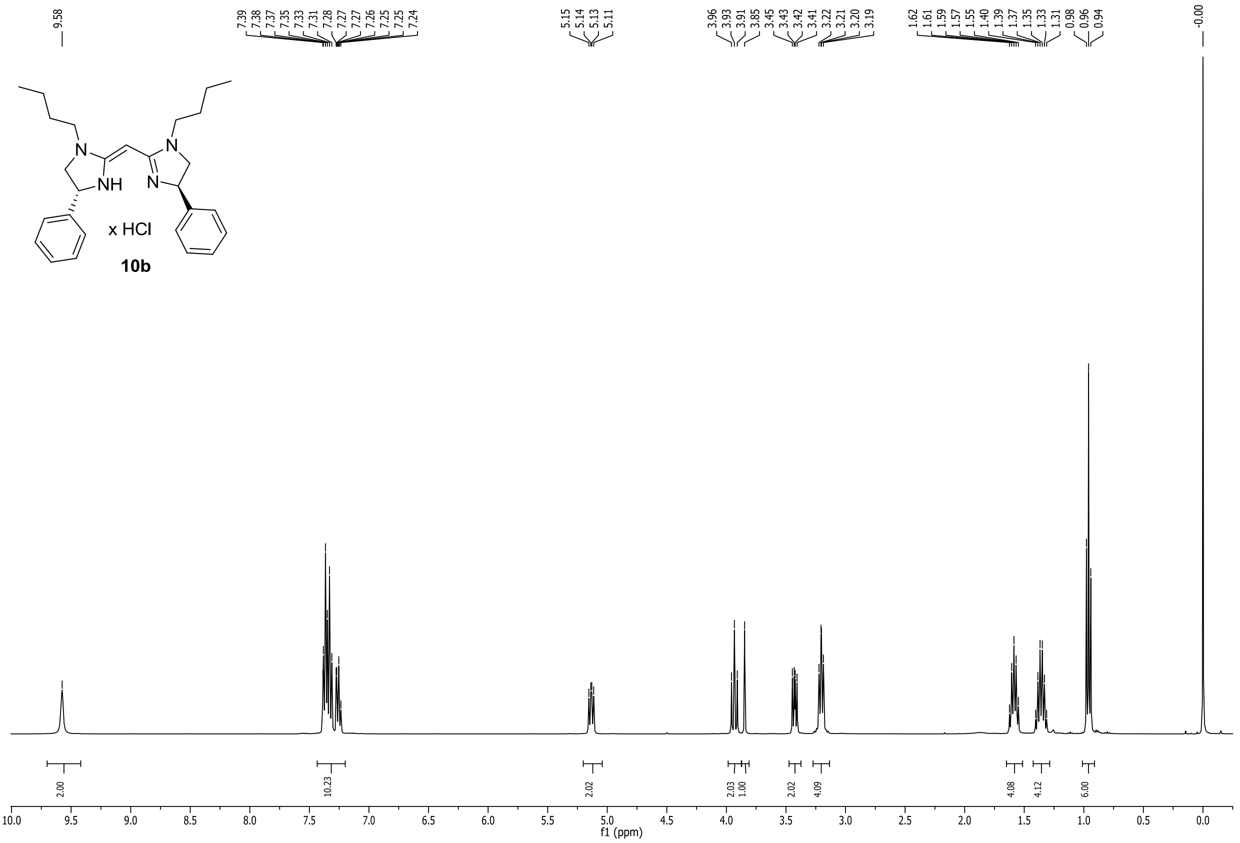

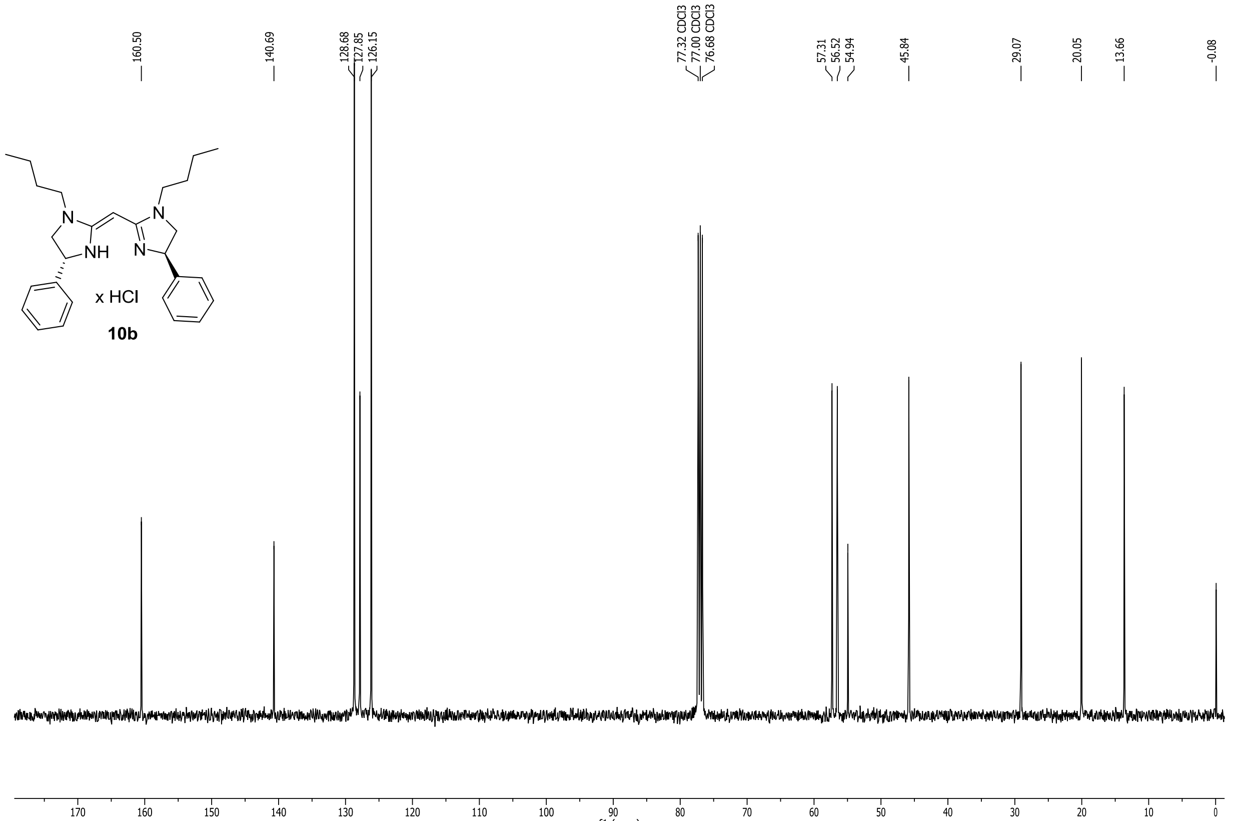

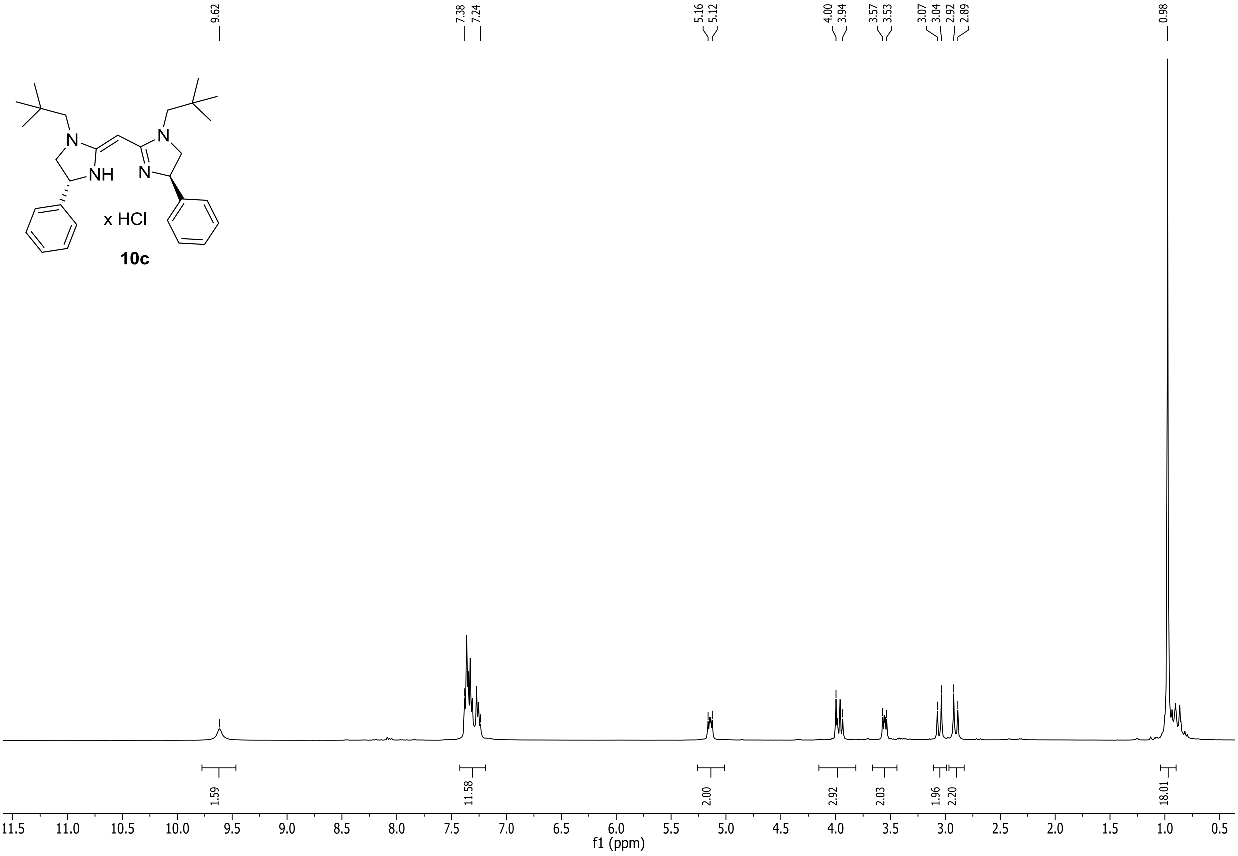
9.62
7.38
7.24
5.16
5.12
4.00
3.94
3.57
3.53
3.07
3.04
2.92
2.89
0.98
x HCl
10c
1.59
11.58
2.00
2.92
2.03
1.96
2.20
18.01
f1 (ppm)

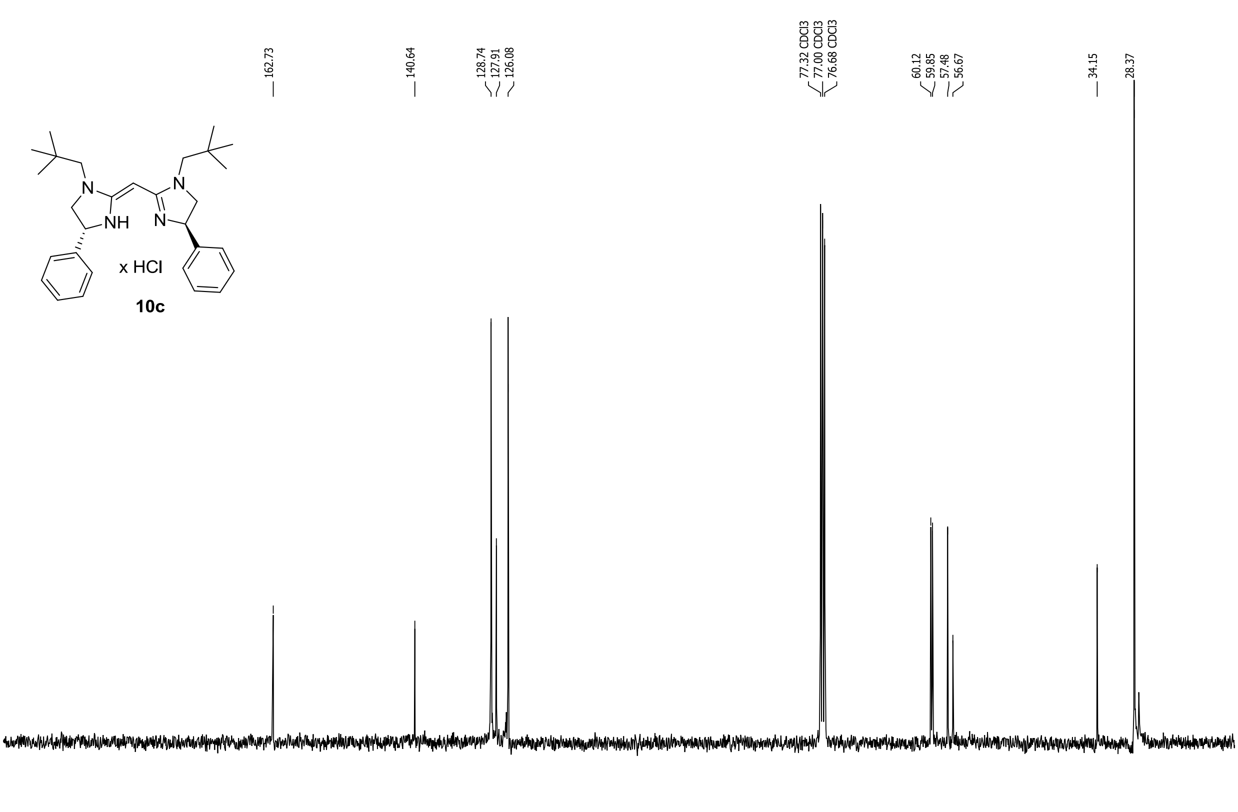
162.73
140.64
128.74
127.91
126.08
77.32 CDCl3
77.00 CDCl3
76.68 CDCl3
60.12
59.85
57.48
56.67
34.15
28.37
x HCl
10c
f1 (ppm)

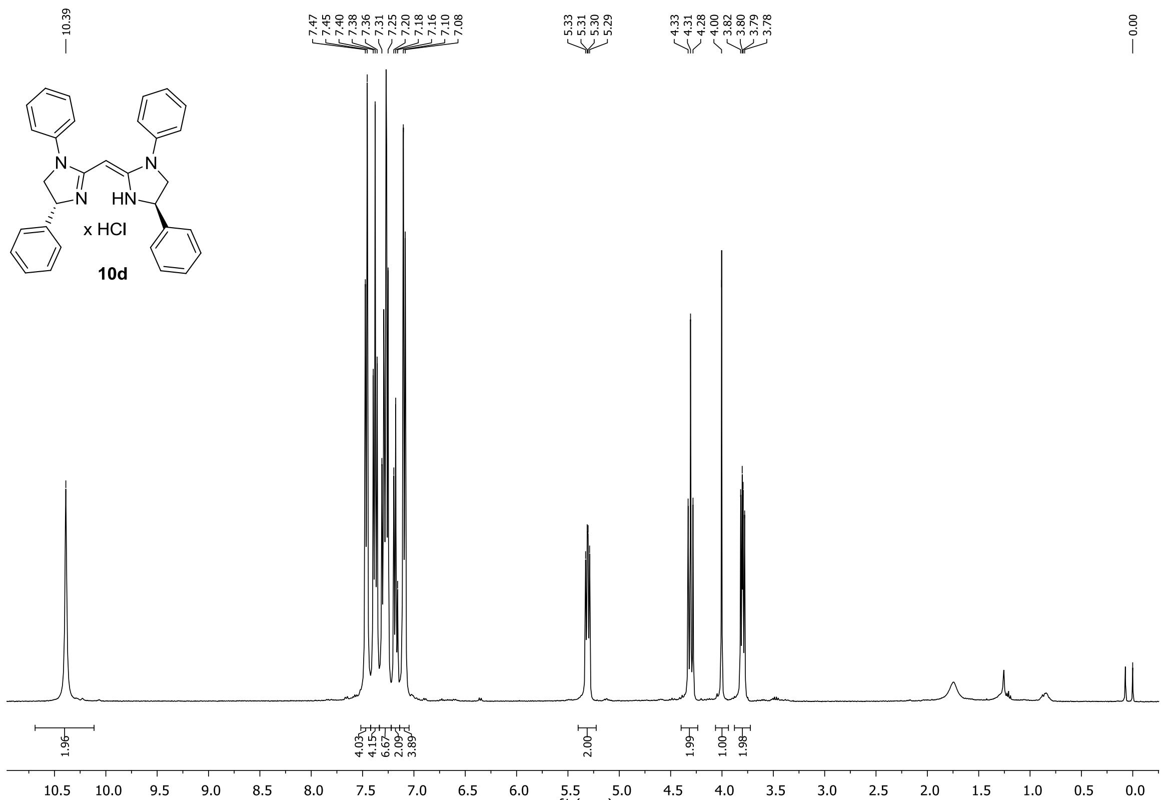
10.39
7.47 7.45 7.40 7.38 7.36 7.31 7.25 7.20 7.18 7.16 7.10 7.08
5.33 5.31 5.30 5.29
4.33 4.31 4.28 4.00 3.82 3.80 3.79 3.78
0.00
x HCl
10d
HN
N
1.96
4.03 4.15 6.67 2.09 3.89
2.00
1.99
1.00
1.98
f1 (ppm)

c

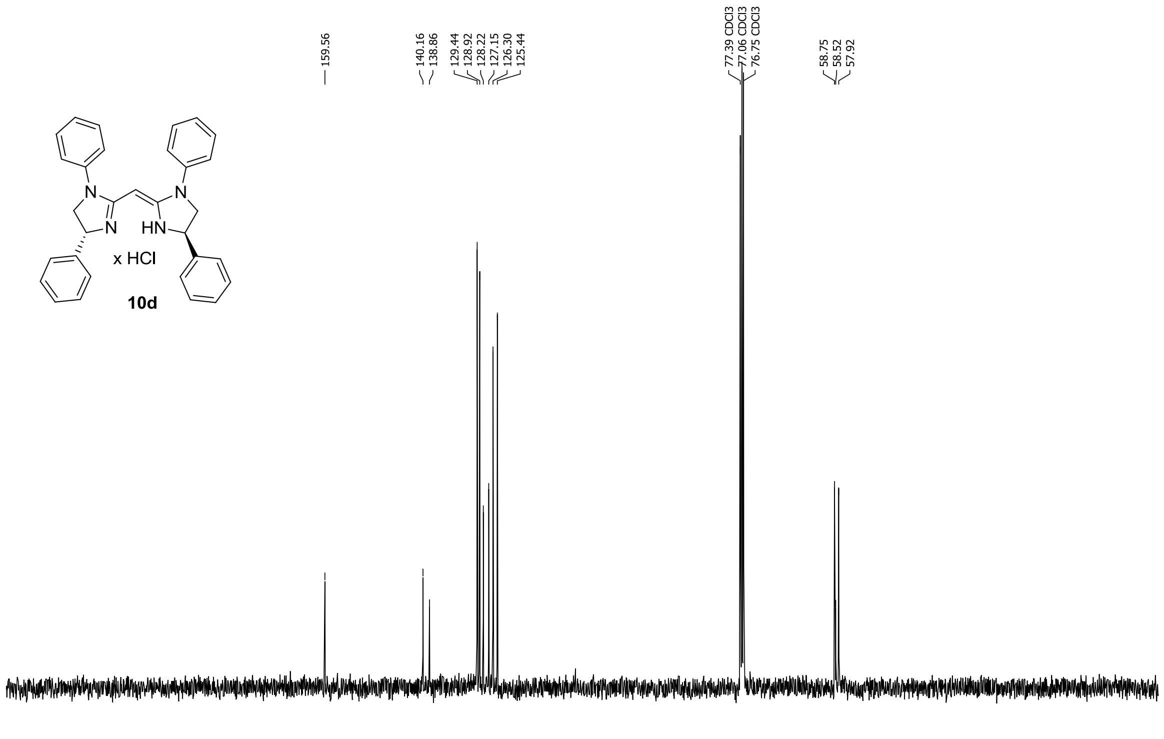
159.56
140.16 138.86
129.44 128.92 128.22 127.15 126.30 125.44
77.39 CDCl3 77.06 CDCl3 76.75 CDCl3
58.75 58.52 57.92
x HCl
10d
HN
N
f1 (ppm)

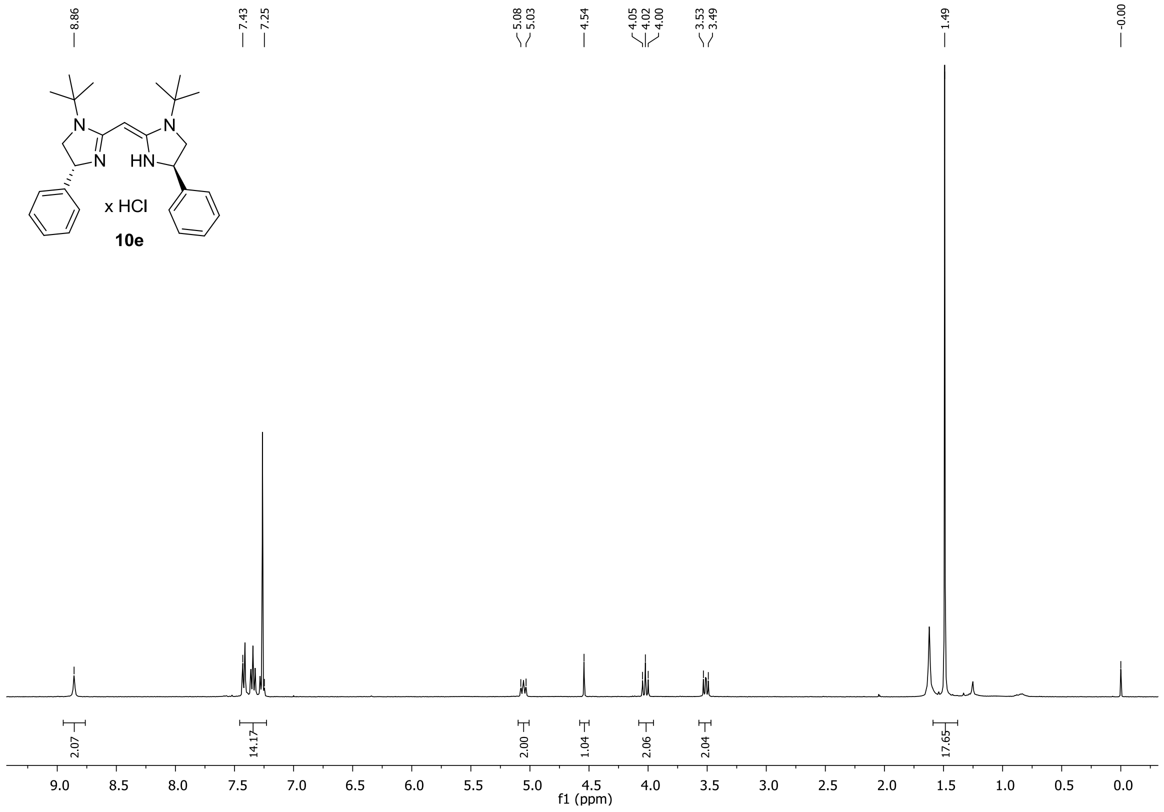
8.86
7.43
7.25
5.08
5.03
4.54
4.05
4.02
4.00
3.53
3.49
1.49
-0.00
x HCl
10e
2.07
14.17
2.00
1.04
2.06
2.04
17.65
f1 (ppm)

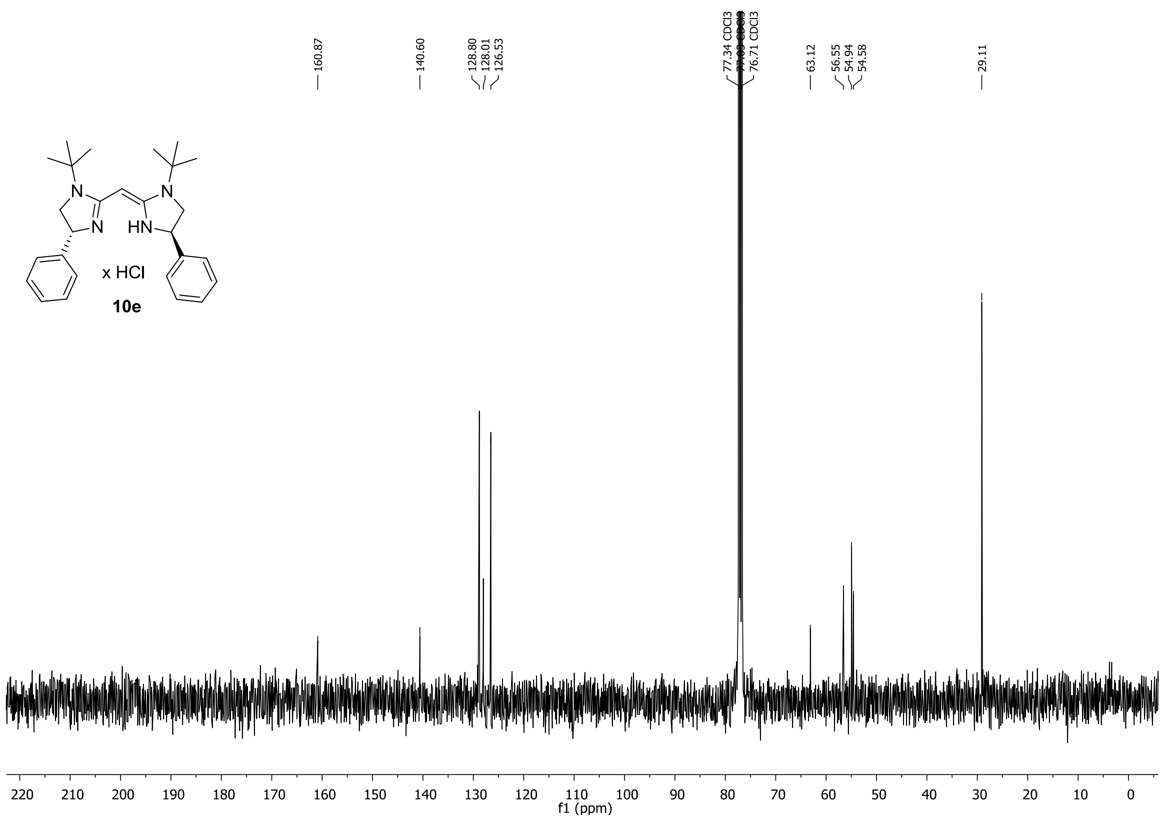
160.87
140.60
128.80
128.01
126.53
77.34 CDCl3
76.71 CDCl3
63.12
56.55
54.94
54.58
29.11
x HCl
10e
f1 (ppm)

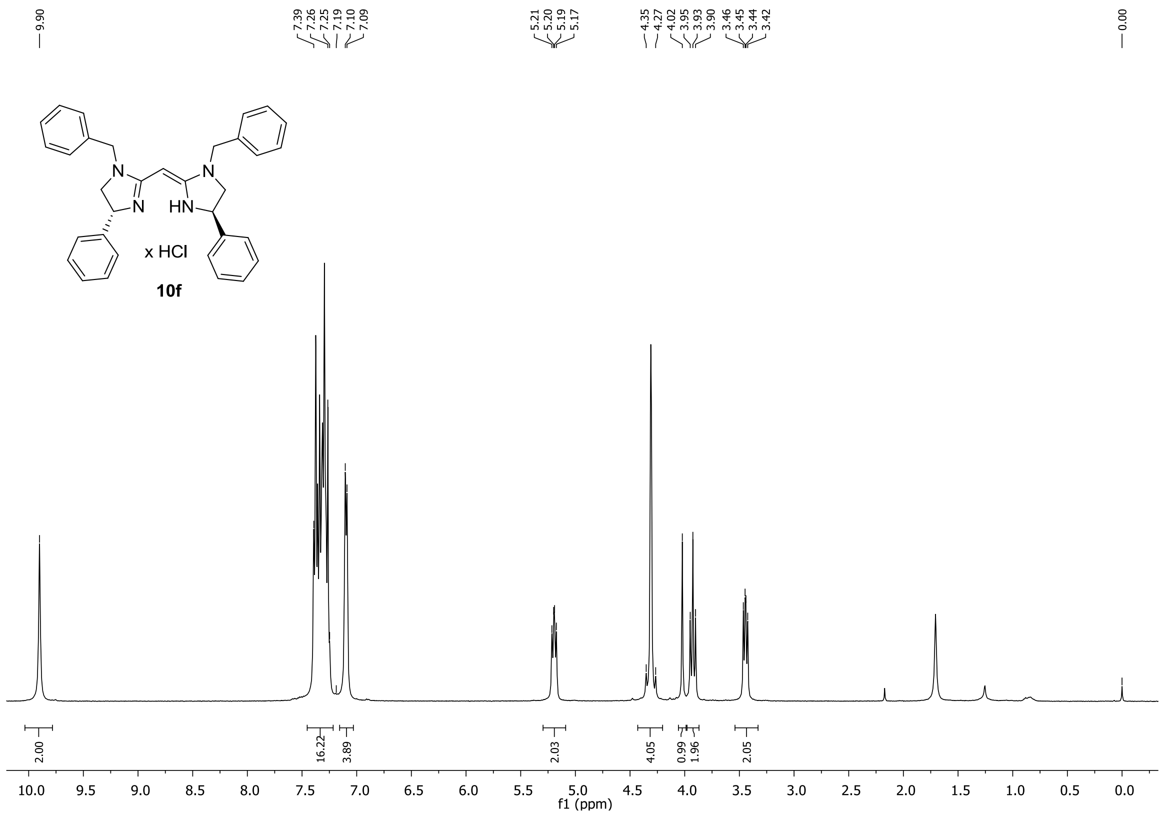
9.90
7.39
7.26
7.25
7.19
7.10
7.09
5.21
5.20
5.19
5.17
4.35
4.27
4.02
3.95
3.93
3.90
3.46
3.45
3.44
3.42
0.00
N
N
HN
x HCl
10f
2.00
16.22
3.89
2.03
4.05
0.99
1.96
2.05
f1 (ppm)

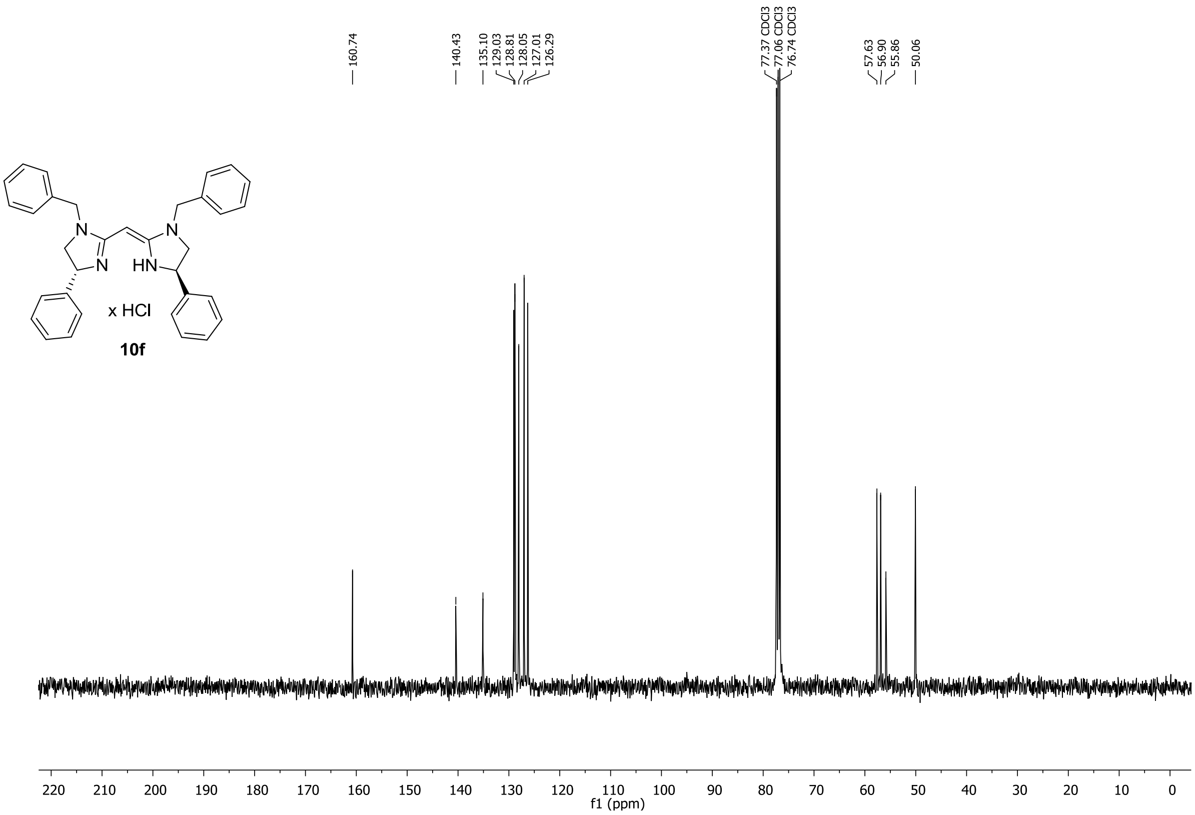
160.74
140.43
135.10
129.03
128.81
128.05
127.01
126.29
77.37 CDCl3
77.06 CDCl3
76.74 CDCl3
57.63
56.90
55.86
50.06
N
N
HN
x HCl
10f
f1 (ppm)

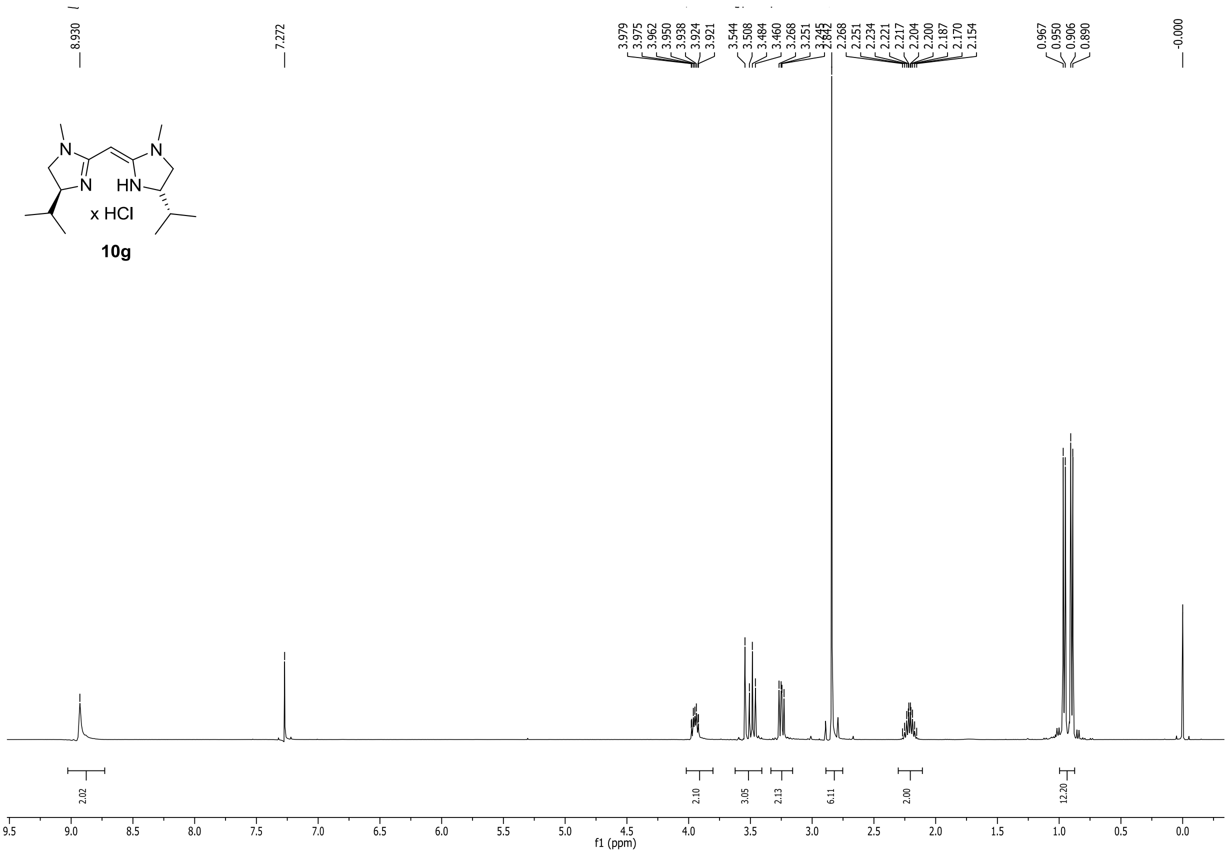
8.930
7.272
3.979 3.975 3.962 3.950 3.938 3.924 3.921
3.544 3.508 3.484 3.460 3.268 3.251
2.842
2.268 2.251 2.234 2.221 2.217 2.204 2.200 2.187 2.170 2.154
0.967 0.950 0.906 0.890
-0.000
x HCl
10g
2.02
2.10
3.05
2.13
6.11
2.00
12.20
f1 (ppm)

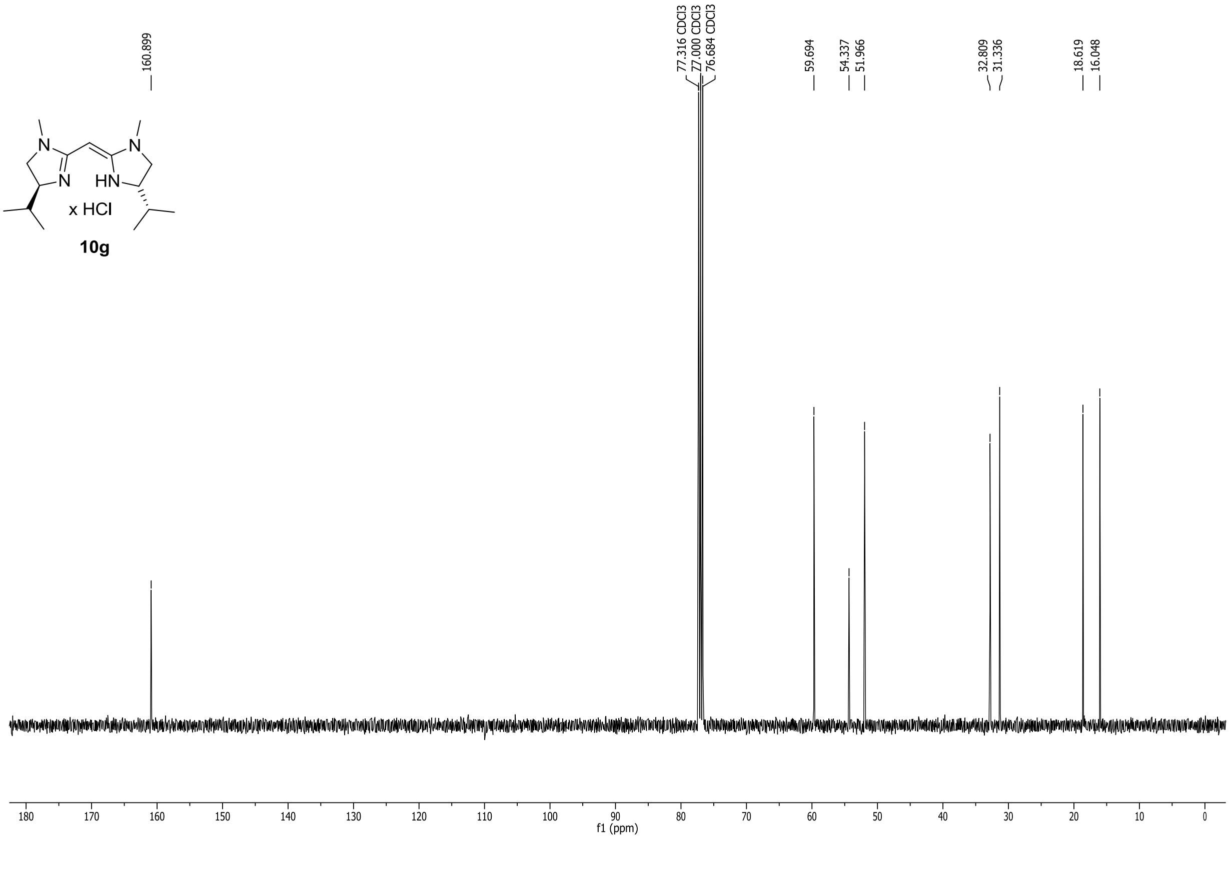
160.899
77.316 CDCl3
77.000 CDCl3
76.684 CDCl3
59.694
54.337
51.966
32.809
31.336
18.619
16.048
x HCl
10g
f1 (ppm)

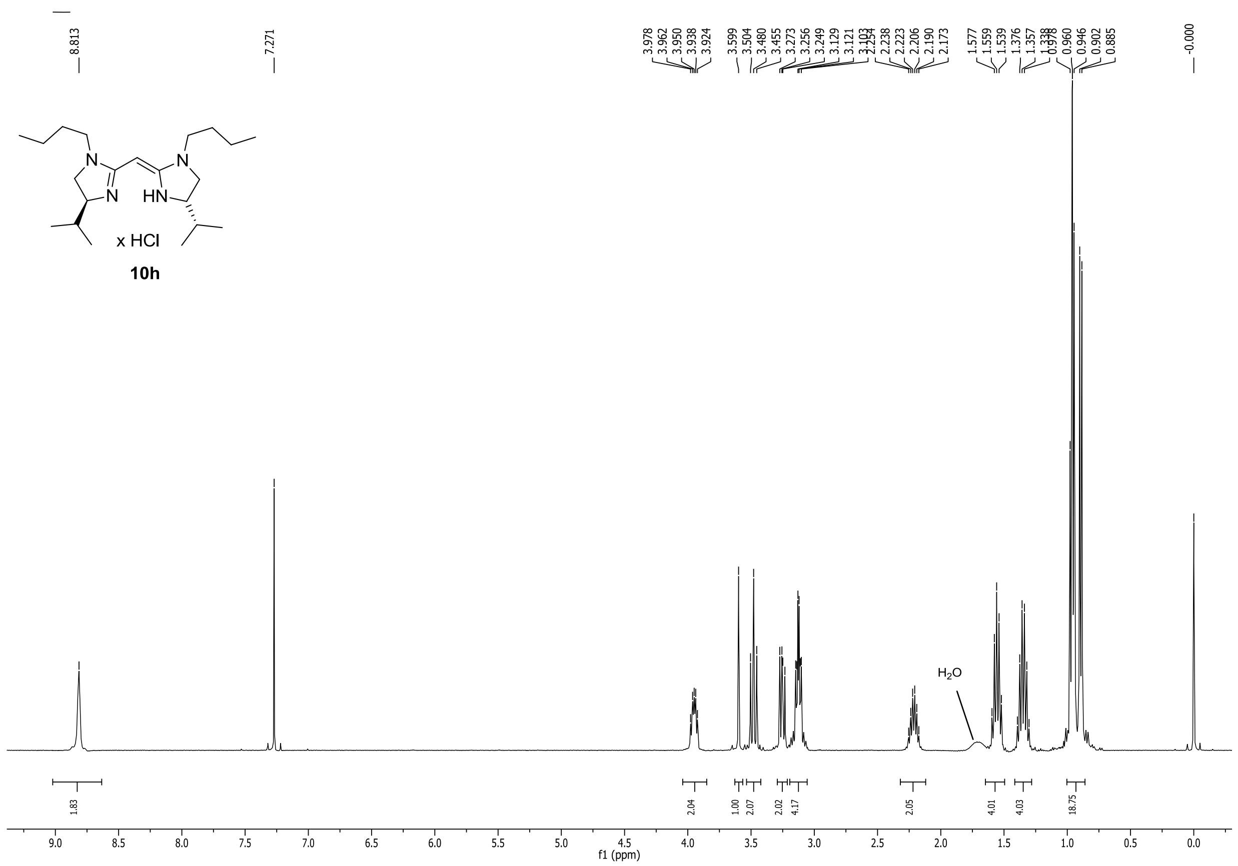
8.813
7.271
3.978
3.962
3.950
3.938
3.924
3.599
3.504
3.480
3.455
3.273
3.256
3.249
3.129
3.121
3.103
2.254
2.238
2.223
2.206
2.190
2.173
1.577
1.559
1.539
1.376
1.357
1.338
0.978
0.960
0.946
0.902
0.885
-0.000
N
N
N
HN
x HCl
10h
H2O
1.83
2.04
1.00
2.07
2.02
4.17
2.05
4.01
4.03
18.75
f1 (ppm)

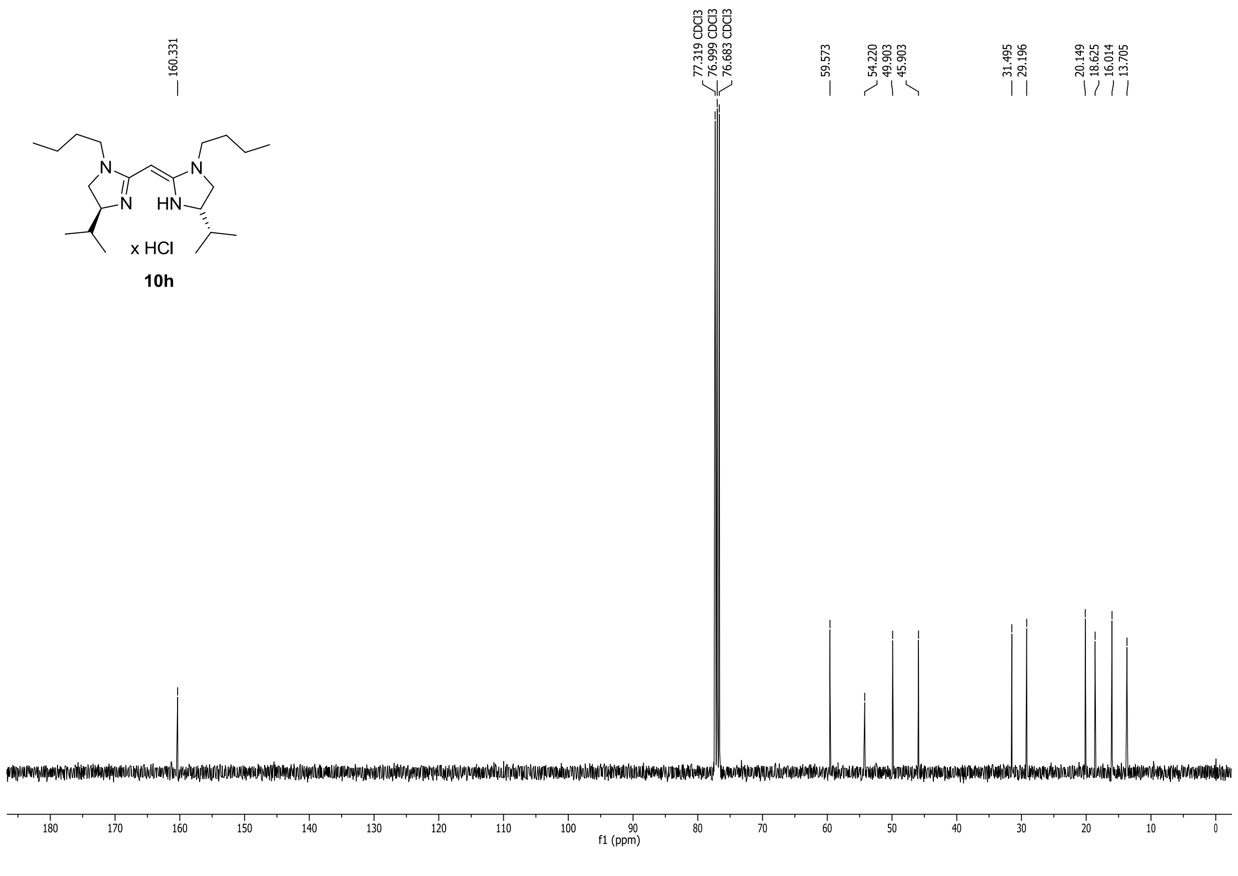
160.331
77.319 CDCl3
76.999 CDCl3
76.683 CDCl3
59.573
54.220
49.903
45.903
31.495
29.196
20.149
18.625
16.014
13.705
N
N
N
HN
x HCl
10h
f1 (ppm)

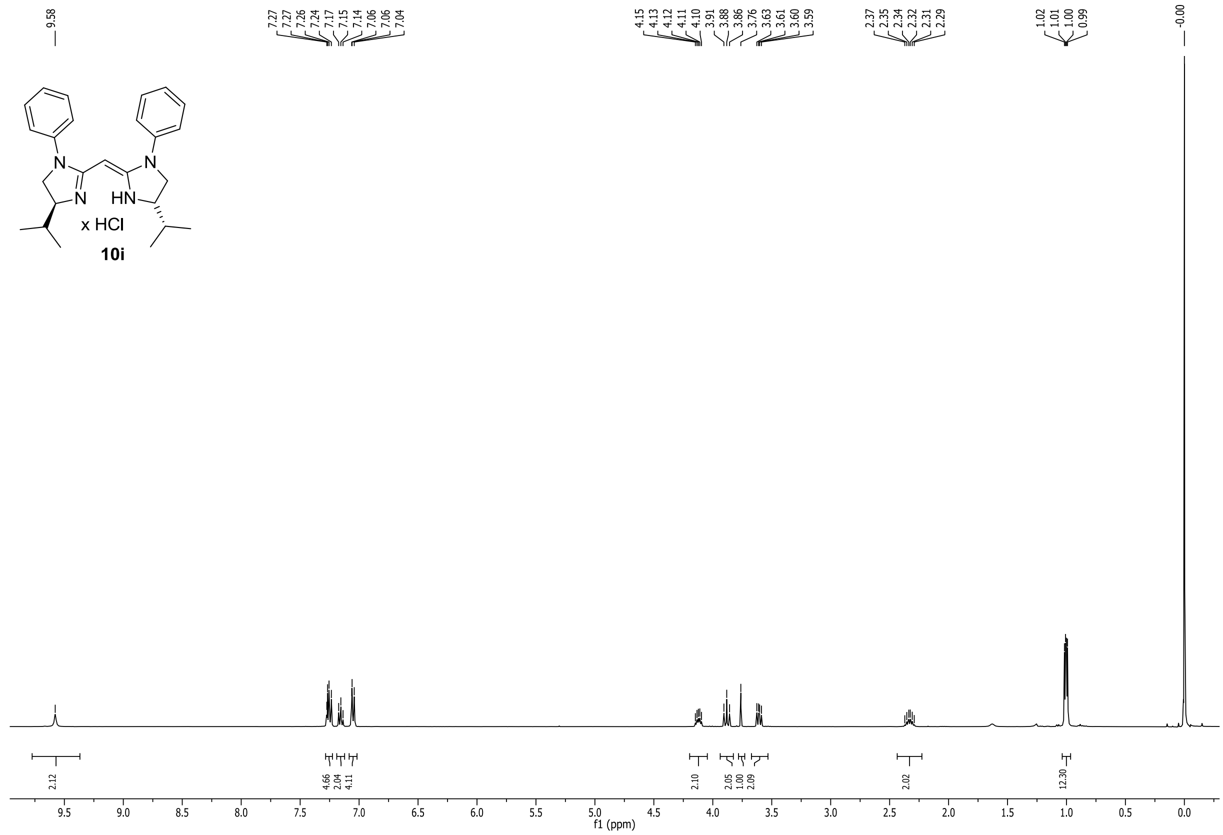
9.58
7.27
7.27
7.26
7.24
7.17
7.15
7.14
7.06
7.06
7.04
4.15
4.13
4.12
4.11
4.10
3.91
3.88
3.86
3.76
3.63
3.61
3.60
3.59
2.37
2.35
2.34
2.32
2.31
2.29
1.02
1.01
1.00
0.99
-0.00
x HCl
10i
2.12
4.66
2.04
4.11
2.10
2.05
1.00
2.09
2.02
12.30
f1 (ppm)

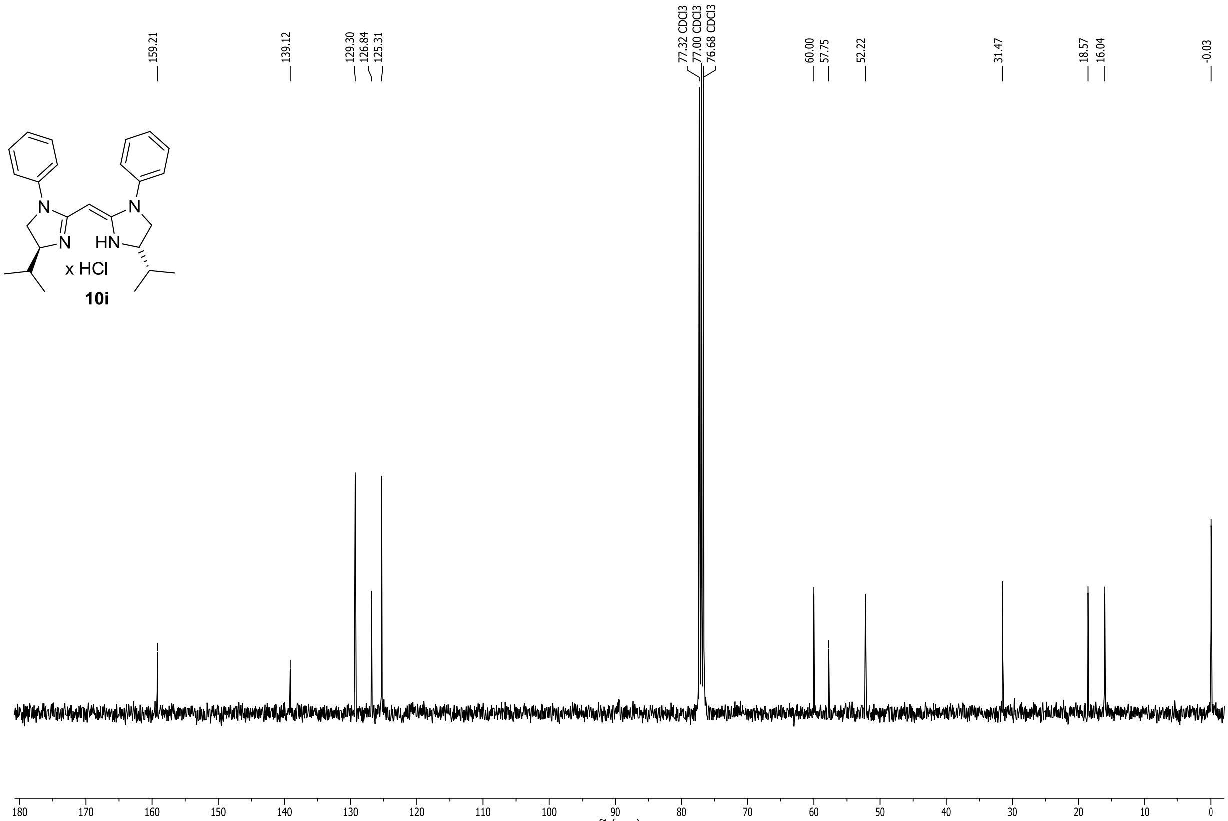
159.21
139.12
129.30
126.84
125.31
77.32 CDCl3
77.00 CDCl3
76.68 CDCl3
60.00
57.75
52.22
31.47
18.57
16.04
-0.03
x HCl
10i
f1 (ppm)

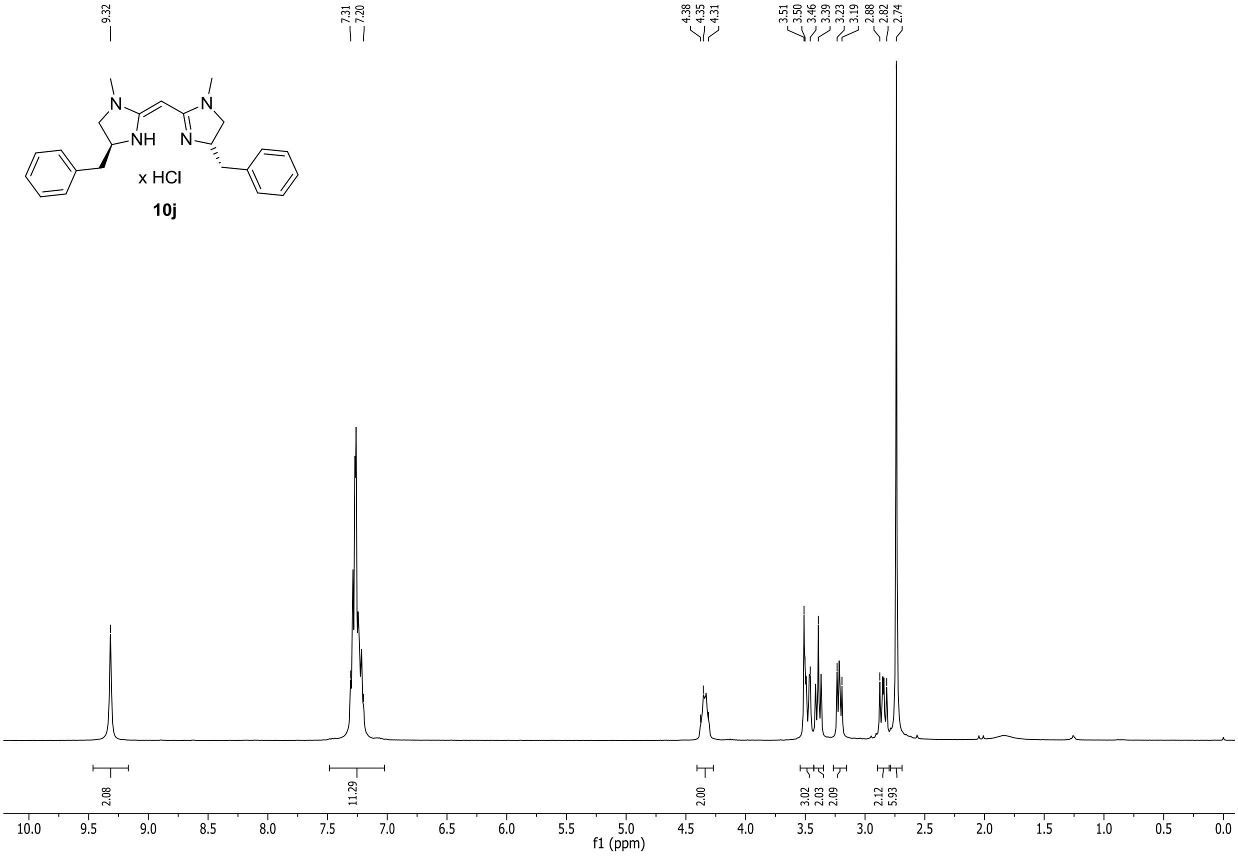
9.32
7.31
7.20
4.38
4.35
4.31
3.51
3.50
3.46
3.39
3.23
3.19
2.88
2.82
2.74
NH
N
N
N
x HCl
10j
2.08
11.29
2.00
3.02
2.03
2.09
2.12
5.93
f1 (ppm)

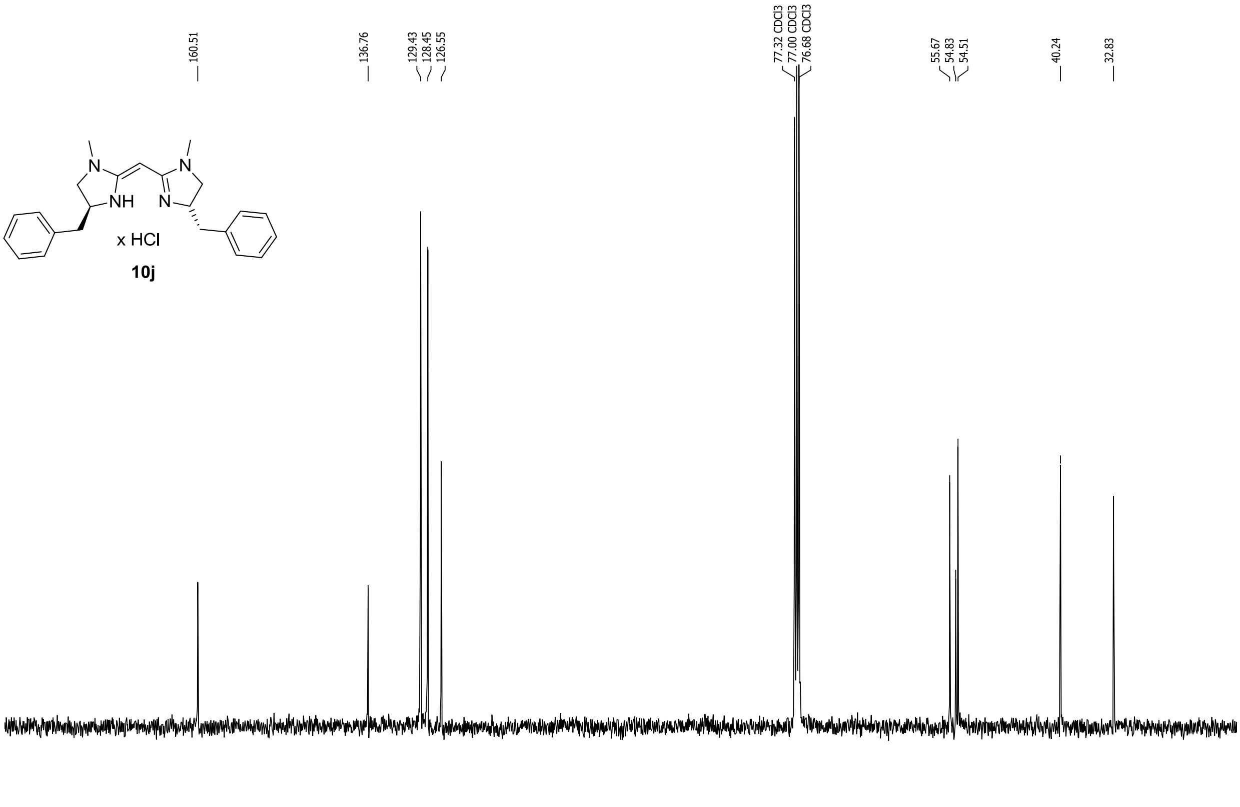
160.51
136.76
129.43
128.45
126.55
77.32 CDCl3
77.00 CDCl3
76.68 CDCl3
55.67
54.83
54.51
40.24
32.83
NH
N
N
N
x HCl
10j
f1 (ppm)

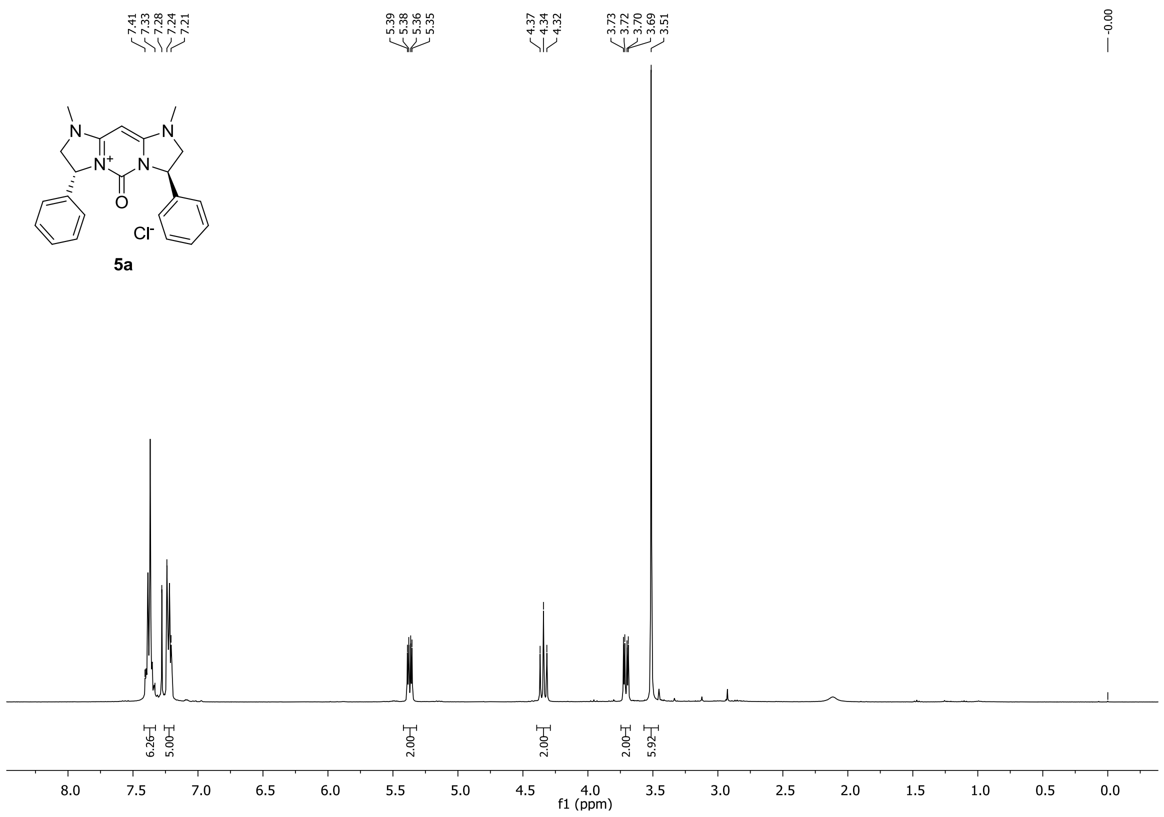
7.41
7.33
7.28
7.24
7.21
5.39
5.38
5.36
5.35
4.37
4.34
4.32
3.73
3.72
3.70
3.69
3.51
-0.00
Cl⁻
5a
6.26
5.00
2.00
2.00
2.00
5.92
f1 (ppm)

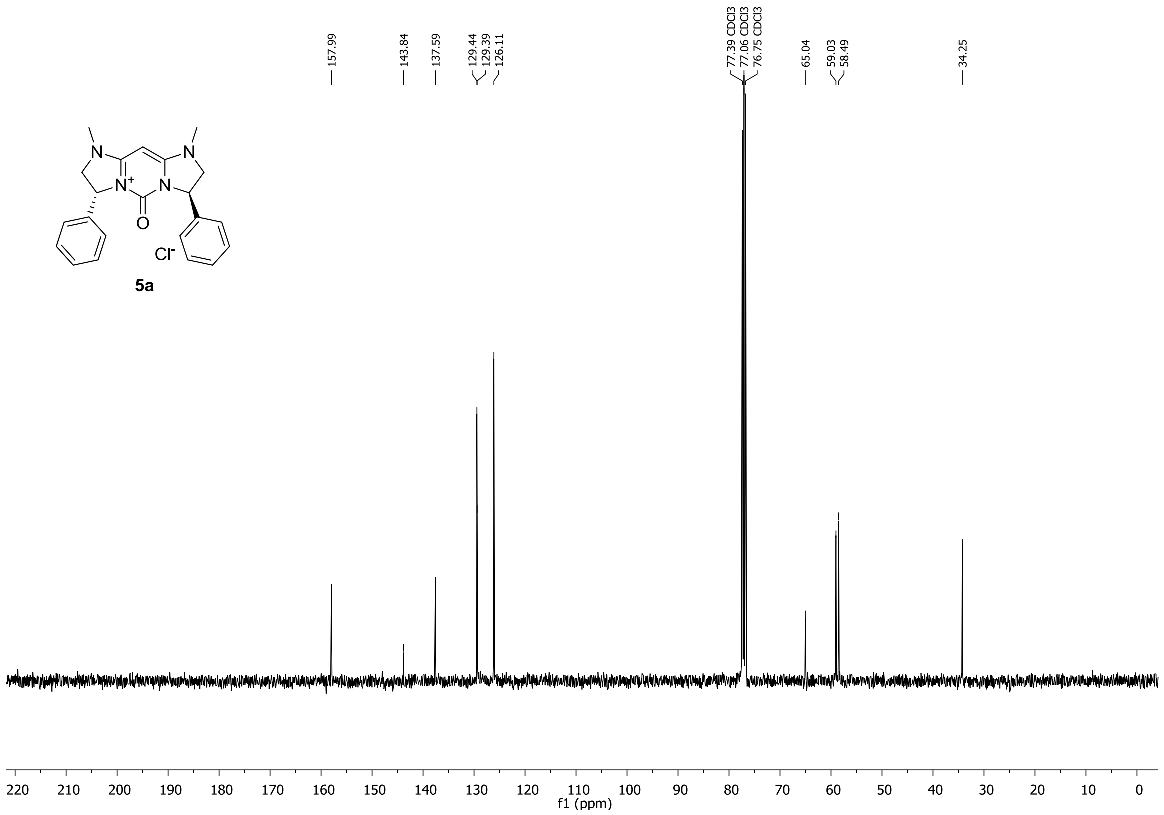
157.99
143.84
137.59
129.44
129.39
126.11
77.39 CDCl3
77.06 CDCl3
76.75 CDCl3
65.04
59.03
58.49
34.25
Cl⁻
5a
f1 (ppm)

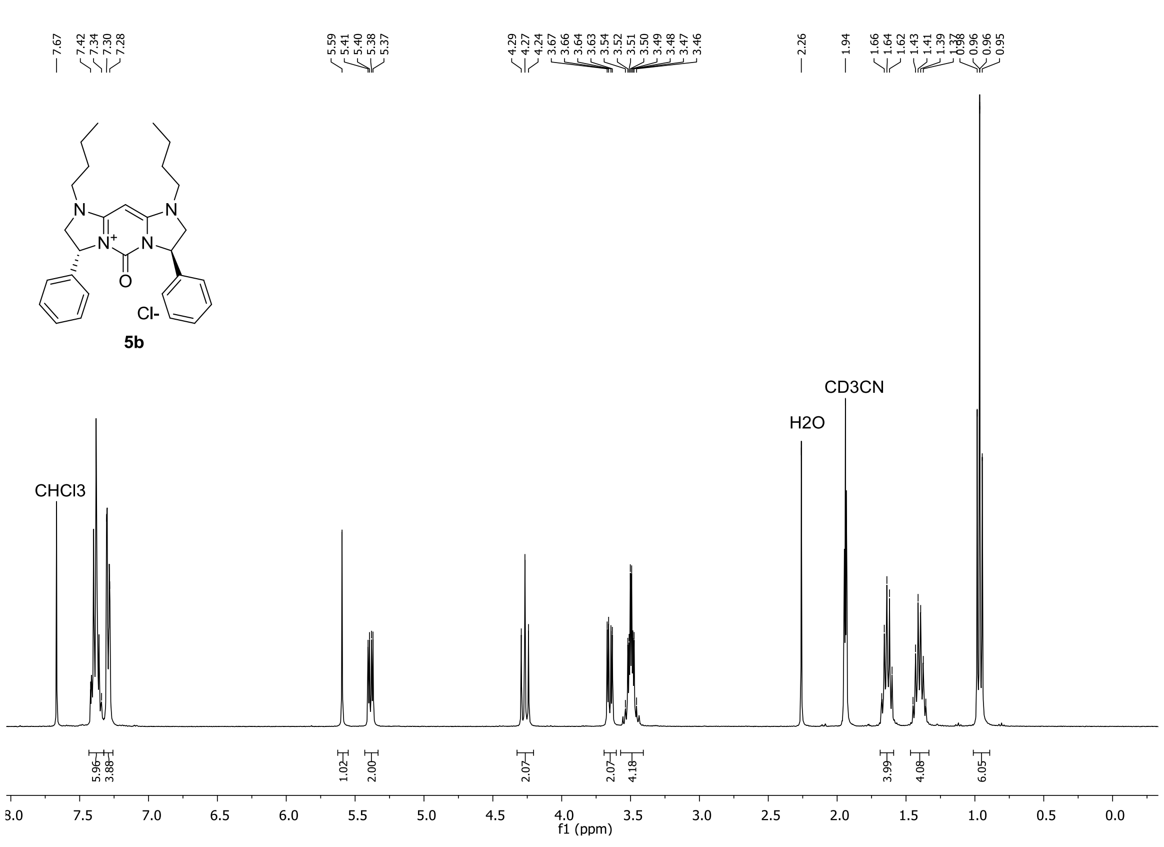
7.67
7.42
7.34
7.30
7.28
5.59
5.41
5.40
5.38
5.37
4.29
4.27
4.24
3.67
3.66
3.64
3.63
3.54
3.52
3.51
3.50
3.49
3.48
3.47
3.46
2.26
1.94
1.66
1.64
1.62
1.43
1.41
1.39
0.96
0.95
N
N
N+
N
O
Cl-
5b
CHCl3
H2O
CD3CN
5.96
3.88
1.02
2.00
2.07
2.07
4.18
3.99
4.08
6.05
f1 (ppm)

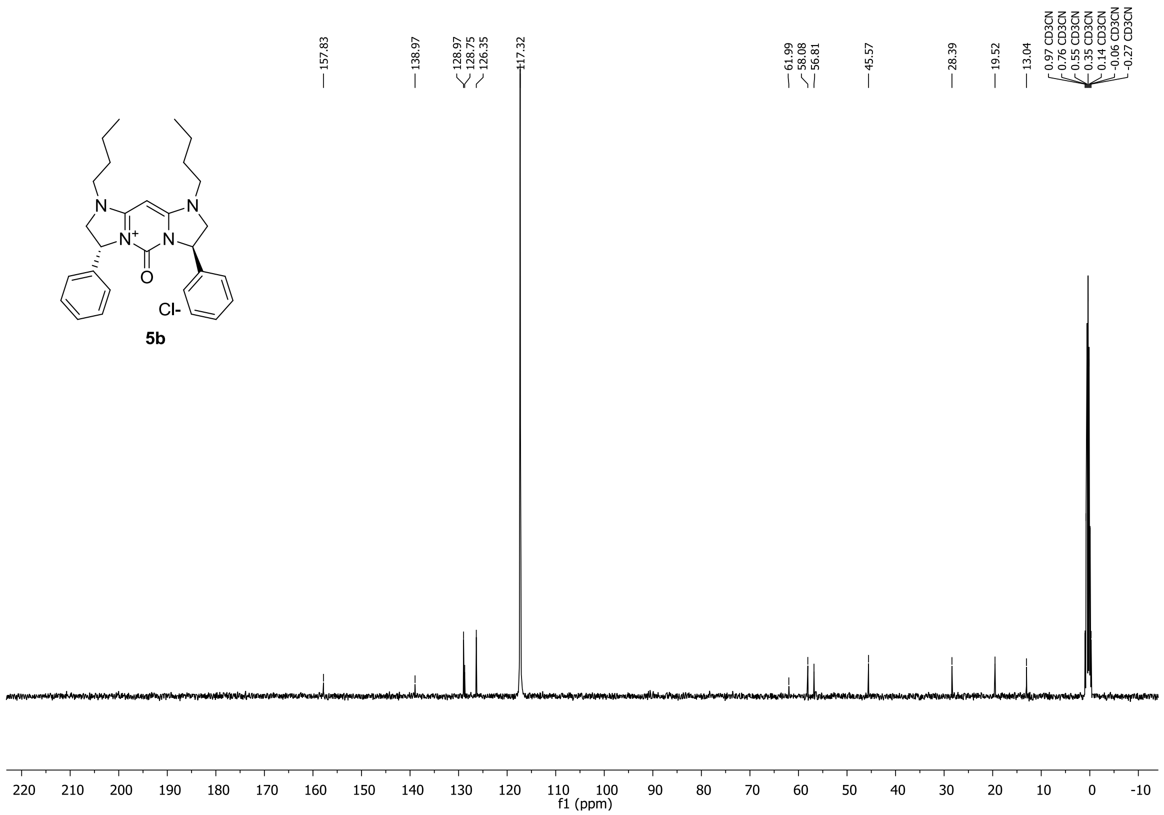
157.83
138.97
128.97
128.75
126.35
117.32
61.99
58.08
56.81
45.57
28.39
19.52
13.04
0.97 CD3CN
0.76 CD3CN
0.55 CD3CN
0.35 CD3CN
0.14 CD3CN
-0.06 CD3CN
-0.27 CD3CN
N
N
N+
N
O
Cl-
5b
f1 (ppm)

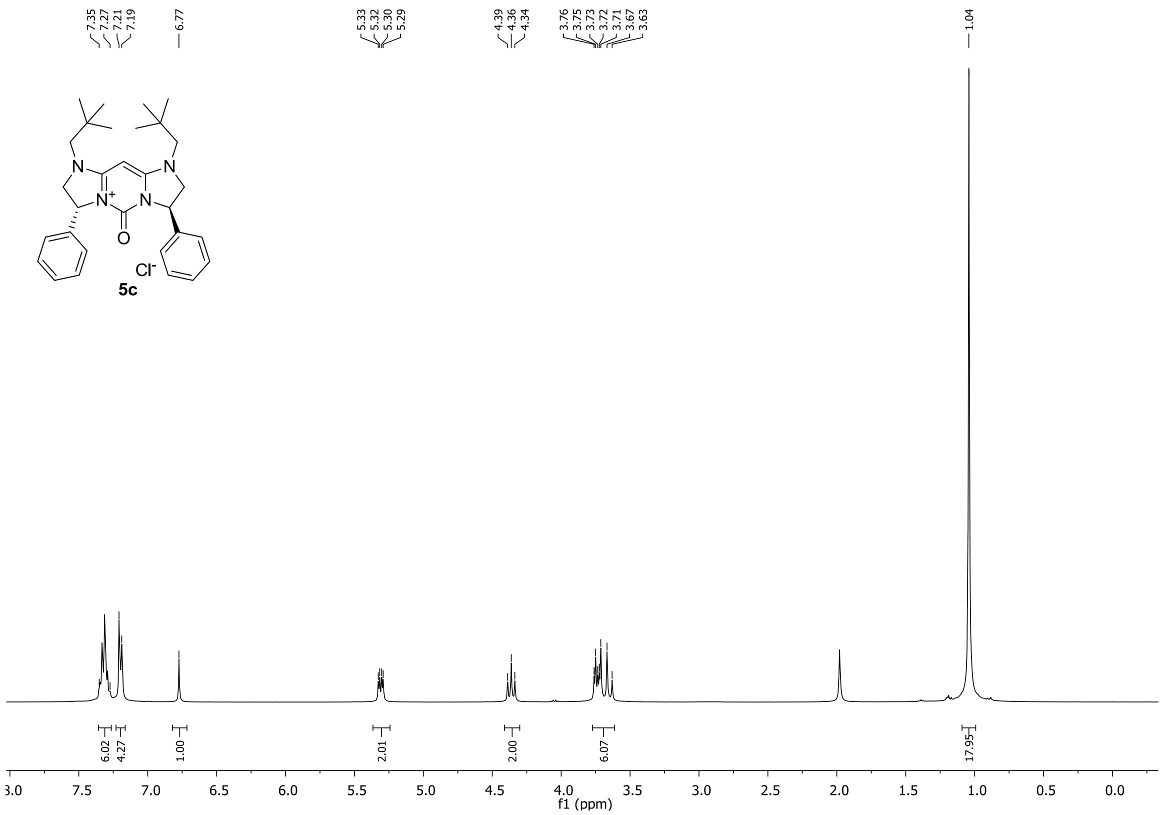
7.35
7.27
7.21
7.19
6.77
5.33
5.32
5.30
5.29
4.39
4.36
4.34
3.76
3.75
3.73
3.72
3.71
3.67
3.63
1.04
N
N+
N
N
O
Cl-
5c
6.02
4.27
1.00
2.01
2.00
6.07
17.95
f1 (ppm)

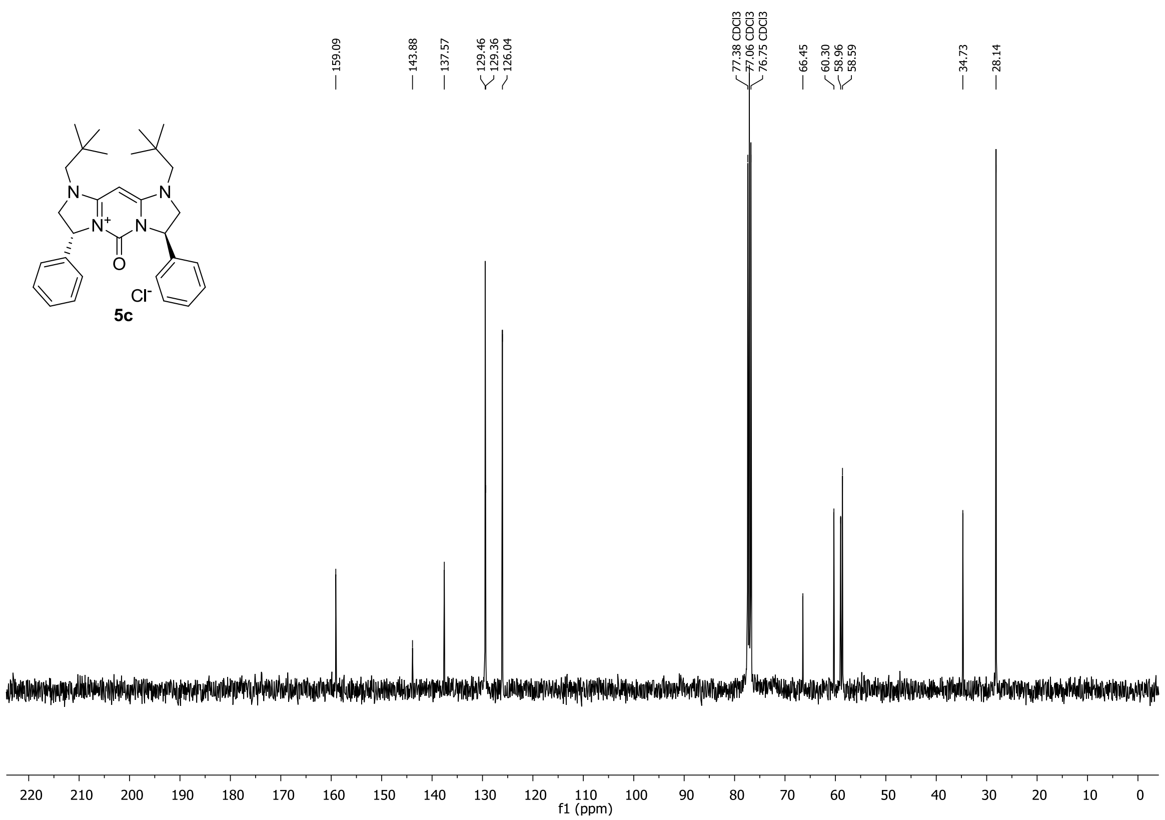
159.09
143.88
137.57
129.46
129.36
126.04
77.38 CDCl3
77.06 CDCl3
76.75 CDCl3
66.45
60.30
58.96
58.59
34.73
28.14
N
N+
N
N
O
Cl-
5c
f1 (ppm)

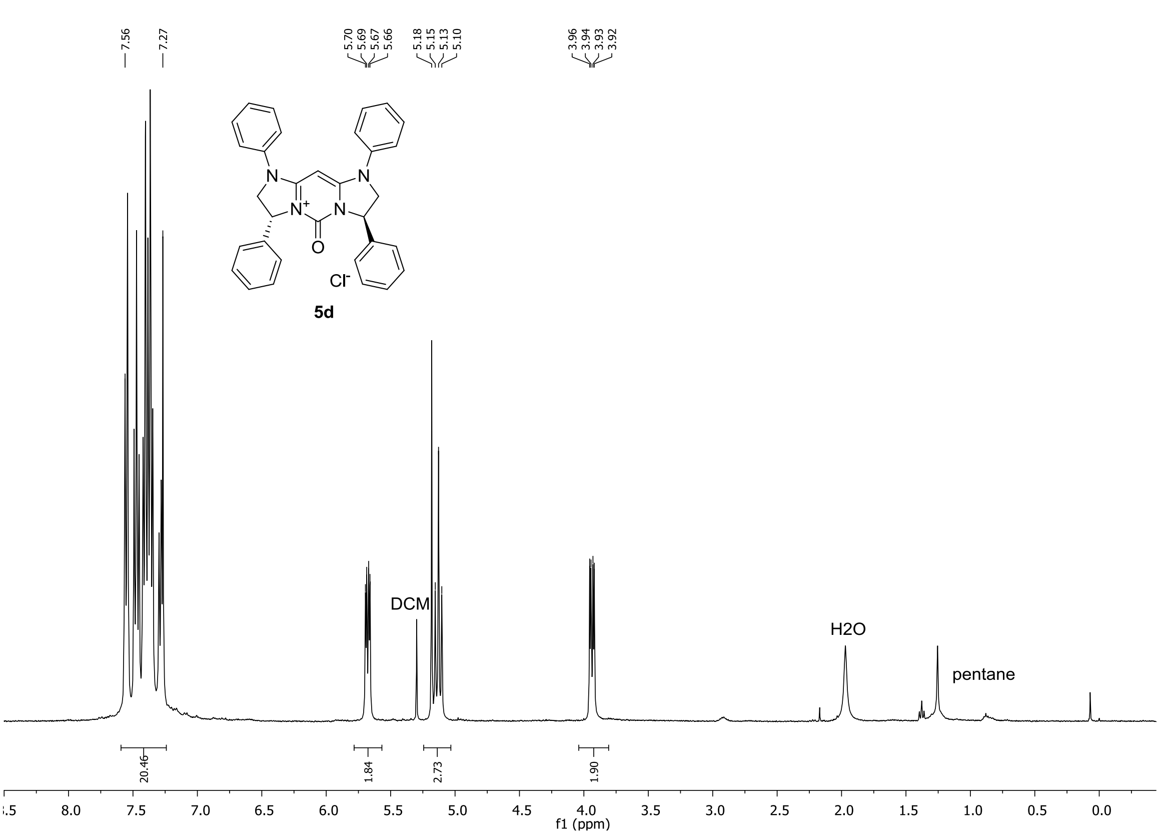
7.56
7.27
5.70
5.69
5.67
5.66
5.18
5.15
5.13
5.10
3.96
3.94
3.93
3.92
N
N
N+
N
N
O
Cl-
5d
DCM
H2O
pentane
20.46
1.84
2.73
1.90
f1 (ppm)

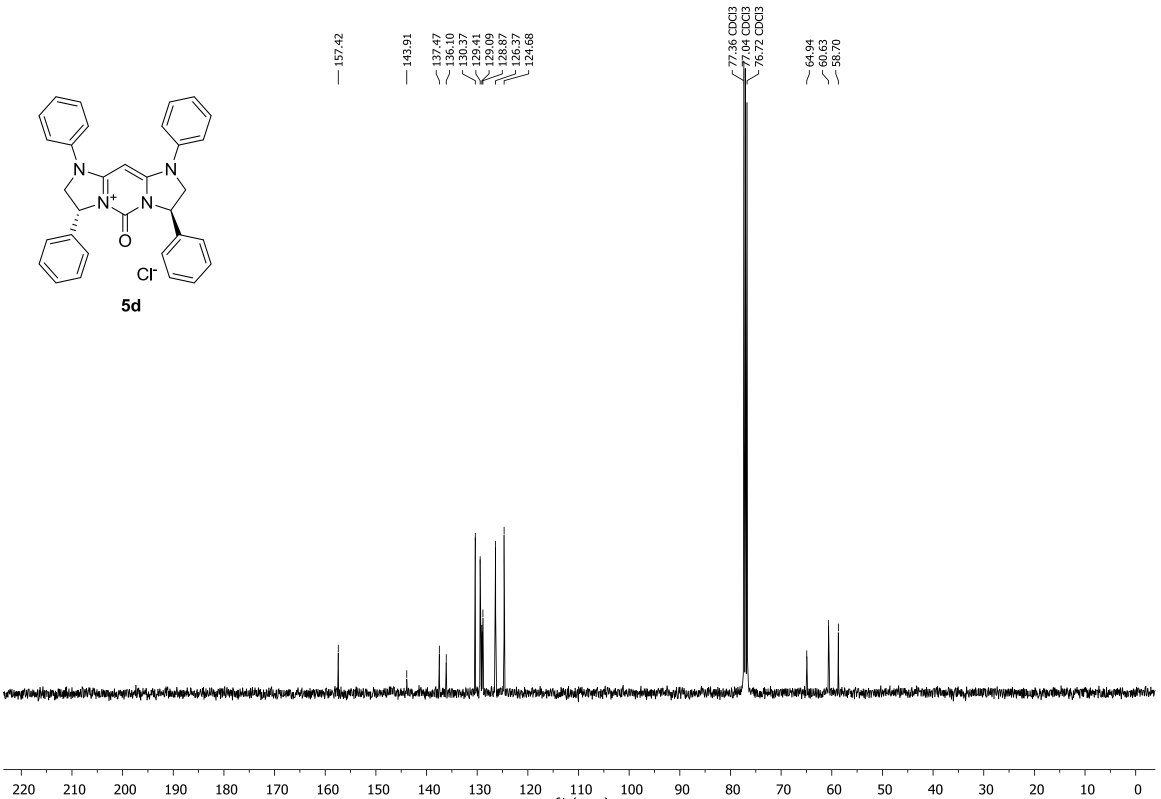
157.42
143.91
137.47
136.10
130.37
129.41
129.09
128.87
126.37
124.68
77.36 CDCl3
77.04 CDCl3
76.72 CDCl3
64.94
60.63
58.70
N
N
N+
N
N
O
Cl-
5d
f1 (ppm)

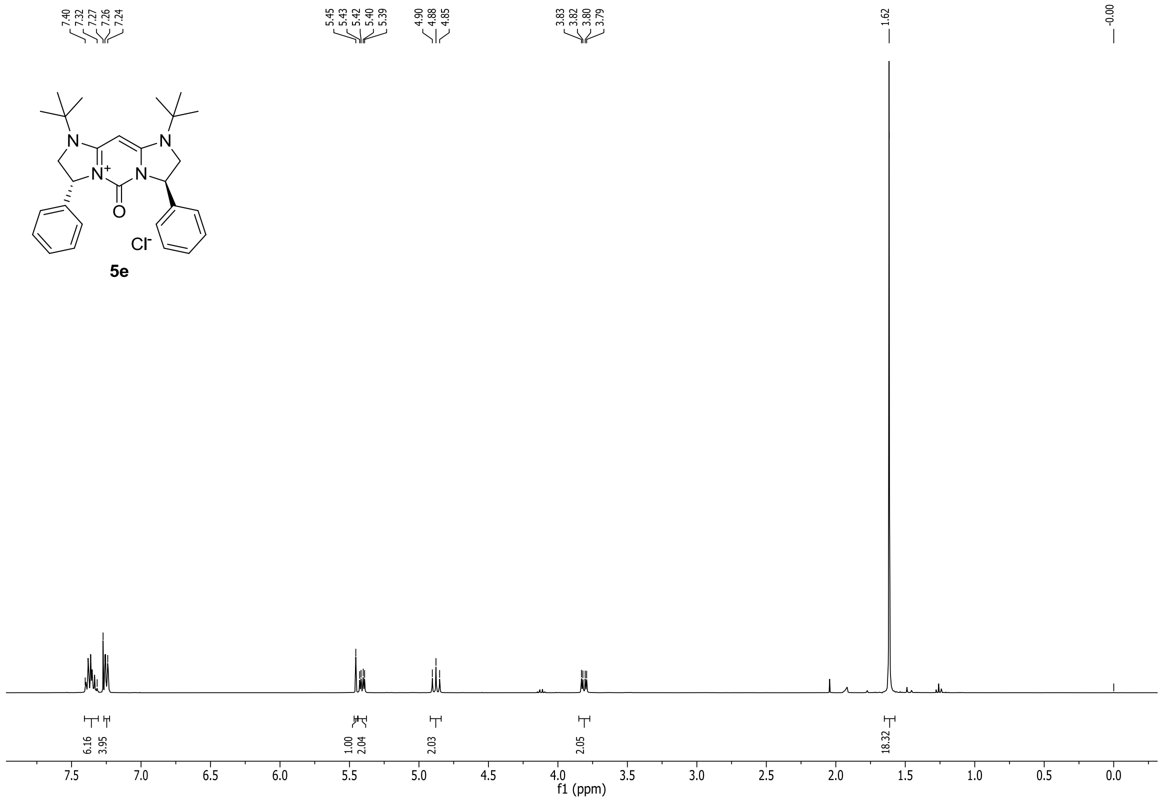
7.40
7.32
7.27
7.26
7.24
5.45
5.43
5.42
5.40
5.39
4.90
4.88
4.85
3.83
3.82
3.80
3.79
1.62
-0.00
N
N
N+
N
O
Cl-
5e
6.16
3.95
1.00
2.04
2.03
2.05
18.32
f1 (ppm)

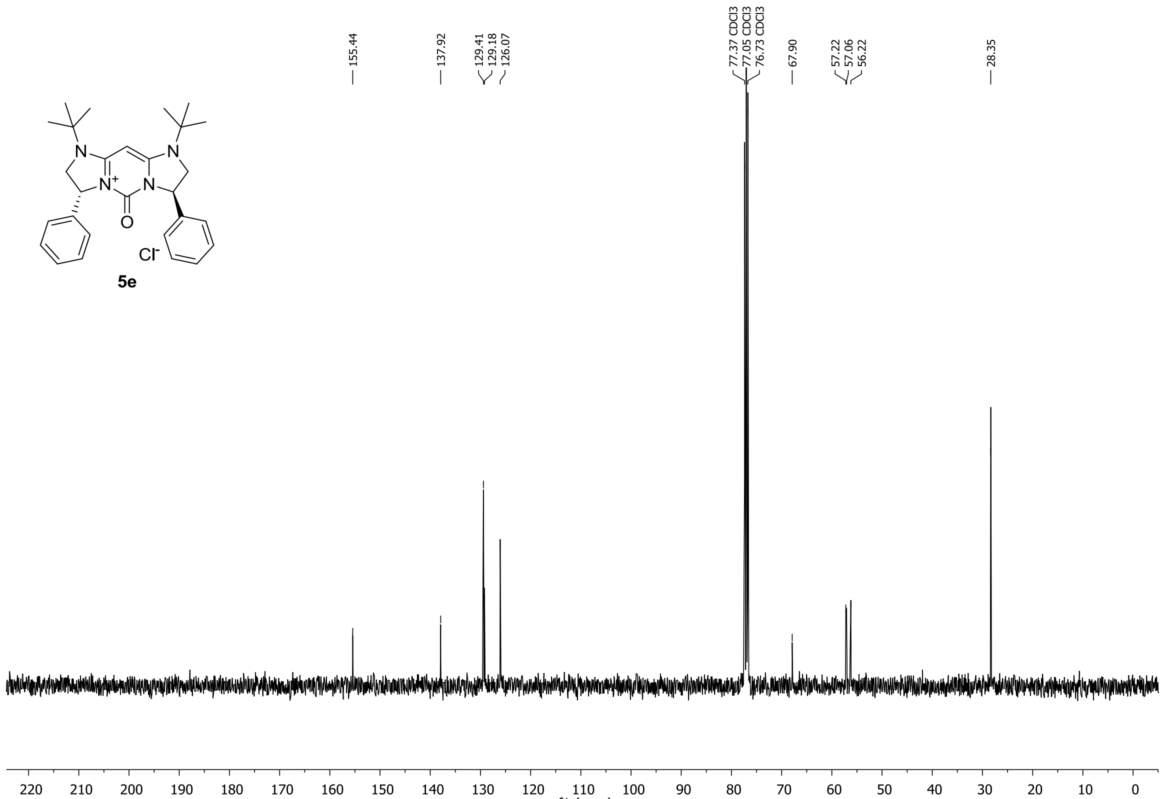
155.44
137.92
129.41
129.18
126.07
77.37 CDCl3
77.05 CDCl3
76.73 CDCl3
67.90
57.22
57.06
56.22
28.35
N
N
N+
N
O
Cl-
5e
f1 (ppm)

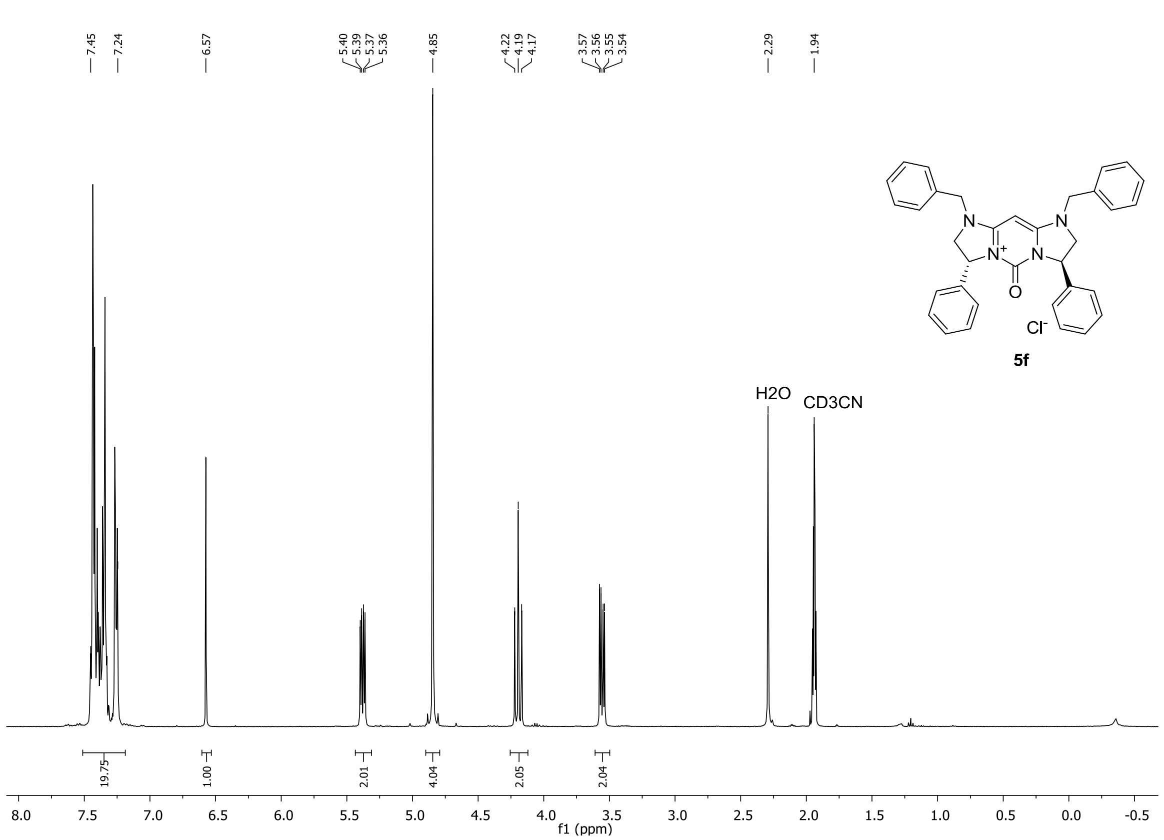
7.45
7.24
6.57
5.40
5.39
5.37
5.36
4.85
4.22
4.19
4.17
3.57
3.56
3.55
3.54
2.29
1.94
H2O
CD3CN
Cl-
5f
19.75
1.00
2.01
4.04
2.05
2.04
f1 (ppm)

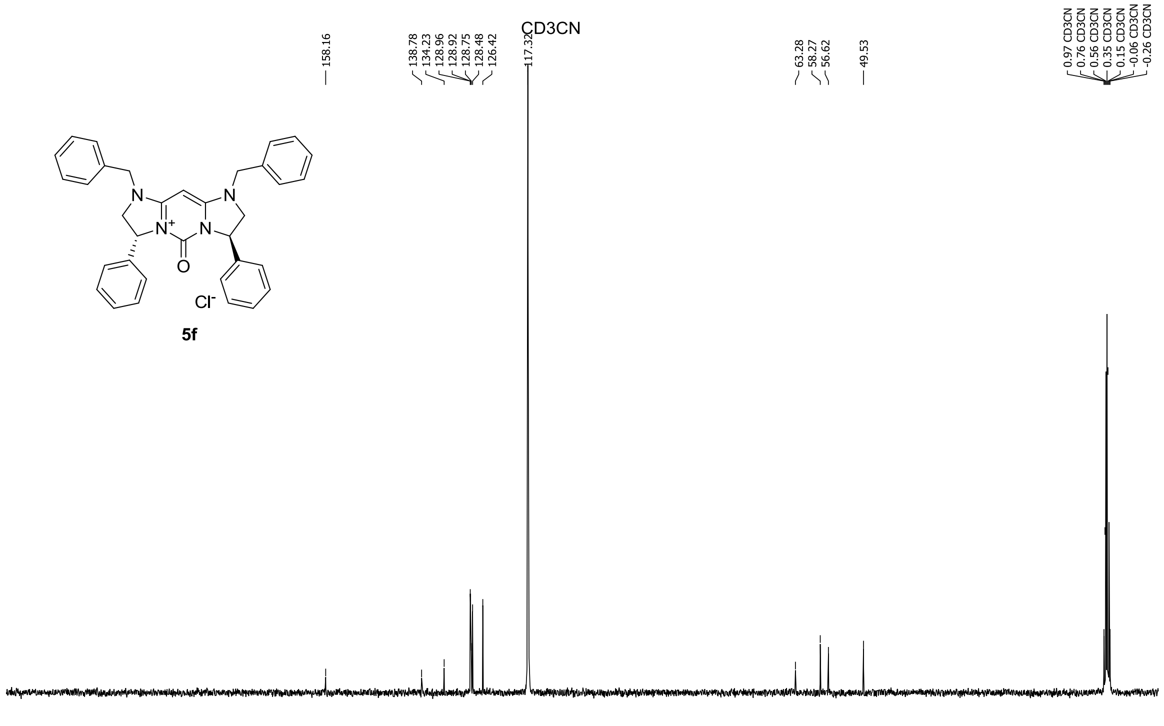
158.16
138.78
134.23
128.96
128.92
128.75
128.48
126.42
117.32
CD3CN
63.28
58.27
56.62
49.53
0.97 CD3CN
0.76 CD3CN
0.56 CD3CN
0.35 CD3CN
0.15 CD3CN
-0.06 CD3CN
-0.26 CD3CN
Cl-
5f
f1 (ppm)

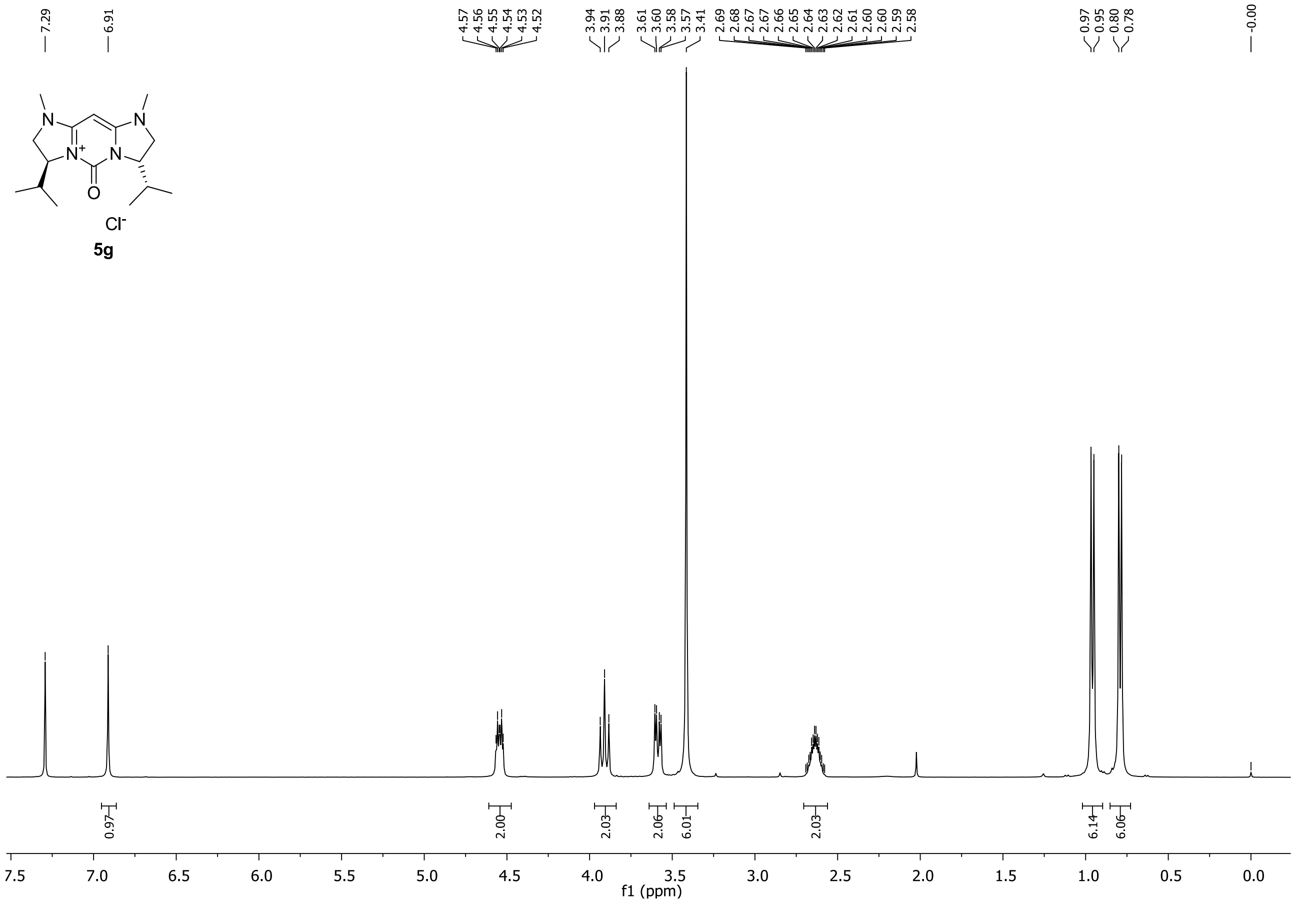
7.29
6.91
4.57 4.56 4.55 4.54 4.53 4.52
3.94 3.91 3.88
3.61 3.60 3.58 3.57 3.41
2.69 2.68 2.67 2.67 2.66 2.65 2.64 2.63 2.62 2.62 2.61 2.60 2.60 2.59 2.58
0.97 0.95 0.80 0.78
-0.00
0.97
2.00
2.03
2.06
6.01
2.03
6.14
6.06
f1 (ppm)

5g

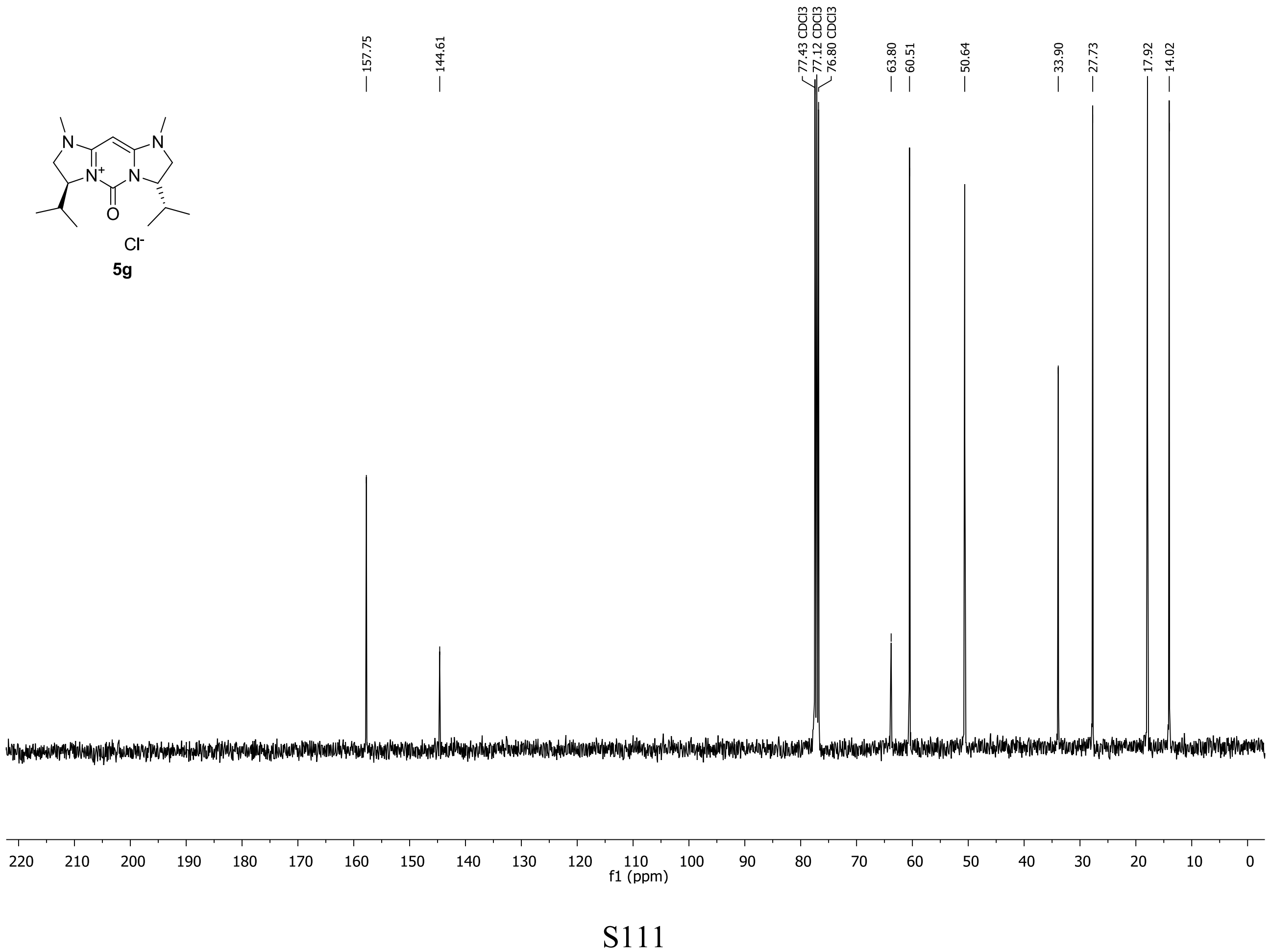
157.75
144.61
77.43 CDCl3
77.12 CDCl3
76.80 CDCl3
63.80
60.51
50.64
33.90
27.73
17.92
14.02
f1 (ppm)

5g

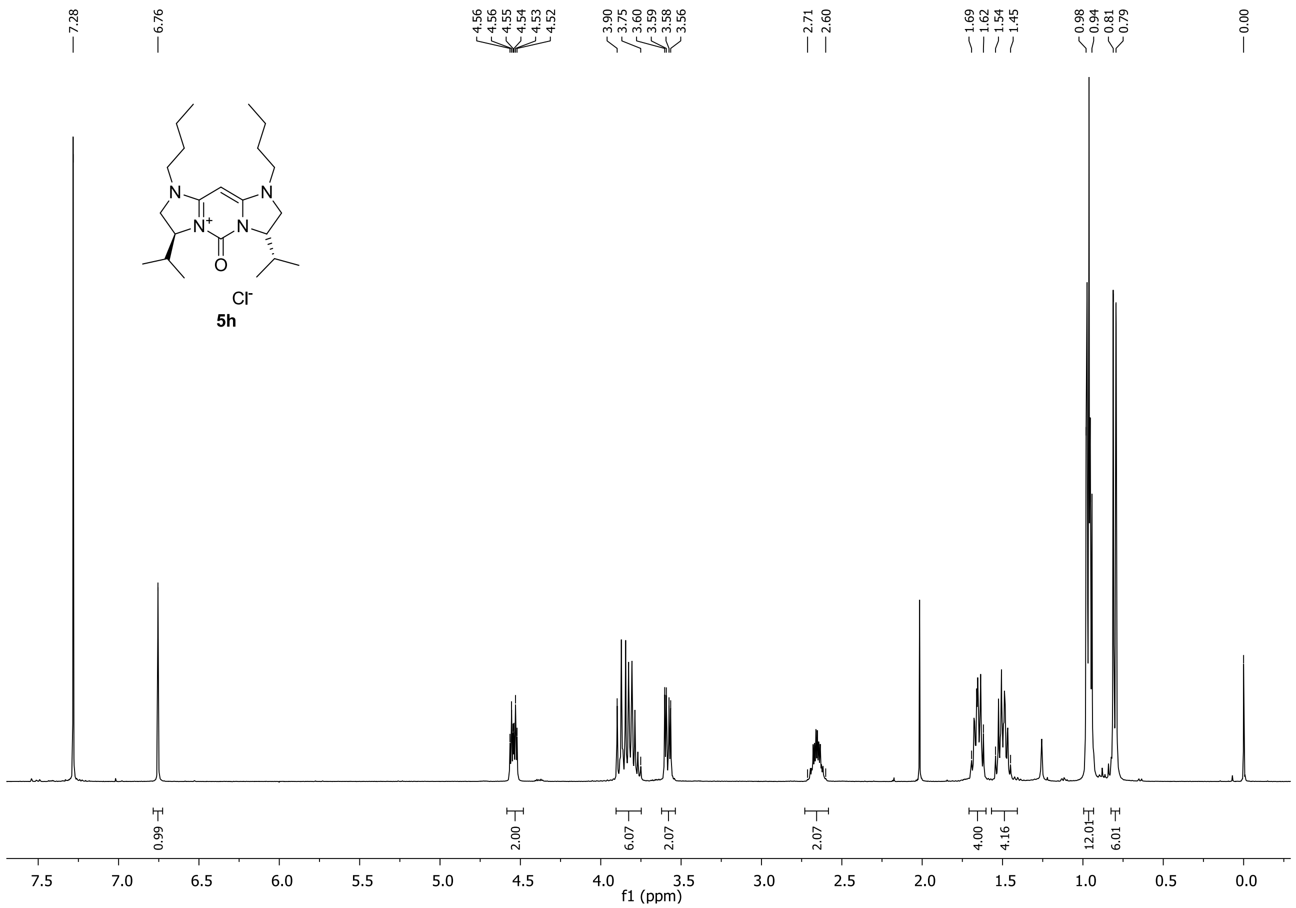
7.28
6.76
4.56
4.56
4.55
4.54
4.53
4.52
3.90
3.75
3.60
3.59
3.58
3.56
2.71
2.60
1.69
1.62
1.54
1.45
0.98
0.94
0.81
0.79
0.00
Cl-
5h
0.99
2.00
6.07
2.07
2.07
4.00
4.16
12.01
6.01
f1 (ppm)

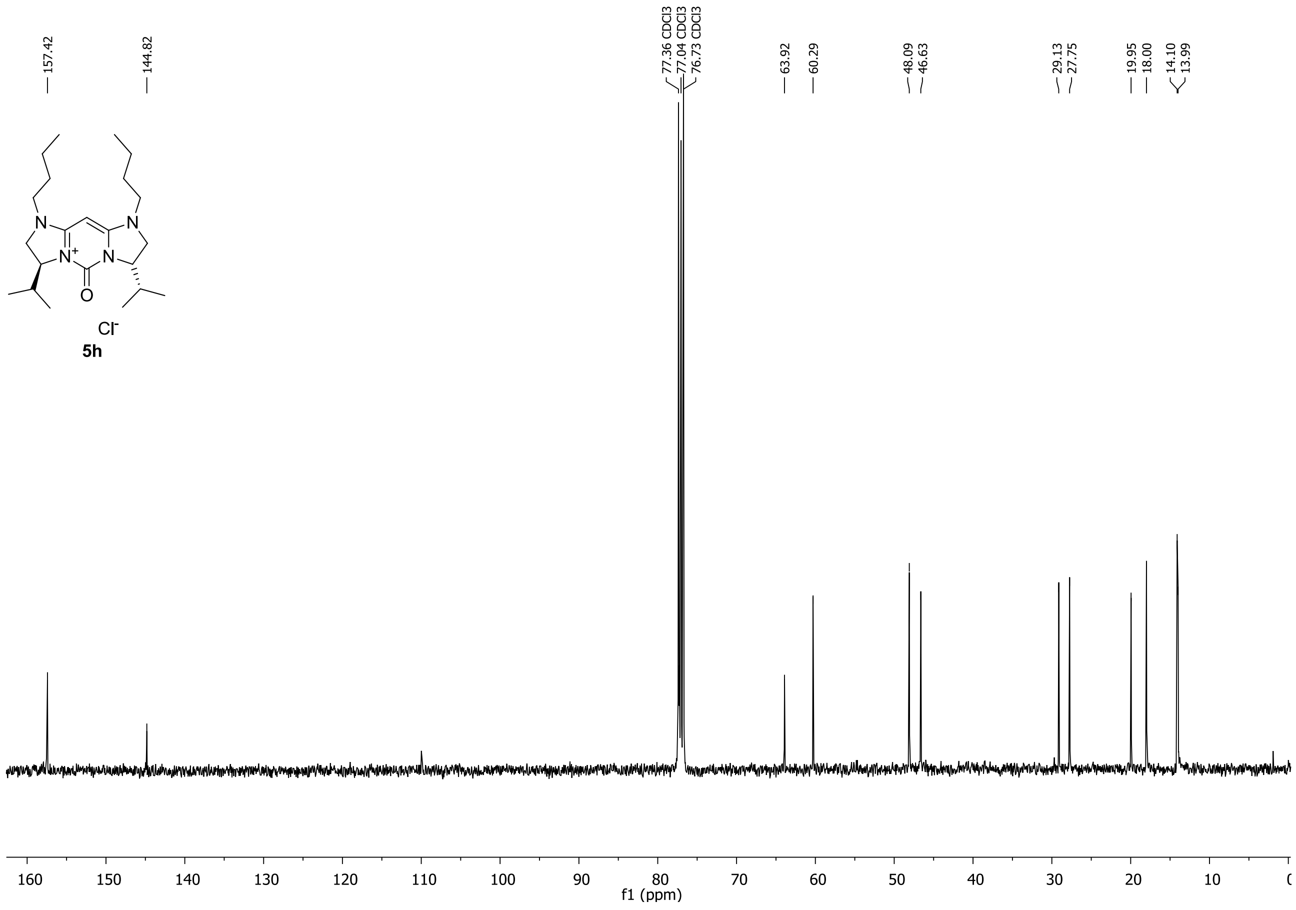
157.42
144.82
77.36 CDCl3
77.04 CDCl3
76.73 CDCl3
63.92
60.29
48.09
46.63
29.13
27.75
19.95
18.00
14.10
13.99
Cl-
5h
f1 (ppm)

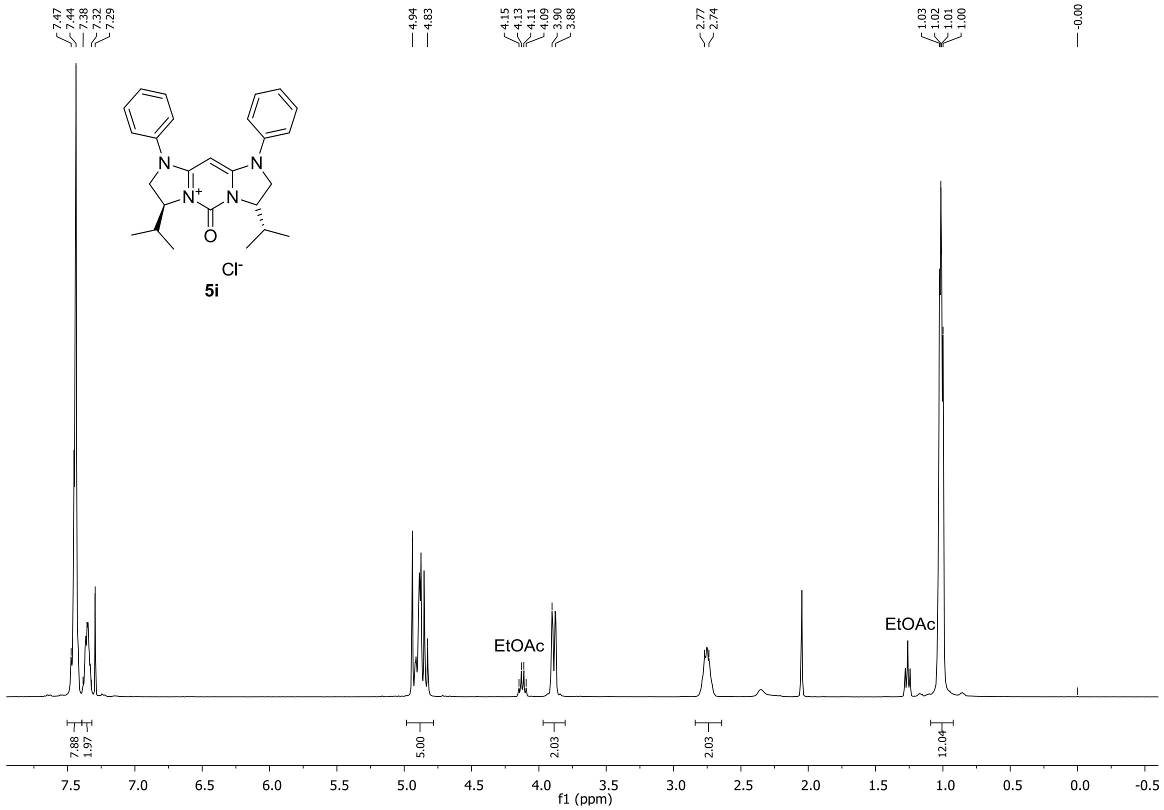
7.47
7.44
7.38
7.32
7.29
4.94
4.83
4.15
4.13
4.11
4.09
3.90
3.88
2.77
2.74
1.03
1.02
1.01
1.00
-0.00
N
N+
N
N
O
Cl-
5i
EtOAc
EtOAc
7.88
1.97
5.00
2.03
2.03
12.04
f1 (ppm)

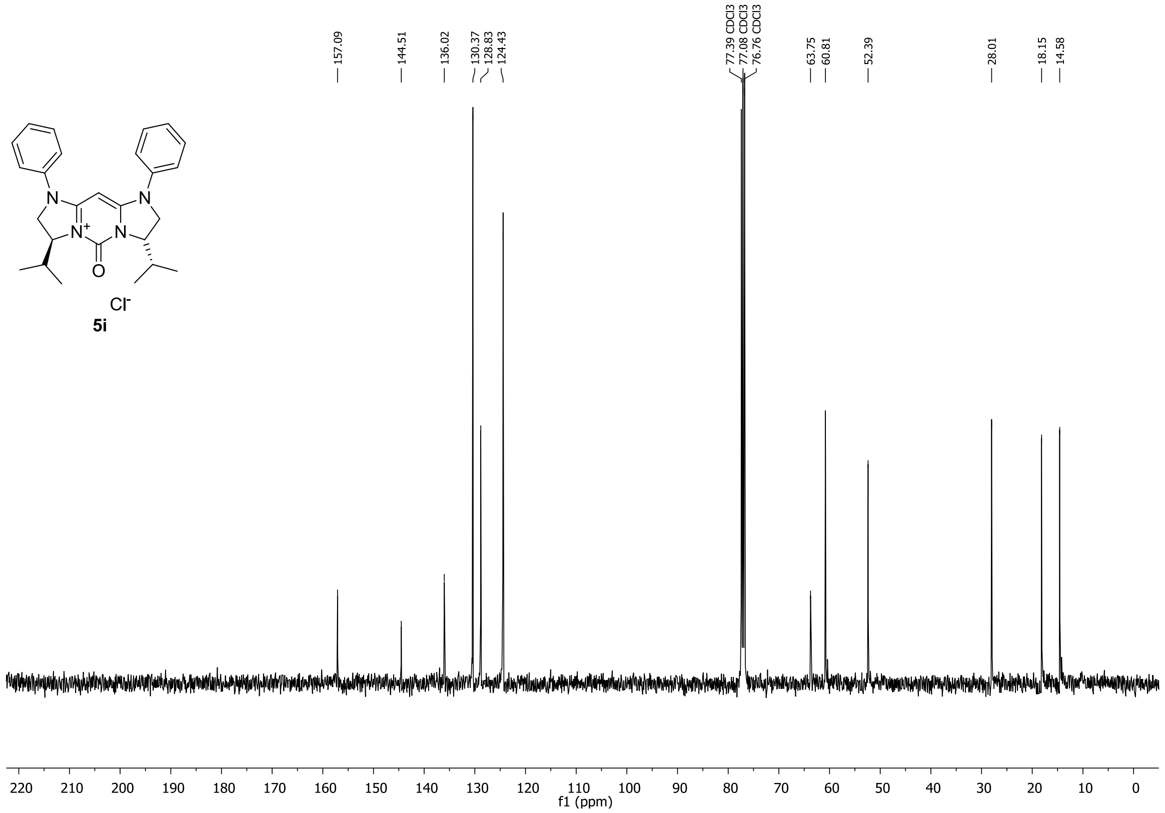
157.09
144.51
136.02
130.37
128.83
124.43
77.39 CDCl3
77.08 CDCl3
76.76 CDCl3
63.75
60.81
52.39
28.01
18.15
14.58
N
N+
N
N
O
Cl-
5i
f1 (ppm)

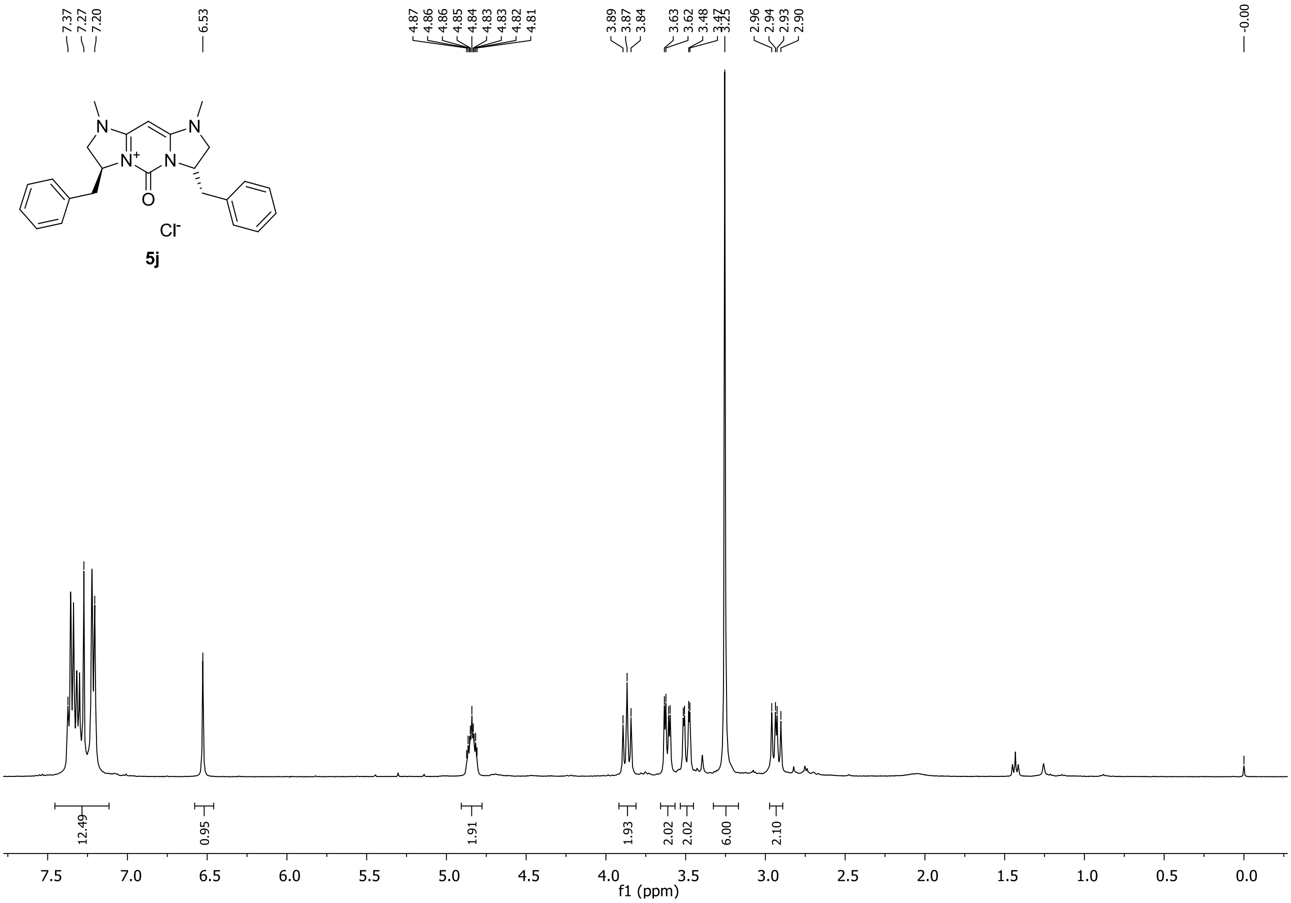
7.37
7.27
7.20
6.53
4.87
4.86
4.86
4.85
4.84
4.83
4.83
4.82
4.81
3.89
3.87
3.84
3.63
3.62
3.48
3.47
3.25
2.96
2.94
2.93
2.90
-0.00
Cl⁻
5j
12.49
0.95
1.91
1.93
2.02
2.02
6.00
2.10
f1 (ppm)

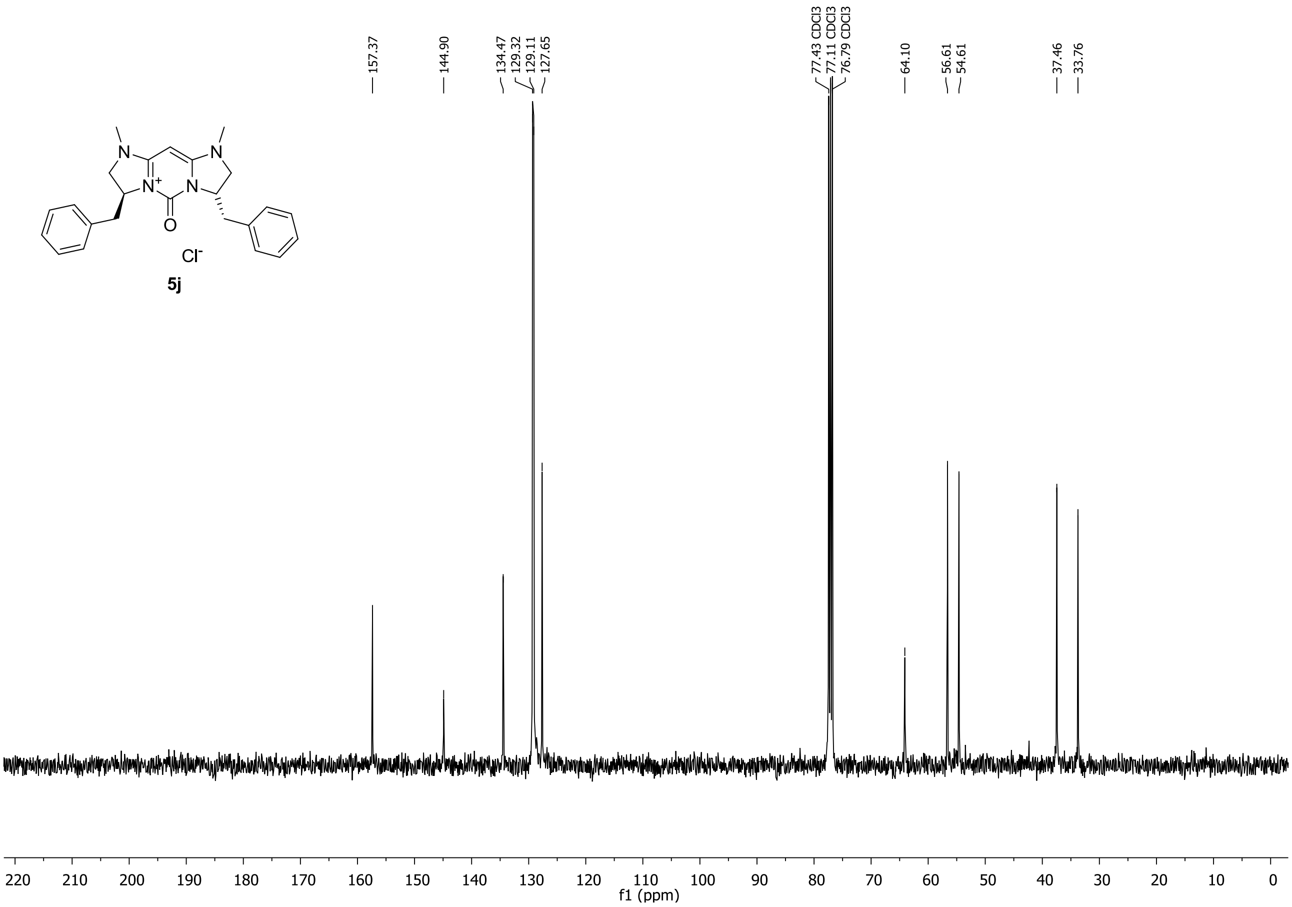
157.37
144.90
134.47
129.32
129.11
127.65
77.43 CDCl3
77.11 CDCl3
76.79 CDCl3
64.10
56.61
54.61
37.46
33.76
Cl⁻
5j
f1 (ppm)

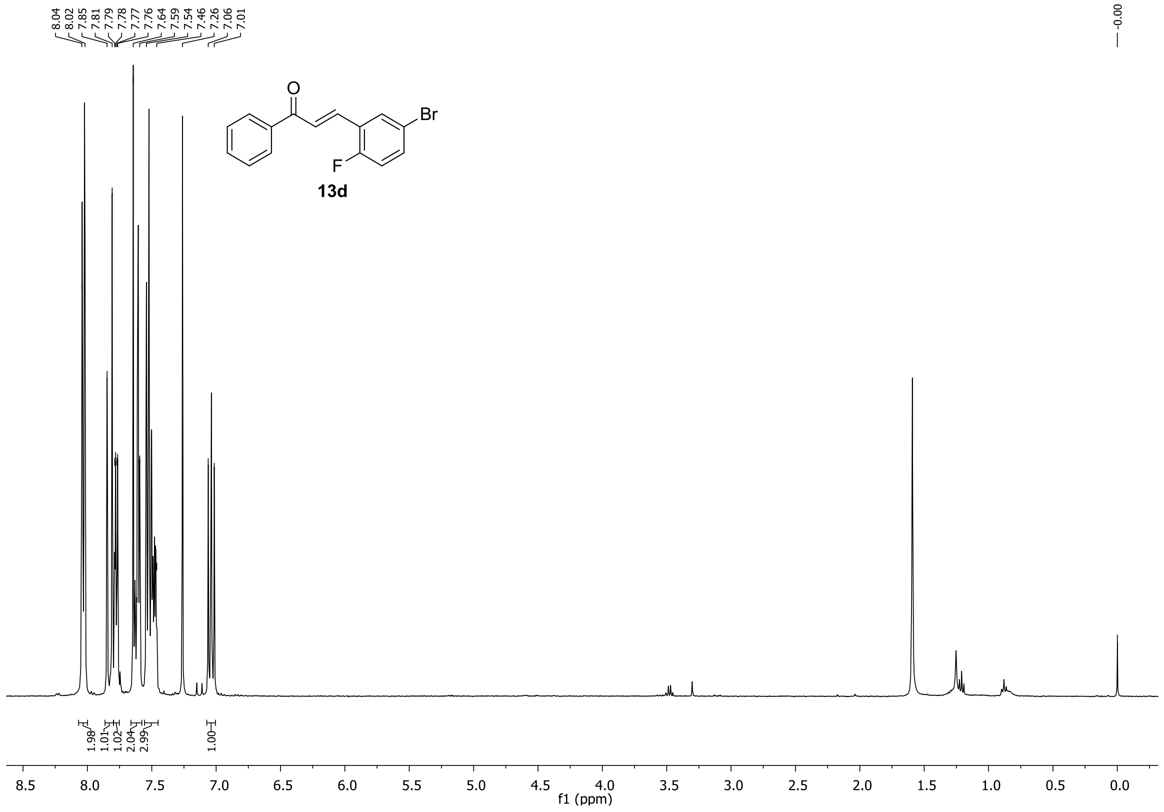
8.04 8.02 7.85 7.81 7.79 7.78 7.77 7.76 7.64 7.59 7.54 7.46 7.26 7.06 7.01
-0.00
Br
F
O
13d
1.98 1.01 1.02 2.04 2.99 1.00
f1 (ppm)

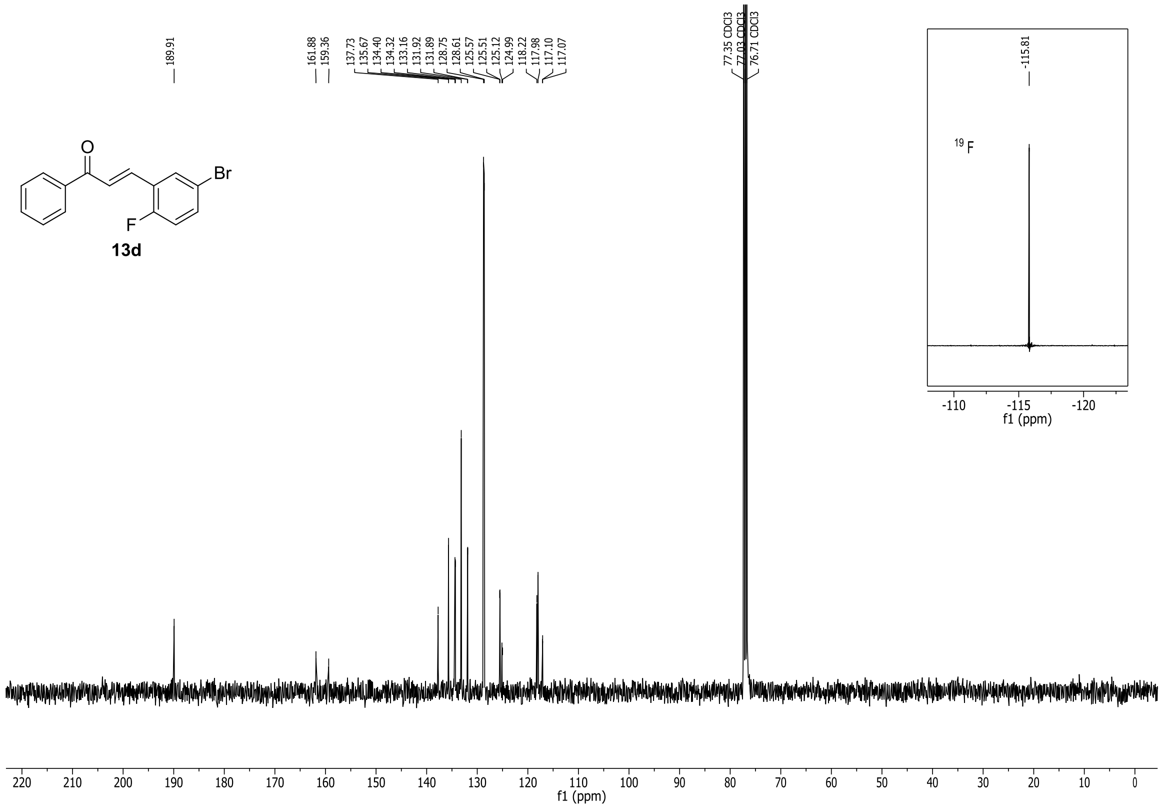
189.91
161.88 159.36
137.73 135.67 134.40 134.32 133.16 131.92 131.89 128.75 128.61 125.57 125.51 125.12 124.99 118.22 117.98 117.10 117.07
77.35 CDCl3 77.03 CDCl3 76.71 CDCl3
-115.81
19 F
Br
F
O
13d
f1 (ppm)

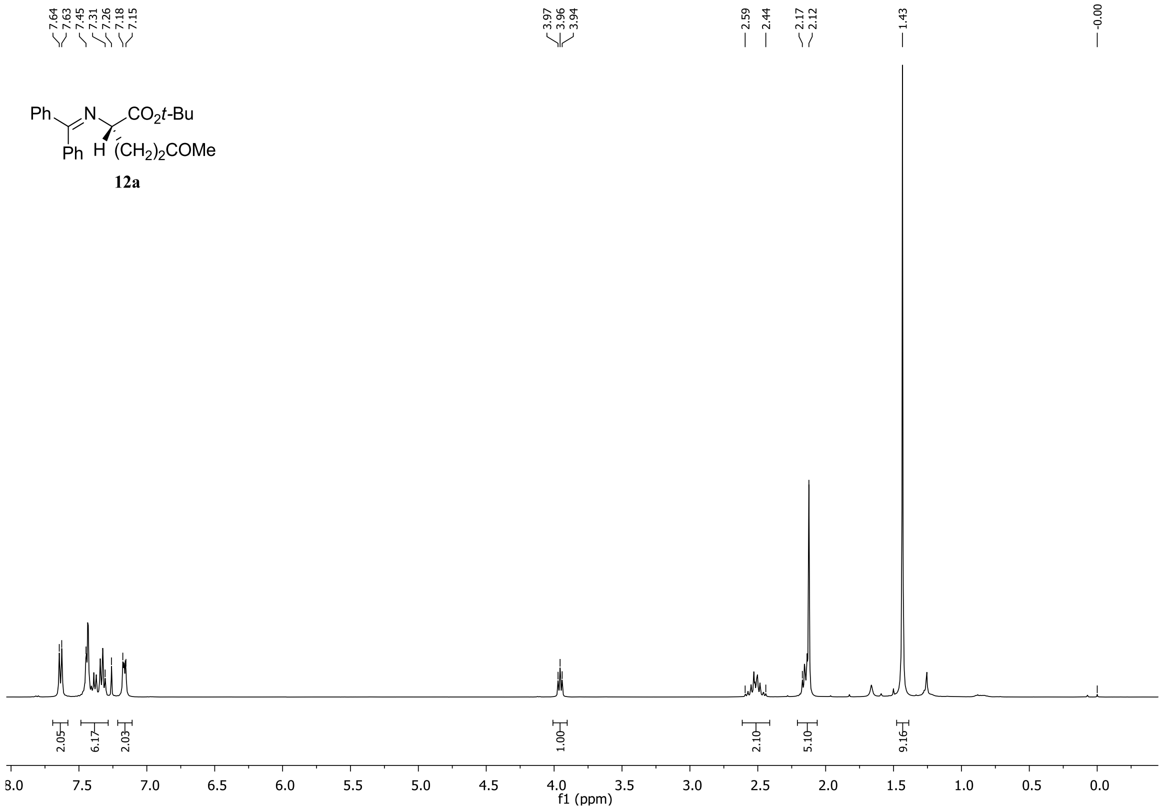
7.64
7.63
7.45
7.31
7.26
7.18
7.15
3.97
3.96
3.94
2.59
2.44
2.17
2.12
1.43
-0.00
Ph
N
CO2t-Bu
Ph
H
(CH2)2COMe
12a
2.05
6.17
2.03
1.00
2.10
5.10
9.16
8.0 7.5 7.0 6.5 6.0 5.5 5.0 4.5 4.0 3.5 3.0 2.5 2.0 1.5 1.0 0.5 0.0
f1 (ppm)

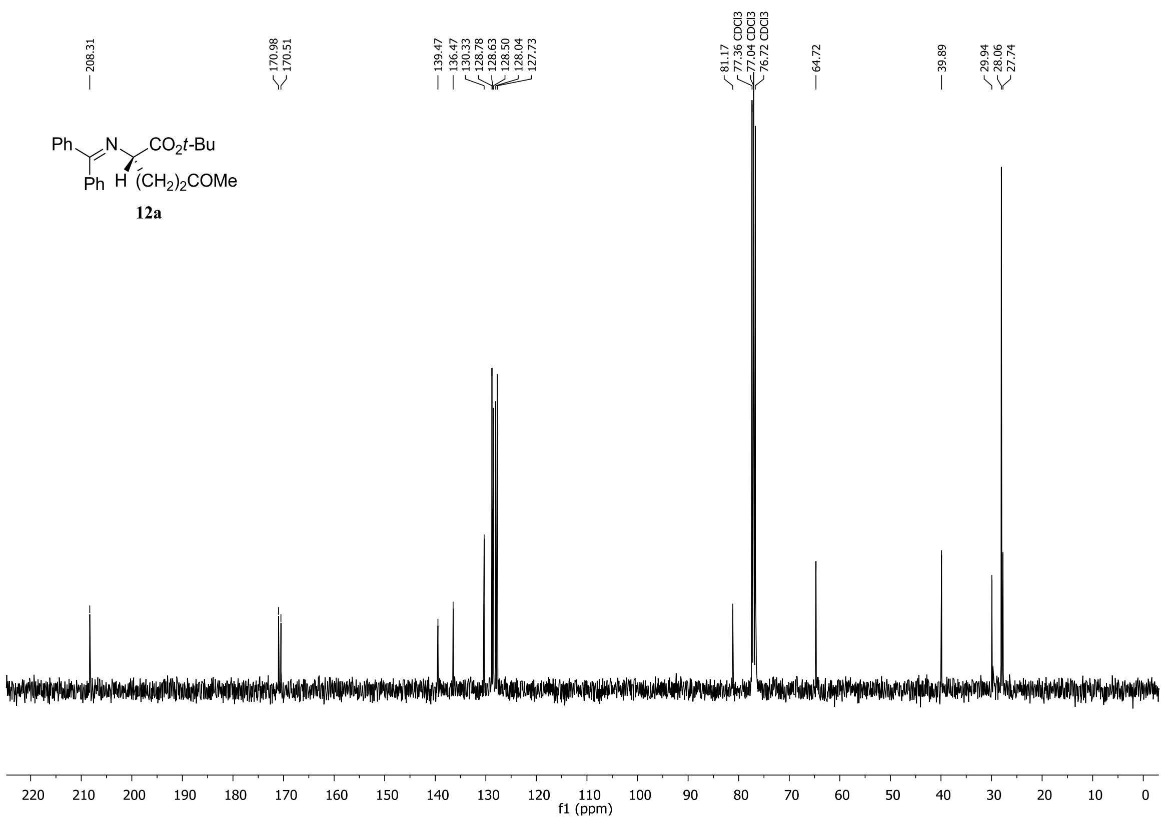
208.31
170.98
170.51
139.47
136.47
130.33
128.78
128.63
128.50
128.04
127.73
81.17
77.36 CDCl3
77.04 CDCl3
76.72 CDCl3
64.72
39.89
29.94
28.06
27.74
Ph
N
CO2t-Bu
Ph
H
(CH2)2COMe
12a
220 210 200 190 180 170 160 150 140 130 120 110 100 90 80 70 60 50 40 30 20 10 0
f1 (ppm)

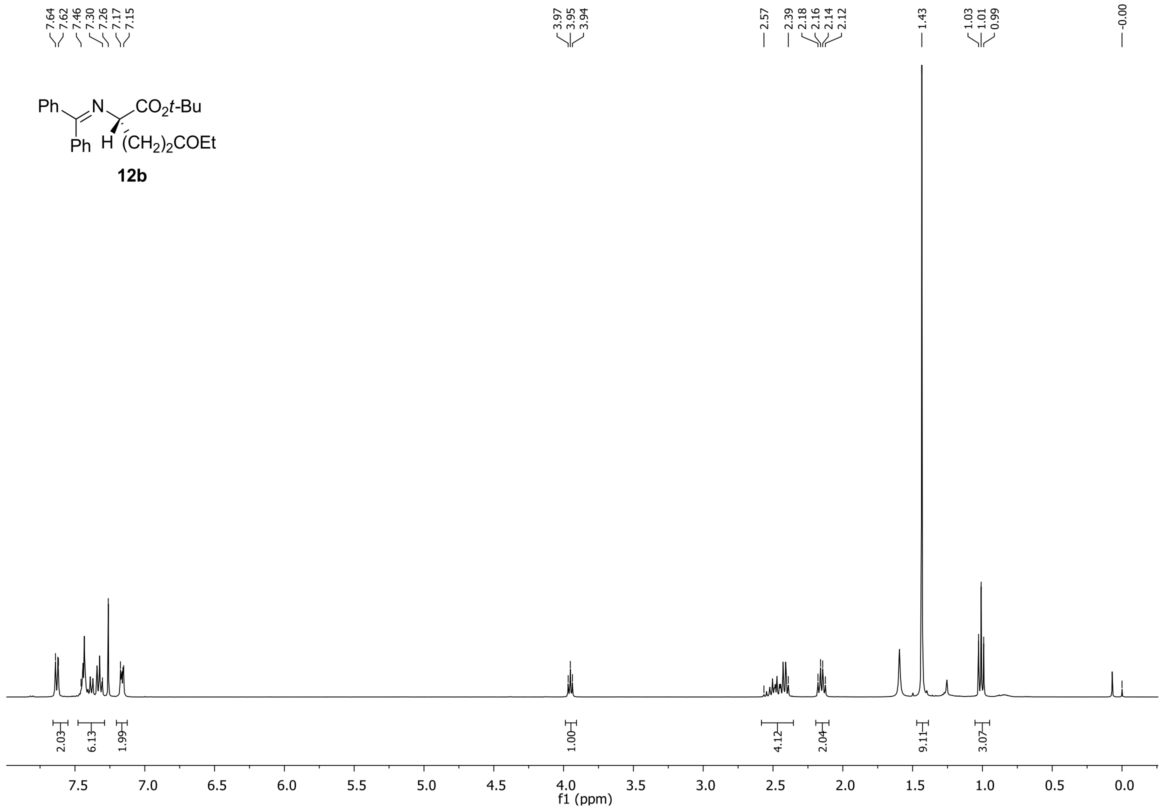
7.64
7.62
7.46
7.30
7.26
7.17
7.15
3.97
3.95
3.94
2.57
2.39
2.18
2.16
2.14
2.12
1.43
1.03
1.01
0.99
-0.00
Ph
N
CO2t-Bu
Ph
H
(CH2)2COEt
12b
2.03
6.13
1.99
1.00
4.12
2.04
9.11
3.07
f1 (ppm)

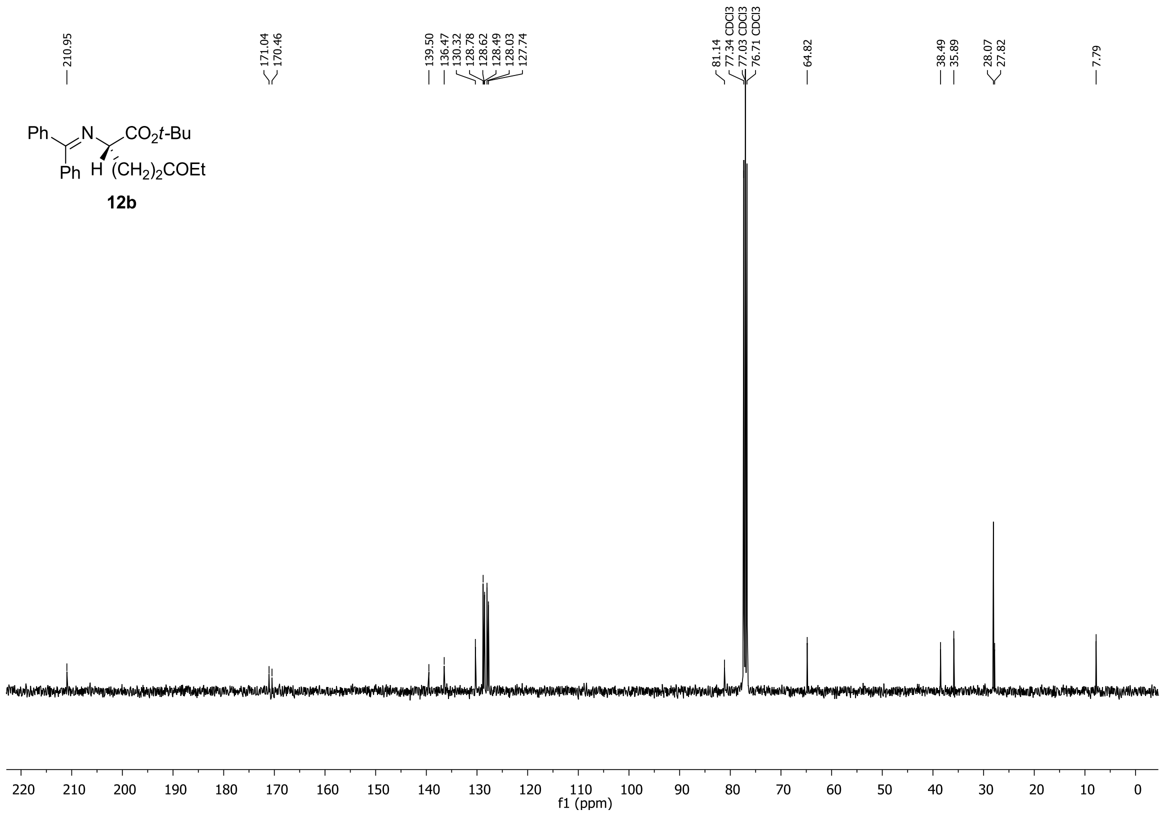
210.95
171.04
170.46
139.50
136.47
130.32
128.78
128.62
128.49
128.03
127.74
81.14
77.34 CDCl3
77.03 CDCl3
76.71 CDCl3
64.82
38.49
35.89
28.07
27.82
7.79
Ph
N
CO2t-Bu
Ph
H
(CH2)2COEt
12b
f1 (ppm)

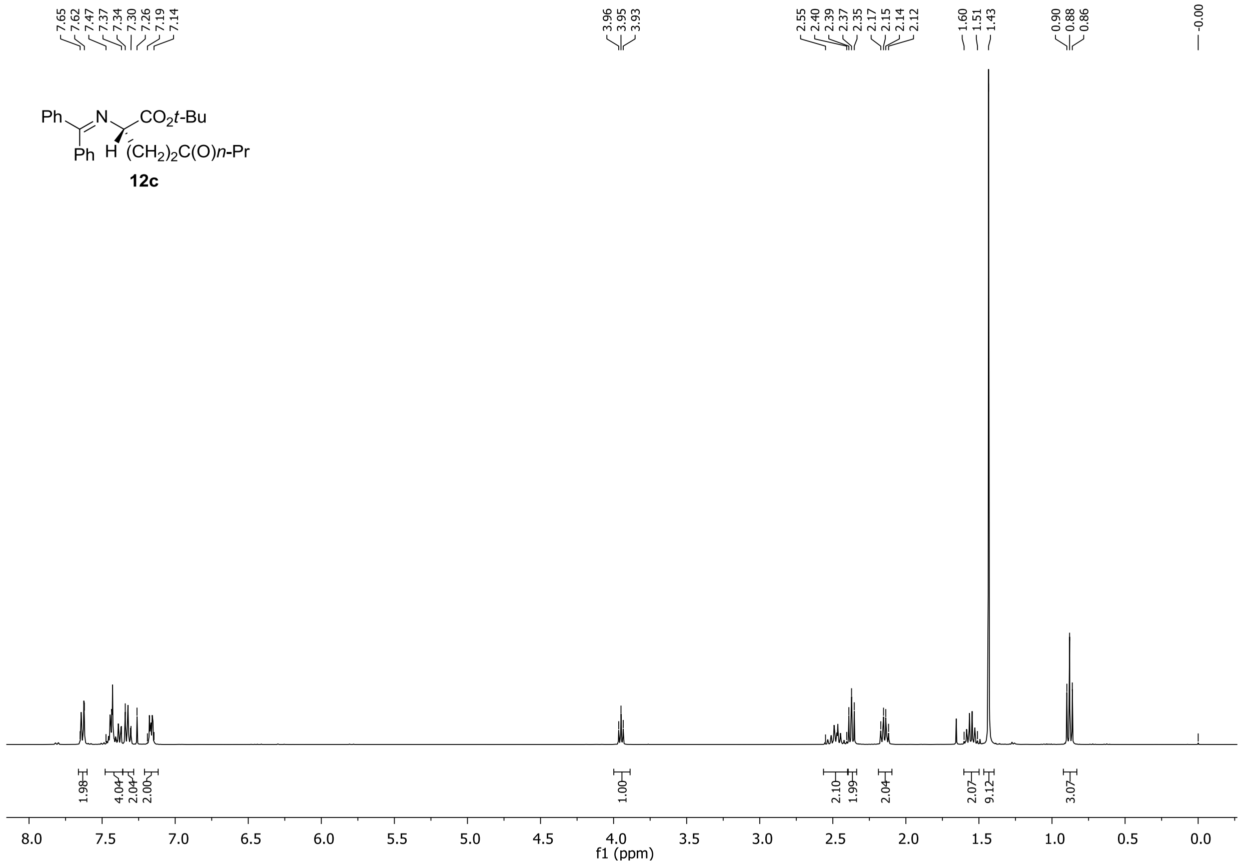
7.65
7.62
7.47
7.37
7.34
7.30
7.26
7.19
7.14
3.96
3.95
3.93
2.55
2.40
2.39
2.37
2.35
2.17
2.15
2.14
2.12
1.60
1.51
1.43
0.90
0.88
0.86
-0.00
Ph
N
CO2t-Bu
Ph
H
(CH2)2C(O)n-Pr
12c
1.98
4.04
2.04
2.00
1.00
2.10
1.99
2.04
2.07
9.12
3.07
f1 (ppm)

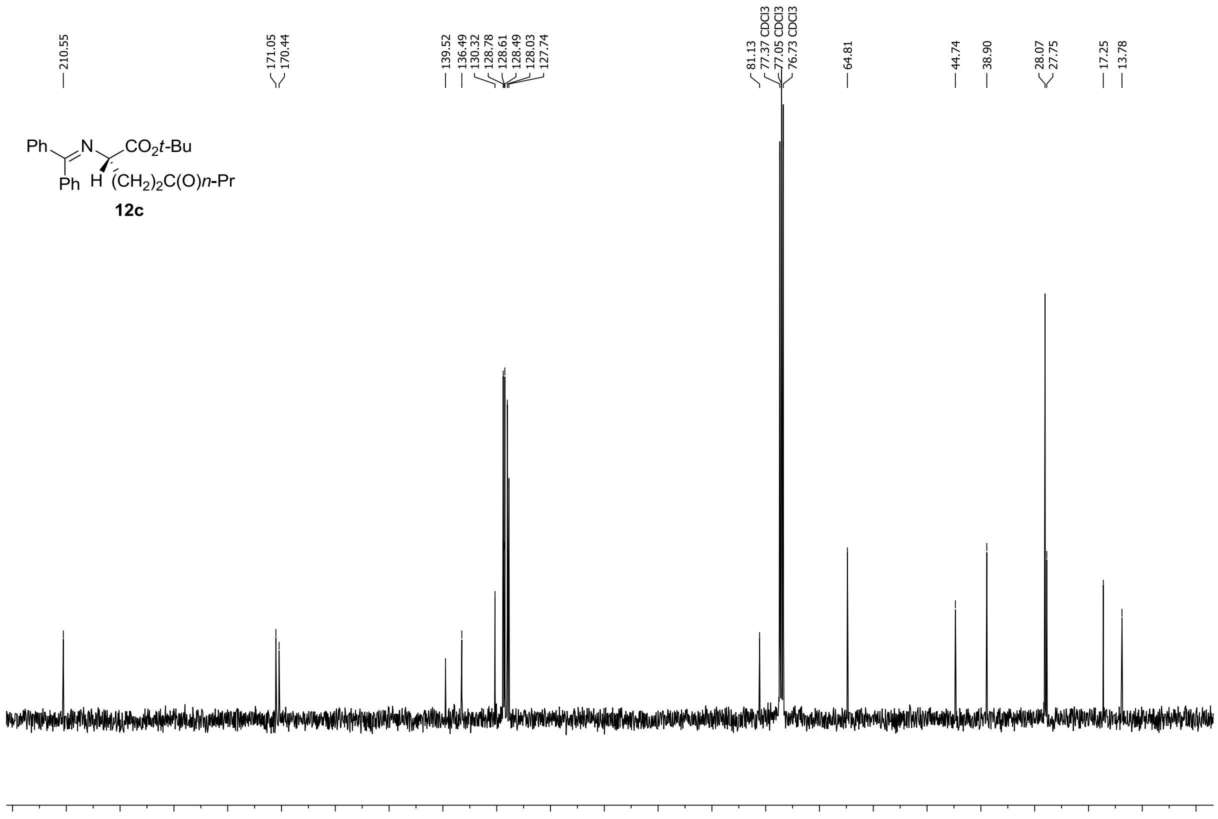
210.55
171.05
170.44
139.52
136.49
130.32
128.78
128.61
128.49
128.03
127.74
81.13
77.37 CDCl3
77.05 CDCl3
76.73 CDCl3
64.81
44.74
38.90
28.07
27.75
17.25
13.78
Ph
N
CO2t-Bu
Ph
H
(CH2)2C(O)n-Pr
12c
f1 (ppm)

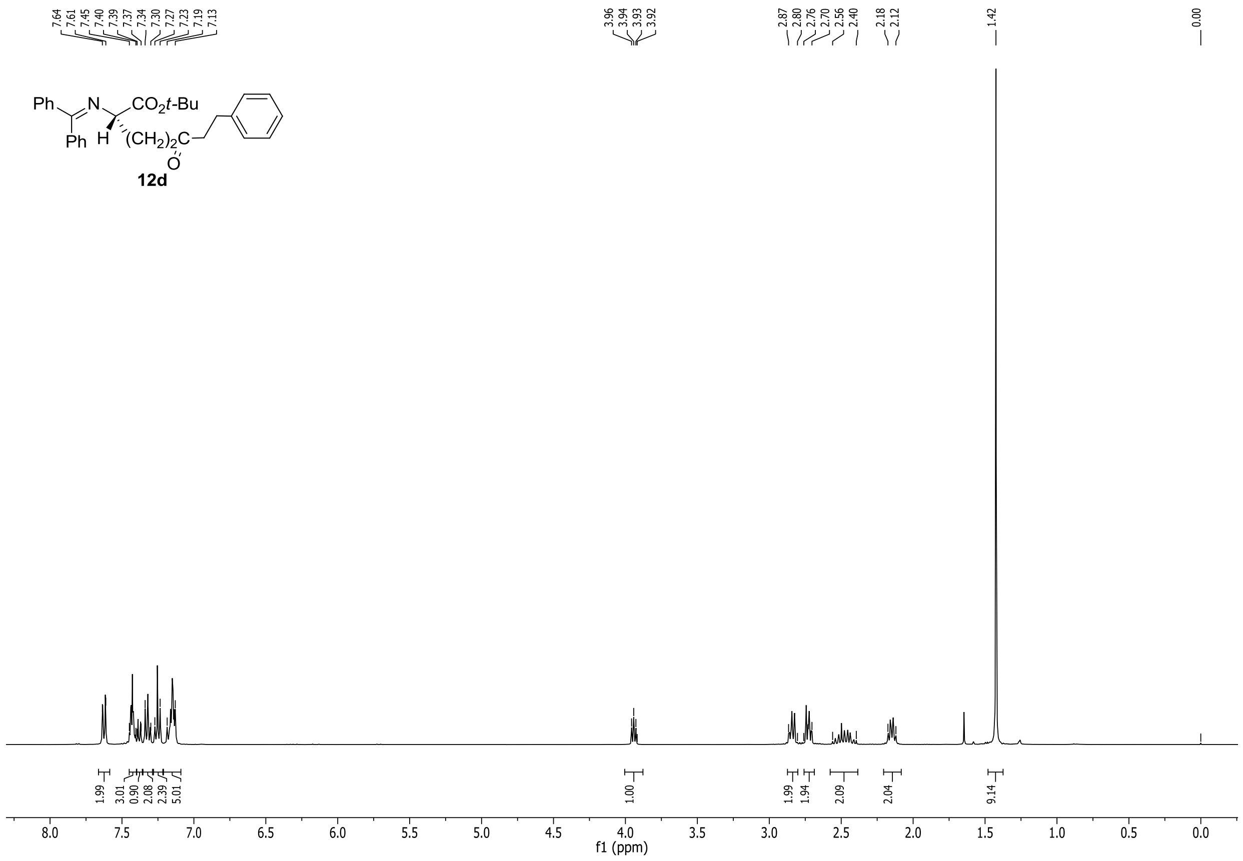
7.64
7.61
7.45
7.40
7.39
7.37
7.34
7.30
7.27
7.23
7.19
7.13
3.96
3.94
3.93
3.92
2.87
2.80
2.76
2.70
2.56
2.40
2.18
2.12
1.42
0.00
Ph
N
CO2t-Bu
Ph
H
(CH2)2C
O
12d
1.99
3.01
0.90
2.08
2.39
5.01
1.00
1.99
1.94
2.09
2.04
9.14
f1 (ppm)

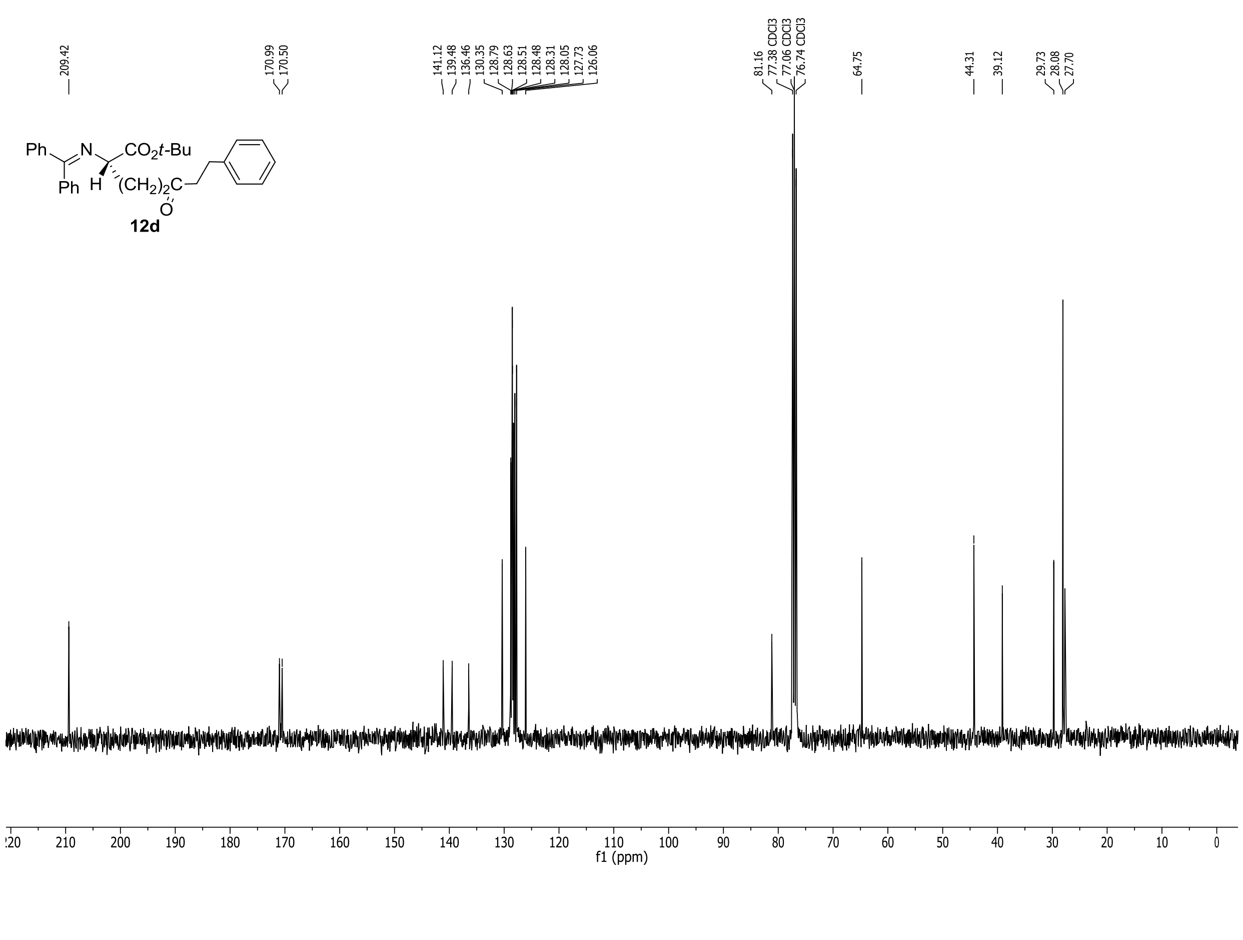
209.42
170.99
170.50
141.12
139.48
136.46
130.35
128.79
128.63
128.51
128.48
128.31
128.05
127.73
126.06
81.16
77.38 CDCl3
77.06 CDCl3
76.74 CDCl3
64.75
44.31
39.12
29.73
28.08
27.70
Ph
N
CO2t-Bu
Ph
H
(CH2)2C
O
12d
f1 (ppm)

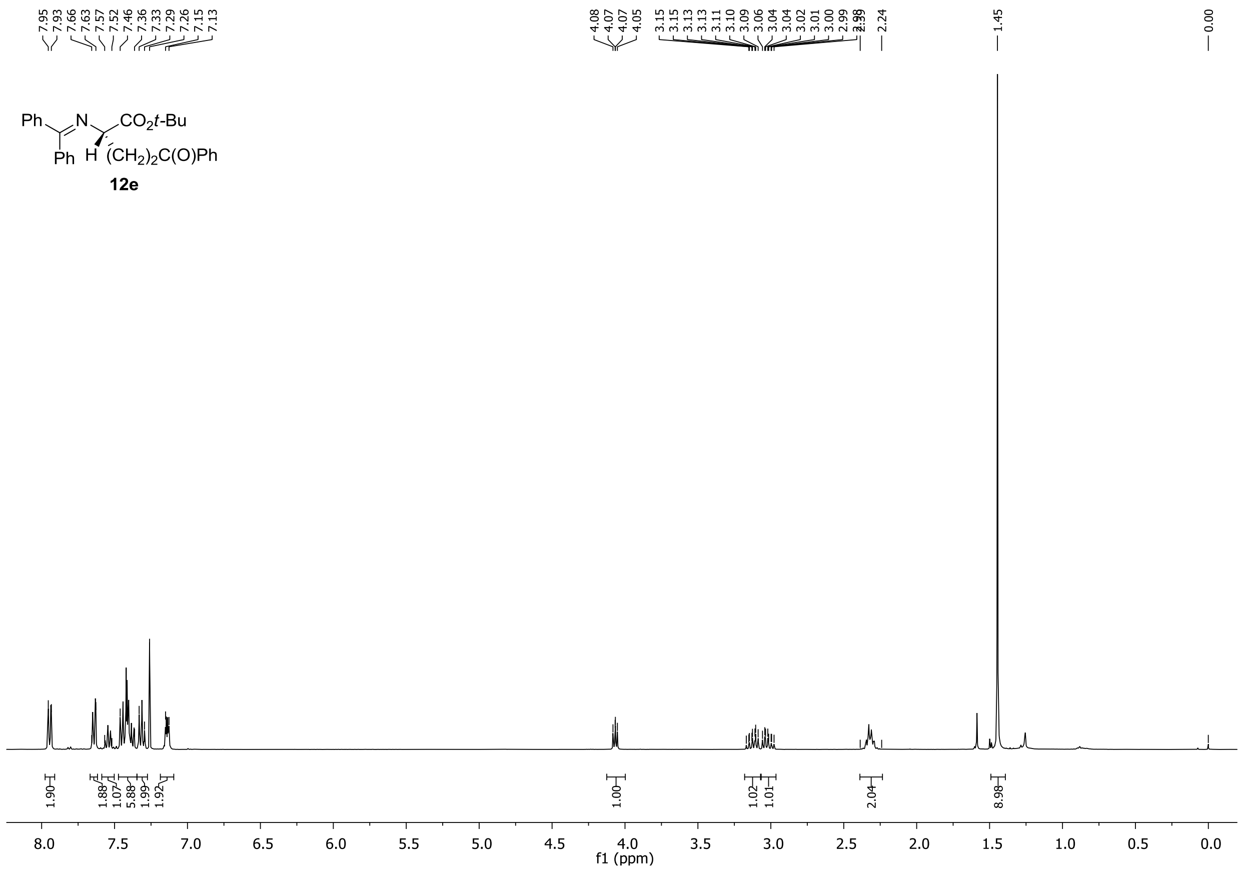

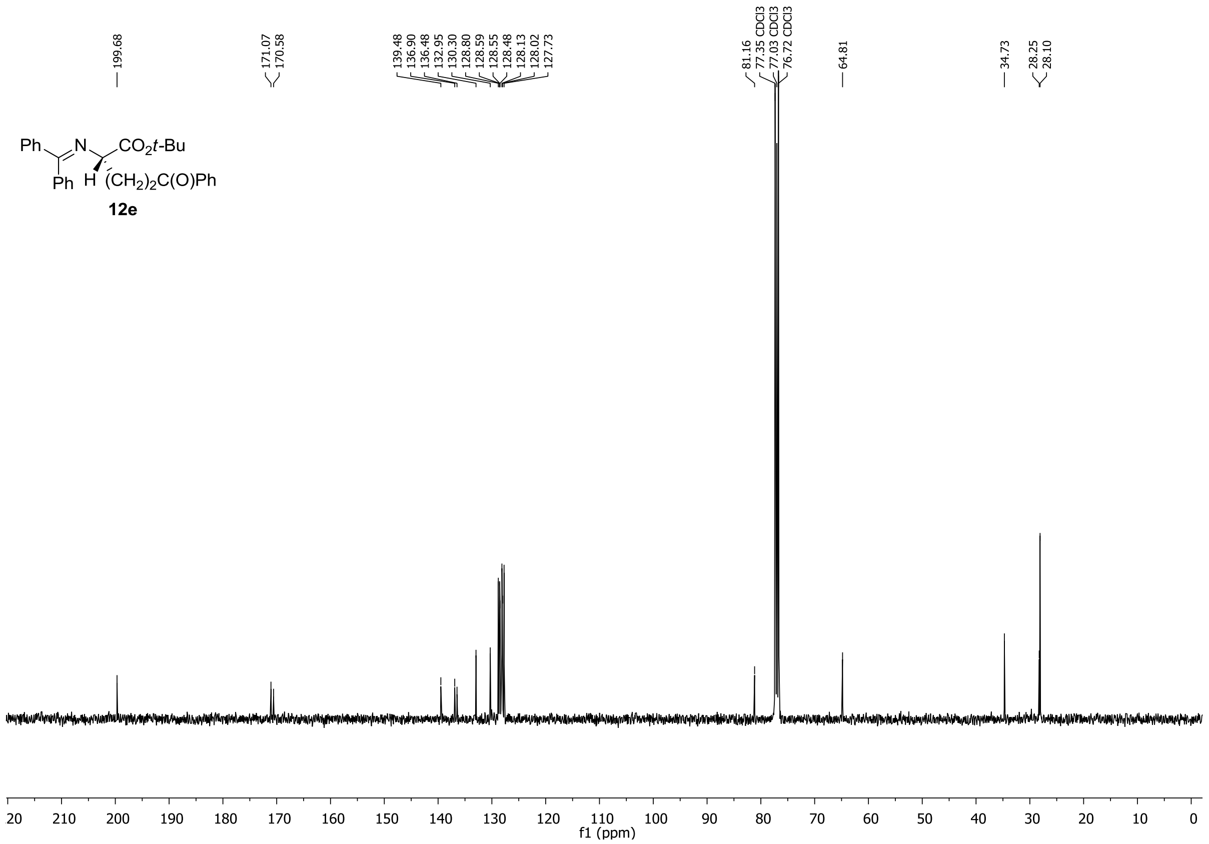

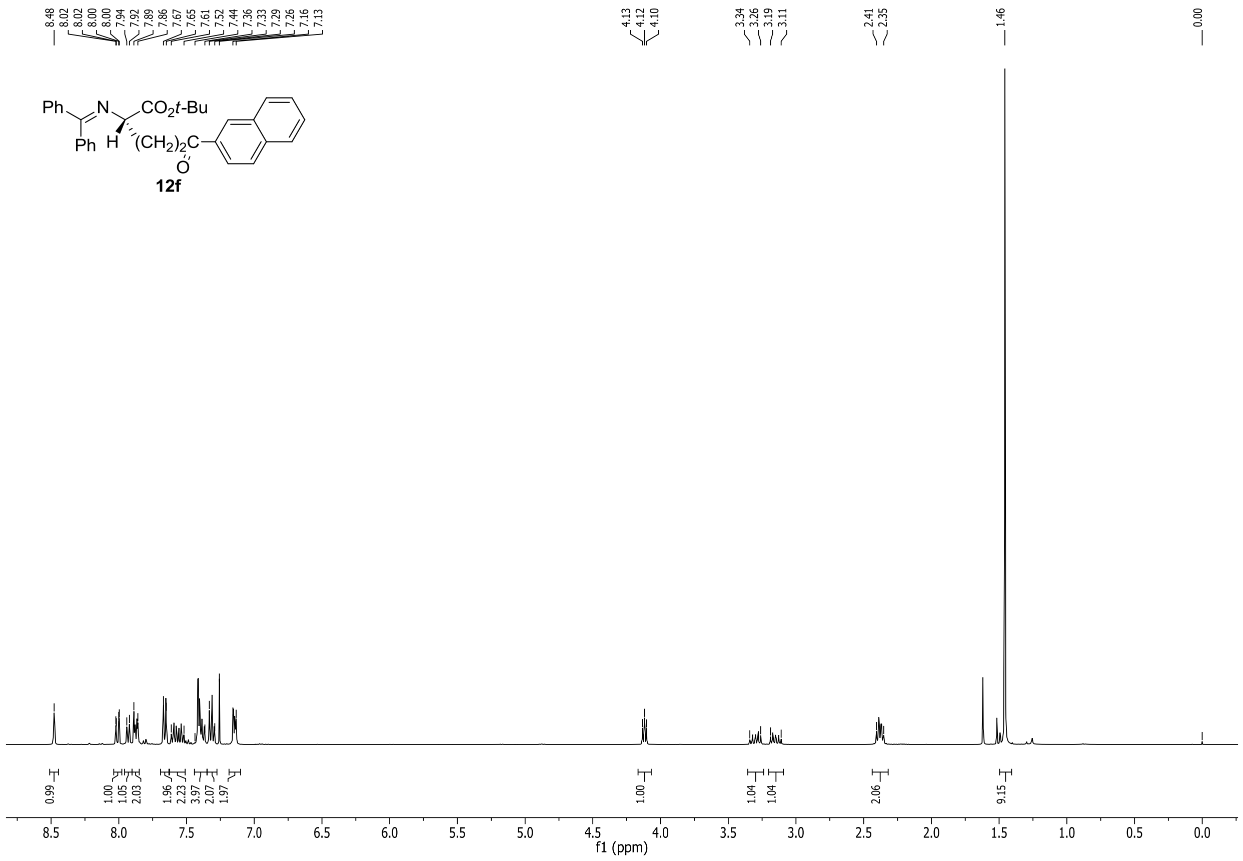
8.48 8.02 8.02 8.00 8.00 7.94 7.92 7.89 7.86 7.67 7.65 7.61 7.52 7.44 7.36 7.33 7.29 7.26 7.16 7.13
4.13 4.12 4.10
3.34 3.26 3.19 3.11
2.41 2.35
1.46
0.00
Ph
N
CO2t-Bu
Ph
H
(CH2)2C
O
12f
0.99
1.00 1.05 2.03
1.96 2.23 3.97 2.07 1.97
1.00
1.04 1.04
2.06
9.15
f1 (ppm)

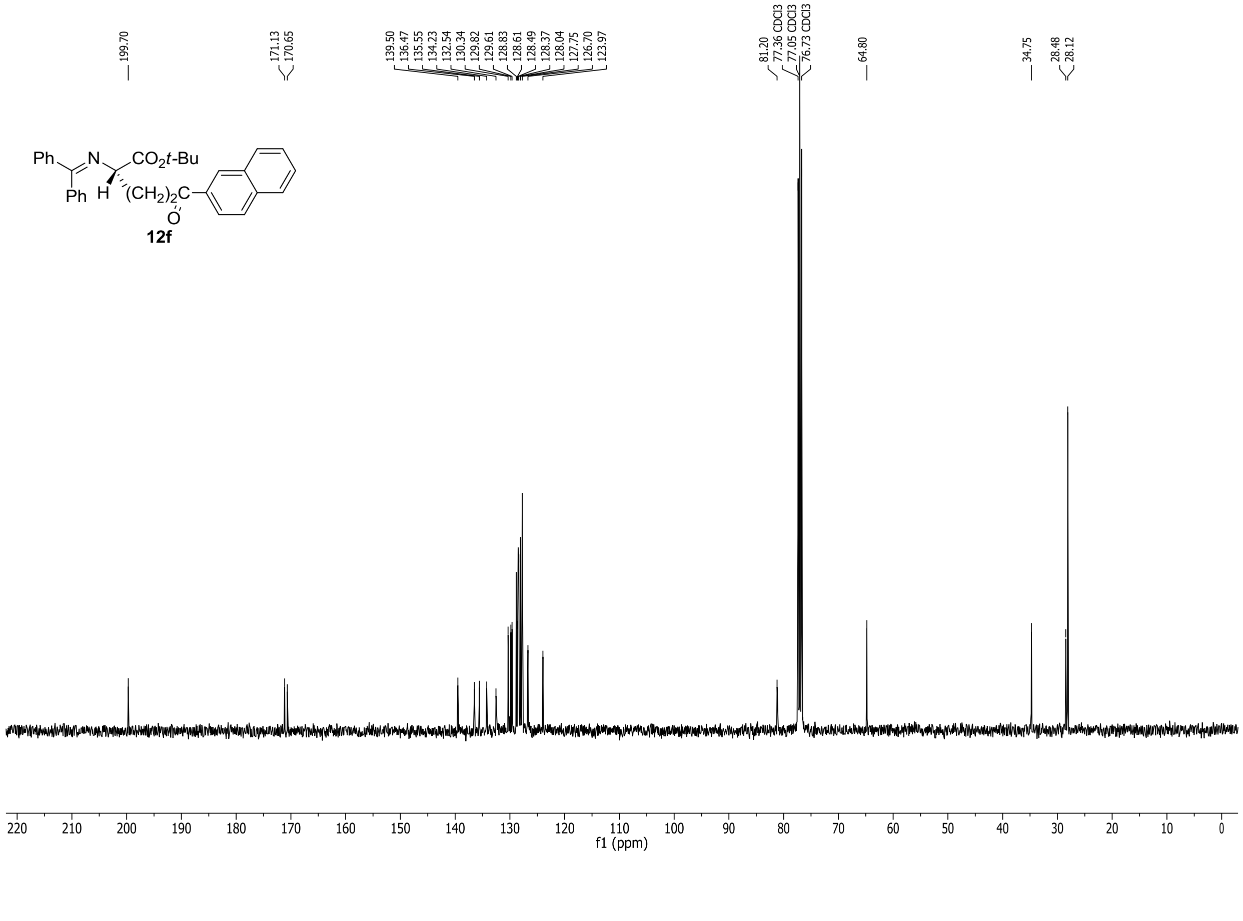
199.70
171.13 170.65
139.50 136.47 135.55 134.23 132.54 130.34 129.82 129.61 128.83 128.61 128.49 128.37 128.04 127.75 126.70 123.97
81.20 77.36 CDCl3 77.05 CDCl3 76.73 CDCl3
64.80
34.75
28.48 28.12
Ph
N
CO2t-Bu
Ph
H
(CH2)2C
O
12f
f1 (ppm)

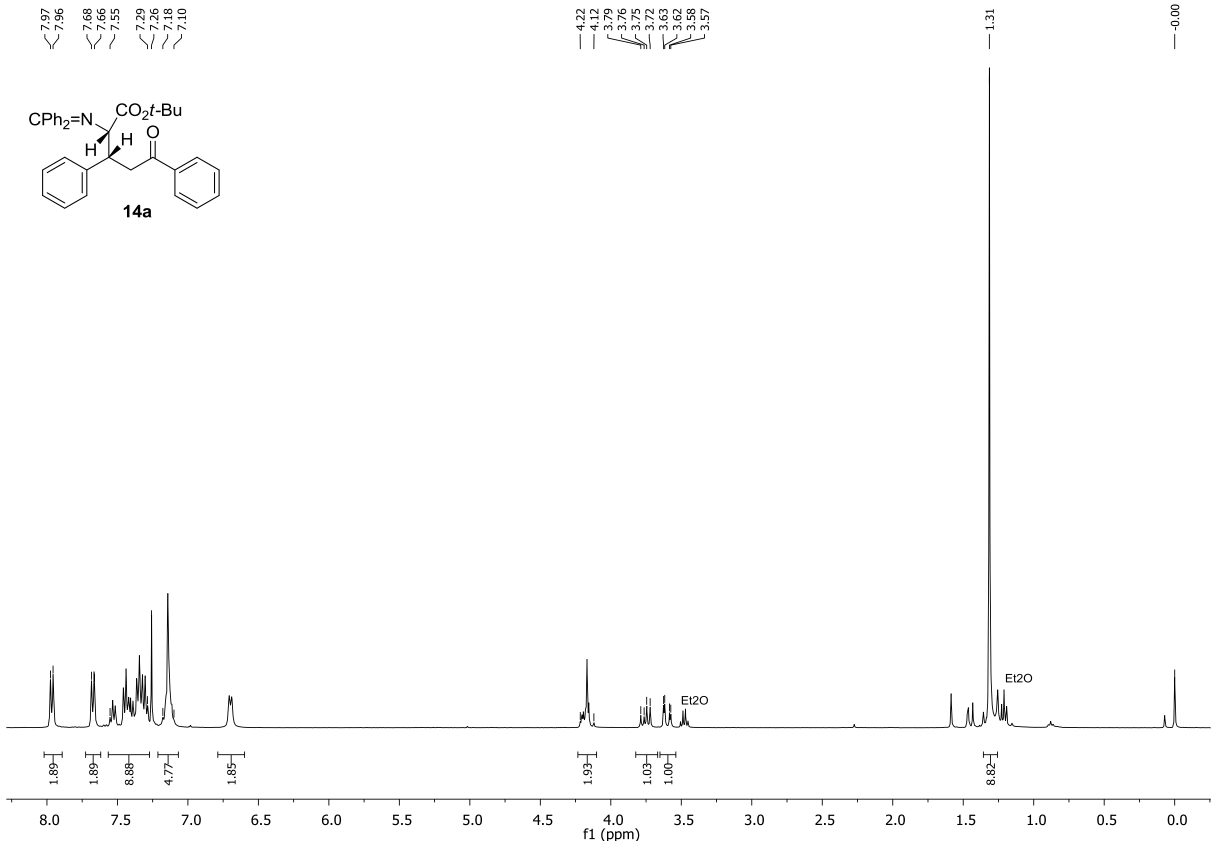
7.97
7.96
7.68
7.66
7.55
7.29
7.26
7.18
7.10
4.22
4.12
3.79
3.76
3.75
3.72
3.63
3.62
3.58
3.57
1.31
-0.00
CPh2=N
CO2t-Bu
14a
Et2O
Et2O
1.89
1.89
8.88
4.77
1.85
1.93
1.03
1.00
8.82
f1 (ppm)

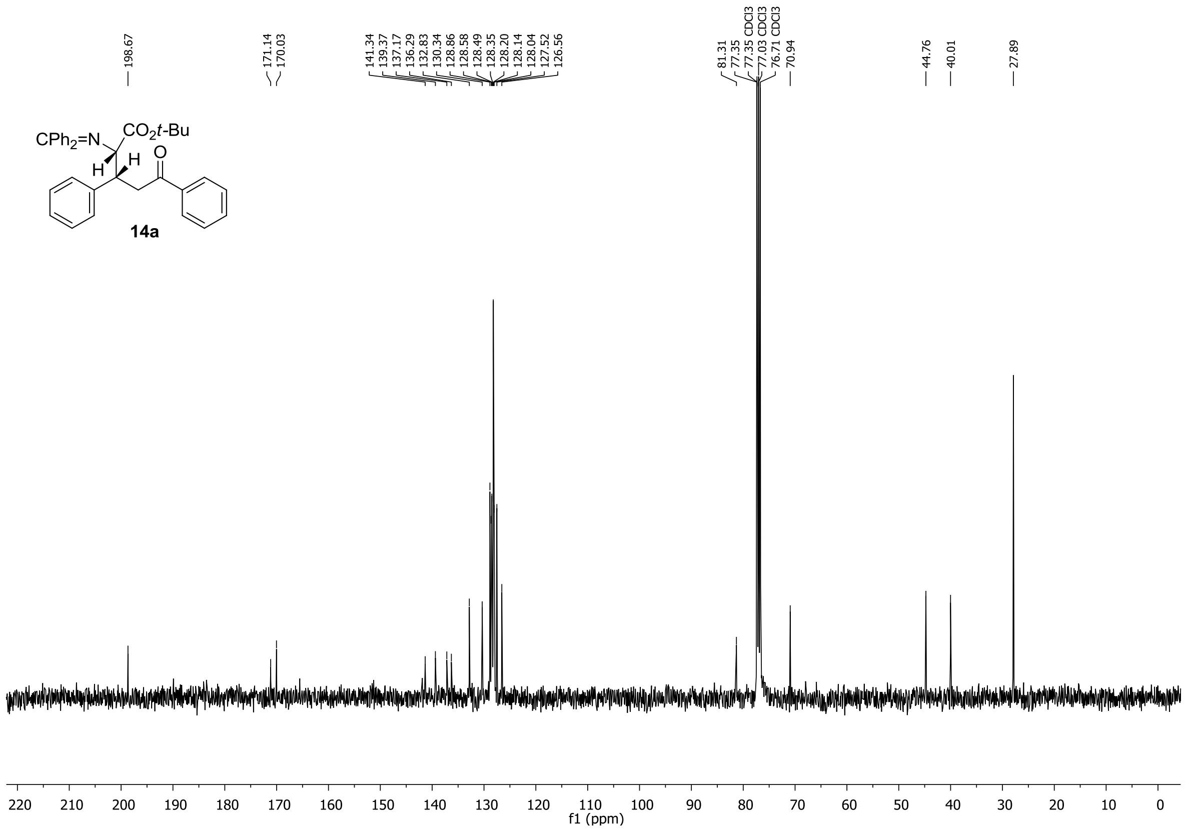
198.67
171.14
170.03
141.34
139.37
137.17
136.29
132.83
130.34
128.86
128.58
128.49
128.35
128.20
128.14
128.04
127.52
126.56
81.31
77.35 CDCl3
77.35 CDCl3
77.03 CDCl3
76.71 CDCl3
70.94
44.76
40.01
27.89
CPh2=N
CO2t-Bu
14a
f1 (ppm)

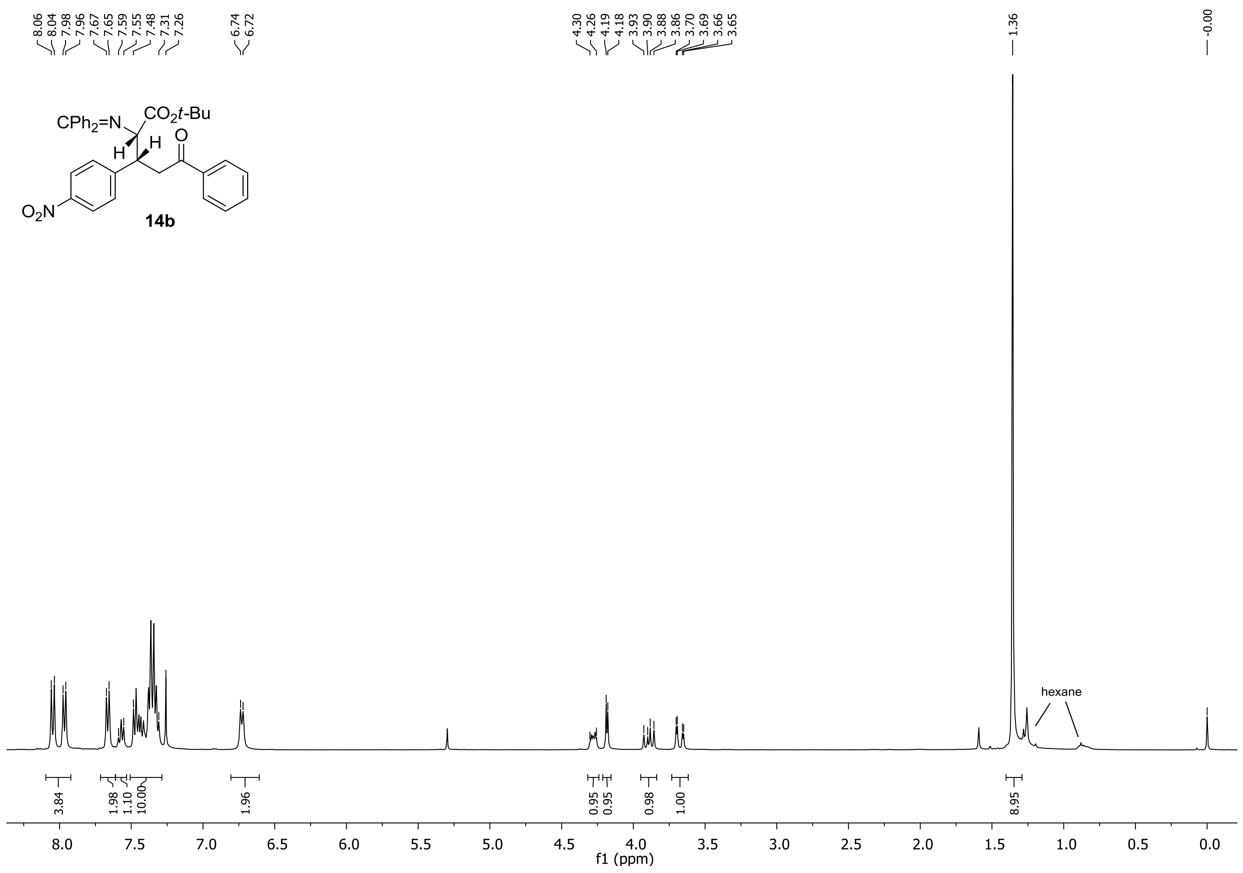
8.06
8.04
7.98
7.96
7.67
7.65
7.59
7.55
7.48
7.31
7.26
6.74
6.72
4.30
4.26
4.19
4.18
3.93
3.90
3.88
3.86
3.70
3.69
3.66
3.65
1.36
-0.00
CPh2=N
CO2t-Bu
H
H
O
O2N
14b
hexane
3.84
1.98
1.10
10.00
1.96
0.95
0.95
0.98
1.00
8.95
8.0 7.5 7.0 6.5 6.0 5.5 5.0 4.5 4.0 3.5 3.0 2.5 2.0 1.5 1.0 0.5 0.0
f1 (ppm)

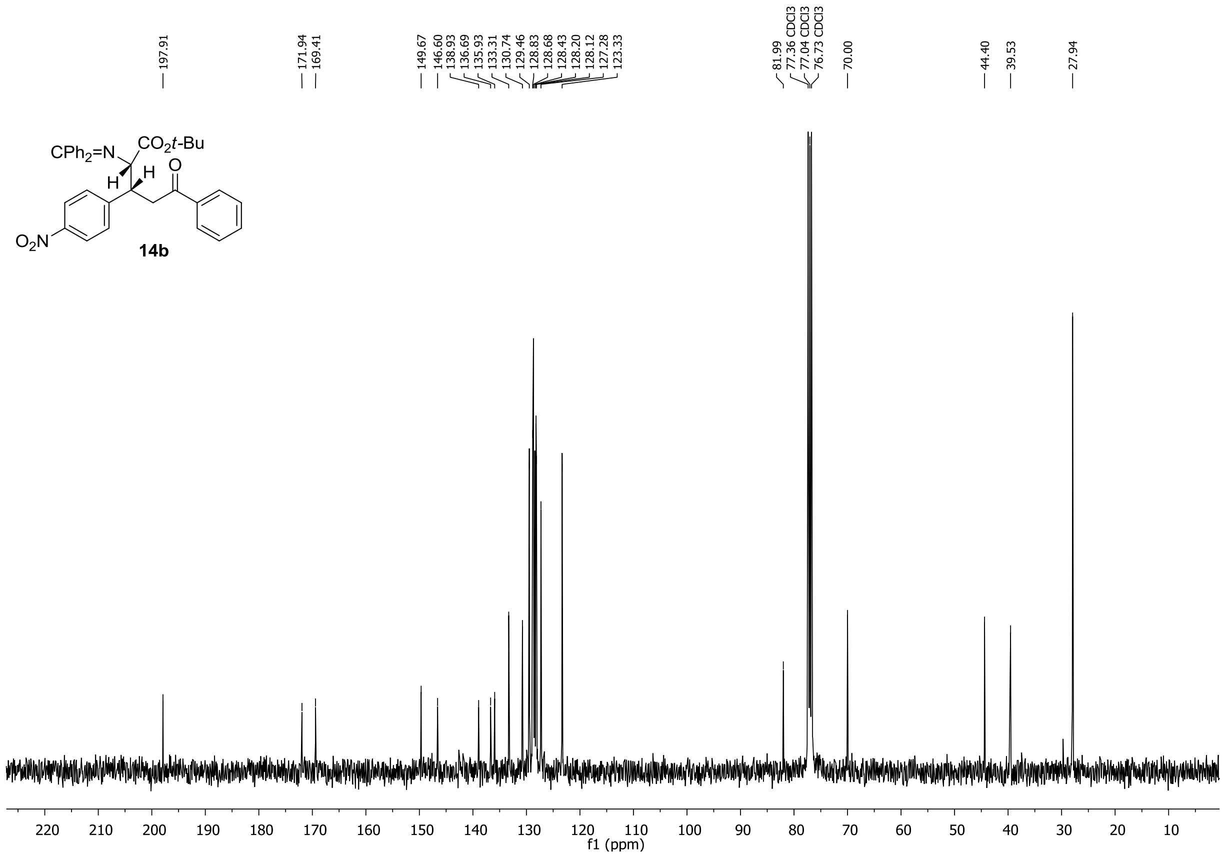
197.91
171.94
169.41
149.67
146.60
138.93
136.69
135.93
133.31
130.74
129.46
128.83
128.68
128.43
128.20
128.12
127.28
123.33
81.99
77.36 CDCl3
77.04 CDCl3
76.73 CDCl3
70.00
44.40
39.53
27.94
CPh2=N
CO2t-Bu
H
H
O
O2N
14b
220 210 200 190 180 170 160 150 140 130 120 110 100 90 80 70 60 50 40 30 20 10
f1 (ppm)

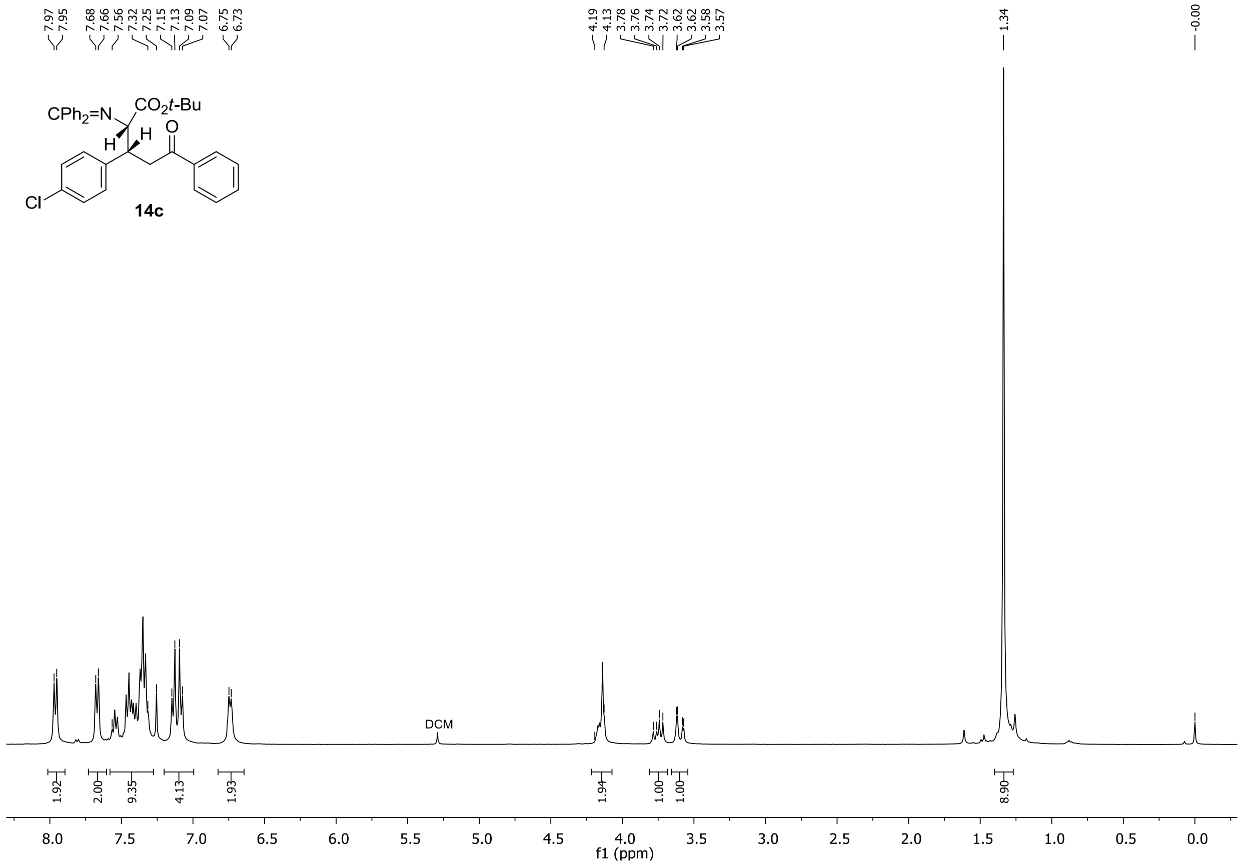
7.97
7.95
7.68
7.66
7.56
7.32
7.25
7.15
7.13
7.09
7.07
6.75
6.73
4.19
4.13
3.78
3.76
3.74
3.72
3.62
3.62
3.58
3.57
1.34
-0.00
CPh2=N
CO2t-Bu
H
H
O
Cl
14c
DCM
1.92
2.00
9.35
4.13
1.93
1.94
1.00
1.00
8.90
f1 (ppm)

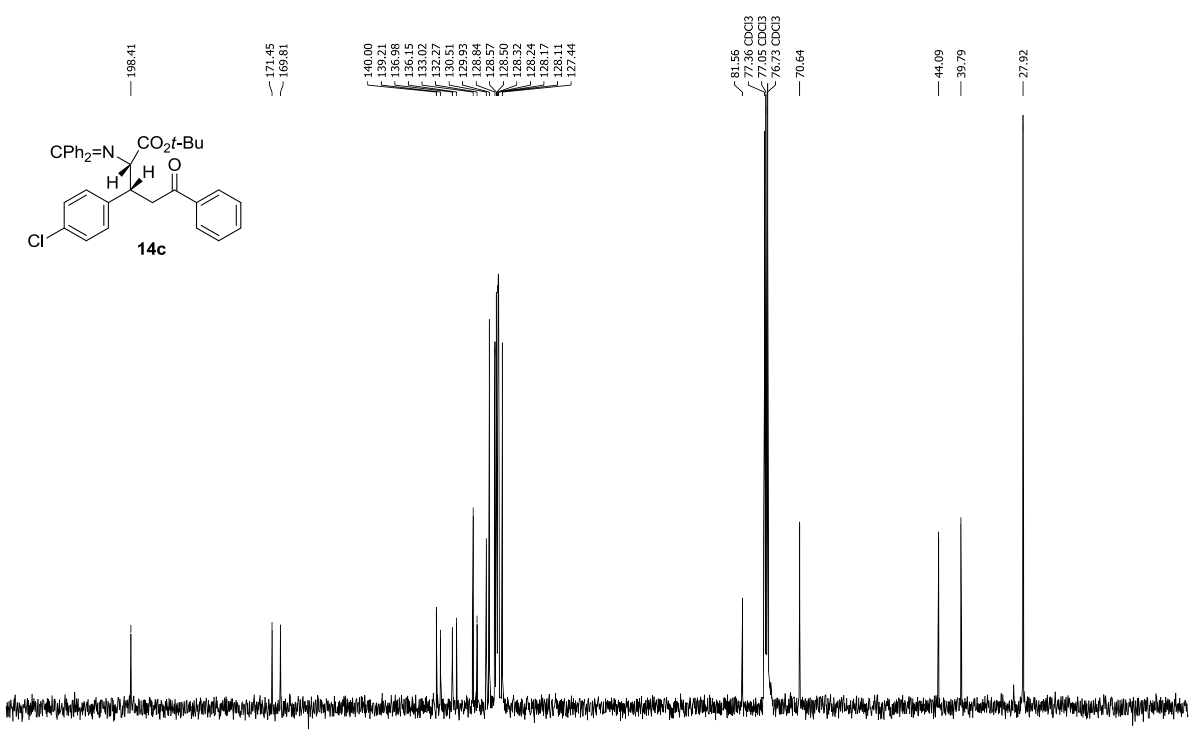
198.41
171.45
169.81
140.00
139.21
136.98
136.15
133.02
132.27
130.51
129.93
128.84
128.57
128.50
128.32
128.24
128.17
128.11
127.44
81.56
77.36 CDCl3
77.05 CDCl3
76.73 CDCl3
70.64
44.09
39.79
27.92
CPh2=N
CO2t-Bu
H
H
O
Cl
14c
f1 (ppm)

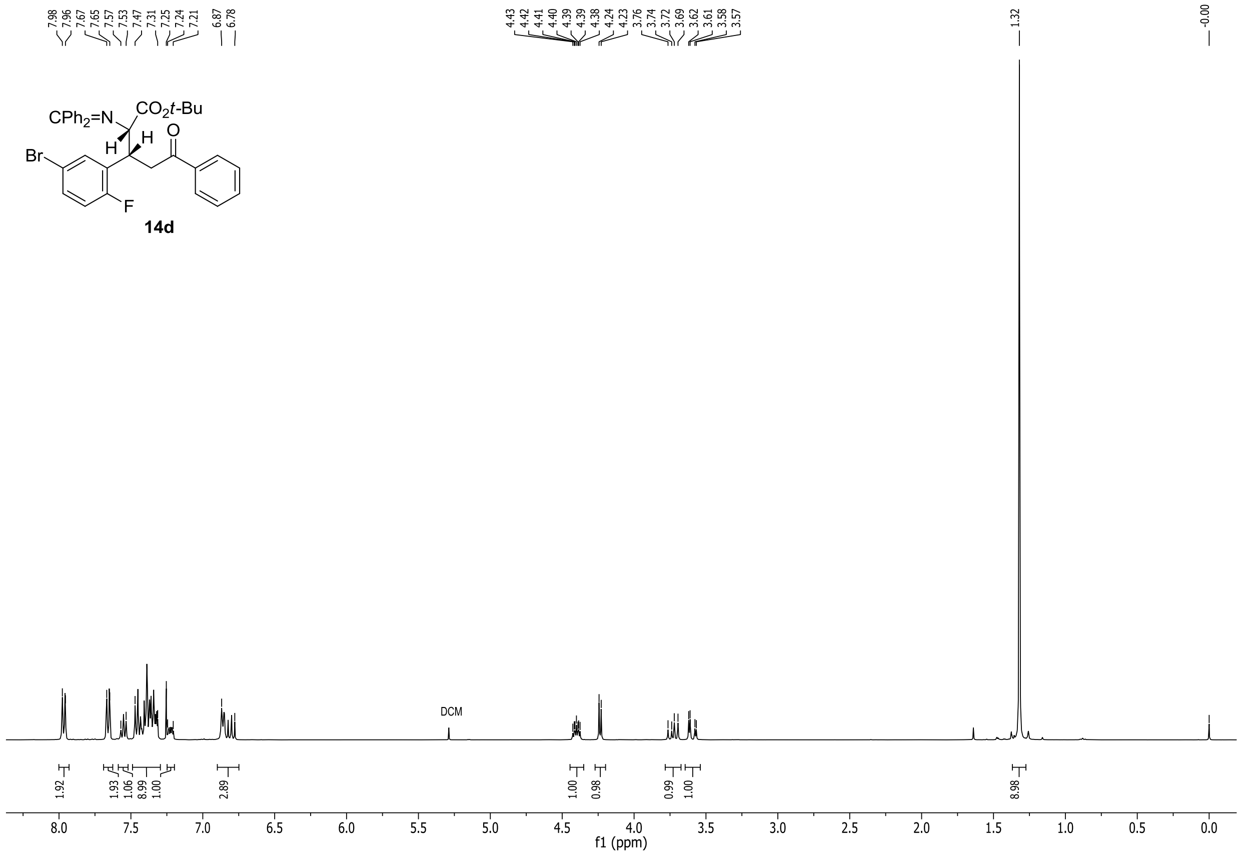
CPh2=N CO2t-Bu
14d
DCM
f1 (ppm)

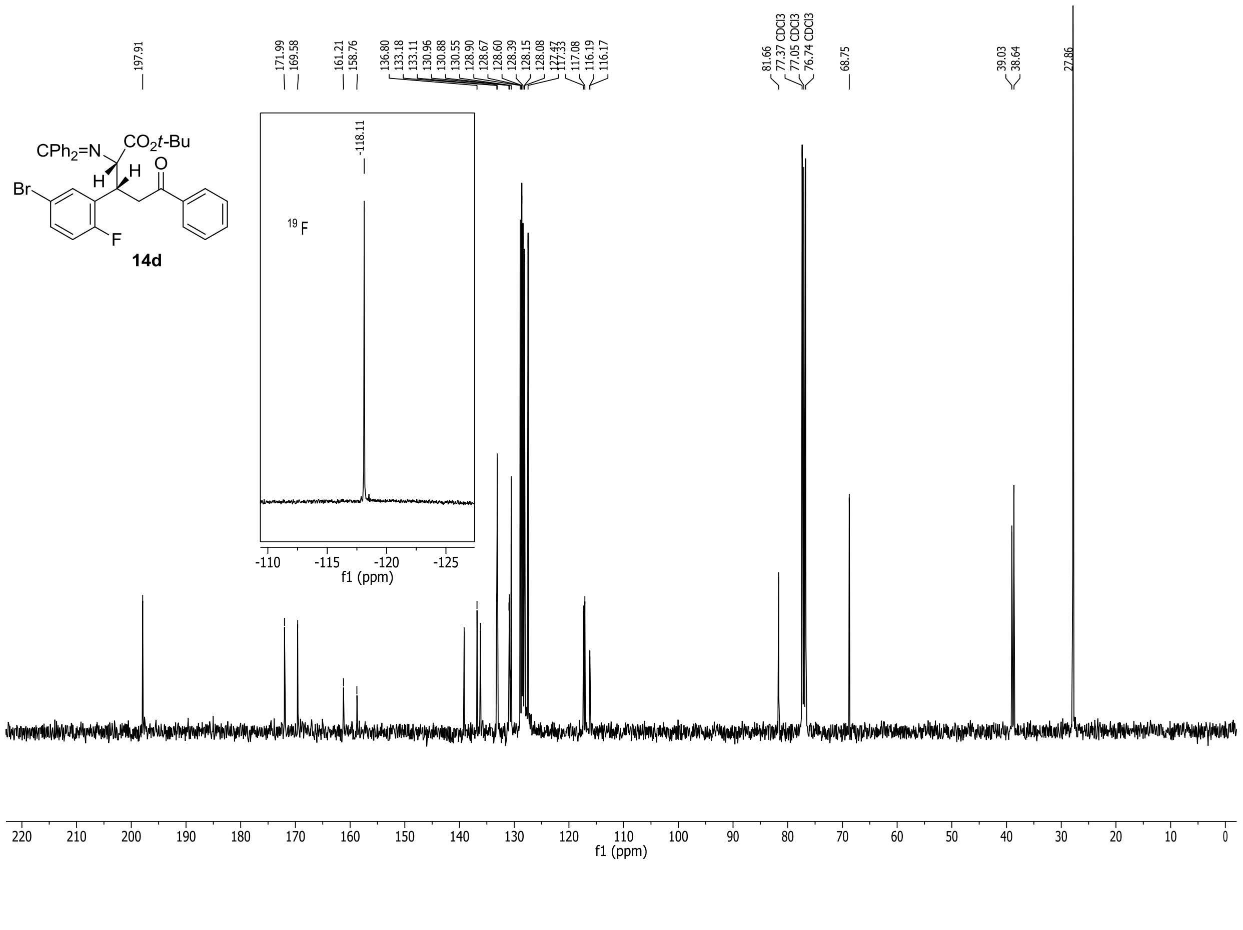
CPh2=N CO2t-Bu
14d
19F
-118.11
f1 (ppm)

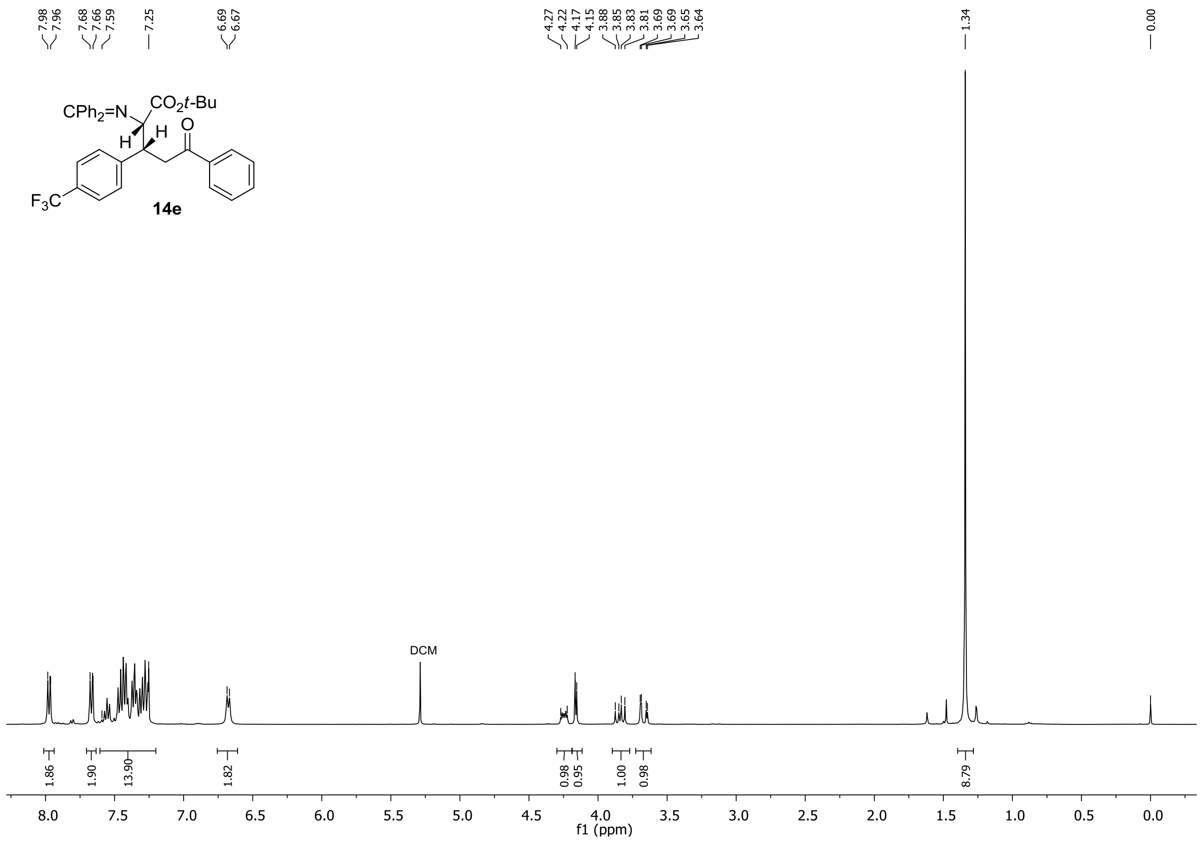

CPh2=N CO2t-Bu
H H O
F3C
14e
DCM
f1 (ppm)

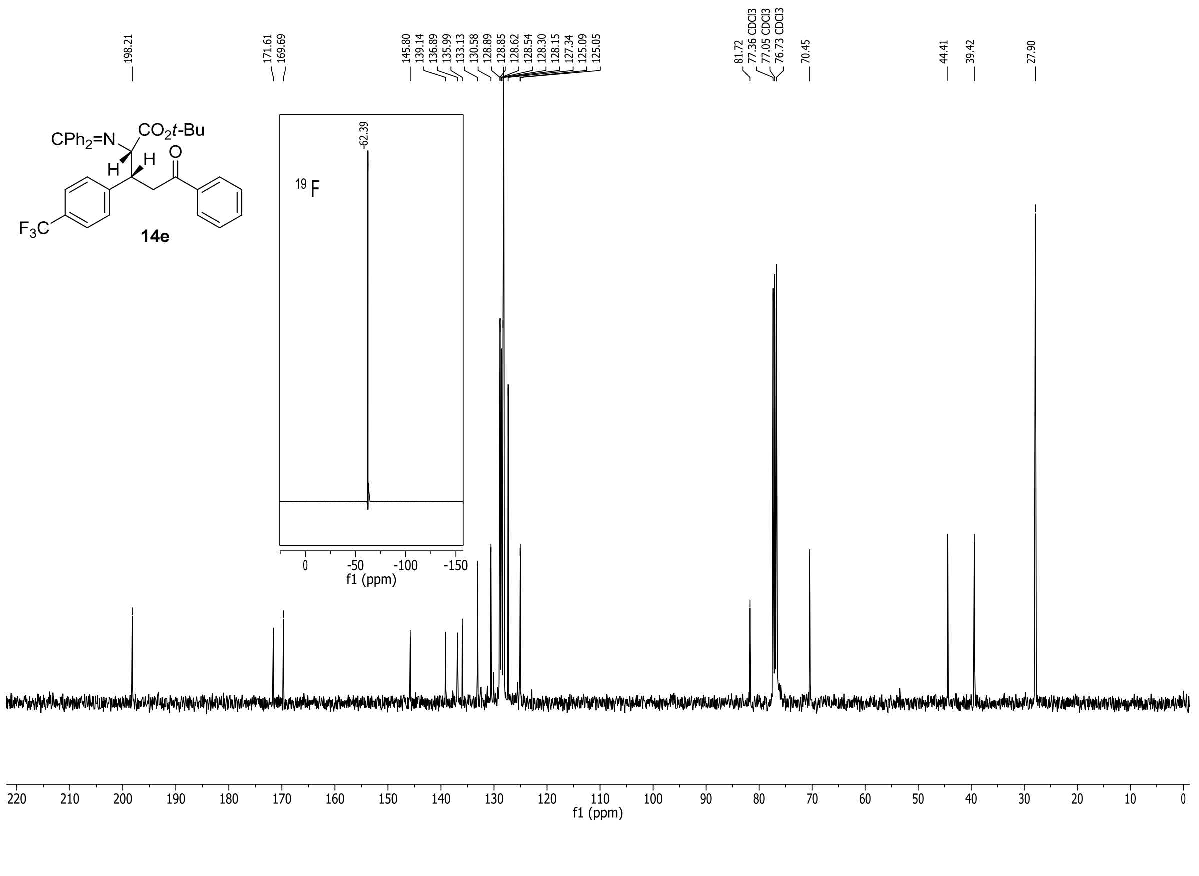

CPh2=N CO2t-Bu
H H O
F3C
14e
19F
-62.39
f1 (ppm)

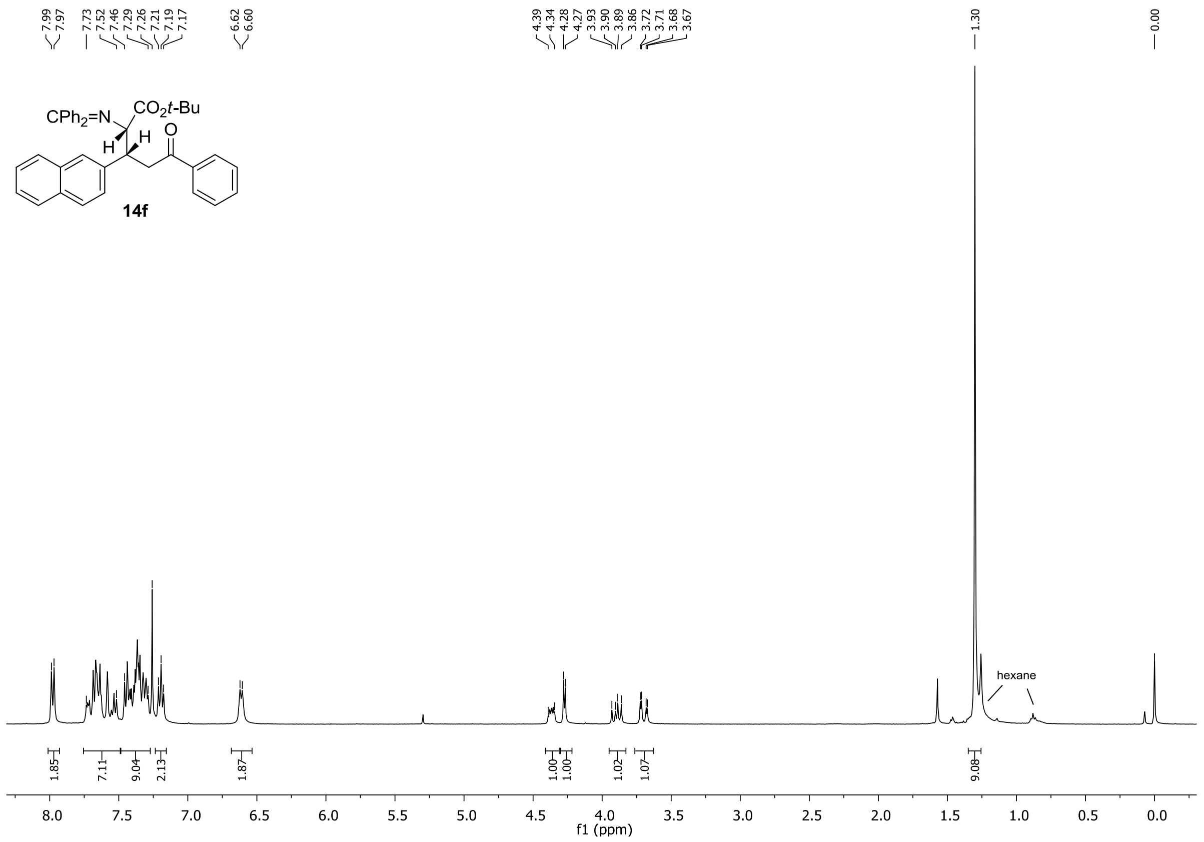

7.99
7.97
7.73
7.52
7.46
7.29
7.26
7.21
7.19
7.17
6.62
6.60
4.39
4.34
4.28
4.27
3.93
3.90
3.89
3.86
3.72
3.71
3.68
3.67
1.30
0.00
CPh2=N
CO2t-Bu
H
H
O
14f
hexane
1.85
7.11
9.04
2.13
1.87
1.00
1.00
1.02
1.07
9.08
f1 (ppm)

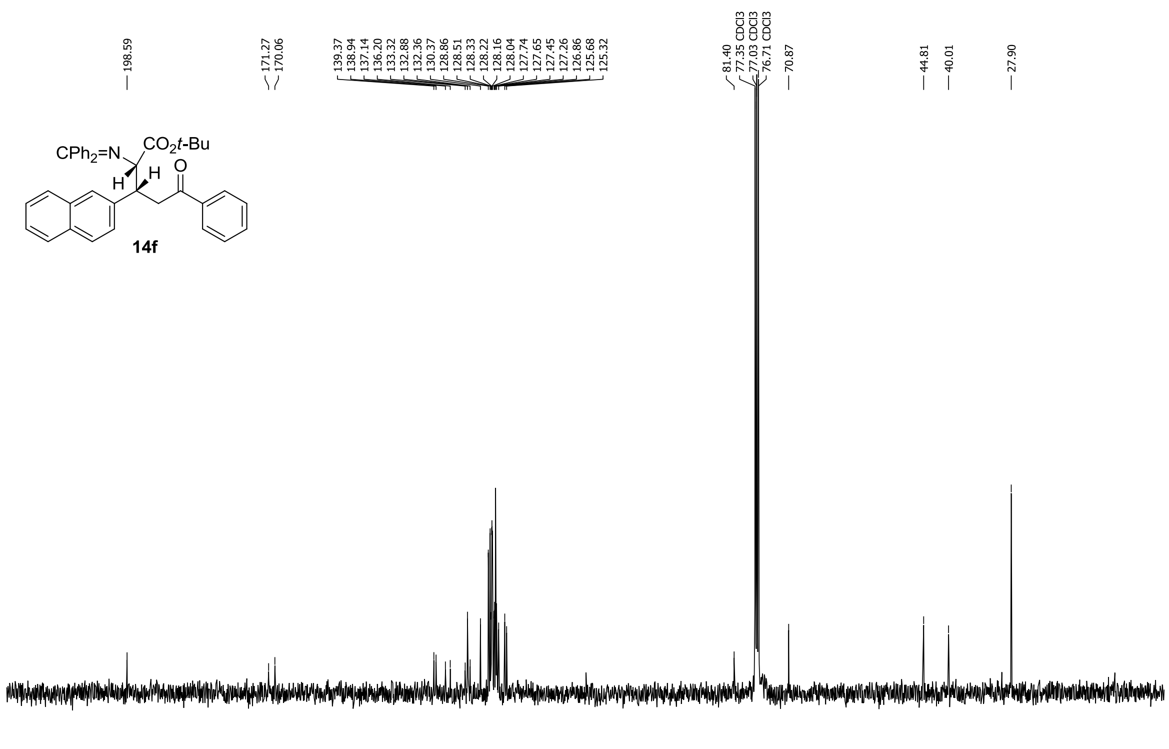

198.59
171.27
170.06
139.37
138.94
137.14
136.20
133.32
132.88
132.36
130.37
128.86
128.51
128.33
128.22
128.16
128.04
127.74
127.65
127.45
127.26
126.86
125.68
125.32
81.40
77.35 CDCl3
77.03 CDCl3
76.71 CDCl3
70.87
44.81
40.01
27.90
CPh2=N
CO2t-Bu
H
H
O
14f
f1 (ppm)

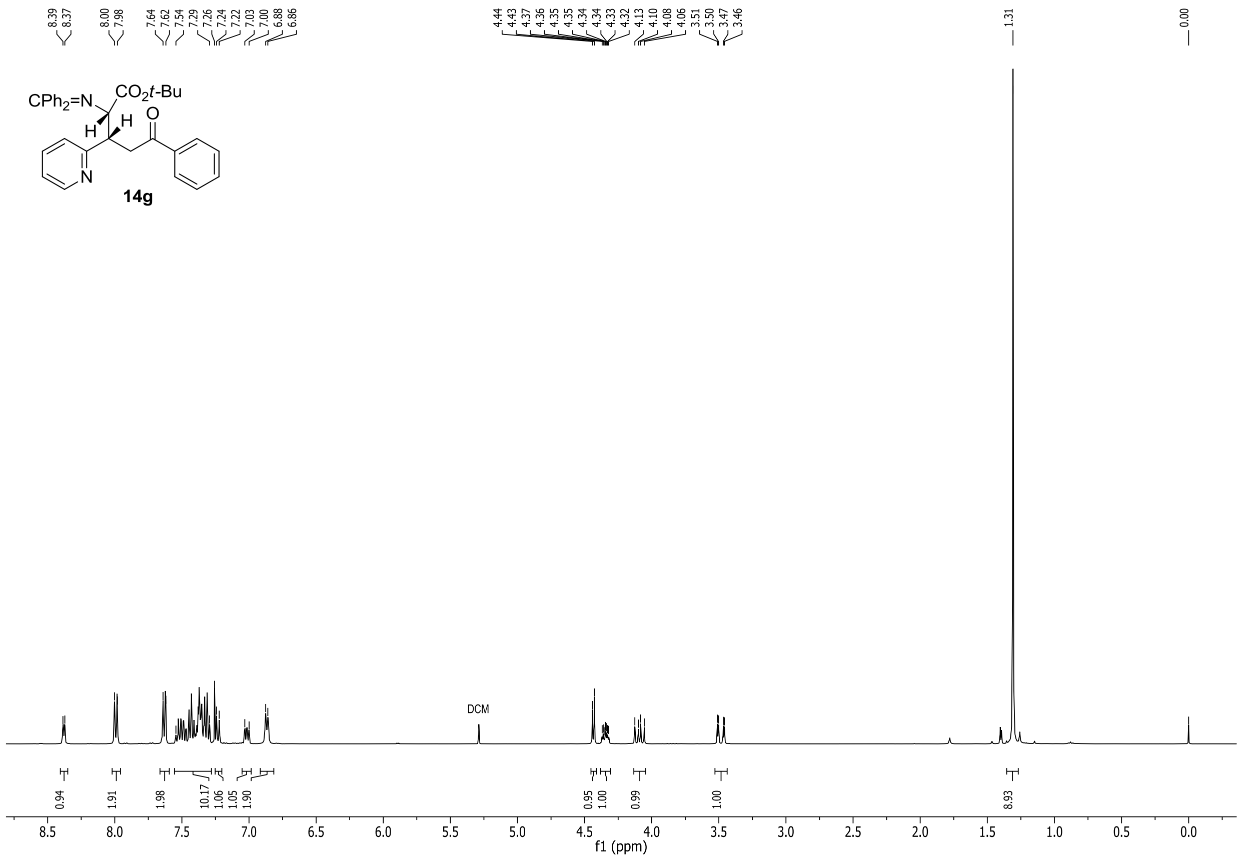
CPh2=N CO2t-Bu
14g
DCM
f1 (ppm)

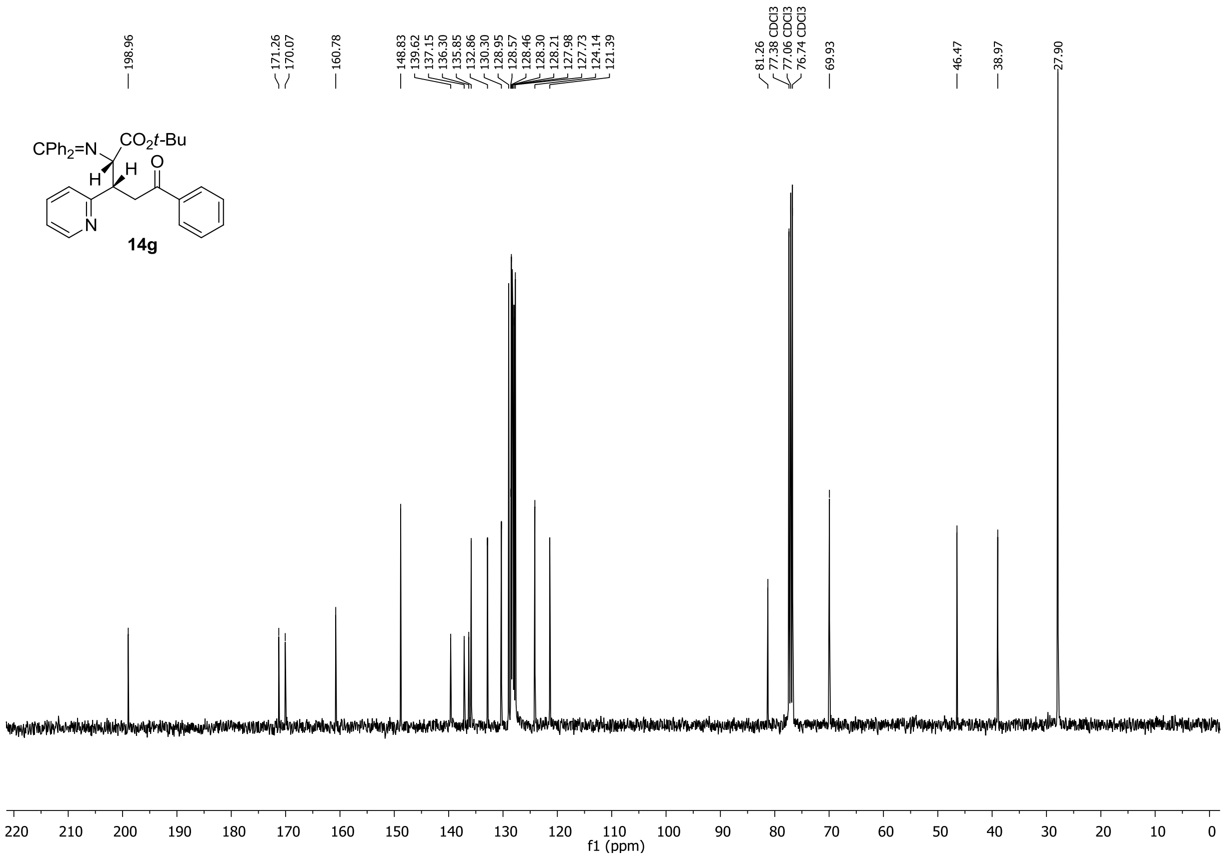
CPh2=N CO2t-Bu
14g
f1 (ppm)

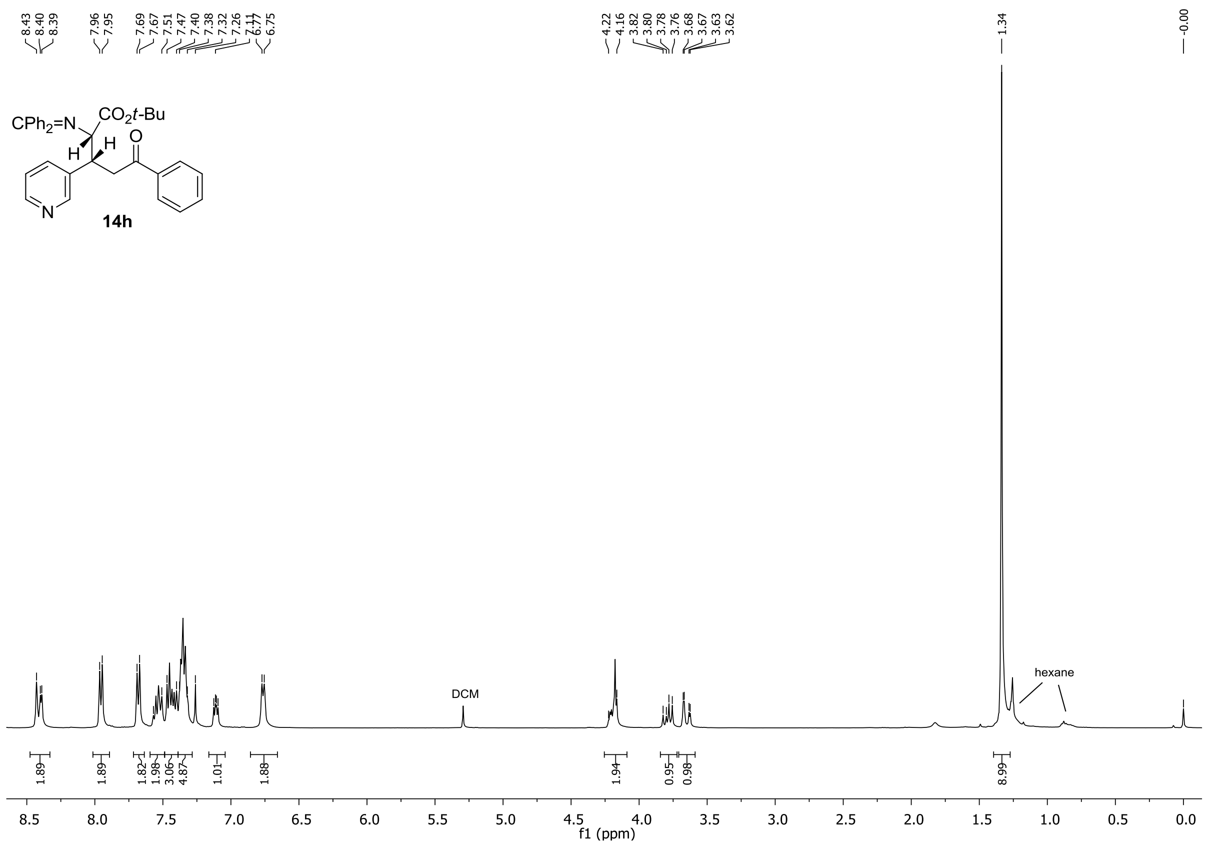
8.43
8.40
8.39
7.96
7.95
7.69
7.67
7.51
7.47
7.40
7.38
7.32
7.26
7.11
6.77
6.75
4.22
4.16
3.82
3.80
3.78
3.76
3.68
3.67
3.63
3.62
1.34
-0.00
CPh2=N
CO2t-Bu
H
H
O
N
14h
DCM
hexane
1.89
1.89
1.82
1.98
3.06
4.87
1.01
1.88
1.94
0.95
0.98
8.99
f1 (ppm)

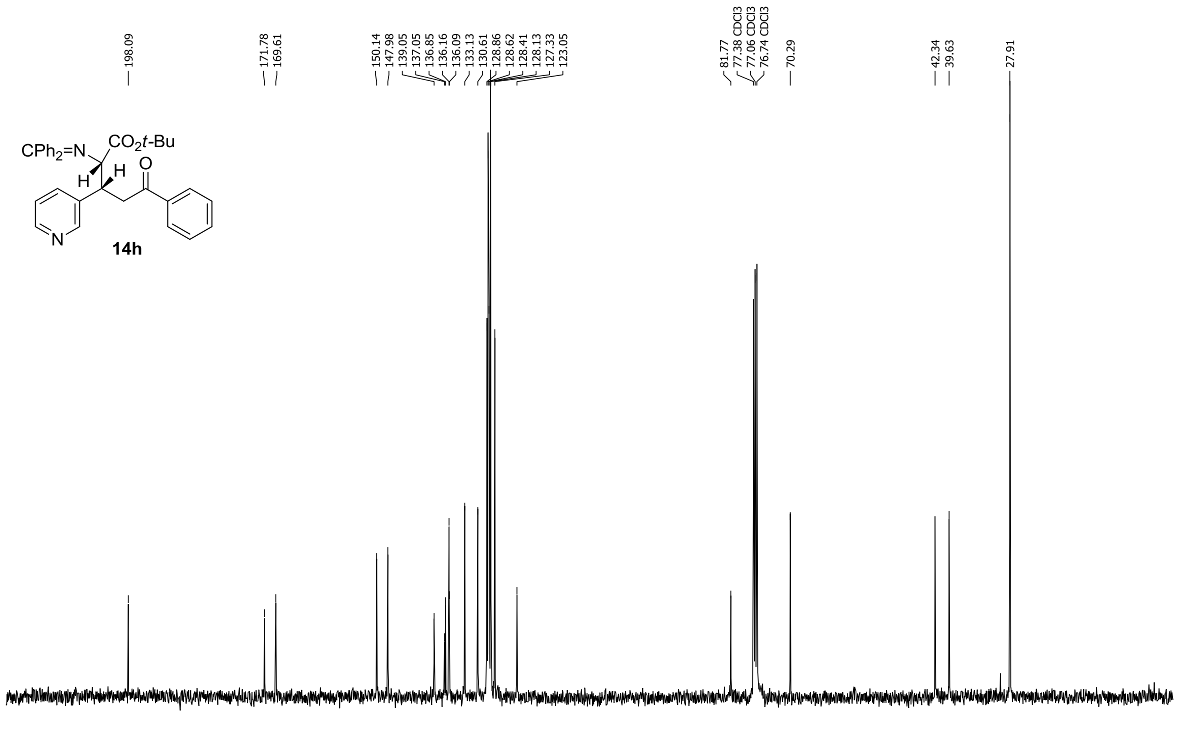
198.09
171.78
169.61
150.14
147.98
139.05
137.05
136.85
136.16
136.09
133.13
130.61
128.86
128.62
128.41
128.13
127.33
123.05
81.77
77.38 CDCl3
77.06 CDCl3
76.74 CDCl3
70.29
42.34
39.63
27.91
CPh2=N
CO2t-Bu
H
H
O
N
14h
f1 (ppm)

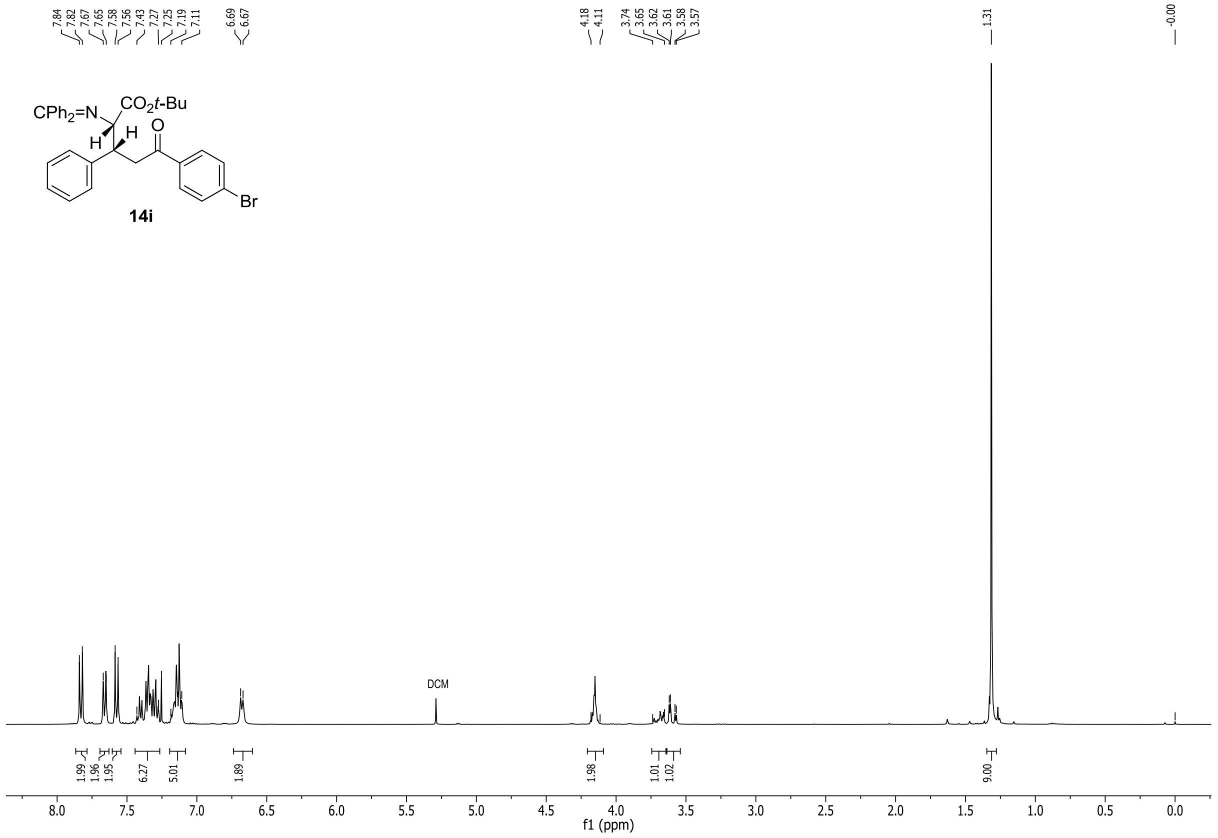
7.84
7.82
7.67
7.65
7.58
7.56
7.43
7.27
7.25
7.19
7.11
6.69
6.67
4.18
4.11
3.74
3.65
3.62
3.61
3.58
3.57
1.31
-0.00
CPh2=N
CO2t-Bu
H
H
O
Br
14i
DCM
1.99
1.96
1.95
6.27
5.01
1.89
1.98
1.01
1.02
9.00
f1 (ppm)

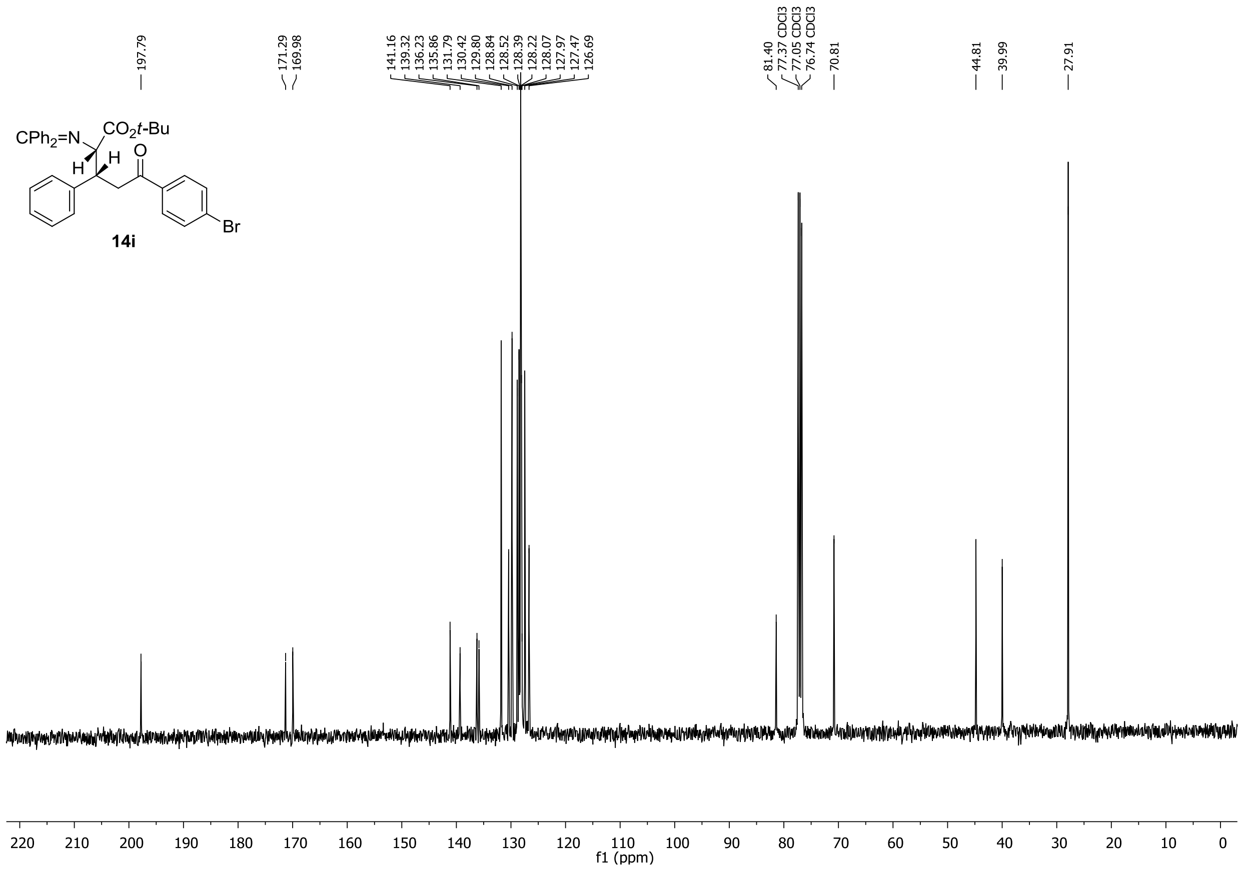
197.79
171.29
169.98
141.16
139.32
136.23
135.86
131.79
130.42
129.80
128.84
128.52
128.39
128.22
128.07
127.97
127.47
126.69
81.40
77.37 CDCl3
77.05 CDCl3
76.74 CDCl3
70.81
44.81
39.99
27.91
CPh2=N
CO2t-Bu
H
H
O
Br
14i
f1 (ppm)

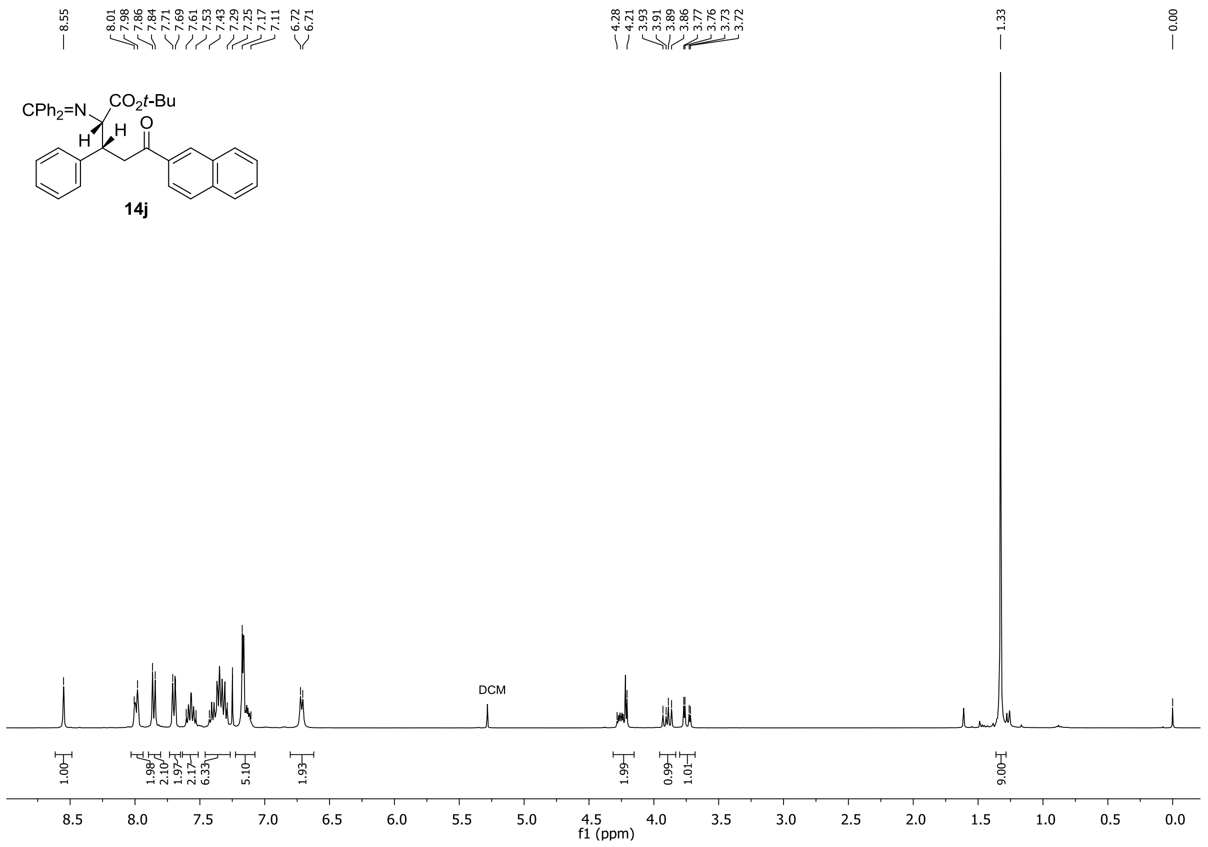
CPh2=N
CO2t-Bu
H
H
O
14j
DCM
f1 (ppm)

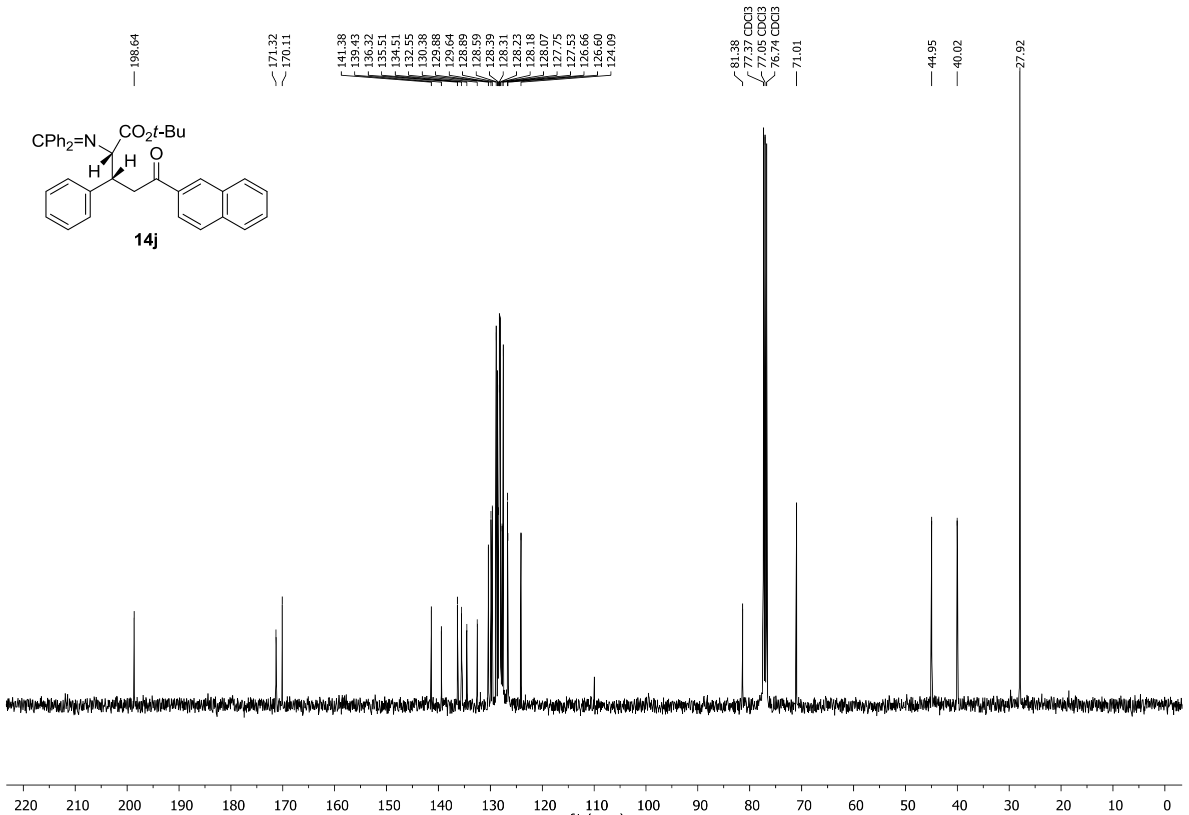
CPh2=N
CO2t-Bu
H
H
O
14j
CDCl3
f1 (ppm)

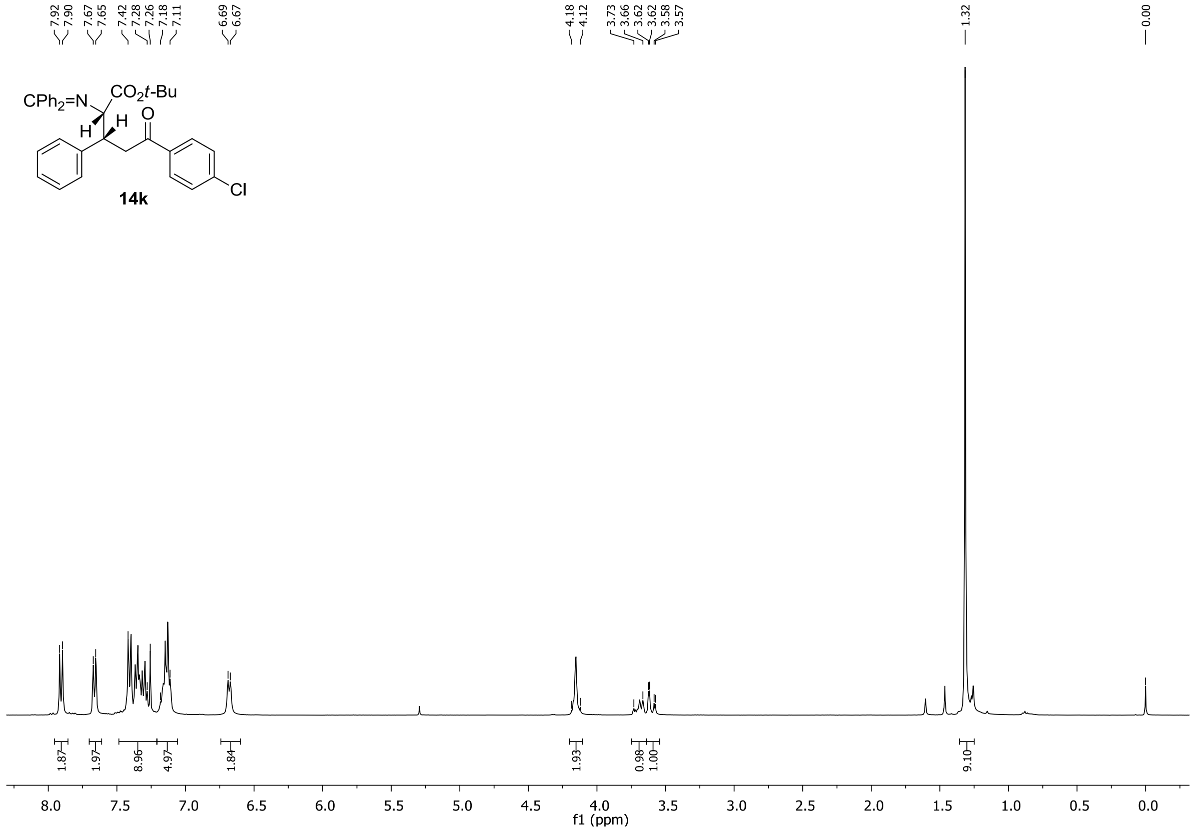

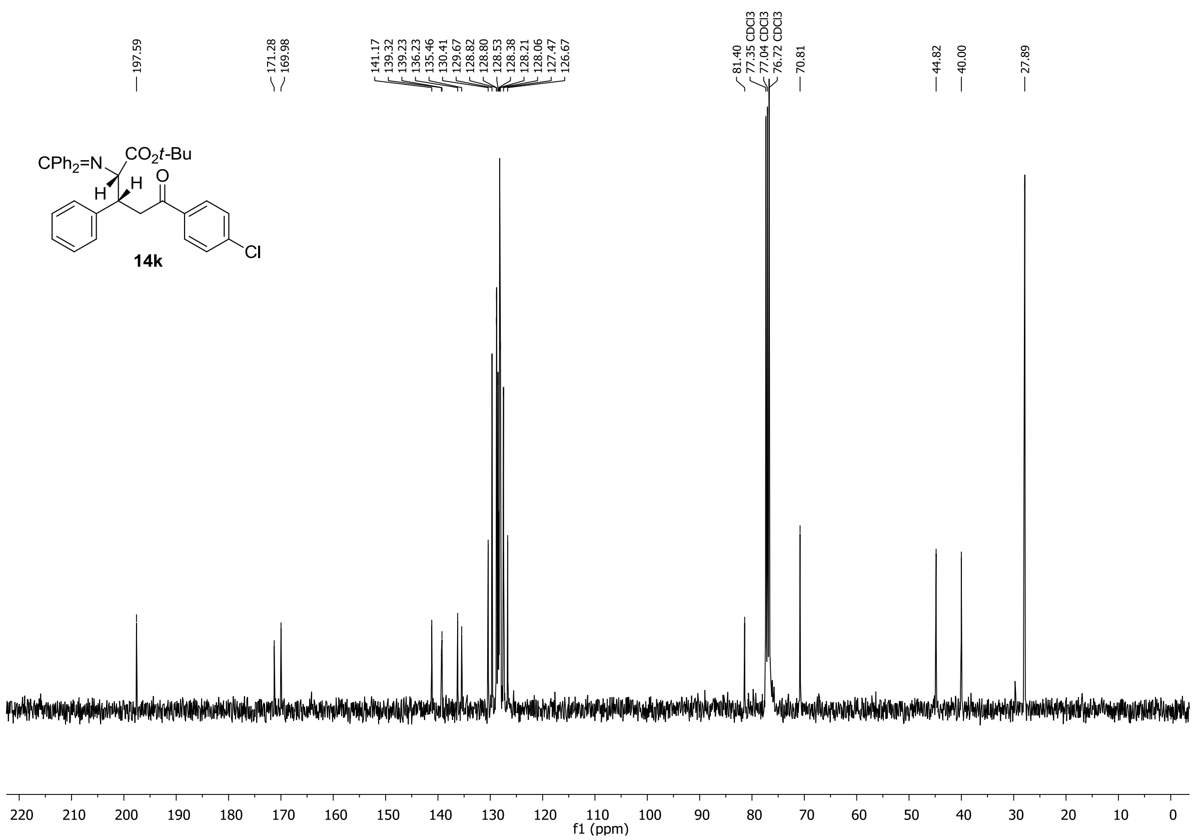

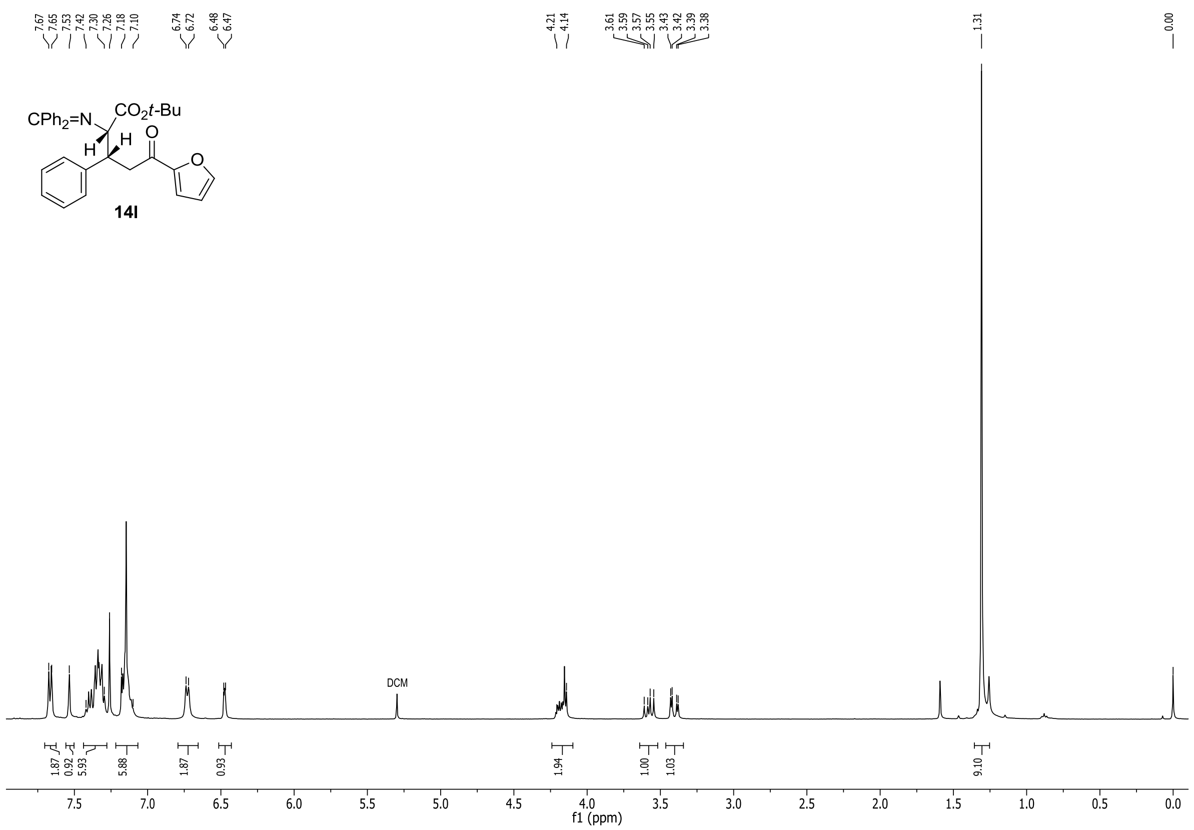
7.67
7.65
7.53
7.42
7.30
7.26
7.18
7.10
6.74
6.72
6.48
6.47
4.21
4.14
3.61
3.59
3.57
3.55
3.43
3.42
3.39
3.38
1.31
0.00
CPh2=N CO2t-Bu
H H O
O
14l
DCM
1.87
0.92
5.93
5.88
1.87
0.93
1.94
1.00
1.03
9.10
f1 (ppm)

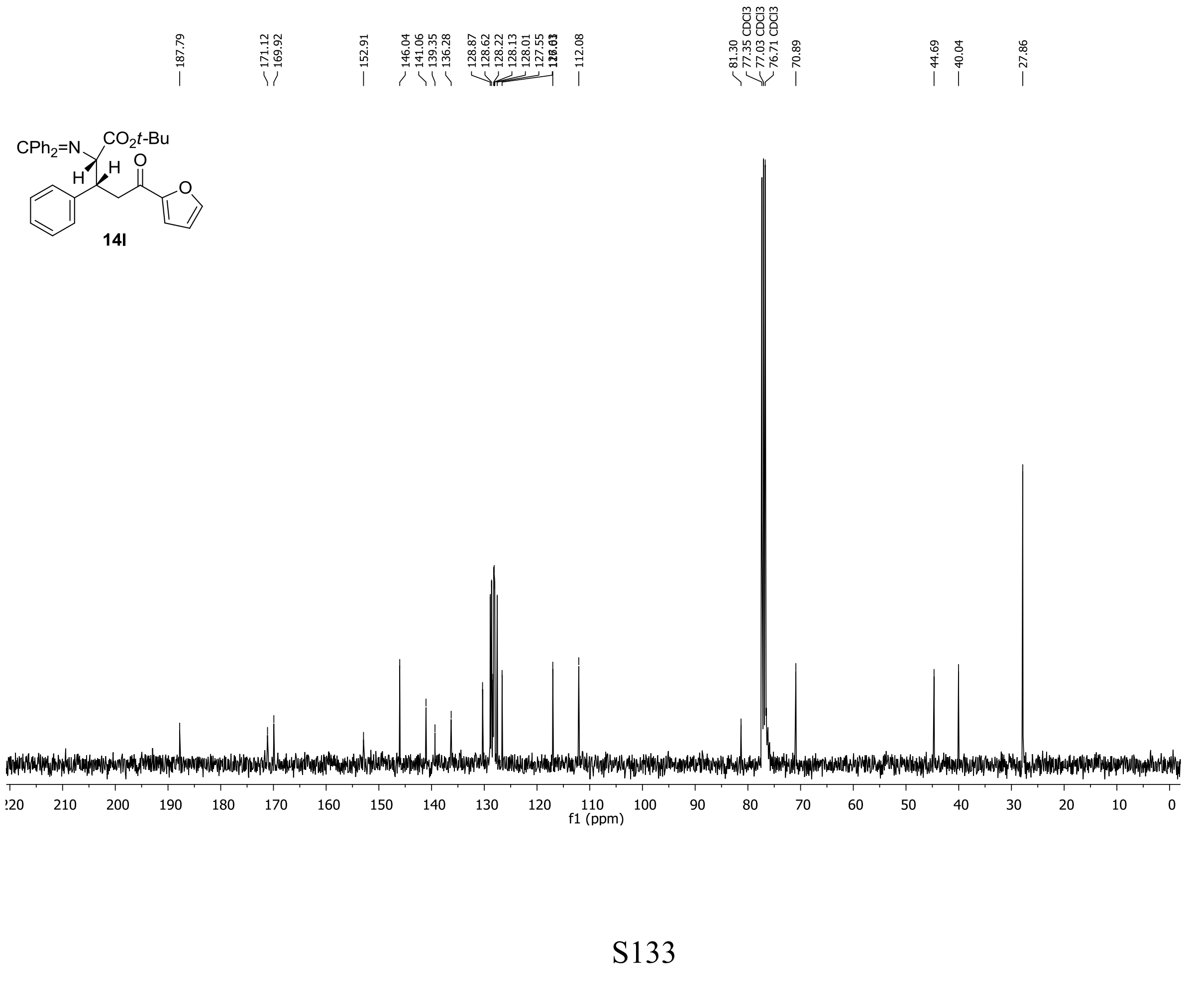
187.79
171.12
169.92
152.91
146.04
141.06
139.35
136.28
128.87
128.62
128.22
128.13
128.01
127.55
112.08
81.30
77.35 CDCl3
77.03 CDCl3
76.71 CDCl3
70.89
44.69
40.04
27.86
CPh2=N CO2t-Bu
H H O
O
14l
f1 (ppm)

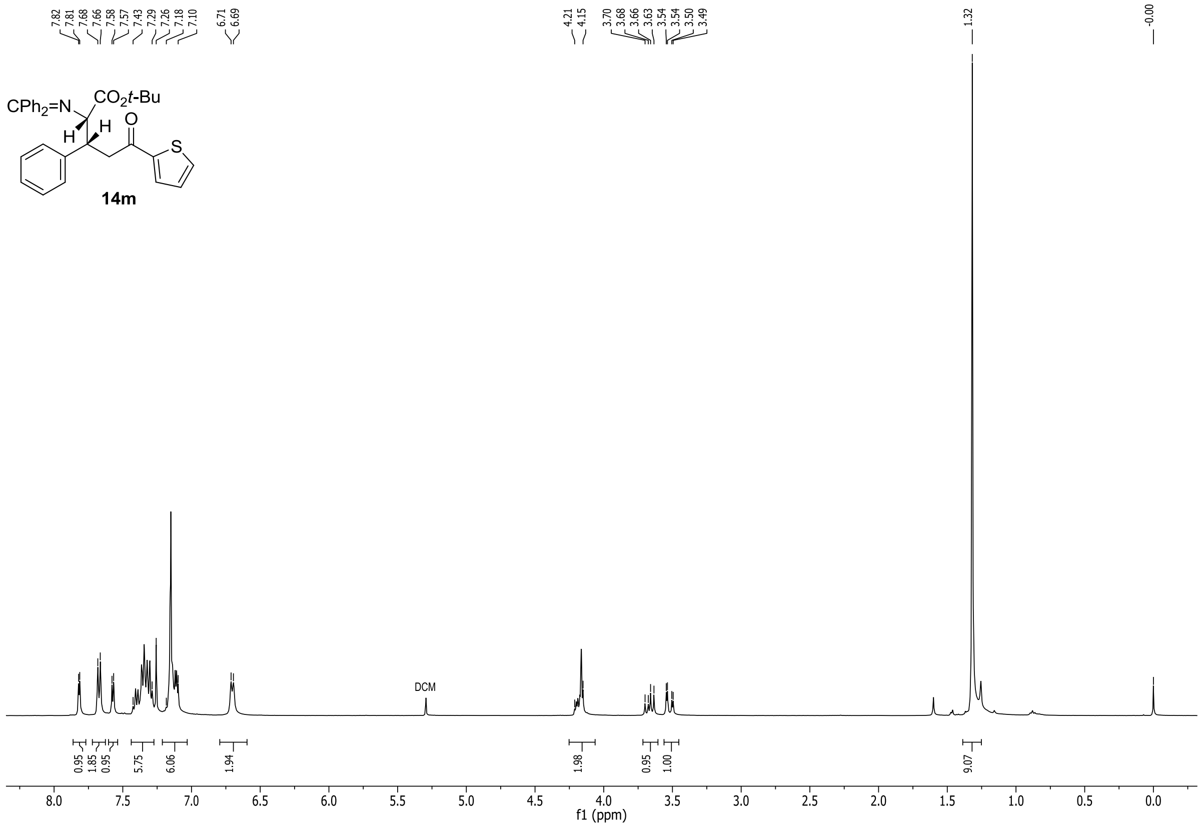

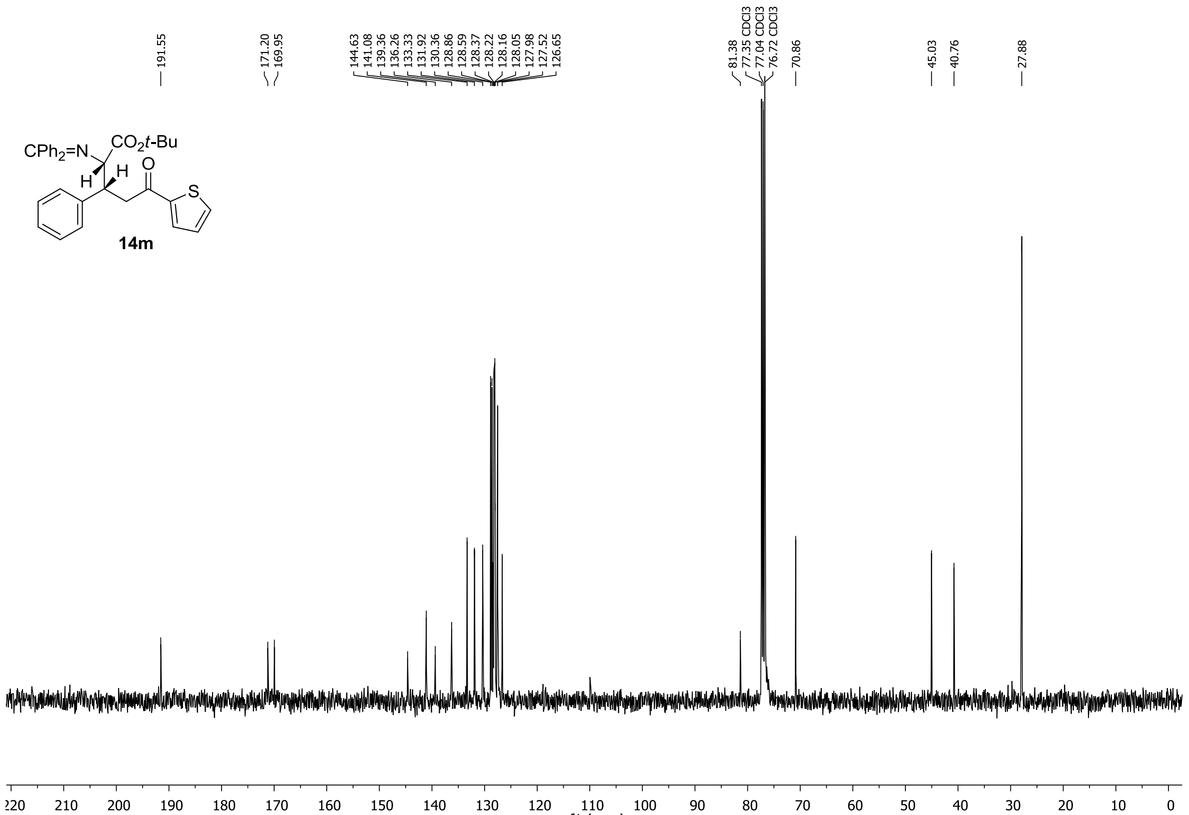

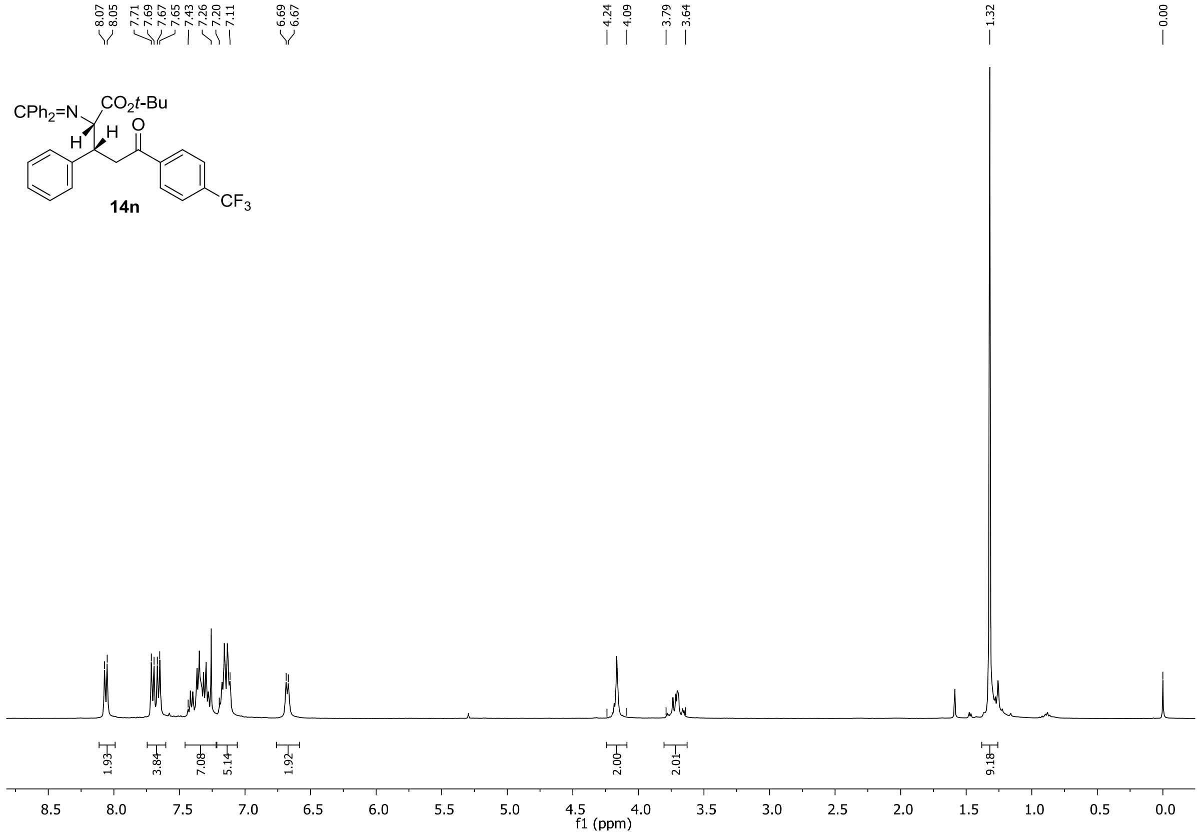
8.07
8.05
7.71
7.69
7.67
7.65
7.43
7.26
7.20
7.11
6.69
6.67
4.24
4.09
3.79
3.64
1.32
0.00
CPh2=N
CO2t-Bu
H
H
O
CF3
14n
1.93
3.84
7.08
5.14
1.92
2.00
2.01
9.18
f1 (ppm)

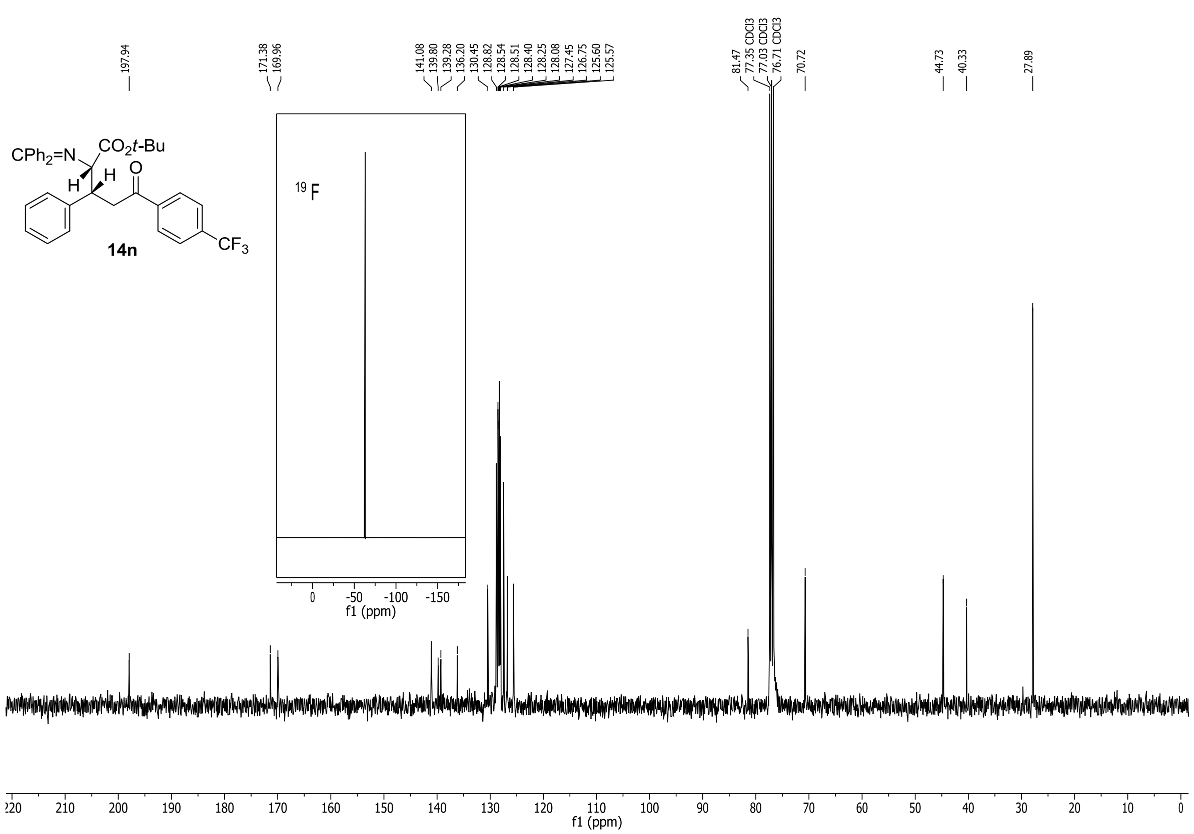
197.94
171.38
169.96
141.08
139.80
139.28
136.20
130.45
128.82
128.54
128.51
128.40
128.25
128.08
127.45
126.75
125.60
125.57
81.47
77.35 CDCl3
77.03 CDCl3
76.71 CDCl3
70.72
44.73
40.33
27.89
CPh2=N
CO2t-Bu
14n
CF3
19F
f1 (ppm)

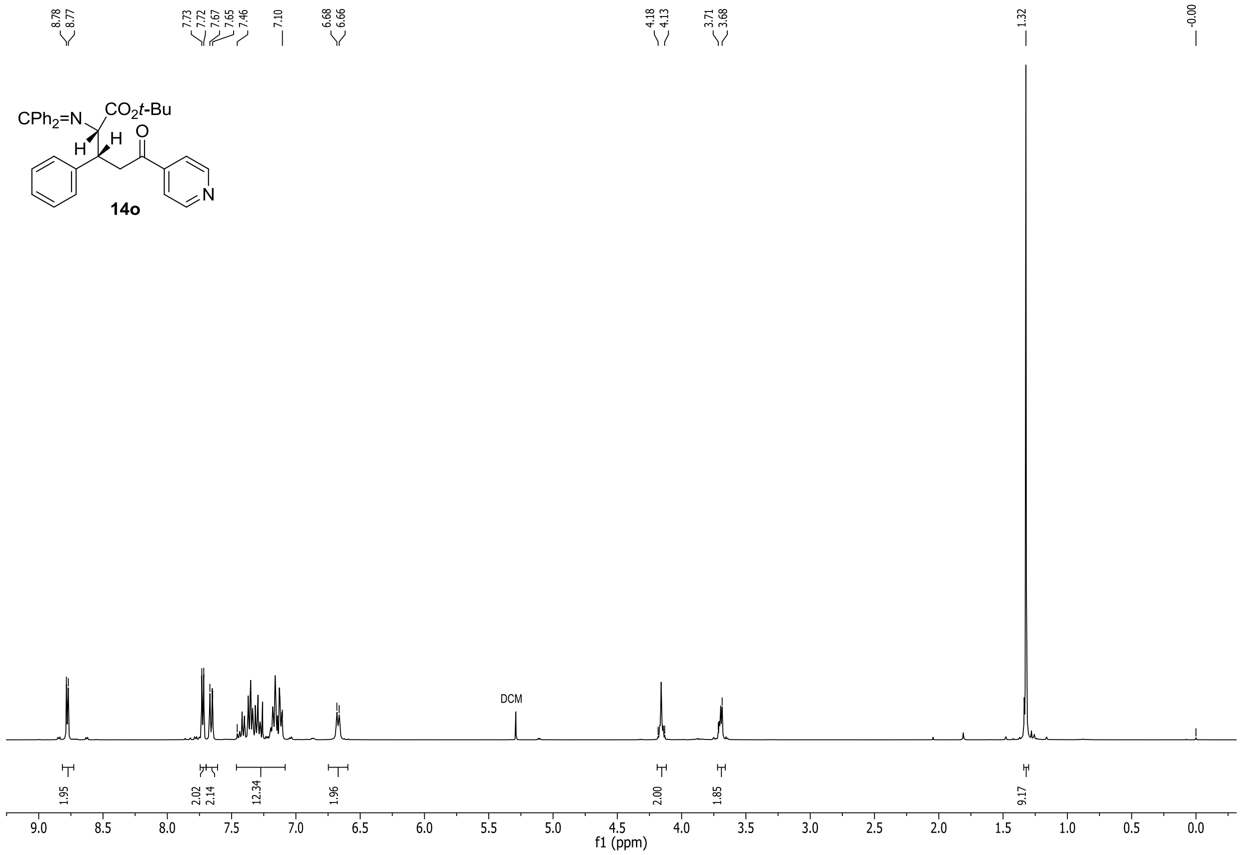

CPh2=N CO2t-Bu
14o
DCM
f1 (ppm)

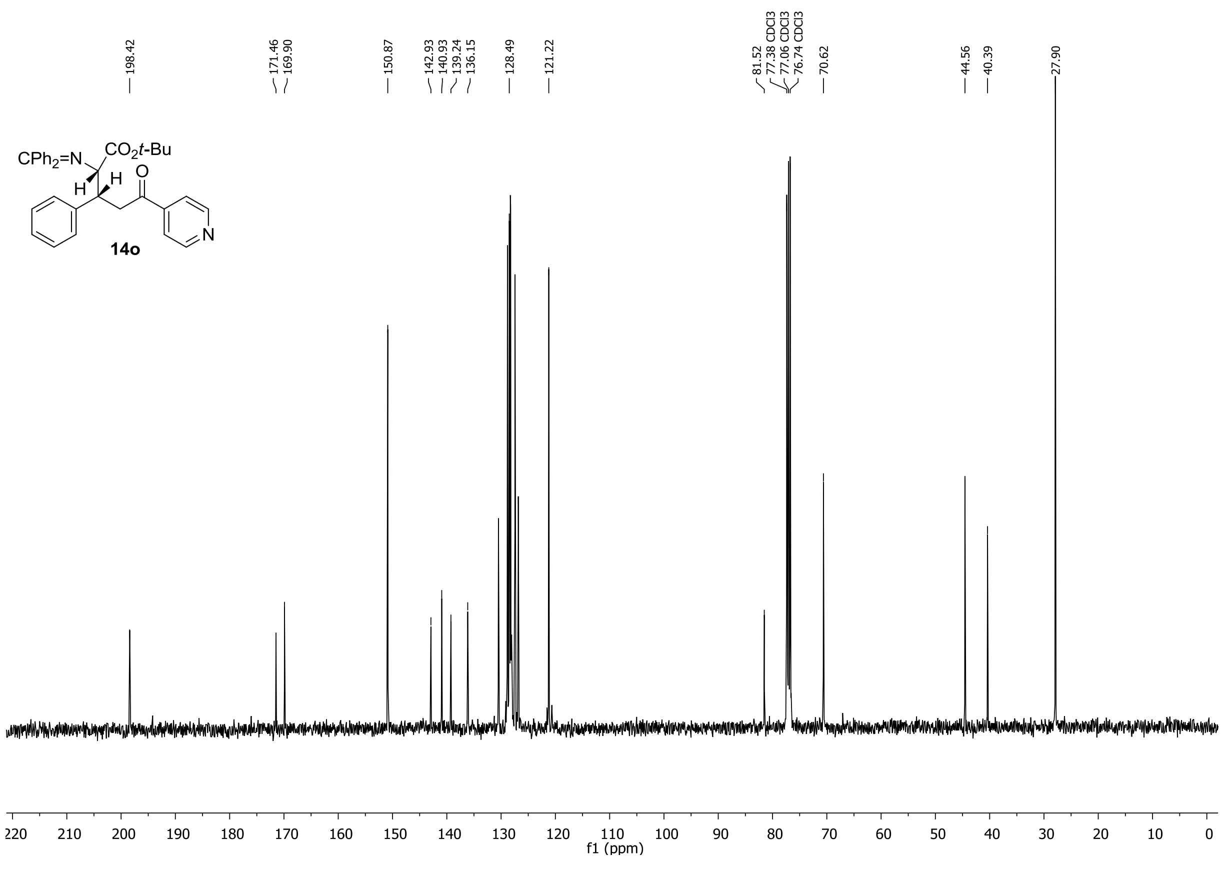

CPh2=N CO2t-Bu
14o
f1 (ppm)

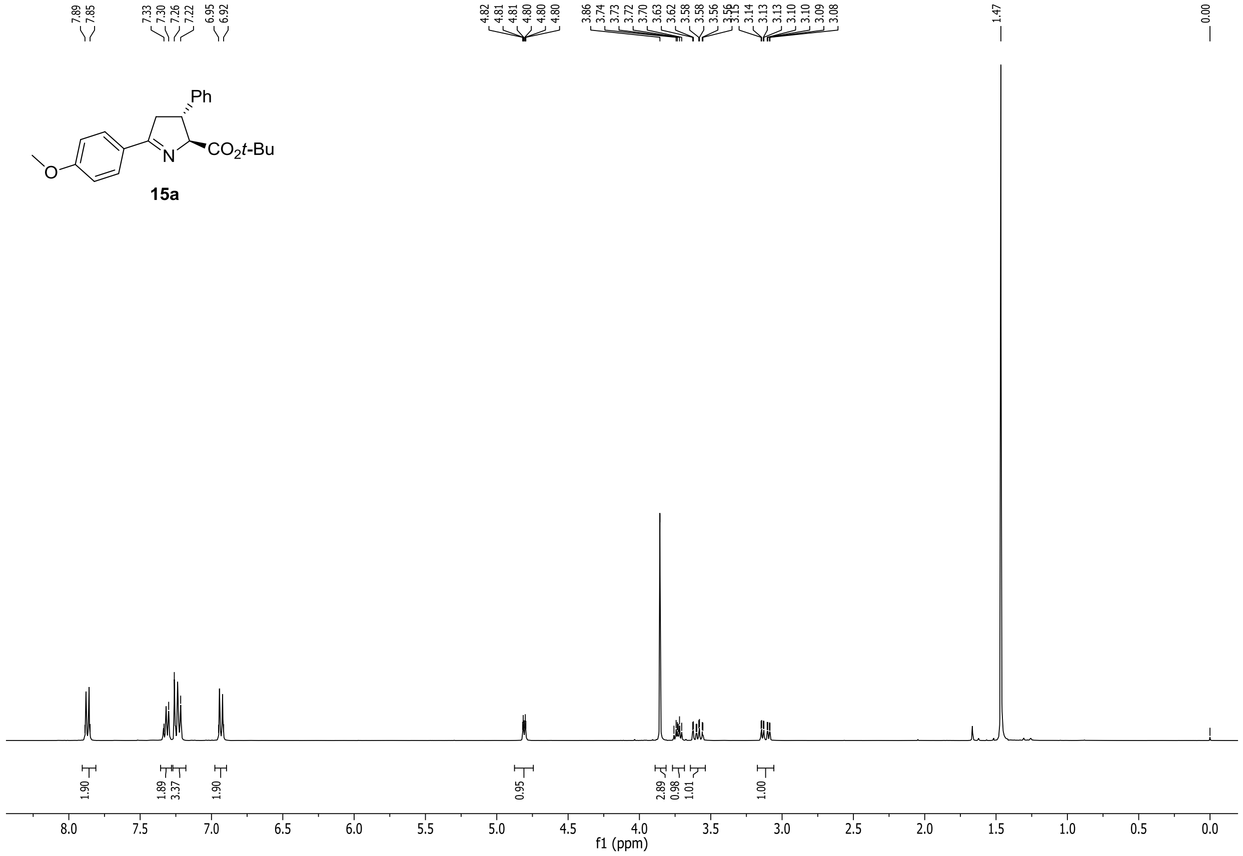
7.89
7.85
7.33
7.30
7.26
7.22
6.95
6.92
4.82
4.81
4.81
4.80
4.80
4.80
3.86
3.74
3.73
3.72
3.70
3.63
3.62
3.58
3.58
3.56
3.56
3.15
3.14
3.13
3.13
3.10
3.09
3.08
1.47
0.00
Ph
CO2t-Bu
N
O
15a
1.90
1.89
3.37
1.90
0.95
2.89
0.98
1.01
1.00
8.0 7.5 7.0 6.5 6.0 5.5 5.0 4.5 4.0 3.5 3.0 2.5 2.0 1.5 1.0 0.5 0.0
f1 (ppm)

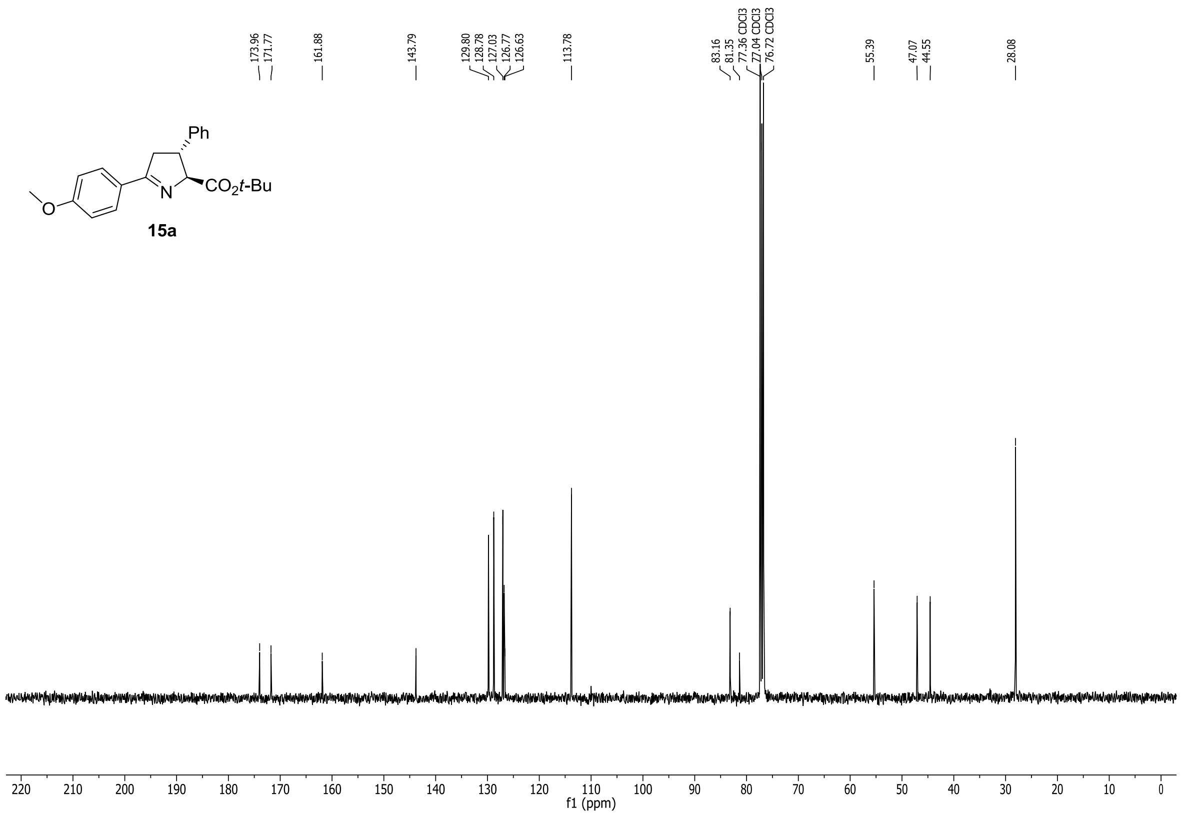
173.96
171.77
161.88
143.79
129.80
128.78
127.03
126.77
126.63
113.78
83.16
81.35
77.36 CDCl3
77.04 CDCl3
76.72 CDCl3
55.39
47.07
44.55
28.08
Ph
CO2t-Bu
N
O
15a
220 210 200 190 180 170 160 150 140 130 120 110 100 90 80 70 60 50 40 30 20 10 0
f1 (ppm)

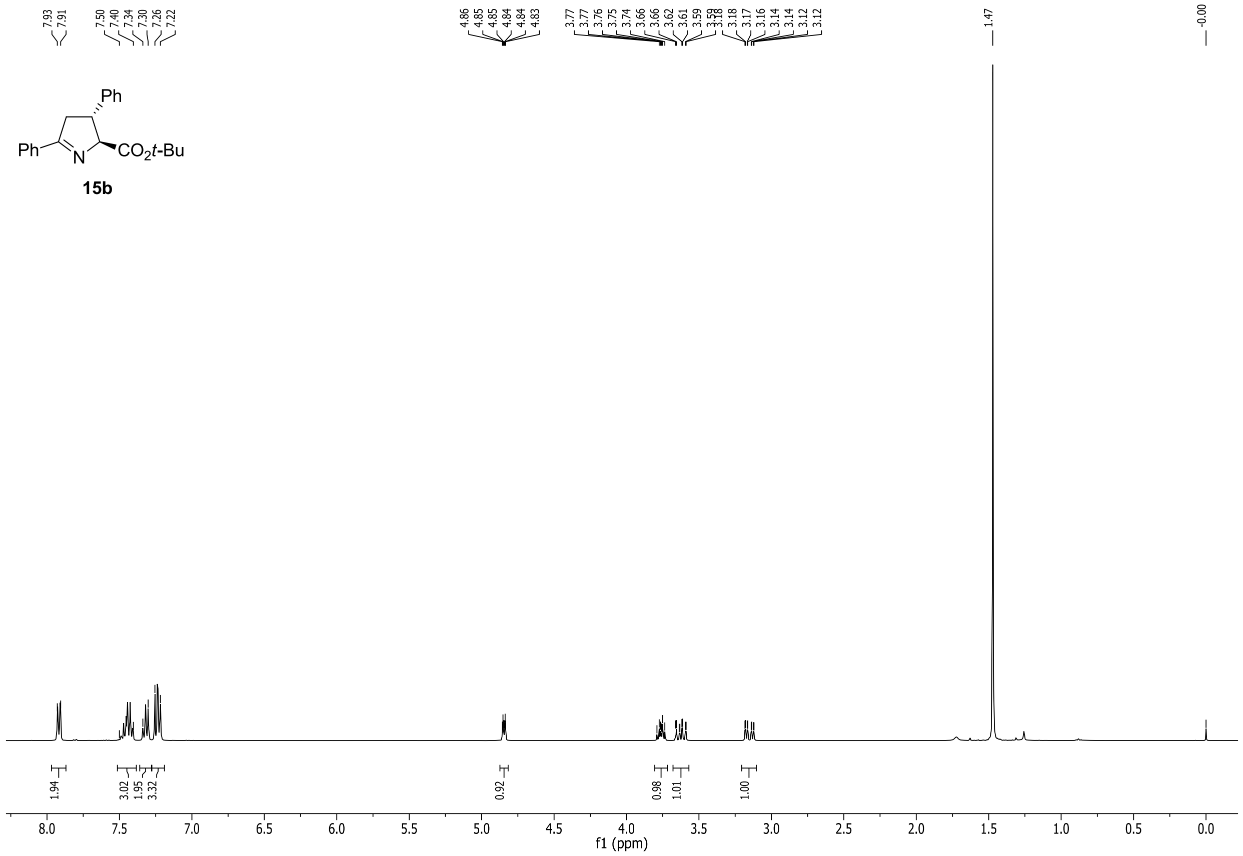

7.93 7.91 7.50 7.40 7.34 7.30 7.26 7.22
4.86 4.85 4.85 4.84 4.84 4.83
3.77 3.77 3.76 3.75 3.74 3.66 3.62 3.61 3.59 3.59 3.18 3.18 3.17 3.16 3.14 3.14 3.12 3.12
1.47
-0.00
Ph
Ph
N
CO2t-Bu
15b
1.94 3.02 1.95 3.32 0.92 0.98 1.01 1.00
8.0 7.5 7.0 6.5 6.0 5.5 5.0 4.5 4.0 3.5 3.0 2.5 2.0 1.5 1.0 0.5 0.0
f1 (ppm)

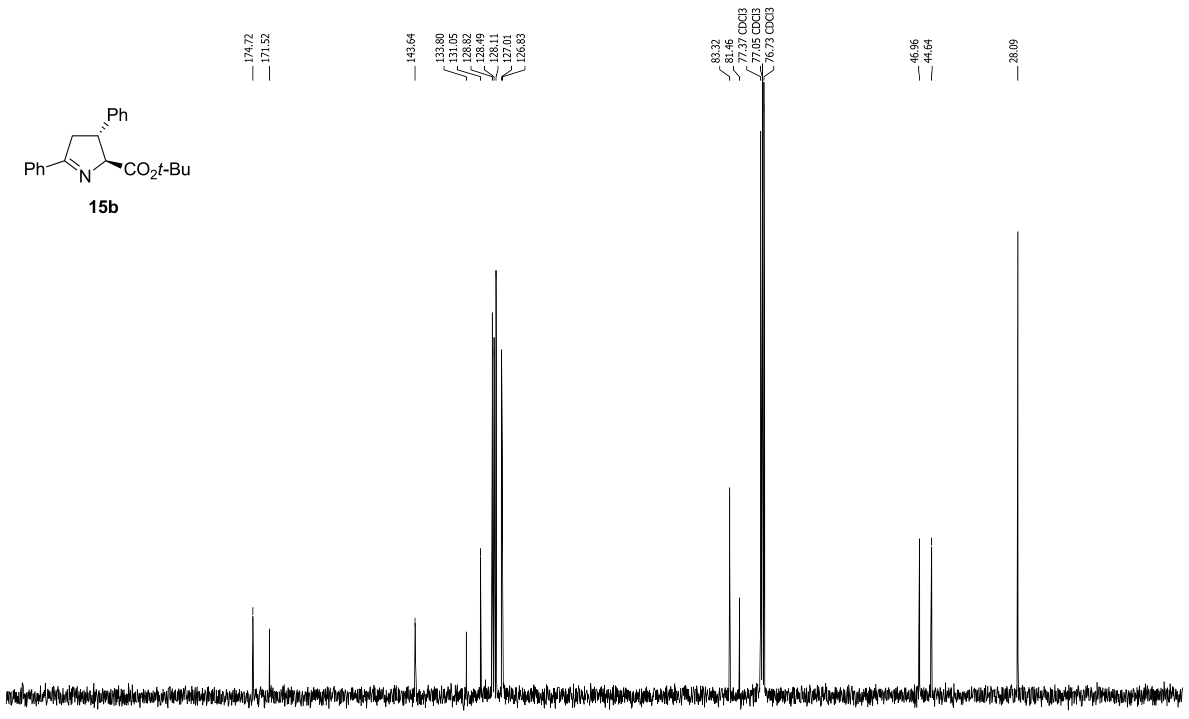

174.72 171.52
143.64
133.80 131.05 128.82 128.49 128.11 127.01 126.83
83.32 81.46 77.37 CDCl3 77.05 CDCl3 76.73 CDCl3
46.96 44.64
28.09
Ph
Ph
N
CO2t-Bu
15b
220 210 200 190 180 170 160 150 140 130 120 110 100 90 80 70 60 50 40 30 20 10 0
f1 (ppm)

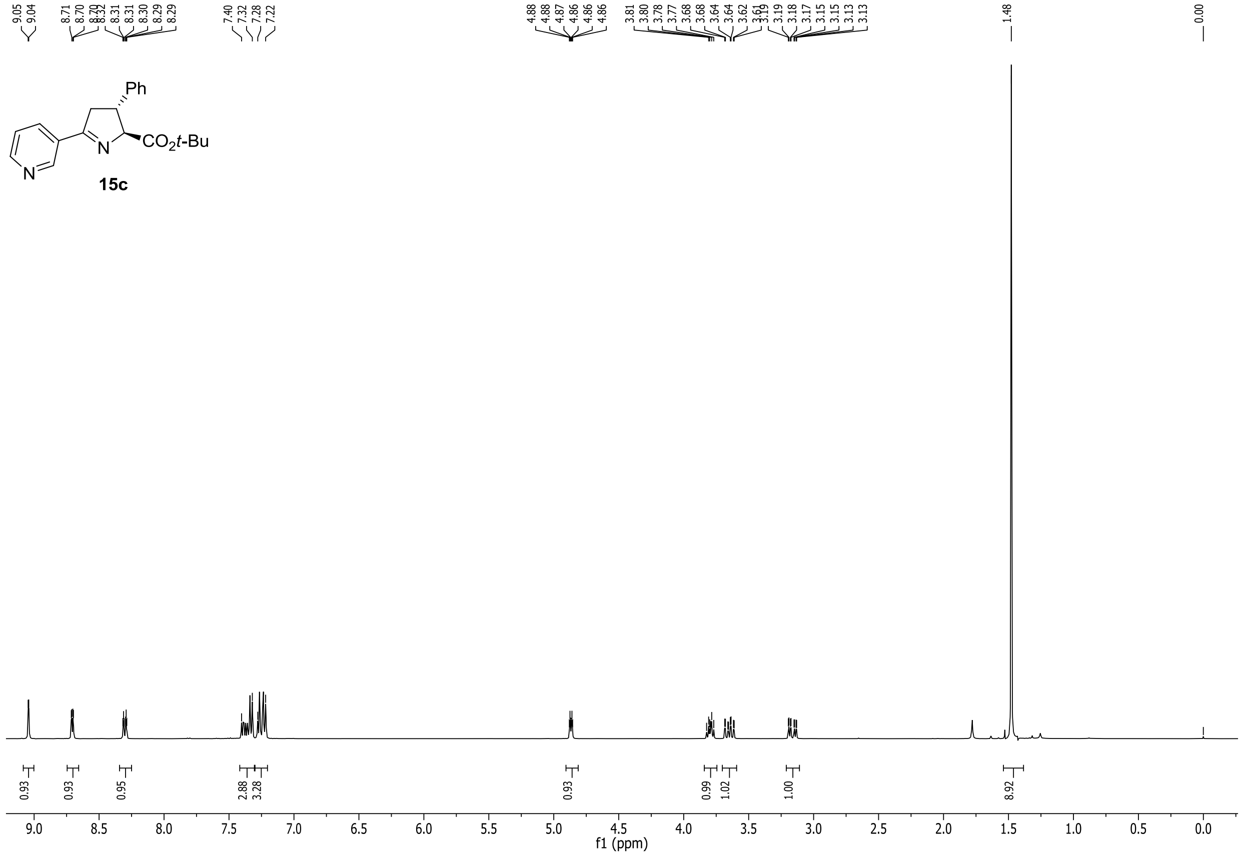
9.05
9.04
8.71
8.70
8.32
8.31
8.31
8.30
8.29
8.29
7.40
7.32
7.28
7.22
4.88
4.88
4.87
4.86
4.86
4.86
3.81
3.80
3.78
3.77
3.68
3.68
3.64
3.62
3.61
3.19
3.19
3.18
3.17
3.15
3.15
3.13
3.13
1.48
0.00
Ph
N
N
CO2t-Bu
15c
0.93
0.93
0.95
2.88
3.28
0.93
0.99
1.02
1.00
8.92
9.0 8.5 8.0 7.5 7.0 6.5 6.0 5.5 5.0 4.5 4.0 3.5 3.0 2.5 2.0 1.5 1.0 0.5 0.0
f1 (ppm)

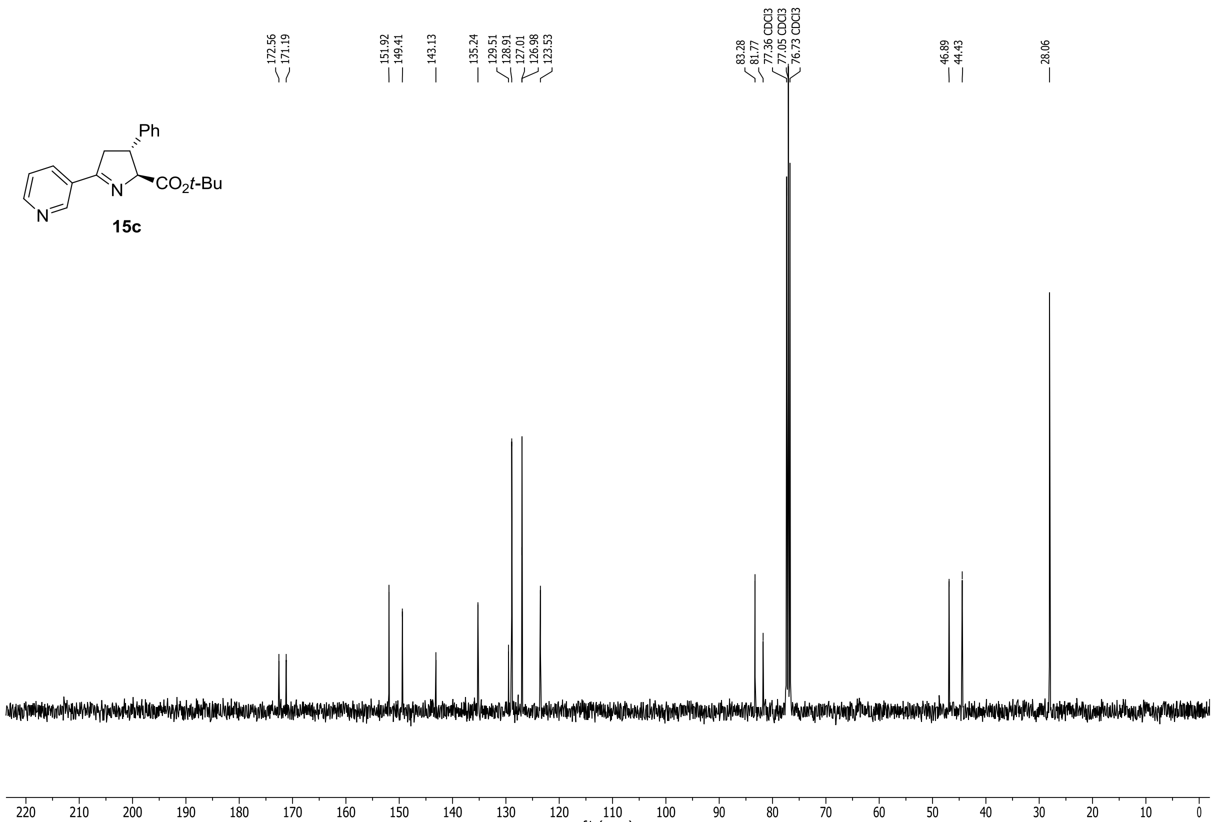
172.56
171.19
151.92
149.41
143.13
135.24
129.51
128.91
127.01
126.98
123.53
83.28
81.77
77.36 CDCl3
77.05 CDCl3
76.73 CDCl3
46.89
44.43
28.06
Ph
N
N
CO2t-Bu
15c
220 210 200 190 180 170 160 150 140 130 120 110 100 90 80 70 60 50 40 30 20 10 0
f1 (ppm)

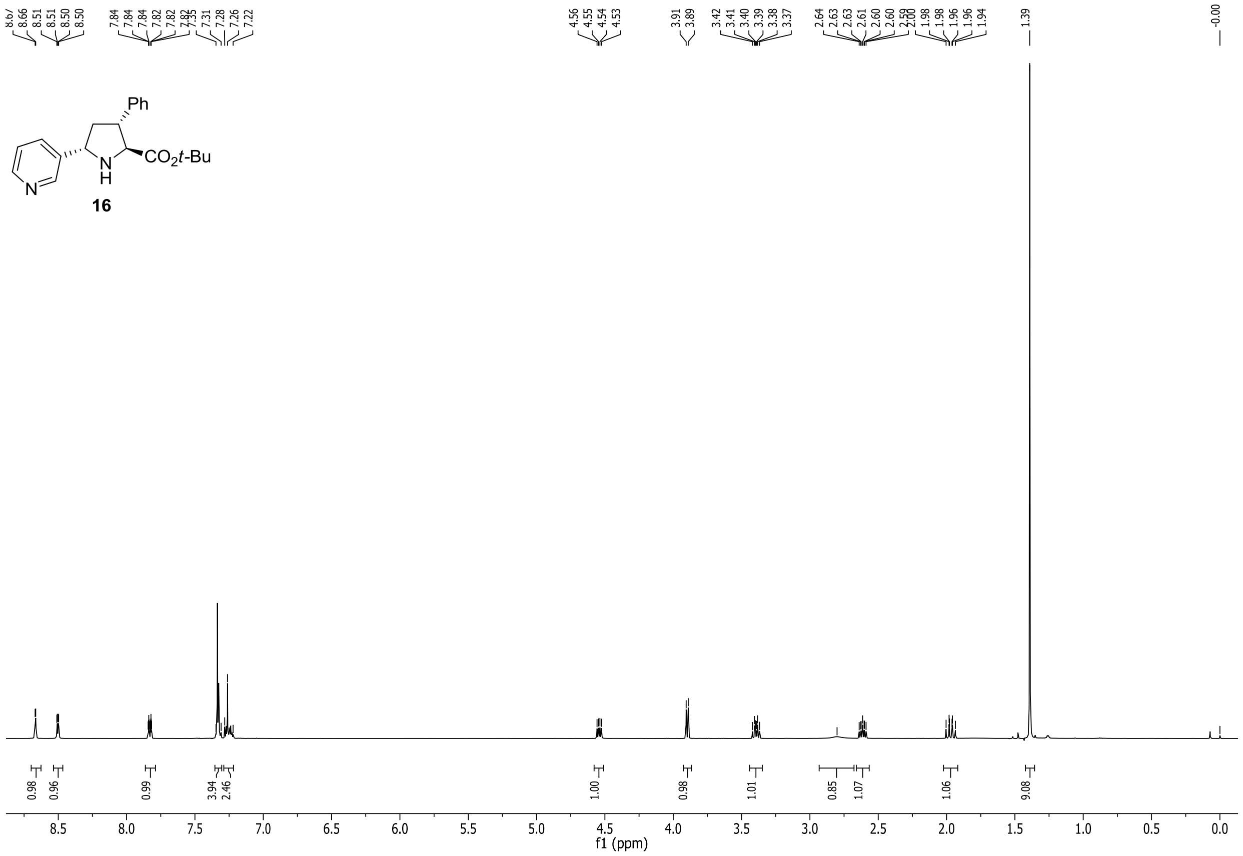
Ph
N
H
CO$_2$t-Bu
16
4.56
4.55
4.54
4.53
3.91
3.89
3.42
3.41
3.40
3.39
3.38
3.37
1.39
-0.00
0.98
0.96
0.99
3.94
2.46
1.00
0.98
1.01
0.85
1.07
1.06
9.08
f1 (ppm)

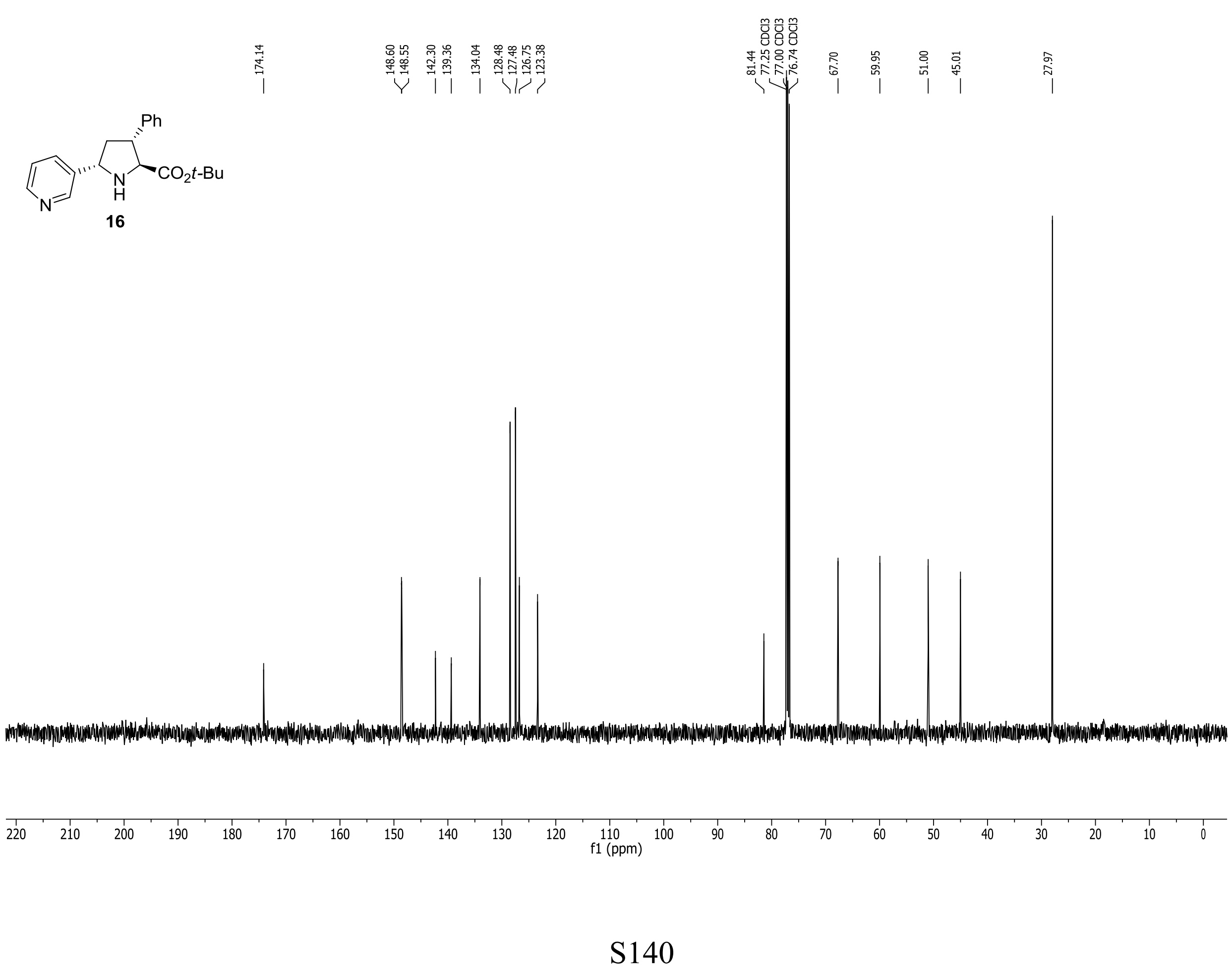
Ph
N
H
CO$_2$t-Bu
16
174.14
148.60
148.55
142.30
139.36
134.04
128.48
127.48
126.75
123.38
81.44
77.25 CDCl3
77.00 CDCl3
76.74 CDCl3
67.70
59.95
51.00
45.01
27.97
f1 (ppm)
